# Deforestation and Carbon Stock Loss in Brazil’s Amazonian Settlements

**DOI:** 10.1007/s00267-016-0783-2

**Published:** 2016-10-24

**Authors:** Aurora Miho Yanai, Euler Melo Nogueira, Paulo Maurício Lima de Alencastro Graça, Philip Martin Fearnside

**Affiliations:** 1 Department of Environmental Dynamics, National Institute for Research in Amazonia (INPA), Av. André Araújo, 2936, Manaus, Amazonas 69067-375 Brazil; 2 Environmental Services sub-network, Brazilian Research Network on Climate Change (RedeClima), Av. André Araújo, 2936, Manaus, Amazonas 69067-375 Brazil

**Keywords:** Agrarian reform, Settlement project, Colonization, Carbon, Amazon forest, Global warming

## Abstract

We estimate deforestation and the carbon stock in 2740 (82 %) of the 3325 settlements in Brazil’s Legal Amazonia region. Estimates are made both using available satellite data and a carbon map for the “pre-modern” period (prior to 1970). We used data from Brazil’s Project for Monitoring Deforestation in Amazonia updated through 2013 and from the Brazilian Biomes Deforestation Monitoring Project (PMDBBS) updated through 2010. To obtain the pre-modern and recent carbon stocks we performed an intersection between a carbon map and a map derived from settlement boundaries and deforestation data. Although the settlements analyzed occupied only 8 % of Legal Amazonia, our results indicate that these settlements contributed 17 % (160,410 km^2^) of total clearing (forest + non-forest) in Legal Amazonia (967,003 km^2^). This represents a clear-cutting of 41 % of the original vegetation in the settlements. Out of this total, 72 % (115,634 km^2^) was in the “Federal Settlement Project” (PA) category. Deforestation in settlements represents 20 % (2.6 Pg C) of the total carbon loss in Legal Amazonia (13.1 Pg C). The carbon stock in remaining vegetation represents 3.8 Pg C, or 6 % of the total remaining carbon stock in Legal Amazonia (58.6 Pg C) in the periods analyzed. The carbon reductions in settlements are caused both by the settlers and by external actors. Our findings suggest that agrarian reform policies contributed directly to carbon loss. Thus, the implementation of new settlements should consider potential carbon stock losses, especially if settlements are created in areas with high carbon stocks.

## Introduction

Historically, the movement of landless families to Brazilian Legal Amazonia (henceforth referred to as “Legal Amazonia”) was driven by government programs such as the National Integration Program (PIN) in the 1970s. “Legal Amazonia” is a 5.1 million-km^2^ administrative area decreed in 1953; roughly three-quarters of this region was originally Amazon forest and one-quarter non-forest vegetation such as central Brazilian savanna (*cerrado*). The PIN featured construction of major roads (e.g., the Transamazon Highway) and colonization along these roads (Brazil, PR 1970; Fearnside 1986a; Kohlhepp 2002). Since then, Legal Amazonia has been the target of a succession of settlement policies.

Brazil’s Amazonian settlements comprise mainly landless family farmers from southern and southeastern Brazil (Caviglia-Harris et al. 2013; Fearnside 2008). Additionally, there are areas where farmers migrated from consolidated frontier regions (e.g., Rondônia and Mato Grosso) to settlements located in areas of frontier expansion (e.g., southern Amazonas and southern Pará) (Carrero and Fearnside 2011). In both cases, the aim is to receive a permission to occupy a piece of land and later to gain title to it. Brazil’s National Institute for Colonization and Agrarian Reform (INCRA) classifies federal settlements into two groups: “traditional” and “environmentally distinctive” (Brazil, INCRA 2015a). Traditional settlements are characterized by division into properties (*lotes*) where, after some years, the settlers receive property titles and can manage the land with more autonomy (Brazil, INCRA 2013). Federal Settlement Projects (PAs = *Projetos de Assentamento Federal*) are currently the predominant form of “traditional” settlement (Brazil, INCRA 2015a), but other settlement models followed similar patterns in the past, such as Integrated Colonization Projects (PICs = *Projetos Integrados de Colonização*) and Directed Settlement Projects (PADs = *Projetos de Assentamento Dirigido*) (Fearnside 1986b). Since 1999, traditional settlements can only be installed in areas with some previous deforestation (Brazil, MEPF 1999) because the most common activities developed in traditional settlements are agriculture and cattle ranching.

**Fig. 1:**
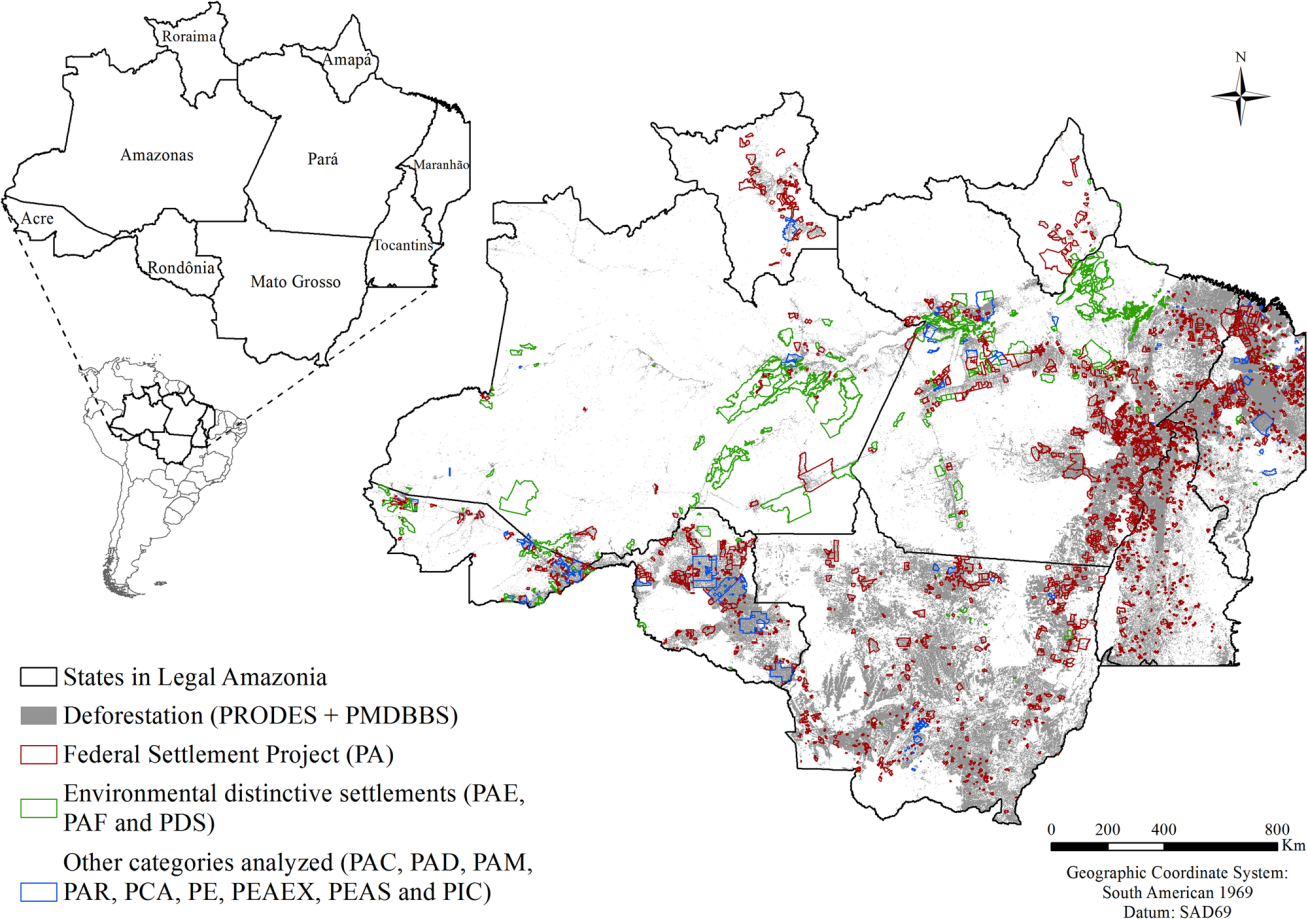
Brazil’s Legal Amazonia region showing deforestation (PRODES through 2013 and PMDBBS through 2010) and settlements classified by category as “traditional” or “environmentally distinctive”. See categories of settlements detailed in Table 1

The environmentally distinctive settlements are destined for traditional populations for activities with low deforestation impact, such as agro-extractive activities and sustainable forest management. Agro-Extractivist Settlement Projects (PAEs = *Projetos de Assentamento Agroextrativista*), Sustainable Development Projects (PDSs = *Projetos de Desenvolvimento Sustentável*) and Forest Settlement Projects (PAFs = *Projetos de Assentamento Florestal*) are federal environmentally distinctive settlements (Brazil, INCRA 2012) (Fig. 1).

Implementation of traditional settlements can occur (i) by the government distributing land for colonization in the specific area planned for a settlement, or (ii) by either redistribution or “regularization” of previously occupied land (Duchelle et al. 2014). In the first case, high-level authorities decide to create a settlement and choose where the settlement will be created (public land or private land acquired for agrarian reform). This form of settlement occurred mainly during the colonization process in the 1970s (Fearnside 1986a). In the second form of implementation, the land is previously occupied by landless people linked with social movement organizations (e.g., the MST: Landless Rural Workers Movement). In this case, the settlement would not necessarily be implemented in the area occupied initially: INCRA can choose a new site to allocate these families (Simmons et al. 2010).

This second form of settlement stems from the process known as “spontaneous occupation”. This type of occupation is related to agricultural expansion and consists of individuals (i.e., *posseiros*) who gradually occupy non-designated public lands (i.e., *terras devolutas*) by clearing areas in a pattern similar to that in official settlements with the aimed of facilitating the recognition of land by INCRA. This type of occupation does not have a political objective, as in the case of social movement organizations such as the Landless Rural Workers Movement (Caldas et al. 2010; Simmons et al. 2010).

In the case of environmentally distinctive settlements, some differences exist between the categories. In the PDS and PAF categories, the area selected for implementing the settlement has to be public (federal, state, or municipal) (Brazil, INCRA 1999, 2003). In the PAE category there is no such requirement (Brazil, INCRA 1987). Environmentally distinctive settlements can be installed in areas of primary forest, whether or not the areas have previously been inhabited by traditional populations. Settlers only receive a concession for use of the land, which means that they do not receive title to a *lote* as in traditional settlements. In PAF settlements, the areas to be used for forestry production can be used individually, in community or a mixture of both (Brazil, INCRA 2006). In PAEs, the settlements are organized around *agrovilas* (planned agricultural villages) where the families live. *Lotes* destined for the settlers’ production are located elsewhere in settlement, in some cases far from the *agrovilas* (Silveira and Wiggers 2013). In the PDS category, the division into *lotes* can be made if settlers request an individual area or if division into *lotes* is needed to avoid territorial conflicts between settlers (Guerra 2002). Despite the difference in property arrangements in both traditional (in *lotes)* and environmentally distinctive settlements (*agrovilas* and *lotes)*, the Project for Monitoring Amazonian Deforestation (PRODES) data used in the present study and the road network (Brazil, IBAMA 2016) show that deforestation in both groups is concentrated along the access roads (known as *vicinais*) opened inside the settlements. The carbon stock is stored in areas of remaining vegetation far from the roads and, when the law is followed, in the Legal Reserve and the Permanent Preservation Areas (APPs)

Traditional and environmentally distinctive settlements are similar in terms of the process of settlement implementation (land acquisition, registration and selection of settlers, and provision of infrastructure such as roads, water and electricity). INCRA is responsible for providing these items, and, in some cases, there is participation of institutions, such as the state government, organized civil society and IBAMA (Brazilian Institute of Environment and Renewable Natural Resources).

Most settlements were designed without concern for environmental impacts, biophysical conditions, and local limitations (Caviglia-Harris and Harris 2011). For example, settlements established along the Transamazon Highway in the 1970s failed to create sustainable agricultural communities because most were in areas with poor soil (only 3 % of the soil was considered fertile) or steep topography; most of the deforested land was soon converted to pasture (Mahar 1989). The Transamazon Highway settlements also failed to fulfill their stated purpose of providing a solution to poverty, overpopulation, and inequality in land distribution in Brazil’s Northeast region (Fearnside 1986a; Moran 1981; Smith 1982).

Conversion of forest to agriculture and pasture in settlements in Legal Amazonia has been especially accentuated in traditional settlements such as Federal Settlement Projects (PAs). In recent decades, with the availability of markets for timber, the deforestation process in settlements has often started with logging, followed by clearing for agriculture or pasture (Alencar et al. 2016). The main direct vectors of deforestation in Brazil’s Amazonian settlements are (i) extensive cattle ranching, (ii) illegal logging, and (iii) slash-and-burn agriculture. Direct vectors are related to the productive activities of the settlers. The indirect vectors of deforestation in settlements are related to the lack of policies to support and improve the production activities in the settlements. The main indirect vectors are (i) inadequate technical assistance (which is focused on providing credit, mainly for cattle ranching), (ii) illegal land appropriation (*grilagem*) and possession of several *lotes* by a single owner, and (iii) absence of environmental monitoring (Alencar et al. 2016).

In general, deforestation patterns in settlements, mainly in traditional categories, have an orthogonal arrangement known as the “fishbone”, where clearing spreads out from access roads perpendicular to the main highway. Deforestation begins from the access road (at the front of each property) and advances toward the back of the property, independent of property size or shape. The remaining forest in areas with the fishbone pattern is characterized by long linear forest corridors (Caviglia-Harris and Harris 2011; Oliveira Filho and Metzger 2006; Simmons et al. 2016; Tucker et al. 1984).

In 2012 the Federal Prosecutors’ Office (MPF *=* *Ministério Público Federal*) indicated INCRA as one of the main actors responsible for deforestation in Legal Amazonia. Among factors that increase deforestation in settlements are irregular proceedings for creation and installation of settlements and the environmental “regularization” of land that has been illegally cleared for pasture (Brazil, MPF 2012). Due to the MPF’s action, in 2013 INCRA announced a commitment to reduce the deforestation rate by 80 % in settlements by 2020 as compared to the deforestation rate in 2005 (Brazil, MPF 2013). This represents ~4190 km^2^, according to our estimate for 2005 deforestation (5238 km^2^).

It is well known that forest clear-cutting in settlements is significant (Brandão Jr. and Souza Jr. 2006; Brandão Jr. et al. 2012; Brazil, MPF 2013; Pacheco 2009; Schneider and Peres 2015). Thus, estimates of original and remaining carbon stock in settlements are important for evaluating their current and future contributions to carbon emissions.

In order to improve our understanding of deforestation dynamics in Amazonian settlements, we estimated clearing (through 2013 based on PRODES monitoring and through 2010 based on PMDBBS monitoring) and carbon stock (original and remaining carbon in forest and non-forest vegetation) in 2740 settlements. Carbon estimates were made based on a recent carbon map developed for Legal Amazonia by Nogueira et al. (2015). The “original” carbon stock refers to the carbon stock in vegetation before 1970, when intense degradation had not yet affected the forest. This is denominated the “pre-modern” period (Nogueira et al. 2015).

In Legal Amazonia, settlements are among the categories where deforestation pressure justifies REDD (Reducing Emissions from Deforestation and Degradation) investments. A significant carbon emission reduction could be obtained in settlement areas through this mechanism (Ezzine-de-Blas et al. 2011). Our study can contribute by reporting the carbon stocks in settlements with potential for implanting the REDD mechanism. This information is important for improving agrarian reform policies to reflect the value of maintaining carbon stocks in settlement areas.

## Methods

### Study Area

**Fig. 2:**
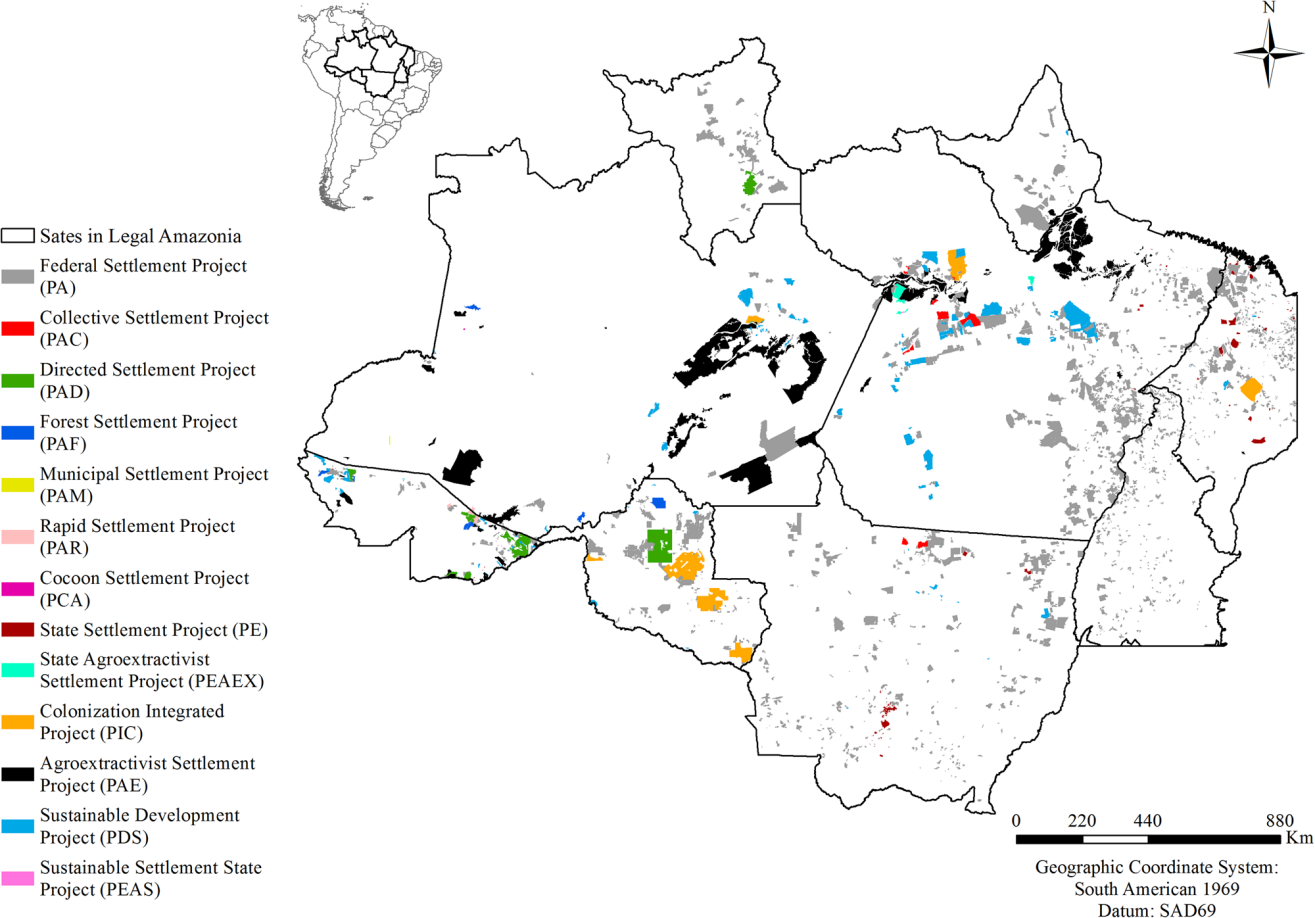
Distribution of settlements by type in Brazil’s Legal Amazonia region

The present study contemplated settlements in Legal Amazonia, an administrative region comprising Acre, Amazonas, Amapá, Mato Grosso, Pará, Roraima, Rondônia, and part of Tocantins and Maranhão states. The total area of settlements analyzed was 397,254.3 km^2^, which represents 8 % of Legal Amazonia (5,068,433 km^2^) (Brazil, INPE 2015a). We analyzed 2740 settlements distributed among 13 categories (Fig. 2; Table 1 and Supplementary Material: Table S1).

**Table 1:** Categories of settlements analyzed in comparison with INCRA data

Categories	Total settlements (Brazil, INCRA 2015b)^a^	Settlements analyzed in the current study	Percentage of settlements analyzed
Federal Settlement Project (PA = *Projeto de Assentamento Federal*)	2399	2117	88 %
Collective Settlement Project (PAC = *Projeto de Assentamento Conjunto*)	16	16	100 %
Directed Settlement Project (PAD = *Projeto de Assentamento Dirigido*)	8	8	100 %
Agro-Extractivist Settlement Project (PAE = *Projeto de Assentamento Agroextrativista*)	407	374	92 %
Forest Settlement Project (PAF = *Projeto de Assentamento Florestal*)	7	7	100 %
Municipal Settlement Project (PAM = *Projeto de Assentamento Municipal*)	1	1	100 %
Rapid Settlement Project (PAR = *Projeto de Assentamento Rápido*)	3	2	67 %
Cocoon Settlement Project (PCA = *Projeto de Assentamento Casulo*)	69	10	14 %
Sustainable Development Project (PDS = *Projeto de Assentamento de Desenvolvimento Sustentável*)	106	101	95 %
State Settlement Project (PE = *Projeto de Assentamento Estadual*)	317	89	28 %
State Agro-extractivist Settlement Project (PEAEX = *Projeto de Assentamento Estadual Agroextrativista*)	6	6	100 %
State Sustainable Settlement Project (PEAS = *Projeto Estadual de Assentamento Sustentável*)	2	1	50 %
Integrated Colonization Project (PIC = *Projeto Integrado de Colonização*)	11	8	73 %
Total	3352	2740	82 %

^a^ Based on INCRA data available at http://painel.incra.gov.br/sistemas/index.php. Accessed 7 May 2015

### Adjusting Data in INCRA’s Vector Map of Settlements

The vector map of Brazil’s settlements was obtained from the National Institute for Colonization and Agrarian Reform (Brazil, INCRA 2014) in the Geographic Coordinate System and South American 1969 Datum (SAD 69). Based on this map, we excluded settlements that are not 100 % inside the boundaries of Legal Amazonia, non-settlement categories (i.e., conservation units) present in INCRA’s settlement map and those settlements that were not listed in INCRA’s report on settlements through May 2015 (Brazil, INCRA 2015b).

Some settlements had more than one polygon shown on the map and in its associated attribute table (Brazil, INCRA 2014). Polygons were excluded in cases where the coordinates of these polygons were located in municipalities other than those indicated in INCRA’s report (Brazil, INCRA 2015b) and as indicated by the official location of the municipalities (Brazil, IBGE 2013). In cases of overlapping polygons representing the same settlement with equal area and shape, we maintained one and deleted the others. In cases where two polygons of the same settlement were near each other, we calculated the polygon areas and maintained the polygon with the area closest to the area value provide by INCRA (Brazil, INCRA 2015b). In cases where the sum of the areas of two polygons was close to the value reported by INCRA (Brazil, INCRA 2015b), we performed a merge of these polygons. This resulted in a map with only one polygon per settlement. These procedures were carried out using ArcGIS software. Problems with data and overlap in the INCRA data have also been detected by Le Tourneau and Bursztyn (2010).

### Quantification of Deforestation and Carbon Stock in Settlements in Legal Amazonia

Deforestation was estimated based on mosaics for states in Legal Amazonia in vector format from Brazil’s PRODES updated through 2013 (Brazil, INPE 2015b). PRODES is responsible for detecting clear-cutting in areas of forest vegetation in all of Legal Amazonia, including forest patches in areas where the predominant vegetation is in non-forest categories such as savanna and pioneer formations (Brazil, INPE 2000). In areas with predominance of non-forest vegetation (parts of Mato Grosso, Maranhão, and Tocantins), we used a vector map of clearing in the *cerrado* (central Brazilian savanna) biome from the Brazilian Biomes Deforestation Monitoring Project (PMDBBS) updated through 2010 (Brazil, IBAMA 2015). The *cerrado* biome is the second largest Brazilian biome (204.7 million hectares) and is characterized by vegetation classified as savanna (61 %), forest (32 %), and *campestre* (herbaceous and bushy species) (7 %) (Sano et al. 2007).

PRODES classes are forest, non-forest (i.e., savanna and other types of non-forest vegetation, such as pioneer formations), hydrography (watercourses), clouds (i.e., areas of forest covered by clouds), and annual deforestation by clear-cutting. The monitoring identifies clear-cuts >6.25 ha in area. PMDBBS has a single clearing class (which includes both cutting *cerrado* savanna vegetation and deforesting forest patches located in the *cerrado* biome) and detects clearings ≥2 ha in area. Areas of burn scars and vegetation in the process of regrowth were not considered.

We used the clip tool in ArcGIS software to cut the PRODES and PMDBBS maps according to settlement boundaries. We then used the dissolve tool in order to simplify the attribute tables of the PRODES and PMDBBS maps. We performed a union of both deforestation maps, together with the map of settlement boundaries, in order to have all information in a single vector map.

Overlap between PRODES and PMDBBS data was expected, especially in the non-forest PRODES class, because PMDBBS monitoring occurs in areas where the predominant vegetation type is non-forest. However, the forest, cloud, and deforestation classes of PRODES also had overlap with the PMDBBS clearing class. In areas of overlap between PRODES deforestation and PMDBBS clearing, we maintained the PRODES classification due to the PRODES deforestation mapping being annual. Overlap occurred in an area of 6148.3 km^2^ or 43 % of the total clearing mapped by PMDBBS. In the cases of overlap between the PMDBBS clearing class and the non-forest class of PRODES (7419.6 km^2^ or 92 % of the total clearing detected by PMDBBS considered in the present study = 8075.9 km^2^; see Table 3), forest (263.1 km^2^ or 3 %), and clouds (388.2 km^2^ or 5 %), we maintained the PMDBBS clearing class because the minimum area mapped is 2 ha and it is possible that PRODES could not identify some deforestation patches.

We used the carbon vector map for Legal Amazonia developed by Nogueira et al. (2015) to estimate the carbon stock for both the pre-modern and recent periods. The “recent” period uses PRODES data for ~2013 and PMDBBS data for ~2010. The carbon vector map refers to a map of vegetation with original biomass for each vegetation class. We performed an intersection between the carbon map and settlements with PRODES and PMDBBS data. We then calculated the area (in hectares) of each polygon and the corresponding carbon stock.

The carbon map comprised 39 classes of vegetation, without considering specific physiognomic levels. This map was developed from a biomass dataset of sampled plots scattered in vegetation classified as non-forest (*n* = 1277 plots and sub-plots with varied size), forest (*n* = 2317, 1-ha size), and contact zones (*n* = 553, 1-ha size) in Legal Amazonia. In plots of forest and contact zones, the biomass of trees was estimated mainly from wood volume per hectare that was inventoried by the RadamBrasil Project (Brazil, Projeto RadamBrasil 1973–1983; Nogueira et al. 2008a). Wood-volume estimates in each plot were converted to biomass from a dataset of mean wood density by taxon, weighted by the volumes of the different species in each plot (Nogueira et al. 2005, 2007). Estimates of the biomass of small trees, non-tree components and belowground biomass were added from several sources (Nogueira et al. 2008a). Allometric equations were used to estimate tree biomass in 10 plots (1-ha size) situated in open and dense forests in the southwestern Amazonia and in some plots or sub-plots in non-forest vegetation (Nogueira et al. 2008b, 2015). The mean carbon values per hectare were derived for the different vegetation classes from the sampled plots and were attributed to each mapped vegetation type identified in Legal Amazonia as a whole based on the classification by Brazil, IBGE (1992) and Veloso et al. (1991).

Additionally, the carbon map includes hydrography (watercourses) and urban area classes (Nogueira et al. 2015). We observed that 63 % (4038.8 km^2^) of the PRODES hydrography matched the carbon map hydrography. We decided to use the hydrography from the carbon map due to the fact that some of the classes in the PMDBBS map and in the PRODES map (e.g., forest) overlap with the hydrography of the carbon map (Supplementary Material: Table S2), which made it impossible to estimate carbon stock in these areas. In addition, the hydrography of the carbon map is slightly more detailed (6374.9 km^2^) than the PRODES hydrography (6359.7 km^2^).

Part of the PRODES hydrography that does not overlap with the carbon map hydrography was excluded from our analysis. This represents 1 % (2318.6 km^2^) of the total initial area (399,623.4 km^2^) (Table 2). In addition, 2.3 km^2^ was reclassified as deforestation in areas with PMDBBS deforestation overlap.

**Table 2:** Initial area (km^2^), excluded areas (urban areas and part of the PRODES hydrography) with percentage of excluded areas in relation to the initial area, and the final area analyzed by settlement category

Category	Initial total area	Excluded areas (%)	Updated total area
PA	205,449.5	252.5 (0.1 %)	205,197.0
PAC	4041.5	11.5 (0.3 %)	4030.0
PAD	15,603.4	12.8 (0.1 %)	15,590.6
PAE	106,881.8	1822.8 (1.7 %)	105,059.0
PAF	3181.2	2.6 (0.1 %)	3178.6
PAM	87.0	–	87.0
PAR	851.1	1.2 (0.1 %)	849.9
PCA	53.8	1.2 (2.2 %)	52.6
PDS	31,107.3	103.8 (0.3 %)	31,003.5
PE	4846.4	1.8 (0.0 %)	4844.7
PEAEX	2093.6	28.8 (1.4 %)	2064.9
PEAS	33.7	–	33.7
PIC	25,393.0	130.1 (0.5 %)	25,262.9
Total	399,623.4	2369.0 (0.6 %)	397,254.3

In the case of urban areas, we observed overlapping with PRODES classes (Supplementary Material: Table S3), but, because this was a small area (50.5 km^2^), we excluded it from our analysis. Thus, excluded areas (urban areas and part of the PRODES hydrography) represent 1 % (2369.0 km^2^) of the total initial area of the settlements (399,623.4 km^2^) and did not impact the carbon estimates. Table 2 indicates the updated area for each settlement category.

It is important to highlight that in our results the areas covered by clouds were included in the carbon stock estimates, since these areas were classified as forest by PRODES in previous years. In addition, we included in the carbon stock estimates the areas of non-forest that PMDBBS did not map as clearing. We specify the amount of carbon from forest and non-forest classes according to PRODES data in Table S4 (Supplementary Material).

### Estimation of Forest Before and Remaining Forest after Official Creation of Settlements in Legal Amazonia

Since some settlements were partially or totally cleared before the official creation date, quantifying pre-settlement deforestation is needed in order to assess deforestation rates free of the effect of prior clearing. Because the annual deforestation data in the PRODES vector maps produced by Brazil’s National Institute for Space Research (INPE) only began in 2000 (for some areas) and in 2001 (for the remaining areas), we restricted consideration to the settlements created from 2000 onwards. In order to have a sufficient period after settlement creation to allow assessment of deforestation rates in the settlements, 2008 was chosen as the cutoff for the creation year for settlements to be evaluated for pre-settlement deforestation. In 950 settlements created officially between 2000 and 2008, we calculated the original total area of forest (prior to any clearing) and the remaining forest available in the year of creation. The total area of forest was estimated based on deforestation and forest data. Remaining forest in the year of creation for each settlement was estimated by the difference between the area cleared through the year of official creation of the settlement and the original total area of forest. We decided to use the area cleared through the year of creation because there are settlements created at the beginning of the year and others at the end and because PRODES annual deforestation rates are calculated based on satellite images for the period from August of the previous year to July of the current (nominal) year (Brazil, INPE 2008). For example, “2001” deforestation represents clearing from August 2000 through July 2001.

In addition, we estimated the mean clearing per year in each settlement category in the period when deforestation rates in Legal Amazonia were high (through 2005) and in the period when the rates slowed (2006–2013). We also calculated the area of remaining forest for each year. The annual mean clearing rates were calculated from 1 year after official creation of the settlement to 2005 and from 2006 to 2013. For settlements created between 2006 and 2008, we considered the period from 1 year after creation to 2013. We compared the areas of remaining forest between the traditional settlement types (PAs) and “environmentally distinctive” settlements (PAEs and PDSs). For settlements created from 2000 to 2004 we estimated the average deforestation rate per year for each category of settlement in the period with high deforestation rates (from 1 year after creation through 2005) and the rates during the “slowdown” (2006–2013) based on PRODES data. We compared traditional and “environmentally distinctive” settlements in these two periods.

Settlements created between 2000 and 2008 with cleared areas mapped by PMDBBS inside of their boundaries were excluded from this analysis. We only used PRODES data in these cases because these deforestation data are annual.

### Comparison of Traditional and Environmentally Distinctive Settlements Inside and Outside of the arc of Deforestation

We estimated the mean annual deforestation per family considering a period of 5 years from settlement creation, considering settlements created from 2000 to 2008. We used PRODES data and settlement information on the number of families in each settlement as indicated in INCRA’s report (Brazil, INCRA 2015b). We excluded settlements without information on the number of families and settlements with 100 % of their forest cleared before the official creation of the settlement. Traditional and environmentally distinctive settlements were separated based on whether the settlement is located in municipalities inside or outside of the arc of deforestation. The arc of deforestation is the crescent-shaped area along the eastern and southern edges of the Amazon forest where deforestation activity is concentrated (e.g., World Bank 1998).

Inside the arc of deforestation, we analyzed 287 traditional settlements: PAs = 278 settlements (27,682 families); PCAs = 2 (155 families) and PEs = 7 (670 families). We analyzed 43 environmentally distinctive settlements: PAEs = 15 settlements (1949 families); PAFs = 3 (768 families) and PDSs = 25 (6033 families).

Outside of the arc of deforestation we analyzed 256 traditional settlements: PAs = 224 settlements (30,510 families); PACs = 14 (3076 families); PCAs = 3 (281 families) and PEs = 15 (1134 families). We analyzed 277 environmentally distinctive settlements: PAEs = 223 (67,422 families); PAFs = 3 (364 families) and PDSs = 51 (14,209 families).

We assumed that number of families reported by INCRA was settled in the year of creation. We only considered deforestation that occurred after official creation of settlement to estimate the mean area cleared per family in each settlement.

## Results

### Contribution of Settlements to Deforestation and Estimation of Pre-Modern (Before 1970) and “Recent” Remaining Carbon Stock in Legal Amazonia

Although the settlements analyzed occupied only 8 % of the total area of Legal Amazonia, settlements contributed 17 % (160,410 km^2^) of the total clearing in Legal Amazonia (967,003 km^2^; Nogueira et al. 2015), considering estimates of clearing in both PRODES through 2013 (152,334 km^2^) and PMDBBS through 2010 (8076 km^2^) (Table 3).

**Table 3:** Area (km^2^) of land cover based on PRODES and PMDBBS data for each settlement category analyzed

Category	Deforestation			Forest (PRODES for 2013)	Non-forest (PRODES for 2013)^a^	Clouds (PRODES for 2013)^b^	Water (Carbon map)^c^	Total
	PRODES for 2013	PMDBBS for 2010	Total					
PA	108,351.6	7282.1	115,633.7	56,488.5	17,434.7	14,954.6	685.6	205,197.0
PAC	1638.0	–	1638.0	1863.3	22.4	488.9	17.5	4030.0
PAD	10,241.0	–	10,241.0	4866.9	123.9	350.1	8.7	15,590.6
PAE	4859.9	1.3	4861.2	70,563.6	5809.3	18,716.1	5108.8	105,059.0
PAF	272.6	–	272.6	2739.6	7.9	157.7	0.8	3178.6
PAM	0.0	–	0.0	86.4	–	–	0.6	87.0
PAR	349.8	–	349.8	497.7	–	0.1	2.4	849.9
PCA	26.9	1.7	28.6	18.5	0.5	5.0	0.0	52.6
PDS	3987.2	7.0	3994.2	19,977.2	807.7	6073.8	150.5	31,003.5
PE	2162.2	728.5	2890.7	334.6	1493.2	115.9	10.2	4844.7
PEAEX	489.7	–	489.7	817.8	48.6	684.9	23.9	2064.9
PEAS	2.3	–	2.3	23.1	–	8.4	–	33.7
PIC	19,953.1	55.2	20,008.3	3046.1	1098.8	743.7	366.0	25,262.9
Total	152,334.2	8075.9	160,410.1	161,323.1	26,847.1	42,299.2	6374.9	397,254.3

^a^ Note that non-forest is a constant class in PRODES, remaining the same in all years. Here we present only the non-forest area that PMDBBS did not map as cleared

^b^ These areas were occupied by forest in previous years. Note that the area of clouds is only for the year 2013, unlike the area value for deforestation in PRODES for 2013, which represents the cumulative area up to that year

^c^ The carbon map (Nogueira et al. 2015) is derived from the vegetation map of Legal Amazonia at a scale of 1:250,000 from Brazil, IBGE (1992)

Deforestation in settlements represents 20 % (2.53 Pg C detected by PRODES through 2013 and 0.05 Pg C detected by PMDBBS through 2010; see Table 4) of the total carbon loss in Legal Amazonia (13.1 Pg C; Nogueira et al. 2015). The vegetation in areas that are currently occupied by settlements originally held 6.4 Pg of carbon (pre-modern estimate; see Table 4). This represents 9 % of the total carbon stock in Legal Amazonia (71.7 Pg C: Nogueira et al. 2015) in the pre-modern period.

**Table 4:** Estimation of carbon (Pg C) per land-cover class in the pre-modern period (before 1970) and in “recent” remaining vegetation

Category	Carbon stock losses by deforestation		Forest (PRODES for 2013)	Non-forest (PRODES for 2013)	Clouds (PRODES for 2013)	Carbon stock estimate	
	PRODES for 2013)	PMDBBS for 2010				Pre-modern period	Recent^a^
PA	1.82	0.0412	0.97	0.11	0.27	3.21	1.35
PAC	0.028	–	0.03	0.00011	0.01	0.07	0.04
PAD	0.18	–	0.08	0.00218	0.01	0.27	0.09
PAE	0.081	0.0000042	1.24	0.06	0.34	1.72	1.64
PAF	0.0046	–	0.05	0.00	0.00	0.05	0.05
PAM	0.00000065	–	0.00	–	–	0.0015	0.0015
PAR	0.0054	–	0.01	–	0.00	0.01	0.01
PCA	0.0004	0.000025	0.00	0.00	0.00	0.0009	0.00044
PDS	0.069	0.00010	0.36	0.01	0.11	0.55	0.48
PE	0.038	0.0031	0.01	0.01	0.00	0.06	0.014
PEAEX	0.0077	–	0.01	0.00	0.01	0.03	0.03
PEAS	0.000042	–	0.00	–	0.00	0.0006	0.0006
PIC	0.30	0.00083	0.05	0.01	0.01	0.38	0.073
Total	2.53	0.0452	2.82	0.20	0.76	6.36	3.78

^a^ Classes of forest, non-forest and areas covered by clouds are included

The remaining carbon stock in settlements represents 6 % (3.8 Pg C) of Legal Amazonia’s total remaining carbon stock (58.6 Pg C) (Nogueira et al. 2015). The reduction to 3.8 Pg C in comparison to the pre-modern period indicates a loss of 2.6 Pg C (41 %). According to PRODES in 2013, the remaining carbon stock (3.8 Pg C) is stored in classes of forest (2.8 Pg C or 75 %), non-forest (0.2 Pg C or 5 %) and in areas of forest covered by cloud (0.8 Pg C or 20 %) (Table 4). The distribution of PRODES and PMDBBS classes by vegetation type is presented in the Supplementary Material (Table S4). This estimate does not include carbon absorption by vegetation regrowth in deforested areas or carbon lost by human-induced or natural degradation (e.g., logging and mortality due to drought and fire) in areas that are currently forested.

**Fig. 3:**
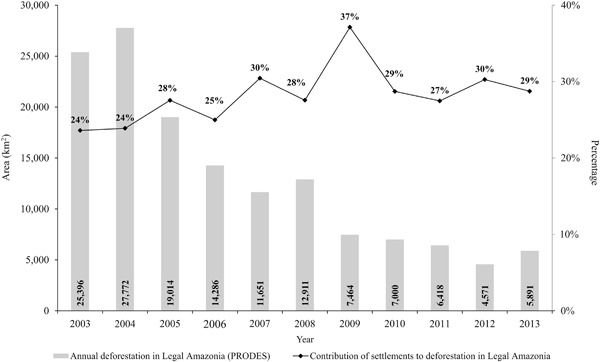
Annual deforestation in Brazil’s Legal Amazonia region (Brazil, INPE 2015c) and the respective contribution (%) of settlement deforestation to total deforestation

Considering only clear-cutting estimated by PRODES, deforestation in settlements represents 20 % (152,334 km^2^) of the total deforestation mapped by PRODES in Legal Amazonia through 2013 (758,638 km^2^) (Brazil, INPE 2015a). Figure 3 shows the percentage contribution of settlements to annual deforestation in Legal Amazonia. Between 2003 and 2013 the average annual rate of deforestation (PRODES) in settlements was 3469.7 km^2^, indicating an annual average contribution of 27 % to the annual total deforestation rate (12,943 km^2^) in Legal Amazonia.

### Deforestation by Settlement Category

In the 2740 settlements analyzed, 41 % (152,334.2 km^2^ estimated by PRODES ~2013 and 8075.9 km^2^ by PMDBBS ~2010, total = 160,410.1 km^2^) of the original vegetation (390,879.4 km^2^) was cleared. Three settlement categories were responsible for 91 % of the total deforestation: Federal Settlement Projects (PAs) with 72 % (115,633.7 km^2^), Integrated Colonization Projects (PICs) with 12 % (20,008.3 km^2^) and Directed Settlement Projects (PADs) with 6 % (10,241.0 km^2^) (Table 3).

The original vegetation was totally lost in 156 settlements (6 % of the projects). Most of these were in the PA category (142 settlements) located in Maranhão (100), Tocantins (19), Mato Grosso (11), Pará (10), and Rondônia (2). Other categories in the same situation were Agro-Extractivist Settlement Projects (PAEs) (2), “Cocoon” (*Casulo*) Settlement Projects (PCAs) (3), Sustainable Development Projects (PDSs) (2), all in Maranhão, and State Settlement Projects (PEs) (7) in Maranhão (6) and Acre (1).

In 1611 settlements (59 % of the total), deforestation by clear-cutting had already reached 50 % or more of the original vegetation cover by 2013. Out of this total, 93 % (or 1504 settlements) were in the PA category, which represents 71 % of the total PAs analyzed (= 2117 settlements). Deforestation also exceeded 50 % in 56 % (50) of the PE settlements, 16% (16) of the PDS settlements, 3 % (12) of the PAE settlements, 100 % (8) of the PIC settlements, 80 % (8) of the PCAs, 44 % (7) of the Collective Settlement Projects (PACs), and 75 % (6) of the PAD settlements. Most of these settlements are located in Pará (565 or 56 % of total settlements analyzed in this state), followed by Maranhão (384 or 75 %), Mato Grosso (284 or 72 %), and Tocantins (166 or 49 %). Acre had 59 or 45 % of the settlements analyzed in this situation and Amazonas state had only 8 (7 %).

Settlements with the largest areas of clear-cut were: the Gy-Paraná PIC in Rondônia with 4023.2 km^2^ or 85 % of its original vegetation cleared through 2013, the Ouro Preto PIC in Rondônia with 3983.4 km^2^ or 91 % of its original vegetation cleared and Tucumã PA in Pará with 3646.1 km^2^ or 90 % of its original vegetation cleared. The cleared area in the Gy-Paraná PIC represents 3 % of the total area cleared in the settlements analyzed. The Ouro Preto PIC and Tucumã PA each represents 2 % in relation to the total cleared area in settlements.

### Estimation of Pre-Modern (before 1970) and “Recent” Remaining Carbon Stock by Settlement Category

The remaining carbon (92 % or 3.5 Pg C) in settlement areas is concentrated in the PA, PAE, and PDS categories. The PAE category has the highest carbon stock (1.6 Pg C or 43 % of total the carbon stock in settlements in 2013), followed by PAs with 1.3 Pg C (36 %) and PDSs with 0.5 Pg C (13 %) (Table 4).

In 180 (7 %) of the settlements there was no clear-cutting detected by PRODES and PMDBBS monitoring. This situation occurred mainly in the PAE category (in 156 settlements, or 42 % of the settlements in this category); of these, 146 were located in Pará, 6 in Amazonas, and 4 in Amapá. The PA category had no clearing in 20 (or 1 %) of the settlements, of which 7 were in Mato Grosso, 4 in Tocantins, 4 in Pará, 3 in Maranhão, 1 in Roraima, and 1 in Amapá. The PE category had three settlements (or 3 %) without clearing: two settlements in Mato Grosso and one in Maranhão. The PDS category had one settlement (1 % of the settlements of this category), which was located in Pará.

The settlement with the largest carbon stock was the Aripuanã-Guariba PAE with 0.2 Pg C (or 5 % of the total remaining carbon stock in forest + non-forest in 2013). The remaining vegetation in the Aripuanã-Guariba PAE in 2013 covered 10,300.8 km^2^ (or 99 % of the area of the settlement). Other settlements with high carbon stocks were the Terruã PAE with 0.2 Pg C (or 4 % of the total carbon stock in 2013) and 9538.5 km^2^ (or 100 %) of its vegetation remaining and the Purus PAE with 0.13 Pg C (or 3 % of the total carbon stock in 2013) and 7500.0 km^2^ (or 98 %) of its vegetation remaining. All of these settlements are in Amazonas state. Table S5 in the Supplementary Material gives the areas of all 2740 settlements analyzed in the present study with their respective land-cover classes and carbon estimates.

### Estimation of Deforestation Before and After Official Creation of Settlements

The analysis of 950 settlements (35 % of the total settlements analyzed) created officially between 2000 and 2008 indicated that 15 % (or 146 settlements) had no clearing in forest vegetation before the official creation of the settlement. This occurred mainly in two categories: PAE (82 % or 119 settlements) in Amazonas and Pará states and in PA (12 % or 17 settlements), most of which were located in Roraima, Mato Grosso, and Maranhão. Settlements that were totally cleared before settlement creation represented 10 % (or 95 settlements). Most of these were in the PA category (90 settlements) located in Maranhão, Tocantins, and Pará states (Supplementary Material: Table S6).

Comparison of the areas of remaining forest between traditional settlements (PAs) and “environmentally distinctive” settlements (PAEs and PDSs) created between 2000 and 2008 indicated that for the PAs (584 settlements analyzed) 58 % or 21,859.5 km^2^ of original forest remained in the year of official creation of the settlements. The estimated total area of forest prior to any clearing was 37,582.2 km^2^ inside the settlement boundaries. The area of remaining forest in the year of creation was further reduced through 2013 by 29 % (6231.3 km^2^) due to deforestation activity in the settlements. In the case of the PAE and PDS categories (239 and 76 settlements analyzed, respectively), 95 % or 61,099.7 km^2^ (PAE) and 93 % or 26,133.9 km^2^ (PDS) of remaining forest was present in the year of creation. Out of this total, only 1 % (338.8 km^2^) and 5 % (1342.7 km^2^) was cleared through 2013 in PAE and PDS settlements, respectively (Supplementary Material: Table S7).

We compared deforestation by settlement type in the two periods with differing deforestation rates in Legal Amazonia: the period with high deforestation rates (through 2005) and the “slowdown” (2006–2013). The PA settlements created from 2000 to 2004 had an average of deforestation rate of 134.5 km^2^ per year in the period from 1 year after settlement creation to 2005, while from 2006 to 2013 the average of deforestation rate per year was 51.5 km^2^. In the PDS and PAE categories the average deforestation rates in the period of 1 year after creation to 2005 were 5.6 and 1.5 km^2^ per year, respectively. From 2006 to 2013 the average deforestation rates per year were 3.5 km^2^ (PDSs) and 1.7 km^2^ (PAEs). These results indicate that settlements followed the general tendency of deforestation rates in Legal Amazonia as a whole. Comparing traditional settlements (PAs) and “environmentally distinctive” settlements (PDSs and PAEs) from 1 year after creation to 2005, the average annual clearing in the traditional settlement category (134.5 km^2^) was 18.9 times higher than the rate in the “environmentally distinctive” settlements (7.12 km^2^). From 2006–2013 the traditional settlements cleared 9.9 times more (51.1 km^2^) in comparison with “environmentally distinctive” settlements (5.16 km^2^) (Supplementary Material: Table S7).

### Comparison of Traditional and Environmentally Distinctive Settlements Inside and Outside of the Arc of Deforestation

In the arc of deforestation, the families in the traditional settlement category cleared, on average, 1.7 ha (±2.2) per family per year (in the 5-year period after official creation of the settlement). Similarly, environmentally distinctive settlements located inside the arc of deforestation had an average clearing per family of 1.6 (±4.1) ha per year. In individual traditional and environmentally distinctive settlements the maximum average areas cleared per family were 29.1 ha (in the Petronilio Alves Batista PA in Pará) and 24.2 ha (in the Cernambi PDS in Rondônia).

Outside of the arc of deforestation, average area cleared per family was 1.0 (±2.1) ha per year. In environmentally distinctive settlements, each family cleared on average an area of 0.2 (±0.5) ha per year. The maximum average clearing per family in an individual traditional settlement was 29.2 ha per year (in the Alcobaça PA in Pará). In environmentally distinctive settlements the maximum average clearing per family was only 3.0 ha per year (in the Liberdade PDS in Pará).

## Discussion

### Contribution of Settlements to Deforestation and Carbon Stock Reduction in Legal Amazonia

Our finding that the settlements analyzed contributed 17 % of the total clear-cutting and 20 % of the total carbon lost in Legal Amazonia shows the importance of settlements. Despite only 8 % (397,254 km^2^) of Legal Amazonia being occupied by settlements and despite most of the cumulative deforestation (83 % or 806,593 km^2^) being outside of the settlements analyzed, the contribution of these settlements to deforestation rates and to carbon loss were both substantial and increased over time.

Most of the carbon stock loss (2.2 Pg C or 86 % of the total carbon loss in settlements) occurred in settlements situated in the arc of deforestation, where deforestation pressure is intense and the number of settlements is large (2190 settlements or 80 % of the total). In the arc of deforestation, the original carbon stock per hectare in vegetation is low in comparison with other areas, such as eastern Amazonas, northern Pará, and southern Amapá, where most of the PAE and PDS settlements are located (Fig. 1). In these areas, deforestation rates are still low but per hectare carbon stocks are greater in comparison with the arc of deforestation (Fearnside 1997; Fearnside 2010; Nogueira et al. 2015). Future deforestation in the PAE and PDS categories would therefore result in increasing carbon emission per unit area deforested. Despite these categories being “environmentally distinctive”, deforestation could progress in these areas in the future.

We observed that some settlements in areas of strong deforestation pressure (i.e., the arc of deforestation) are more vulnerable to deforestation than those situated far from these areas, regardless of whether these settlements are environmentally distinctive (PDSs, PAEs, and PAFs) or traditional (e.g., PAs, PICs, and PADs). Deforestation spreads faster in settlements in the “traditional” category as compared to “environmentally distinctive” settlements in areas of intense deforestation pressure. There is also pressure from loggers to extract timber in remaining forest areas inside settlements. Access to and transportation of timber is facilitated by the road network in the settlements (Arima et al. 2013). The result is that the landscape is more fragmented in comparison with settlements located in areas with low deforestation pressure. Deforestation rates in settlements depend on the decisions of settlers to clear-cut original forest or to reuse areas of secondary vegetation (Fearnside 1984). Furthermore, actor contributions to deforestation depend on who the dominant actors are in the area in question (Godar et al. 2012).

The annual rate of deforestation in both types of settlement (“traditional” and “environmentally distinctive”) followed the deforestation trend in Legal Amazonia as a whole indicated by PRODES. The rate had a peak in 2004 and decreased over the subsequent years, with slight increases in 2008 and 2013. Alencar et al. (2016) found the same tendency in the deforestation dynamics inside and outside of settlements in an analysis of settlements in the Amazonia biome. The “Amazonia biome”, defined by the Brazilian Institute of Geography and Statistics (IBGE) in 2004, is a 4.2 million-km^2^ area where the predominant original vegetation was Amazonian forest, although it also includes enclaves of non-forest vegetation (Brazil, IBGE 2004). The Amazonia biome is entirely contained within Legal Amazonia except for a very small area in the state of Maranhão.

Previous studies have been limited to analyzing deforestation in settlements using PRODES data (Brandão Jr. and Souza Jr. 2006; Brandão Jr. et al. 2012; Pacheco 2009). Schneider and Peres (2015) estimated deforestation in settlements using PRODES and PMDBBS data, as in our study, thereby including settlements in Mato Grosso, Maranhão, and Tocantins states located in savanna areas. These authors analyzed 1911 settlements with a total area of 267,092 km^2^ using data through 2011 from PRODES for the Amazonia biome and through 2009 from PMDBBS for the *cerrado* (16 %) and *pantanal* (Paraná River wetland) (1 %) biomes. They estimated that 55 % (146,937 km^2^) was cleared in the settlement areas they studied, representing a contribution of 13 % to the total clearing (1,092,211 km^2^) estimated in their study for Legal Amazonia.

Our study’s methodology was similar to that of Schneider and Peres (2015), although we did not use data for the *Pantanal* biome. However, due to the fact that we analyzed 829 settlements (130,162 km^2^) more than Schneider and Peres (2015) (Fig. 2; Table 1) and used PRODES data through 2013 we found different estimates for original vegetation lost (41 % or 160,410 km^2^) and for the contribution of settlements (17 %) to total deforestation in Legal Amazonia. This is because we considered the estimate of Nogueira et al. (2015) for vegetation loss (967,003 km^2^). If all settlements were included in the analysis, the impact of settlements on deforestation in Legal Amazonia would be somewhat higher. Carbon lost in settlements is also higher than estimated because our study does not consider the carbon lost by degradation in remaining forest, such as the carbon stock reduction by legal logging in areas of community forest management or by illegal logging of “legal reserve” areas inside the settlements. “Legal reserves” refer to a percentage of each property that must be maintained as forest under Brazil’s Forest Code (both Law 4.771/1965 and the current Code under Law 12.651/2012).

Deforestation in settlements is driven by settlement history, size, location, number of settlers, and the agricultural system they use (Ezzine-de-Blas et al. 2011; Pacheco 2009). Moreover, not all of the deforestation estimated in settlements can be attributed to the settlers’ activities. This is because, depending on how the settlement was obtained by INCRA, cleared areas could have already been present before the settlements were created (Pacheco 2009). Settlers often spontaneously arrive and begin clearing at a site that will only be officially established as a settlement area several years later. Schneider and Peres (2015) found that forest loss begins ~4 years before the official document (*portaria*) is issued creating a settlement. Our study analyzed 950 settlements created in the period from 2000 to 2008 and estimated that 42 % of the forest cover had been lost through the year of official creation of settlements in the PA category. In the “environmentally distinctive” settlements the percentages were 5 % for PAE and 7 % for PDS. Alencar et al. (2013) found that, in settlements created since 1997, an average of 38 % of the forest was lost before settlement creation.

Governance policies for land tenure in the states in Legal Amazonia are among the least effective in Brazil (Peres and Schneider 2012). To reduce and control deforestation in settlements, INCRA must make efforts to take effective control of agrarian reform lands, to ensure land access to landless families, recuperate degraded areas, and counter illegal deforestation in settlements. Governance policies that control illegal logging inside of settlements and support only activities with low impact must be strengthened in the settlements that have already been implemented. Furthermore, INCRA should intervene by changing its policy of considering clearing as a form of land “improvement” (*benfeitoria*) for purposes of granting land tenure rights (Fearnside 1979; Mahar 1989). Cattle ranching is the main activity when settlements are created in areas with poor soil, resulting in increasing deforestation (Fearnside 1986a, 2001; Le Tourneau and Bursztyn 2010). In the initial stage of colonization, INCRA should limit concessions to one 100-ha *lote*, not authorizing larger holdings, since cattle ranching tends to predominate in larger properties (Godar et al. 2012). Reydon et al. (2015) propose developing a land-governance system where the property can be registered, identified, and updated based on satellite images and information provided by landowners. The ideal territorial management system should be integrated at all institutional scales (federal, state, and municipal). Currently, the Rural Environmental Registry (CAR = *Cadastro Ambiental Rural*) exists to promote the identification and integration of environmental information on rural properties, including those in settlements. This information can contribute to an environmental regularization of rural properties and assist activities such as deforestation monitoring, especially in legal reserves (RLs) and permanent preservation areas (APPs) (Brazil, MMA 2016). Fearnside (2001) suggested applying high taxes to land sales and increasing the difficulty of transferring land titles in order to deal with the “industry of invasion” (i.e., settlers receiving land from INCRA and selling, only to seek a new property in another settlement). Despite the above suggestions for governance policies being “more easily said than done”, INCRA has to start to take adequate control of existing settlements before creating new settlements in intact forest.

Private Colonization Projects (PCPs = *Projetos de Colonização Particular*) were an important form of settlement in the 1970s and early 1980s. These areas are not officially classified as “settlements” and are not included in INCRA databases. Private settlements gave rise to many new municipalities; they were major sites of deforestation in the past and continue contributing to Amazonian clearing today. Most PCPs were in Mato Grosso, such as Sinop, Vera, Nova Bandeirantes, Apiacás, Alta Floresta, Paranaíta, Juruena, Colíder, Terra Nova, and Porto dos Gaúchos (Galvão 2013). In Pará, the Tucumã private colonization project initiated a major deforestation hotspot.

### Environmentally Distinctive Settlements (PAE, PDS, and PAF)

Environmentally distinctive settlements are established by both federal and state governments. We focus our discussion on federal environmentally distinctive settlements (PAE, PDS, and PAF), for which INCRA is responsible, rather than “State Agro-Extractivist Settlement Projects (PEAEX = *Projetos de Assentamento Estadual Agroextrativista*) and “State Sustainable Settlement Projects” (PEAS = *Projetos Estaduais de Assentamento Sustentável*) (Brazil, INCRA 2015b).

The carbon stock remaining in PAE, PDS, and PAF settlements (57 % of the total, or 2.2 Pg C) is relevant. This shows the importance of these three categories in terms of future carbon emissions if deforestation were to advance in these areas. This is a consequence of the large area comprised by these categories (54 % of the total = 124,853 km^2^, see Table 3) that still is covered by vegetation (forest and non-forest) and the greater per hectare carbon stock in this vegetation.

We observed that in 180 settlements (7 % of the total) no clear-cut polygons were mapped by the monitoring systems (PRODES and PMDBBS). Most of these settlements (118 settlements, or 66 % of those with no clearing) are in the PAE category, and they are identified as islands in the northern part of Pará state (e.g., PAE Ilha do Pará, PAE Ilha Maracujá I, and PAE Ilha Ituquara). In contrast, we found that in consolidated frontier areas (e.g., Maranhão) or in areas where deforestation rates are high (e.g., Pará and Mato Grosso) clear-cutting had exceeded 50 % of the settlement project area in some environmentally distinctive settlements, and some settlements had even lost all of their original vegetation.

We found that families settled in environmentally distinctive and traditional settlements cleared similar average areas per year (1.7 and 1.6 ha per family, respectively) if the settlement is located in arc of deforestation. This demonstrates the vulnerability of these settlements in areas with high deforestation pressure. The environmentally distinctive settlements are therefore not so different as compared to the traditional settlements if both are inside the arc of deforestation, indicating that both categories of settlement can have similar projected deforestation in the continued absence of mechanisms to prevent clear-cutting.

INCRA has reportedly been allowing families without an extractivist profile to be settled in PAE and PDS settlements (Guerra 2002; Silveira and Wiggers 2013). This will inevitably lead to the settlers deforesting rather than extracting non-timber forest products such as rubber (*Hevea brasiliensis*) and Brazil nuts (*Bertholetia exelsa*). Even in settlements where the families have the forest extractivist profile (e.g., rubber tappers), there is a tendency to abandon extractivist activities and shift to cattle ranching due low prices of non-timber forest products (Gomes et al. 2012). For example, in PDS São Salvador in Acre the decline in rubber price and factors such as difficulty in selling rubber and the distance from rubber-tapper houses to the areas where rubber extraction is done led the settlers (former rubber tappers) to invest in agriculture and cattle ranching. Cattle are easy to sell and access to rural credit for cattle made the settlers invest in expansion of pasture. Cattle therefore became important as a strategy for savings and as a source of income for settlers (Salisbury and Schmink 2007). The major concern regarding the PAE, PDS, and PAF categories is that, when areas of forest are transformed into settlements, the expectation will be for settlers to receive financial credit for cattle ranching, thereby threatening the forest resources (Guerra 2002). Thus, pre-existing socioeconomic factors and the geographical configuration of the frontier in which the settlements are located influence the deforestation process (Pacheco 2009).

Furthermore, there have been cases where protected areas had part of their boundaries degazetted in order to allocate the areas to “environmentally distinctive” settlements. For example, in Amazônia National Park in Pará (Law n° 12,678 of 25 July 2012: Article 3) 18,699 ha (2.5 % of the park) was transferred to INCRA for creation of “sustainable” settlements. This precedent could represent a threat to protected areas in Legal Amazonia.

### Vulnerability of Settlements to Deforestation and Carbon Loss

Settlements become vulnerable to deforestation when activities such as illegal logging take place inside their boundaries (Fearnside 2001). In Pará state there are reports of logging companies encouraging creation of settlements in forest areas just for timber extraction. In these cases, locations are chosen for the new settlements based on timber resources rather than on whether or not there is population in these areas (Greenpeace 2007). Settlements also become vulnerable when smallholders sell their properties and move to new settlements or to other locations outside of settlement areas. Thus, the area available for deforestation increases when settlers sell their lots to newcomers, who purchase multiple lots and consolidate them into a single ranch (Carrero and Fearnside 2011; Fearnside 2001, 2008).

In addition, deforestation dynamics in Amazonian settlements have been impacted by the new Brazilian Forest Code (Law no 12.651/2012), especially due to modification of the forest reforestation rule for illegally cleared portions of the Legal Reserve and the Permanent Preservation Areas (APPs = *Áreas de Preservação Permanente*) (Alencar et al. 2013). We observed that the percentage contribution of settlements to total deforestation increased from 27 % in 2011 to 30 % in 2012 (Fig. 3). Part of this increase could be a reflection of the new law. In the new Forest Code there is no obligation to reforest areas that were cleared through July of 2008 in the “legal reserve” of each property because this cleared area is recognized as “consolidated”. The requirement for recovering lost riparian vegetation in “permanent preservation areas” (APPs) depends on the property size and, in some cases, on river width; the areas required became smaller in comparison to the previous Forest Code. Thus, one could have cases in settlement areas where there are two properties of the same size (e.g.,100 ha) with different areas of forest in each property (e.g., one with 20 ha of forest and the other with 80 ha), but both are considered to be “regular”. This can occur because, if the clearing in the property with only 20 ha of remaining forest occurred prior to 2008, the clearing is considered to be legal (Alencar et al. 2013).

In Apuí municipality in southern Amazonas, for example, 109.1 km^2^ (9.6 %) of the area of APP on the edges of watercourses (30–500 m on each side) were cleared through 2012. Out of this total, 75 % (81.4 km^2^) were in settlement areas: the Juma Federal Settlement Project (PA Juma), PAE Aripuanã Guariba, and PAE São Benedito. Most of the illegal clearing occurred in PA Juma, with 74.4 km^2^ or 68 % of the total APP area having been cleared in the settlement. PAE Aripuanã Guariba was second with 6.7 km^2^ or 6 %, followed by PAE São Benedito with 0.3 km^2^ or 0.3 % (Fonseca et al. 2014). These results indicate the impact of the new rules of the Forest Code in settlement areas and the risks of expanding deforested areas in properties inside of settlements due to the new rules.

We did not differentiate deforestation resulting from settlers’ activities from that done by external actors. Future studies are needed to distinguish deforestation activities by settlers from those by external actors such as ranchers and loggers (Fearnside 2008). This is because, depending on the actor profile, the dynamics of deforestation spread in settlements can be either more intense or more moderate (Fearnside 2008; Godar et al. 2014).

The role of settlements in deforestation dynamics is significant in comparison with other land-title categories (*categorias fundiárias*) in Legal Amazonia. In 2013, settlement projects contributed 29 % (1399.9 km^2^) of the total deforestation in Legal Amazonia. The second greatest contribution to deforestation was in areas of “land lacking title information”, which contributed 23 % (1121.4 km^2^) of deforestation in 2013, followed by “private property” with 20 % (994.0 km^2^) and “non-designated public lands” with 14 % (665.2 km^2^) (IPAM et al. 2014). Thus, one of biggest challenges for agrarian reform policies in Brazil is to provide land access to settlers and, at the same time, to protect the remaining forest in settlements in Legal Amazonia (Brandão Jr. and Souza Jr. 2006; Brandão Jr. et al. 2013).

## Conclusions

Our findings indicate that settlements have an important role in deforestation dynamics and in future carbon emissions in Brazil’s Legal Amazonia region. Estimates of “pre-modern” (~1970) carbon stocks and of “recent” remaining carbon stocks in 2010 and 2013 improve our understanding of the current situation in Amazonian settlements and the impact of agrarian reform policies.

The contribution of settlements to carbon stock loss has been increasing over the years, and it is expected to continue to increase due the creation of new settlements in areas with high carbon stocks. The risk of loss is especially great given our finding that, in areas under high deforestation pressure, settlers in “environmentally distinctive” settlements have deforestation behavior similar to settlers in “traditional” settlements, implying vulnerability to increased deforestation activity.

Therefore, the agrarian reform policies concerning creation of new settlements should consider the potential carbon stock losses due to the implementation of new settlements and the activities of settlers and other external actors that contribute to deforestation in settlements.

Estimates of deforestation and of carbon stock reduction in Brazil’s Amazonian settlements allow us to assess the impact of agrarian reform policies on land-use and land-cover change. Carbon stock loss in settlement projects could be used as an indicator of the environmental feasibility of current agrarian reform policies in Legal Amazonia.

## Electronic supplementary material

Supplementary Information
