# Supplementary material for: Deforestation and Carbon Stock Loss in Brazil’s Amazonian Settlements
Source: Environ Manage. 2016 Oct 24;59(3):393–409. doi: 10.1007/s00267-016-0783-2 (PMC5306089; doi:10.1007/s00267-016-0783-2)
Supplement: Supplementary file 1 — Supplementary Information [file 267_2016_783_MOESM1_ESM.pdf]

SUPPLEMENTARY MATERIAL

Deforestation and carbon stock loss in Brazil's Amazonian settlements

Aurora Miho Yanai[1], Euler Melo Nogueira[1], Paulo Maurício de Alencastro Graça[1,2], Philip Martin Fearnside[1,2,3]

[1]Department of Environmental Dynamics, National Institute for Research in Amazonia (INPA), Av. André Araújo nº 2936, CEP 69067-375, Manaus, Amazonas, Brazil.

[2]Brazilian Research Network on Climate Change (RedeClima)

[3]Corresponding author

Table S1 Total number of settlements analyzed by state in comparison with INCRA data. ……………………………………………………………………….… p. 2

Table S2 Overlapping areas (km^2) between the carbon map hydrography and PRODES and PMDBBS classes. ………………………………………………………… p. 2

Table S3 Overlapping areas (km^2) between PRODES classes and urban areas (carbon map). ……………………………………………………………………..… p. 2

Table S4 Area (km^2) and carbon stock (Pg) by vegetation type in relation to land-cover classes. ………………………………………………………………………… p. 3

Table S5 Areas of land cover classes and the corresponding carbon for each settlement analyzed. Total deforestation represents the PRODES (~2013) + PMDBBS (~ 2010) data…………………………………………………………………………..… p. 5

Table S6 Estimates of area of forest and of clearing (km^2 and %) through the year of official creation of each settlement and in the period after creation (from one year after creation to 2005 and from 2006 or one year after creation to 2013) in 950 settlements created between 2000 and 2008 ……………………………………..……… p. 108

Table S7 Total area originally forested and area of forest remaining in the year of official creation of each settlement and in two different years (2005 and 2013) and the annual mean area cleared in two periods in Federal Settlement Projects (PA), Sustainable Development Projects (PDS) and Agro-Extractivist Settlement Projects (PAE) created from 2000 to 2008 (total = 899 settlements). ……………… p. 160

Table S1 Total number of settlements analyzed by state in comparison with INCRA data.

States in Legal Amazonia	Total of settlements (Brazil, INCRA 2015b)*	Settlements analyzed in current study	% of settlements analyzed
Acre	143	131	92%
Amapá	49	36	73%
Amazonas	110	109	99%
Maranhão	796	510	64%
Mato Grosso	541	396	73%
Pará	1090	1004	92%
Rondônia	195	169	87%
Roraima	67	48	72%
Tocantins	361	337	93%
Total	3352	2740	82%

*Based on INCRA data available online <http://painel.incra.gov.br/sistemas/index.php>. Accessed 7 May 2015.

Table S2 Overlapping areas (km^2) between the carbon map hydrography and PRODES and PMDBBS classes.

Class	Overlapping areas with carbon map hydrography	Initial area updated
Deforestation through 2013 (PRODES)	417.7 (0%)	152,802.3
Deforestation through 2010 (PMDBBS)	7.5 (0%)	8,081.1
Forest (2013)	774.5 (0%)	162,097.7
Non-forest	935.9 (3%)	27,783.0
Clouds (2013)	199.3 (0%)	42,498.5
Total	2,335.1 (1%)	393,262.6

Table S3 Overlapping areas (km^2) between PRODES classes and urban areas (carbon map).

PRODES class	Overlapping with urban areas	PRODES total area updated without urban areas
Deforestation through 1997	49.9	77,735.6
Deforestation in 2000	0.3	15,746.8
Deforestation in 2001	0.2	11,823.2
Deforestation in 2004	0.02	6,635.2
Forest (2013)	0.02	161,323.1
Non-forest	0.04	26,847.1
Total	50.5	300,111.0

Table S4 Area (km^2) and carbon stock (Pg) by vegetation type in relation to land-cover classes.

	Vegetation type (carbon map)[1]	PRODES (~2013)								PMDBBS (~2010)	
		Deforestation through 2013		Forest in 2013		Non-Forest[2]		Areas covered by clouds		Clearing through 2010[3]	
		Area	Carbon	Area	Carbon	Area	Carbon	Area	Carbon	Area	Carbon
Vegetation types classified as forest by PRODES in accord with Brazil, IBGE (1992)	Forested *campinarana* [*Campinarana* is woody oligotrophic vegetation on white-sand areas]	11.51	0.00	29.11	0.00	7.28	0.00	33.14	0.00	0.00	0.00
	Contact zones[4]	12,302.18	0.19	1,025.92	0.02	1,456.25	0.02	859.13	0.01	998.20	0.01
	Seasonal, deciduous forest	343.18	0.00	132.51	0.00	111.68	0.00	77.88	0.00	96.62	0.00
	Seasonal, semideciduous forest	12,964.62	0.20	5,430.08	0.08	1,021.43	0.02	283.75	0.00	348.07	0.01
	Open-canopy rainforest	49,877.32	0.82	30,385.36	0.47	1,634.80	0.03	2,154.20	0.04	109.69	0.00
	Dense-canopy rainforest	69,385.46	1.29	121,085.90	2.23	4,622.16	0.08	38,332.61	0.70	7.77	0.00
	Pioneer vegetation in areas with riverine influence	710.87	0.00	558.03	0.00	3,408.43	0.00	147.33	0.00	0.26	0.00
	Seasonal forested savanna	4,180.52	0.03	1,711.20	0.01	2,364.13	0.02	113.50	0.00	681.88	0.01
	Total	149,775.67 (98%)	2.53 (100%)	160,358.12 (99%)	2.81 (100%)	14,626.17 (54%)	0.17 (85%)	42,001.54 (99%)	0.76 (100%)	2,242.48 (28%)	0.03 (63%)
Other vegetation types (Non-Forest)	Arboreal *campinarana* and grassy-wood *campinarana*	30.20	0.00	57.07	0.00	51.23	0.00	63.99	0.00	0.00	0.00
	Contact zone between savanna and steppe-like savanna	0.20	0.00	0.00	0.00	3.53	0.00	0.00	0.00	0.00	0.00
	Pioneer vegetation in areas with marine influence	50.29	0.00	100.35	0.00	22.48	0.00	48.13	0.00	0.00	0.00
	Refugium areas	12.21	0.00	4.49	0.00	12.78	0.00	0.00	0.00	0.00	0.00
	Seasonal treed savanna	1,970.64	0.01	381.11	0.00	6,077.52	0.02	86.67	0.00	4,396.49	0.01
	Seasonal grassy-woody savanna	61.99	0.00	63.84	0.00	948.55	0.00	19.54	0.00	264.16	0.00
	Seasonal parkland savanna	432.95	0.00	358.11	0.00	5,104.80	0.01	79.33	0.00	1,172.78	0.00
	Total	2,558.49 (2%)	0.01	964.98 (1%)	0.00	12,220.89 (46%)	0.03 (15%)	297.65 (1%)	0.00	5,833.43 (72%)	0.02 (37%)
	Overall total	152,334.15	2.53	161,323.10	2.82	26,847.05	0.20	42,299.19	0.76	8,075.91	0.05

[1]Carbon map (Nogueira et al. 2015) is derived by vegetation map of Legal Amazonia at a scale of 1:250,000 from Brazil, IBGE (1992);

[2]Overlap between polygons of non-forest class of PRODES and IBGE forest polygons;
[3]Deforestation detected by PMDBBS through 2010 including forest and non-forest vegetation in the *cerrado* biome that PRODES classifies as non-forest and forest for which clear-cutting was not mapped in previous years;
[4]*Contact zones:* Contact between rainforest and seasonal forest; Contact between savanna steppe and seasonal forest; Contact between savanna and seasonal forest; Contact between savanna and rainforest; Contact between *campinarana* (oligotrophic vegetation on white-sand soil) and rainforest.

Table S5 Areas of land-cover classes and the corresponding carbon for each settlement analyzed. Total deforestation represents the PRODES (~ 2013) + PMDBBS (~ 2010) data.

Code	Settlement name	Deforestation				Forest (PRODES)		Non-Forest (PRODES)		Areas covered by clouds (PRODES)		Total vegetation remaining (forest+non-forest+clouds)		Carbon stock in pre-modern period	Water
		PRODES (~2013)		PMDBBS (~2010)											
		Area (ha)	Carbon loss (Mg ha^{-1})	Area (ha)	Carbon loss (Mg ha^{-1})	Area (ha)	Carbon stock (Mg ha^{-1})	Area (ha)	Carbon stock (Mg ha^{-1})	Area (ha)	Carbon stock (Mg ha^{-1})	Area (ha)	Carbon stock (Mg ha^{-1})	Carbon stock (Mg ha^{-1})	Area (ha)
**ACRE**															
AC0003004	PAR MÁRIO LOBÃO	33470.0	5251563.6	0.0	0.0	39363.1	5705046.8					39363.1	5705046.8	10956610.4	236.6
AC0003008	PAR ALELUIA	1505.9	151868.7	0.0	0.0	10409.7	1049814.3			5.8	581.8	10415.4	1050396.1	1202264.8	
AC0005000	PAD PEDRO PEIXOTO	217223.0	39488673.9	0.0	0.0	79661.6	14549041.7			114.7	21383.7	79776.3	14570425.3	54059099.2	
AC0006000	PAD BOA ESPERANÇA	41701.0	6384251.1	0.0	0.0	34394.4	4915175.3			19.6	1973.9	34414.0	4917149.2	11301400.3	19.7
AC0007000	PAD QUIXADÁ	52728.3	9351760.0	0.0	0.0	23030.5	3998864.2					23030.5	3998864.2	13350624.2	
AC0008000	PAD HUMAITÁ	44975.0	7530713.9	0.0	0.0	15912.8	2619450.4					15912.8	2619450.4	10150164.4	
AC0009000	PAD SANTA LUZIA	23970.1	4223165.0	0.0	0.0	18526.9	3294794.7			14246.3	2533121.3	32773.3	5827916.0	10051081.0	
AC0010000	PA ESPINHARA	1235.9	195828.5	0.0	0.0	378.1	54932.3					378.1	54932.3	250760.9	
AC0011000	PA FIGUEIRA	14574.1	1729179.3	0.0	0.0	10787.9	1225297.0					10787.9	1225297.0	2954476.3	
AC0012000	PA VISTA ALEGRE	421.0	76399.1	0.0	0.0	509.2	89961.0					509.2	89961.0	166360.1	
AC0013000	PAE REMANSO	8714.2	1601554.7	0.0	0.0	34921.4	6166605.3					34921.4	6166605.3	7768160.0	
AC0014000	PAE SANTA QUITÉRIA	11605.6	1521866.5	0.0	0.0	31193.7	3814467.3			22.9	2861.1	31216.6	3817328.4	5339195.0	
AC0015000	PA SÃO PEDRO	10256.7	1900808.7	0.0	0.0	15166.7	2816977.1					15166.7	2816977.1	4717785.8	
AC0016000	PAE CHICO MENDES	2655.7	494994.8	0.0	0.0	21180.6	3949599.5					21180.6	3949599.5	4444594.2	
AC0017000	PA PAVÃO	767.4	142839.8	0.0	0.0	3452.8	643659.7			6.3	1183.6	3459.2	644843.2	787683.1	
AC0018000	PAE PORTO DIAS	4363.2	770765.8	0.0	0.0	19905.3	3507063.0					19905.3	3507063.0	4277828.8	
AC0019000	PAE RIOZINHO	1800.1	292295.1	0.0	0.0	28582.2	4802500.2					28582.2	4802500.2	5094795.3	
AC0020000	PA SÃO JOÃO DO BALANCEIO	12580.6	2335082.7	0.0	0.0	5189.2	964423.7					5189.2	964423.7	3299506.3	
AC0022000	PAE PORTO RICO	3069.8	572190.5	0.0	0.0	4758.1	887211.0					4758.1	887211.0	1459401.5	
AC0023000	PA CARÃO	8911.3	1362689.5	0.0	0.0	2324.9	341083.4					2324.9	341083.4	1703772.9	

AC0024000	PA SANTO ANTONIO DO PEIXOTO	9994.2	1788199.5	0.0	0.0	5249.2	948948.7			5249.2	948948.7	2737148.3	
AC0025000	PA CUMARU	4828.4	897126.3	0.0	0.0	2721.8	507131.9			2721.8	507131.9	1404258.3	
AC0026000	PA NAZARÉ	3607.0	365407.4	0.0	0.0	4383.4	459889.1	8.6	863.3	4392.0	460752.3	826159.8	2.9
AC0027000	PA BENFICA	5028.8	926858.6	0.0	0.0	364.9	64072.0			364.9	64072.0	990930.6	
AC0028000	PA NOVO DESTINO	9613.3	1308029.5	0.0	0.0	25762.4	2776138.1			25762.4	2776138.1	4084167.6	65.0
AC0029000	PA FAVO DE MEL	7689.3	1375185.9	0.0	0.0	2175.5	395341.3			2175.5	395341.3	1770527.2	
AC0030000	PA COLIBRI	703.0	120022.8	0.0	0.0	644.0	110483.9			644.0	110483.9	230506.7	
AC0031000	PA BAIXA VERDE	4090.8	759042.3	0.0	0.0	623.1	115811.7			623.1	115811.7	874854.0	
AC0032000	PA SÃO GABRIEL	7468.4	1385858.0	0.0	0.0	2618.8	482487.7			2618.8	482487.7	1868345.7	
AC0033000	PA AMÔNIA	2551.8	426791.5	0.0	0.0	3994.6	647730.8			3994.6	647730.8	1074522.2	71.1
AC0034000	PA TRACUÁ	377.7	70168.9	0.0	0.0	4810.6	896606.9			4810.6	896606.9	966775.8	
AC0035000	PA NOVA CINTRA	1017.6	188180.3	0.0	0.0	420.3	77933.4			420.3	77933.4	266113.7	25.3
AC0036000	PA IUCATAN	840.1	148580.0	0.0	0.0	7.2	1342.3			7.2	1342.3	149922.3	1.5
AC0037000	PA RIO AZUL	447.1	77925.8	0.0	0.0	2538.6	446388.0			2538.6	446388.0	524313.8	
AC0038000	PA AMENA	618.9	94681.8	0.0	0.0	1232.2	194476.3			1232.2	194476.3	289158.0	
AC0039000	PA ENVIRA	3435.9	562471.3	0.0	0.0	2277.8	385420.9			2277.8	385420.9	947892.1	6.7
AC0040000	PA SÃO DOMINGOS	1013.5	175299.4	0.0	0.0	71.6	13069.9			71.6	13069.9	188369.3	
AC0041000	PA CAQUETÁ	11501.2	2106880.9	0.0	0.0	5982.8	1062666.9			5982.8	1062666.9	3169547.7	
AC0042000	PA TRIUNFO	9827.0	1724435.0	0.0	0.0	2460.1	437639.9			2460.1	437639.9	2162074.9	
AC0043000	PA PARANÁ DOS MOURAS	4103.8	762738.9	0.0	0.0	19683.9	3663126.0	24.0	4468.9	19707.8	3667594.9	4430333.8	
AC0045000	PAE CANARY	783.5	98617.8	0.0	0.0	8393.4	876089.1			8393.4	876089.1	974706.8	
AC0046000	PA BOA ÁGUA	3174.8	540965.7	0.0	0.0	948.4	163338.5			948.4	163338.5	704304.1	
AC0047000	PA TARAUACÁ	6411.8	1102185.1	0.0	0.0	6562.4	1141935.9			6562.4	1141935.9	2244121.0	52.9
AC0048000	PA PORTO ACRE	1342.4	151158.5	0.0	0.0	727.5	83958.9			727.5	83958.9	235117.5	
AC0049000	PA GAL. MORENO MAIA	12256.1	2132609.7	0.0	0.0	8321.3	1457427.7			8321.3	1457427.7	3590037.4	63.6
AC0050000	PA VITÓRIA	363.1	55671.5	0.0	0.0	826.0	138056.7			826.0	138056.7	193728.2	43.1
AC0051000	PA SANTA ROSA	1130.7	196739.5	0.0	0.0	3733.2	656414.6			3733.2	656414.6	853154.1	62.9
AC0052000	PA TOCANTINS	14599.4	1704139.6	0.0	0.0	9721.0	1205167.0			9721.0	1205167.0	2909306.5	

AC0053000	PA ORION	11256.0	1985816.5	0.0	0.0	5289.7	932489.9			5289.7	932489.9	2918306.4	5.5
AC0054000	PAE LIMOEIRO	434.2	70080.0	0.0	0.0	9723.5	1326654.5			9723.5	1326654.5	1396734.5	
AC0056000	PA ORIENTE	2277.4	279942.2	0.0	0.0	5651.4	660418.3			5651.4	660418.3	940360.5	
AC0057000	PA LIMEIRA	1419.9	264401.9	0.0	0.0	330.2	61519.9			330.2	61519.9	325921.8	
AC0058000	PA TAQUARI	1091.2	192329.5	0.0	0.0	8439.6	1487565.0			8439.6	1487565.0	1679894.5	
AC0059000	PA LIBERDADE	3995.6	406399.2	0.0	0.0	22362.3	2299244.2	13.3	1342.8	22375.6	2300587.0	2706986.2	94.0
AC0060000	PA ESPINHARA - II	3455.2	362840.8	0.0	0.0	2762.6	279800.9			2762.6	279800.9	642641.7	
AC0061000	PA ALCOBRÁS	5396.5	992078.5	0.0	0.0	2291.2	416329.2			2291.2	416329.2	1408407.8	
AC0062000	PA PORTO ALONSO	4382.7	805253.8	0.0	0.0	4756.3	860004.9			4756.3	860004.9	1665258.7	
AC0063000	PA TRÊS MENINAS	1445.6	267996.1	0.0	0.0	495.1	92030.9			495.1	92030.9	360027.0	
AC0064000	PA PÃO DE AÇUCAR	4475.1	832813.2	0.0	0.0	1771.0	329960.0			1771.0	329960.0	1162773.2	
AC0065000	PA TREZE DE MAIO	1839.0	334088.5	0.0	0.0	980.5	180703.2			980.5	180703.2	514791.7	14.7
AC0066000	PA PRINCEZA	507.6	90631.8	0.0	0.0	556.5	100203.2			556.5	100203.2	190835.0	
AC0068000	PDS SÃO SALVADOR	3220.1	558023.1	0.0	0.0	47652.6	8383364.5	23.9	4191.7	47676.6	8387556.2	8945579.3	
AC0070000	PA TUPÁ	3199.9	382390.6	0.0	0.0	2989.9	304597.4			2989.9	304597.4	686987.9	
AC0071000	PAE EQUADOR	1122.6	209027.5	0.0	0.0	6748.9	1258157.8			6748.9	1258157.8	1467185.3	
AC0072000	PAE CRUZEIRO DO VALE	1867.3	258667.2	0.0	0.0	75712.4	9640213.7	388.7	67489.5	76101.2	9707703.2	9966370.4	
AC0074000	PA NARCISO ASSUNÇÃO	654.8	114258.7	0.0	0.0	3863.0	680125.9	426.0	75078.9	4289.0	755204.8	869463.5	
AC0075000	PA PEDRO FIRMINO	813.5	143371.0	0.0	0.0	5535.0	975596.7	44.6	7859.6	5579.6	983456.4	1126827.4	
AC0076000	PA ZAQUEU MACHADO	3482.1	644722.5	0.0	0.0	276.3	51355.4			276.3	51355.4	696078.0	
AC0077000	PE POLO AGROF. DOM MOACIR	285.8	47480.3	0.0	0.0	34.9	5923.8			34.9	5923.8	53404.1	
AC0078000	PA PORFIRIO PONCIANO	11.9	2211.7	0.0	0.0	1747.6	325883.6			1747.6	325883.6	328095.3	
AC0079000	PE POLO AGROFLORESTAL BRASILÉIA	418.4	72079.9	0.0	0.0	120.3	21185.2			120.3	21185.2	93265.0	
AC0081000	PE POLO AGROF. WILSON PINHEIRO	251.1	28578.7	0.0	0.0	49.5	4993.4			49.5	4993.4	33572.1	
AC0082000	PE POLO AGROF. RODRIGUES ALVES	204.6	37926.5	0.0	0.0	54.3	10088.8			54.3	10088.8	48015.3	
AC0083000	PE POLO AGROF. MÂNCIO LIMA	224.9	40600.7	0.0	0.0	151.0	27579.7			151.0	27579.7	68180.4	
AC0084000	PE POLO AGROF. CRUZEIRO DO SUL	181.6	32235.9	0.0	0.0	138.1	25269.4			138.1	25269.4	57505.3	

AC0085000	PE POLO AGROF. XAPURI I	212.8	39326.2	0.0	0.0	152.3	27626.9			152.3	27626.9	66953.1	
AC0086000	PE POLO AGROF. XAPURI II	202.0	37392.0	0.0	0.0	29.3	5424.3			29.3	5424.3	42816.2	
AC0088000	PE POLO AGROFLORESTAL FEIJÓ	234.1	39322.1	0.0	0.0					0.0	0.0	39322.1	
AC0089000	PE POLO LEITEIRO DE PORTO ACRE	198.2	33839.8	0.0	0.0	7.3	1267.3			7.3	1267.3	35107.0	
AC0090000	PE POLO AGROF. EPITACIOLÂNDIA	61.0	11347.3	0.0	0.0	69.0	12852.0			69.0	12852.0	24199.3	
AC0091000	PA BERLIM RECREIO	12032.3	2042514.0	0.0	0.0	12243.9	2019842.9	174.5	22122.9	12418.5	2041965.9	4084479.8	61.6
AC0092000	PA JOAQUIM DE MATOS	2682.8	489610.0	0.0	0.0	2541.3	460470.4			2541.3	460470.4	950080.4	
AC0093000	PCA CASULO GERALDO MESQUITA	216.4	36338.1	0.0	0.0	6.6	1107.2			6.6	1107.2	37445.3	
AC0099000	PAF PROVIDÊNCIA CAPITAL	3770.2	658561.1	0.0	0.0	28536.0	5017465.5			28536.0	5017465.5	5676026.6	
AC0100000	PA ARCO-IRIS	644.0	120094.3	0.0	0.0	760.0	141721.5			760.0	141721.5	261815.8	
AC0101000	PA ITAMARATY	5277.0	647025.8	0.0	0.0	3529.1	421869.9			3529.1	421869.9	1068895.7	
AC0102000	PAF HAVAÍ	102.3	18201.1	0.0	0.0	29579.3	5256740.4			29579.3	5256740.4	5274941.5	
AC0103000	PDS JAMIL JEREISSATI	4214.7	746954.1	0.0	0.0	38887.3	6879011.1	1700.2	297814.4	40587.5	7176825.4	7923779.5	
AC0104000	PA PORTO LUIZ II	219.5	40847.6	0.0	0.0	1754.2	327002.5			1754.2	327002.5	367850.1	
AC0105000	PDS PORTO LUIZ I	2705.2	492349.0	0.0	0.0	7201.2	1283484.0			7201.2	1283484.0	1775833.0	
AC0106000	PA FORTALEZA	499.9	92815.4	0.0	0.0	500.7	92214.0			500.7	92214.0	185029.4	
AC0107000	PA PARAGUASSU	3237.9	391752.8	0.0	0.0	544.4	59874.8			544.4	59874.8	451627.6	
AC0108000	PA UIRAPURU	889.7	90452.6	0.0	0.0	1024.1	104793.7			1024.1	104793.7	195246.3	
AC0109000	PA JOÃO ADEMIR	86.8	15064.5	0.0	0.0	170.8	29637.9			170.8	29637.9	44702.5	56.5
AC0110000	PA CASTELO	428.7	48088.1	0.0	0.0	47.4	4790.6			47.4	4790.6	52878.6	
AC0114000	PDS NOVA ESPERANÇA	721.3	134506.2	0.0	0.0	2402.1	447936.6			2402.1	447936.6	582442.9	
AC0115000	PAE BARREIRO	539.4	99603.8	0.0	0.0	7622.0	1402207.1			7622.0	1402207.1	1501810.8	
AC0116000	PA PETROLINA	2605.5	483834.9	0.0	0.0	618.4	115104.7			618.4	115104.7	598939.5	
AC0117000	PAF VALENCIA	2408.3	417559.5	0.0	0.0	17941.8	3154757.9	967.7	170560.7	18909.5	3325318.5	3742878.1	
AC0118000	PA JOÃO BATISTA	1477.0	258718.0	0.0	0.0	2446.7	430292.7			2446.7	430292.7	689010.7	
AC0119000	PDS BONAL	1705.1	315754.7	0.0	0.0	9495.2	1770429.6	22.8	4258.7	9518.0	1774688.3	2090443.0	
AC0121000	PA ALBERTO	1265.3	229486.8	0.0	0.0	903.6	159482.7			903.6	159482.7	388969.4	

	SANTIAGO														
AC0122000	PDS MINAS	125.7	23332.0	0.0	0.0	2768.3	513529.7					2768.3	513529.7	536861.7	
AC0123000	PDS FRANCISCO PIMENTEL	2523.4	460739.5	0.0	0.0	51830.0	9518337.1			5.2	967.1	51835.2	9519304.2	9980043.8	
AC0124000	PAF RECANTO	314.4	55405.3	0.0	0.0	7864.0	1386025.8			829.5	146069.0	8693.4	1532094.8	1587500.1	
AC0127000	PDS IVO NEVES	1607.7	257525.6	0.0	0.0	2449.5	368590.8					2449.5	368590.8	626116.5	
AC0128000	PDS WILSON LOPES	8648.6	1610039.9	0.0	0.0	8213.0	1529205.2					8213.0	1529205.2	3139245.1	
AC0129000	PA LUZ DA VIDA	318.7	33684.2	0.0	0.0	60.7	6393.9					60.7	6393.9	40078.1	
AC0130000	PDS POLO RECANTO DO EQUADOR	1043.1	194310.6	0.0	0.0	880.6	164168.5					880.6	164168.5	358479.1	
AC0131000	PDS GERALDO FERNANDES	654.4	121828.6	0.0	0.0	1295.7	241520.6					1295.7	241520.6	363349.2	
AC0132000	PDS POLO PIRÃ-DE-RÃ	1084.7	201143.9	0.0	0.0	281.5	52302.7					281.5	52302.7	253446.6	
AC0136000	PDS LÍDIA CRAVEIRO	274.8	49365.6	0.0	0.0	530.2	95810.3					530.2	95810.3	145175.9	
AC0137000	PA URUBURETAMA	111.7	19401.3	0.0	0.0	2147.1	372609.2					2147.1	372609.2	392010.6	20.9
AC0138000	PDS SETE LAGOAS	374.8	65355.7	0.0	0.0	2029.1	357510.4					2029.1	357510.4	422866.1	
AC0139000	PA CALIFÓRNIA	2537.2	440718.4	0.0	0.0	3120.6	548258.7					3120.6	548258.7	988977.1	
AC0141000	PDS FLORESTA	3104.5	467752.0	0.0	0.0	3285.9	422606.2					3285.9	422606.2	890358.2	
AC0147000	PDS NOVA BAIXA VERDE	2828.1	524814.5	0.0	0.0	2256.4	420192.8					2256.4	420192.8	945007.2	
AC0148000	PA BARRO ALTO	3111.3	533868.7	0.0	0.0	2773.0	478958.2					2773.0	478958.2	1012826.9	
AC0149000	PAE TRIUNFO/PORONGA BA	1754.6	302829.6	0.0	0.0	22718.5	3980586.4					22718.5	3980586.4	4283416.0	357.3
AC0151000	PA MIRITIZAL	582.1	99016.1	0.0	0.0	4359.7	756214.3			69.5	12066.9	4429.3	768281.3	867297.3	
AC0152000	PA BANDEIRANTE	2950.5	297561.6	0.0	0.0	3129.2	315579.7					3129.2	315579.7	613141.2	
AC0153000	PA ANTONIO DE HOLANDA	1115.5	156178.8	0.0	0.0	1303.3	147120.2					1303.3	147120.2	303299.0	
AC0154000	PDS PORTO CARLOS	431.2	79972.2	0.0	0.0	1030.5	192010.1					1030.5	192010.1	271982.3	
AC0155000	PAE MUNDURUCUS	351.7	61200.7	0.0	0.0	7748.3	1357412.2			214.0	37432.4	7962.4	1394844.6	1456045.3	73.9
AC0156000	PA AGRO JURUA	545.8	99698.4	0.0	0.0	1955.2	361891.7					1955.2	361891.7	461590.2	1.0
AC0157000	PDS TONICO SENA	285.3	52403.0	0.0	0.0	9271.0	1718720.8					9271.0	1718720.8	1771123.7	
AC0158000	PA ABIB CURY	60.2	10152.2	0.0	0.0	3.6	562.0					3.6	562.0	10714.2	
AC0161000	PDS NATAL	561.3	97713.5	0.0	0.0	14509.0	2539246.0	0.7	119.7			14509.7	2539365.7	2637079.3	196.5

AC0162000	PDS CROA	351.5	59804.3	0.0	0.0	10127.6	1765166.5			391.9	68574.3	10519.4	1833740.8	1893545.1	36.4
AC0163000	PDS NOVA PROMISSAO	1506.1	278275.9	0.0	0.0	4389.4	807866.2					4389.4	807866.2	1086142.0	
AC0164000	PDS CAMPO ALEGRE	1388.5	257052.2	0.0	0.0	1704.0	315742.1					1704.0	315742.1	572794.3	
	Total in Acre	772447.4	131023212.8	0.0	0.0	1080437.0	175907867.4	0.7	119.7	19720.1	3482266.5	1100157.8	179390253.6	310413466.4	1569.6
**AMAPÁ**				0.0	0.0							0.0	0.0	0.0	
AP0003000	PA PIQUIAZAL	828.8	154548.7	0.0	0.0	1750.7	321750.0	70.0	12810.2	2968.7	553590.8	4789.4	888151.1	1042699.7	
AP0004000	PA PERIMETRAL	4690.3	869939.3	0.0	0.0	19246.4	3595457.0			18058.8	3373136.5	37305.2	6968593.6	7838532.9	1.2
AP0005000	PA CARNOT	6758.8	1252738.2	0.0	0.0	17072.6	3182243.7			15468.1	2883771.1	32540.8	6066014.7	7318752.9	18.3
AP0009000	PA BOM JESUS	3035.9	558761.3	0.0	0.0	15497.7	2889096.4	1264.9	34947.7	13078.3	2437011.4	29840.9	5361055.5	5919816.7	
AP0010000	PA SERRA DO NAVIO	1139.7	212548.5	0.0	0.0	10589.0	1978758.4			11964.6	2235913.8	22553.6	4214672.2	4427220.7	256.6
AP0011000	PA MATÃO DO PIAÇACA	2851.3	495079.5	0.0	0.0	16957.9	3145467.5	6377.9	195501.6	16737.1	3092341.4	40072.9	6433310.5	6928389.9	
AP0012000	PA CEDRO	4991.6	927285.9	0.0	0.0	24977.0	4652107.0	4348.8	237566.5	24335.0	4530426.3	53660.8	9420099.8	10347385.7	
AP0013000	PA MUNGUBA	2552.1	476588.9	0.0	0.0	14163.1	2646881.2			17769.6	3320898.8	31932.6	5967780.1	6444368.9	
AP0014000	PA NOVA COLINA	3043.8	567030.9	0.0	0.0	12335.0	2300222.8	212.1	34334.8	11524.4	2149064.5	24071.5	4483622.1	5050653.1	
AP0015000	PA SÃO BENEDITO DO APOREMA	915.3	62577.2	0.0	0.0	470.8	36087.4	426.9	8765.1	497.4	37093.7	1395.1	81946.2	144523.4	
AP0016000	PA PIQUIA DO AMAPÁ	1251.0	229856.3	0.0	0.0	985.0	183687.7	711.8	69419.5	1388.2	258283.6	3085.1	511390.8	741247.2	
AP0017000	PA AGRO-EXTRATIVISTA MARACÁ	3197.9	503774.1	0.0	0.0	235343.3	42397856.2	61776.3	2214217.6	269802.3	48540605.5	566922.0	93152679.3	93656453.3	1447.5
AP0018000	PA MANOEL JACINTO	1085.4	202409.3	0.0	0.0	8105.9	1511577.5	873.4	162864.1	8354.4	1557919.2	17333.7	3232360.9	3434770.2	282.3
AP0019000	PAE AGRO-EXTRATIVISTA ANAUERAPUCU	1711.6	206527.0	0.0	0.0	7874.0	1324244.3	19821.8	1616675.2	7589.2	1294045.1	35285.1	4234964.6	4441491.6	129.1
AP0020000	PA ITAUBAL	3308.2	571721.3	0.0	0.0	1210.4	208756.3	8323.0	127115.9	804.1	131851.8	10337.4	467724.0	1039445.3	
AP0021000	PA NOVA VIDA	1634.4	304183.9	0.0	0.0	2771.0	516372.2	56.7	3073.1	3926.2	732154.2	6753.9	1251599.5	1555783.4	
AP0022000	PA CRUZEIRO	950.5	70313.8	0.0	0.0	1789.8	149032.1	866.1	20696.2	2838.1	219701.2	5494.0	389429.5	459743.3	
AP0023000	PA NOVA CANAÃ	1684.5	314537.1	0.0	0.0	8765.2	1637870.0			9957.2	1860447.7	18722.4	3498317.7	3812854.7	
AP0024000	PA GOVERNADOR JANARY	769.0	105905.8	0.0	0.0	991.7	176965.5	8493.6	204438.6	1087.4	196721.9	10572.7	578126.0	684031.8	
AP0025000	PA PANCADA DO CAMAIPÍ	927.6	170396.3	0.0	0.0	11113.8	2050521.6			12925.9	2395645.9	24039.7	4446167.5	4616563.8	0.2
AP0026000	PA CUJUBIM	2183.9	396659.5	0.0	0.0	4214.6	784164.9	1506.1	81041.7	2505.8	466353.9	8226.5	1331560.5	1728220.0	

AP0028000	PA PEDRA BRANCA	3440.1	638747.4	0.0	0.0	11571.2	2161479.9			14432.6	2696179.5	26003.8	4857659.3	5496406.7	
AP0029000	PA CORRE ÁGUA	2037.7	343709.5	0.0	0.0	1664.5	293058.2	319.0	20290.1	2115.3	373129.7	4098.8	686478.1	1030187.5	
AP0030000	PA VILA VELHA DO CASSIPORÉ	1370.2	250065.5	0.0	0.0	12873.4	2384695.9	572.1	45523.9	13737.8	2544632.6	27183.3	4974852.4	5224917.9	234.5
AP0031000	PA LOURENÇO	1529.0	283686.6	0.0	0.0	9882.2	1842879.4	26.3	4904.3	15969.4	2980056.0	25877.9	4827839.6	5111526.2	
AP0035000	PA IGARAPÉ GRANDE	184.9	34475.6	0.0	0.0	741.4	138263.6	33.9	6325.9	248.1	46259.2	1023.4	190848.7	225324.2	
AP0037000	PDS - IRINEU E FELIPE	134.8	20868.8	0.0	0.0	5128.1	611962.2	646.8	19745.4	4735.2	649932.0	10510.1	1281639.7	1302508.5	41.7
AP0038000	PA FERREIRINHA	918.6	63830.2	0.0	0.0	2368.4	333758.1	16.6	387.3	2171.3	305110.5	4556.4	639255.9	703086.0	
AP0039000	PA SANTO ANTÔNIO DA PEDREIRA	0.0	0.0	0.0	0.0			745.5	7022.9			745.5	7022.9	7023.0	
AP0044000	PAE IPIXUNA MIRANDA	0.0	0.0	0.0	0.0	855.4	118815.1	929.2	31863.9	863.0	130366.1	2647.6	281045.1	281045.1	157.6
AP0045000	PAE BARREIRO	46.6	8158.6	0.0	0.0	883.9	154674.6	361.0	63168.0	817.5	143070.8	2062.4	360913.4	369072.0	0.5
AP0046000	PAE FOZ DO MAZAGAO VELHO	53.9	9429.3	0.0	0.0	7010.6	1226622.5	556.8	97258.9	6029.8	1054326.3	13597.2	2378207.6	2387636.9	86.5
AP0047000	PAE SUCURIJU	0.0	0.0	0.0	0.0	6040.9	661715.8			3048.5	333935.6	9089.4	995651.4	995651.4	328.1
AP0048000	PAE CARAPANATUBA	23.3	4078.5	0.0	0.0	873.6	152881.5	396.3	69359.8	2194.1	383974.8	3464.1	606216.1	610294.6	167.6
AP0049000	PAE NOSSA SENHORA DA CONCEIÇÃO	0.0	0.0	0.0	0.0	342.8	59987.0	40.9	7163.6	682.0	119346.6	1065.7	186497.2	186497.2	0.2
AP0050000	PAE IGARAPE NOVO	0.0	0.0	0.0	0.0	75.6	13222.4	1.3	229.2			76.9	13451.6	13451.6	7.9
	Total in Amapá	59250.6	10310432.8	0.0	0.0	476537.0	85843135.8	119775.2	5396711.3	520625.6	94027297.9	1116937.8	185267145.0	195577577.8	3159.7
**AMAZONAS**			0.0	0.0	0.0							0.0	0.0	0.0	
AM0006000	PIC BELA VISTA	62473.8	11178508.8	0.0	0.0	42213.4	7575772.1	13811.5	2429077.2	6100.9	1109604.1	62125.8	11114453.4	22292962.3	32541.3
AM0007000	PA RIO JUMA	139500.8	24132503.4	0.0	0.0	593855.8	103418847.2	9449.9	596941.8	3973.4	742173.1	607279.1	104757962.1	128890465.4	2797.2
AM0008000	PA CRAJARI	1297.9	228759.8	0.0	0.0	9179.8	1618030.5			1531.9	270010.1	10711.7	1888040.5	2116800.4	
AM0009000	PA UATUMÃ	5440.6	1009361.0	0.0	0.0	17849.8	3334048.2			226.5	42254.6	18076.3	3376302.8	4385663.8	
AM0010000	PA URUMUTUM	2018.9	350339.8	0.0	0.0	1127.4	195635.1			1545.6	268203.3	2673.0	463838.4	814178.2	
AM0011000	PA PURAQUEQUARA	554.0	102006.5	0.0	0.0	1422.4	264469.1					1422.4	264469.1	366475.5	19.3
AM0012000	PAE ANTIMARY	12475.7	1952612.1	0.0	0.0	259695.7	42101895.4	2697.9	468818.2			262393.7	42570713.6	44523325.8	745.3
AM0013000	PA VILA AMAZÔNIA	35752.4	6482367.9	0.0	0.0	17512.7	3260301.6	4522.7	272967.8	16668.5	3104846.9	38703.9	6638116.3	13120484.2	7356.4
AM0014000	PA NOVA RESIDÊNCIA	823.7	152733.1	0.0	0.0	1098.5	204659.0			141.9	26435.0	1240.4	231093.9	383827.1	133.1
AM0015000	PAE TERRUÃ	1360.4	231654.8	0.0	0.0	897495.4	142823169.7	2.9	522.9	56354.9	9950214.6	953853.2	152773907.2	153005562.0	159.4

AM0016000	PA BERURI	952.1	176857.9	0.0	0.0	21573.3	4019714.2	39.1	7300.4	347.2	64722.3	21959.7	4091736.8	4268594.8	22.8
AM0017000	PA IPORA	8510.9	1577044.3	0.0	0.0	19568.2	3645225.7			311.1	57992.1	19879.3	3703217.8	5280262.1	158.8
AM0018000	PA SAMPAIO	4558.3	818910.5	0.0	0.0	2843.4	527349.3	312.8	58291.4	2339.1	433473.4	5495.3	1019114.1	1838024.6	10.3
AM0019000	PA MATUPI	18168.4	3389406.4	0.0	0.0	16762.4	3129203.9					16762.4	3129203.9	6518610.3	
AM0020000	PA PUXURIZAL	1459.9	272247.4	0.0	0.0	3119.4	581702.8	2.9	532.3	82.8	15445.6	3205.1	597680.7	869928.1	
AM0021000	PA MONTE	52068.5	9312267.5	0.0	0.0	60700.2	10818676.4	416.2	25940.1	7.4	1330.7	61123.7	10845947.2	20158214.7	
AM0022000	PA CANOAS	3417.6	636968.0	0.0	0.0	10981.6	2039492.2			646.0	119437.4	11627.6	2158929.7	2795897.6	
AM0023000	PA TARUMÃ MIRIM	6799.6	1266690.6	0.0	0.0	36862.8	6873370.1			136.6	25479.6	36999.5	6898849.7	8165540.3	17.8
AM0024000	PA ACARI	22794.1	4192308.1	0.0	0.0	114851.6	20865367.7	2032.0	107392.6	10181.1	1894632.2	127064.7	22867392.5	27059700.6	124.3
AM0025000	PAE SANTO ANTÔNIO MOURÃO	3559.1	617169.1	0.0	0.0	12738.1	2287587.1			5368.7	948086.7	18106.7	3235673.9	3852843.0	0.4
AM0026000	PA SANTO ANTÔNIO	1233.6	229140.3	0.0	0.0	2839.1	528236.0			1.0	178.3	2840.0	528414.3	757554.6	
AM0027000	PA ÁGUA BRANCA	788.6	144489.4	0.0	0.0	486.5	90152.9					486.5	90152.9	234642.3	
AM0028000	PA SÃO FRANCISCO	3687.8	619506.6	0.0	0.0	15503.5	2663538.5					15503.5	2663538.5	3283045.0	
AM0029000	PAM IPIXUNA	3.7	645.1	0.0	0.0	8645.0	1515720.8					8645.0	1515720.8	1516366.0	55.1
AM0030000	PA UMARI	1755.7	305770.2	0.0	0.0	6748.9	1187147.4	884.3	154067.7			7633.1	1341215.2	1646985.4	
AM0031000	PA ALIANÇA	560.1	100359.9	0.0	0.0	2231.3	412176.5			0.1	24.1	2231.4	412200.6	512560.5	75.2
AM0032000	PA AQUIDABAN	1134.8	210559.8	0.0	0.0	793.4	147677.6	49.4	9138.2	123.9	23100.5	966.6	179916.4	390476.2	75.0
AM0033000	PA RIO PARDO	3431.2	639028.0	0.0	0.0	24654.5	4596468.0			98.3	18343.5	24752.8	4614811.4	5253839.5	
AM0034000	PA PAQUEQUER	814.9	151920.2	0.0	0.0	1352.9	252285.2			2609.1	486538.3	3962.0	738823.5	890743.7	
AM0035000	PA NAZARÉ	1316.8	244208.8	0.0	0.0	3266.9	609044.8			136.4	25429.8	3403.2	634474.5	878683.4	15.4
AM0036000	PA RIOZINHO	1927.8	359496.0	0.0	0.0	2474.2	461382.9			58.0	10818.6	2532.2	472201.5	831697.5	
AM0037000	PA PACIÁ	572.9	100976.1	0.0	0.0	2276.0	401161.9					2276.0	401161.9	502138.0	
AM0039000	PDS MORENA	317.3	56377.0	0.0	0.0	13700.8	2539294.6			34421.6	6361023.4	48122.4	8900318.0	8956695.1	99.7
AM0040000	PA ENGENHO	417.9	77820.9	0.0	0.0	1841.5	343309.8			2.9	545.6	1844.4	343855.4	421676.3	
AM0041000	PA CAVIANA	830.9	152673.0	0.0	0.0	4110.9	765605.6	11.6	2154.4	290.6	54188.9	4413.1	821948.9	974621.9	94.3
AM0042000	PAE MATUPIRI	319.2	56558.3	0.0	0.0	7901.9	1463284.8	1179.8	206579.1			9081.7	1669863.8	1726422.2	242.6
AM0045000	PA PIABA	712.2	132774.8	0.0	0.0	2683.7	500380.1					2683.7	500380.1	633154.9	
AM0048000	PA BÓIA	642.1	112820.9	0.0	0.0	4828.7	847457.4			835.6	147009.9	5664.3	994467.3	1107288.2	212.7

AM0049000	PAE FLORA AGRÍCOLA	1830.6	339169.1	0.0	0.0	126.4	23480.7			852.1	158170.1	978.5	181650.8	520819.9	
AM0050000	PAE BOTOS	770.0	134127.8	0.0	0.0	97580.0	17056450.2	2784.7	241029.7			100364.7	17297479.8	17431607.6	284.4
AM0051000	PAE JENIPAPOS	1439.3	107562.4	0.0	0.0	37128.5	6570298.2	1496.9	39849.3			38625.4	6610147.5	6717709.9	4.6
AM0054000	PAE CASTANHO	2238.8	404051.5	0.0	0.0	76709.7	14274122.2			6633.0	1235868.5	83342.7	15509990.7	15914042.2	445.1
AM0056000	PAE CANAÃ	755.2	140614.2	0.0	0.0	83553.9	15155026.9	3546.1	116519.1	42559.3	7710121.0	129659.4	22981667.0	23122281.3	930.6
AM0057000	PAE ABACAXIS	2519.7	449074.0	0.0	0.0	584891.5	108175617.9	1815.7	92677.8	89761.1	16647940.9	676468.3	124916236.5	125365310.5	2593.8
AM0058000	PAE CURUPIRA	18722.0	3368743.3	0.0	0.0	14479.1	2614256.9	15659.1	1926985.2	110701.0	20442387.4	140839.3	24983629.5	28352372.8	7126.3
AM0059000	PAE ABACAXIS II	4857.3	900968.8	0.0	0.0	155515.3	28548539.9	3506.1	292743.0	115360.1	21483376.6	274381.5	50324659.5	51225628.3	5532.7
AM0060000	PAE TROCANÃ	3478.3	641941.6	0.0	0.0	57787.6	10268727.2	297.3	29171.3	7672.6	1419087.1	65757.5	11716985.6	12358927.2	2077.6
AM0061000	PA ESPIGÃO DO ARARA	827.2	154251.1	0.0	0.0	3798.3	708297.7			195.3	36415.2	3993.5	744712.9	898964.0	
AM0062000	PA MANAQUIRI I - GLEBA 06	676.0	122986.0	0.0	0.0	2955.6	550874.8			365.8	68202.9	3321.3	619077.7	742063.7	
AM0063000	PA MANAQUIRI II - GLEBA 07	1073.1	190382.2	0.0	0.0	5244.0	976945.4			468.5	87336.1	5712.5	1064281.4	1254663.6	0.4
AM0064000	PDS MANDIOCA	3235.0	557051.4	0.0	0.0	1639.4	301709.6					1639.4	301709.6	858761.0	309.4
AM0065000	PAE INAJÁ	2920.1	515384.0	0.0	0.0	45457.1	8345627.6	2046.0	358516.8	1459.6	269637.3	48962.7	8973781.7	9489165.7	1333.0
AM0066000	PAE ACARÁ	6706.9	1240289.5	0.0	0.0	87679.9	16349429.6	17.0	3166.7	72173.0	13454865.7	159869.8	29807461.9	31047751.4	771.8
AM0067000	PDS RAINHA	1184.5	220149.7	0.0	0.0	17895.5	3336660.0			614.9	114656.0	18510.4	3451316.0	3671465.7	18.1
AM0068000	PAE PIRANHA	995.7	182156.1	0.0	0.0	28901.5	5051364.8	56577.4	9842558.4	13134.6	2298718.0	98613.6	17192641.2	17374797.3	37125.1
AM0069000	PAE NOVO JARDIM	3139.5	572840.6	0.0	0.0	7247.5	1350236.3			25024.1	4663788.8	32271.5	6014025.1	6586865.6	807.5
AM0070000	PDS CUIEIRAS/ANAVILHANAS	1970.9	364863.3	0.0	0.0	187951.0	35011428.8			83.1	15489.1	188034.1	35026917.9	35391781.2	1808.7
AM0073000	PAE TERRA FIRME	306.6	56496.2	0.0	0.0	7053.7	1313453.1					7053.7	1313453.1	1369949.3	
AM0074000	PAE ARIPUANÃ-GUARIBA	15405.6	2691552.5	0.0	0.0	1026435.3	187966567.7	3646.0	292415.6			1030081.3	188258983.3	190950535.8	6064.2
AM0077700	PA PANELÃO	1358.6	252198.6	0.0	0.0	2374.9	442036.1			26.7	4983.7	2401.6	447019.8	699218.4	284.4
AM0080000	PAE SÃO BENEDITO	1066.7	154335.5	0.0	0.0	192500.2	33460126.6	8506.1	381315.2			201006.3	33841441.8	33995777.3	862.4
AM0081000	PAE CABALIANA I	20657.6	3805336.7	0.0	0.0	38681.2	6951996.4	18056.0	3160108.6	2905.8	519720.1	59643.0	10631825.1	14437161.9	7736.3
AM0082000	PAE TUPANA IGAPÓ-AÇU I	438.0	79606.1	0.0	0.0	120298.9	22062505.4	695.5	8166.9	36246.0	6687318.3	157240.4	28757990.7	28837596.8	576.4
AM0083000	PAE PURUS	13200.2	2416984.6	0.0	0.0	593009.6	105899354.1	114385.6	17570467.6	42601.9	7484734.9	749997.1	130954556.5	133371541.1	93685.9

AM0085000	PDS NOVA ESPERANÇA	293.2	54480.4	0.0	0.0	9.7	1811.6			9.2	1702.7	18.9	3514.3	57994.7	19.9
AM0086000	PDS GEDEÃO	1671.4	294600.9	0.0	0.0	9655.0	1701790.5					9655.0	1701790.5	1996391.4	
AM0087000	PAE MARIPITI	3146.6	504111.0	0.0	0.0	79889.5	14575815.4	2073.5	235250.6	34534.6	6360341.7	116497.7	21171407.7	21675518.7	619.6
AM0089000	PAE URUAPIARA	1612.3	276913.3	0.0	0.0	38132.7	6943310.0	799.4	22749.3			38932.2	6966059.3	7242972.6	34.4
AM0090000	PAE SANTA FÉ	90.7	12039.1	0.0	0.0	3743.8	418836.7	873.7	14641.0			4617.5	433477.7	445516.8	3.9
AM0091000	PAE SÃO JOAQUIM	1395.8	249326.1	0.0	0.0	147032.2	26346681.2	34719.5	3793733.6			181751.7	30140414.9	30389740.9	7452.6
AM0092000	PAE FLORESTA DO IPIXUNA	236.4	42958.3	0.0	0.0	28813.4	5295889.0	434.6	76525.5			29248.0	5372414.6	5415372.8	2.0
AM0093000	PAE TUPANA IGAPÓ AÇU II	569.9	103802.3	0.0	0.0	444484.8	82564974.2	25.9	4833.1	3269.9	607622.0	447780.7	83177429.3	83281231.6	140.0
AM0094000	PAE ONÇAS	93.4	16353.4	0.0	0.0	3892.2	681128.5	4682.4	819418.6			8574.6	1500547.1	1516900.6	583.7
AM0095000	PAE CABALIANA II	3939.0	704530.0	0.0	0.0	82071.5	14805685.4	3758.2	657686.7	10080.4	1857671.0	95910.1	17321043.2	18025573.2	14258.8
AM0098000	PAE BELA VISTA II	14812.7	2413658.9	0.0	0.0	13301.5	2332752.0	7950.2	1386234.4	228.8	40055.7	21480.5	3759042.1	6172701.0	23094.6
AM0099000	PAE NOVO ORIENTE	1064.4	187614.3	0.0	0.0	17461.0	3077391.9					17461.0	3077391.9	3265006.2	
AM0100000	PAE LAGO DO ACARÁ	1178.2	193673.6	0.0	0.0	105308.5	19075216.9	960.8	124082.1			106269.3	19199299.0	19392972.5	393.9
AM0101000	PAE RIO AÇUÃ	952.5	167265.3	0.0	0.0	12241.8	2145459.0	42.9	3825.8			12284.8	2149284.8	2316550.1	
AM0103000	PDS REALIDADE	607.5	106966.7	0.0	0.0	42860.0	7552131.7	320.8	56105.7			43180.8	7608237.4	7715204.1	
AM0106000	PAE SANTA MARIA AUXILIADORA	359.8	62921.3	0.0	0.0	34384.1	6050247.6					34384.1	6050247.6	6113169.0	623.3
AM0107000	PAE ILHA DO PARANÁ DE PARINTINS	0.0	0.0	0.0	0.0			715.9	8963.4			715.9	8963.4	8963.4	1122.2
AM0108000	PAE ILHA DO ARAMAÇA	213.4	37344.5	0.0	0.0	6475.5	1133207.1			1608.8	281539.1	8084.3	1414746.2	1452090.7	291.3
AM0109000	PAE ANUMAÃ	2207.2	407218.0	0.0	0.0	10795.8	1960856.1	3375.8	596586.2	9628.2	1790248.2	23799.7	4347690.6	4754908.6	689.8
AM0110000	PAE LAGO DO SÃO RAFAEL	98.4	17176.4	0.0	0.0	65361.6	11530793.8			4769.8	843115.9	70131.4	12373909.7	12391086.1	351.1
AM0115000	PAE NOVO TEMPO ILHA MARIA ANTONIA	0.0	0.0	0.0	0.0			113.5	19860.7			113.5	19860.7	19860.7	34.3
AM0116000	PAE NOVO TEMPO ILHA MURATU	0.0	0.0	0.0	0.0			406.4	71128.3			406.4	71128.3	71128.3	134.5
AM0117000	PAE NOVO TEMPO ILHA JACURUTU	0.0	0.0	0.0	0.0			430.9	75415.4			430.9	75415.4	75415.4	45.1
AM0118000	PAE NOVO TEMPO ILHA DA PACIENCIA	0.0	0.0	0.0	0.0			3098.2	542177.1			3098.2	542177.1	542177.1	1211.2
AM0119000	PDS RIO TACANA	368.4	64115.2	0.0	0.0	806.0	142659.1			2323.9	412571.8	3129.9	555231.0	619346.2	
AM0120000	PDS SAMAUMA	680.0	122913.1	0.0	0.0	35344.9	6452989.5	3318.7	582859.6			38663.6	7035849.1	7158762.3	1575.9

AM0121000	PDS PRIMAVERA	164.1	29709.0	0.0	0.0	18004.1	3243620.6	4921.0	871293.1	324.2	59169.7	23249.3	4174083.4	4203792.5	168.7
AM0123000	PCA SÃO PAULO DE OLIVENÇA	812.7	150667.9	0.0	0.0	1641.9	305868.0	52.5	9686.5	382.0	71224.4	2076.4	386778.9	537446.8	0.6
AM0124000	PAE ILHA DO BAIXIO	0.0	0.0	0.0	0.0			513.6	89885.5			513.6	89885.5	89885.5	101.7
AM0125000	PDS COSTA DO CALDEIRÃO	328.1	59696.3	0.0	0.0	1617.7	283970.9	1250.6	223959.8	68.0	11905.2	2936.3	519835.8	579532.1	255.7
AM0126000	PDS LAGO DO TUCUNARÉ	903.6	168505.3	0.0	0.0	2468.0	460223.4			231.6	43190.8	2699.6	503414.2	671919.5	96.7
AM0131000	PAF RIO IÇÁ	486.6	85812.7	0.0	0.0	33028.9	6097480.2			13858.4	2563960.8	46887.3	8661441.0	8747253.7	81.1
AM0132000	PAE VILA ALTEROSA DO JUI	1878.4	346805.8	0.0	0.0	27218.8	4851191.3	0.2	36.6	21643.7	3878075.1	48862.7	8729303.0	9076108.8	3309.5
AM0133000	PAE GUARANÍ	1972.5	365890.1	0.0	0.0	2277.6	424586.9	305.6	3825.9	5553.3	1035553.1	8136.5	1463965.8	1829855.9	8.8
AM0134000	PDS COSTA DO IRANDUBA	177.8	31127.9	0.0	0.0	1485.4	259956.0	920.3	161053.8			2405.7	421009.9	452137.8	699.7
AM0135000	PDS NOVO REMANSO	13808.4	2558236.6	0.0	0.0	8457.6	1575637.7			136.3	25403.8	8593.9	1601041.5	4159278.1	374.1
AM0136000	PDS AMATARÍ	1067.2	198168.5	0.0	0.0	2625.1	489312.4			161.6	30127.8	2786.7	519440.2	717608.7	
AM0137000	PDS COSTA DA CONCEIÇÃO	3380.6	515300.4	0.0	0.0	5868.3	986221.5	7683.2	1136678.8	2520.8	453251.4	16072.3	2576151.8	3091452.1	1153.0
AM0145000	PDS ITAUBAO	2799.6	484217.9	0.0	0.0	5698.9	979360.4	5.4	901.0	659.3	111290.6	6363.6	1091552.1	1575770.0	420.6
AM0146000	PDS LAGO DO MIRA	1586.8	294167.5	0.0	0.0	4831.7	899579.6			234.4	43630.1	5066.1	943209.7	1237377.2	383.9
AM0147000	PDS BATATA	161.4	30079.0	0.0	0.0	2348.8	437978.3					2348.8	437978.3	468057.3	145.5
AM0148000	PAE BAETAS	3050.9	552820.2	0.0	0.0	34194.6	6035078.9	853.1	102257.8			35047.7	6137336.7	6690157.0	296.3
AM0149000	PAE FORTALEZA	898.1	96928.2	0.0	0.0	25268.9	4329201.3	412.0	33235.1			25680.9	4362436.4	4459364.7	73.6
AM0150000	PAF CURUQUETE	1308.1	210079.5	0.0	0.0	38691.4	6368800.5	793.0	19858.8	112.8	19070.1	39597.2	6407729.4	6617808.9	
AM0151000	PDS AXINIM	287.8	53224.5	0.0	0.0	6717.5	1250406.1	298.5	53070.4	3760.4	701238.5	10776.4	2004715.0	2057939.4	
	Total in Amazonas	586891.4	104110378.8	0.0	0.0	7129197.2	1265742077.2	354558.9	50493235.9	829480.1	152246319.8	8313236.2	1468481632.8	1572592011.7	275586.6
**MARANHÃO**			0.0	0.0	0.0							0.0	0.0	0.0	
MA0006000	PIC BARRA DO CORDA	301270.2	45142816.7	5524.7	828682.9	29626.7	4463342.1			6599.6	996716.5	36226.3	5460058.7	51431558.3	
MA0008000	PA OLHO D AGUA DOS GRILOS	811.9	120104.4	1289.4	189543.0					783.9	115142.2	783.9	115142.2	424789.6	
MA0009000	PA SÃO JOSE DA MATA	1887.8	318672.2	0.0	0.0			0.6	104.4			0.6	104.4	318776.6	
MA0010000	PA ENTRONCAMENTO	4391.3	695781.5	118.6	18248.7	1487.2	220856.1	109.3	17766.5	2.6	436.7	1599.1	239059.2	953089.5	
MA0011000	PA CLEMENTE/CAJAZEIRAS	8742.4	1309705.3	483.8	72475.2	2180.5	326663.2			121.6	18224.1	2302.2	344887.3	1727067.8	
MA0012000	PA MIRA FLORES	6541.6	1210345.7	0.0	0.0	1092.6	202909.7			1539.0	284579.8	2631.6	487489.5	1697835.1	

MA0013000	PA MONTE CRISTO/MARMORANA	6683.3	903734.6	3101.3	456173.7	0.0	2.4			1728.3	253886.3	1728.3	253888.7	1613797.0	7.9
MA0014000	PA SÃO JOÃO DAS NEVES	1520.2	224254.1	2620.0	384804.6					8941.0	1313077.2	8941.0	1313077.2	1922135.9	
MA0015000	PA SANTA TEREZA	1710.8	256293.6	1429.0	214083.5	2456.1	367951.6	1255.1	188031.0			3711.3	555982.7	1026359.8	
MA0016000	PA CAMACAOCA	12748.0	1837769.2	0.0	0.0	417.9	42851.7	3451.7	107395.1	82.1	12270.5	3951.7	162517.3	2000286.5	3886.2
MA0017000	PA JITIRANA	2536.4	251326.7	352.3	46599.2					2311.5	291343.6	2311.5	291343.6	589269.5	
MA0018000	PA SANTA CRUZ	1273.0	202323.7	1285.8	195875.7					1486.3	218608.9	1486.3	218608.9	616808.3	
MA0019000	PA MANGUARY	5725.5	865882.5	0.0	0.0							0.0	0.0	865882.5	43.6
MA0020000	PA KARINÃ	21047.1	3550757.2	0.0	0.0	193.6	32915.1			28.5	4883.5	222.1	37798.6	3588555.8	
MA0022000	PA BOM PASTOR	48602.5	8995211.8	0.0	0.0	4870.8	897770.2			4306.5	791845.4	9177.3	1689615.7	10684827.5	
MA0023000	PA CUMBIQUE	403.0	66510.6	0.0	0.0	285.1	46890.7					285.1	46890.7	113401.4	4.0
MA0026000	PA BREJO/SÍTIO DO MEIO	165.0	19949.9	672.9	52746.7	65.5	8882.3	1545.8	121094.0			1611.3	129976.3	202673.0	
MA0027000	PA RIACHUELO	628.6	97592.2	522.7	77594.4					1204.6	177156.4	1204.6	177156.4	352343.0	
MA0028000	PA RIO DOCE	24046.3	4045620.2	0.0	0.0	1238.4	208293.3	1331.4	240359.7	6762.3	1138231.1	9332.1	1586884.0	5632504.3	392.0
MA0029000	PA CERES	3142.0	514203.2	0.0	0.0	326.1	60759.0			445.2	82965.2	771.3	143724.2	657927.4	
MA0030000	PA EXTRATIVISTA LEITE	3022.1	419916.6	1140.8	160012.7	3410.2	477345.7			1646.7	231652.8	5056.9	708998.4	1288927.7	
MA0031000	PA BURITI/RIO BONITO	28577.4	5288555.6	0.0	0.0	2993.8	554040.2			261.1	48311.1	3254.8	602351.4	5890906.9	
MA0032000	PA CITUSA/VIAMÃO	19246.5	3566772.1	0.0	0.0	4413.2	823059.1	3204.6	594271.4	2737.5	511002.5	10355.3	1928333.0	5495105.1	
MA0033000	PA ALDEIA	513.9	85032.5	0.0	0.0							0.0	0.0	85032.5	0.2
MA0034000	PA TABOLEIRÃO	2330.5	212115.1	300.5	24959.2	1097.0	87030.7			4.2	321.8	1101.1	87352.4	324426.7	
MA0035000	PA REDONDO/ARACANGA	739.9	106693.0	0.0	0.0	45.9	6361.7	1298.0	31048.2			1343.9	37409.9	144103.0	685.6
MA0036000	PA PEDRA PRETA/MORRO DOS GARROTES	22020.9	3904804.1	1.0	190.5	4440.0	829674.8			2848.7	532351.1	7288.7	1362025.9	5267020.6	
MA0037000	PA SANTA INACIA	19918.5	3696812.8	0.0	0.0	625.5	116370.9			1127.7	209773.4	1753.3	326144.3	4022957.1	
MA0038000	PA TRÊS SETUBAL	1285.3	188824.0	2301.1	337843.3					4067.1	597189.5	4067.1	597189.5	1123856.8	
MA0039000	PA UNIÃO, PORTUGAL E SANTO ANTONIO	8478.7	1507229.4	0.0	0.0	319.8	59189.4			64.0	11834.7	383.8	71024.1	1578253.5	
MA0040000	PA SANTO ANTONIO DOS VELOSOS	712.6	104828.0	1315.0	193433.9					3071.6	451177.6	3071.6	451177.6	749439.5	

MA0042000	PA MARACAÇUMÉ	15967.1	2757846.2	0.0	0.0	80.5	14897.4			39.7	7353.1	120.2	22250.5	2780096.7	
MA0043000	PA BENTIVI	537.2	6725.8	0.0	0.0	0.9	11.4	863.8	10814.5			864.7	10826.0	17551.8	
MA0045000	PA BITIUA	12873.2	2007243.0	0.0	0.0	644.7	77774.3	11.8	1980.0	1073.3	129521.4	1729.9	209275.7	2216518.7	173.7
MA0046000	PA PAU SANTO	990.7	166323.1	0.0	0.0							0.0	0.0	166323.1	
MA0047000	PA TAUÁ/SANTA TEREZINHA	183.2	30032.0	0.0	0.0	990.8	165893.2			247.5	41543.8	1238.2	207436.9	237468.9	
MA0048000	PA SERRARIA	708.3	98099.5	0.0	0.0							0.0	0.0	98099.5	60.0
MA0049000	PA PARUÁ	5779.9	912147.3	0.0	0.0	286.1	46699.8	2023.6	170498.7	123.2	14503.3	2433.0	231701.8	1143849.2	
MA0051000	PA PALMEIRAL VIETNÃ	2410.6	399862.6	97.1	16300.3	0.0	1.2	10.5	1760.5	461.8	77532.7	472.3	79294.4	495457.2	
MA0052000	PA OURO	3532.9	593136.4	0.0	0.0	736.4	123626.4	0.1	12.6	156.2	26231.7	892.7	149870.6	743007.0	
MA0053000	PA GAMELEIRA/MATıES	2216.8	332091.8	289.1	43302.8	143.5	21492.0	19.2	2873.3			162.6	24365.3	399759.9	
MA0054000	PA BOCAINA	2284.4	335376.8	358.0	52550.9					135.9	19946.3	135.9	19946.3	407874.0	
MA0055000	PA ENGENHO CENTRAL/CLARICE	4142.9	766683.5	0.0	0.0							0.0	0.0	766683.5	
MA0057000	PA SANTA MARIA DOS FERNANDES	996.6	149478.0	1549.1	228723.5					2145.3	314945.2	2145.3	314945.2	693146.8	
MA0058000	PA CIPÓ CANAÃ	1072.9	160738.4	0.0	0.0							0.0	0.0	160738.4	
MA0059000	PA DIAMANTE NEGRO/JUTAHY	4297.1	667110.3	0.0	0.0	853.1	142198.4	1333.9	95101.5	56.1	9120.2	2243.1	246420.1	913530.5	2387.8
MA0060000	PA FAZENDA PLANALTO PEDESA	16397.9	3037751.8	0.0	0.0	253.7	47115.9			419.0	77894.5	672.7	125010.3	3162762.1	
MA0061000	PA COCEIRA/NOVA ALEGRIA	1597.4	192932.0	1257.6	156975.0	232.9	21464.3	6960.1	405116.4			7192.9	426580.7	776487.7	
MA0064000	PA VILA ESPERANÇA	1330.8	223419.9	0.0	0.0	34.9	5867.7					34.9	5867.7	229287.6	287.7
MA0065000	PA CACIQUE/TUCUMÃ	28842.9	5286277.8	0.0	0.0	942.7	172832.0			71.8	13295.2	1014.5	186127.2	5472405.0	
MA0066000	PA ITACIRA	4712.4	706514.5	31.2	4676.7	3.4	512.3			1.2	176.5	4.6	688.9	711880.0	
MA0068000	PA SÃO BARTOLOMEU/LUZILÂNDIA	6638.7	1061178.7	0.0	0.0			1944.4	52414.9			1944.4	52414.9	1113593.6	
MA0069000	PA TRIÂNGULO DE PRATA	9081.4	1681422.7	0.0	0.0	591.5	109822.1			3.3	614.2	594.8	110436.3	1791859.1	
MA0070000	PA SANTO ANTONIO SEFANS CAREMA	1091.8	180921.5	0.5	69.2	4531.0	748388.4	1.4	234.5	1286.2	213009.8	5818.6	961632.6	1142623.2	5.5
MA0071000	PA SUMAUMA / JACAMIM	6659.1	797879.9	0.0	0.0			187.7	2353.7			187.7	2353.7	800233.6	404.0
MA0072000	PA GALVÃO/CANTANHÊDE	1263.3	153005.4	906.3	109187.1					3070.2	417293.3	3070.2	417293.3	679485.8	15.8
MA0073000	PA TIMBAÚBA	725.0	88242.4	56.4	8110.4					467.9	62427.2	467.9	62427.2	158780.0	

MA0074000	PA BAIXÃO	428.5	62912.3	431.0	63278.6					2515.0	369222.6	2515.0	369222.6	495413.5	
MA0075000	PA BARRIGUDA/BAGACEIRA	1709.4	97959.9	360.3	47189.5					2500.3	278515.7	2500.3	278515.7	423665.2	
MA0076000	PA BITUBA/CHAPADA	2861.6	468490.9	0.0	0.0	179.5	20785.2			2.1	225.6	181.5	21010.8	489501.7	17.1
MA0083000	PA SANTA ROSA	2994.6	406896.4	0.0	0.0	33.3	5584.5	139.3	5053.7	7.1	1185.0	179.6	11823.3	418719.7	
MA0084000	PA LAGO AZUL	12843.0	2303648.4	0.0	0.0	161.0	29790.0			11.0	2033.0	172.0	31823.0	2335471.5	
MA0085000	PA CAJUEIRO	15919.5	2946460.9	0.0	0.0	23.1	4283.8			13.2	2446.1	36.4	6729.8	2953190.7	
MA0086000	PA JAPEL	8383.9	1346821.9	1275.4	200065.2	715.8	108827.2			1848.7	267529.5	2564.5	376356.7	1923243.8	5.4
MA0087000	PA TERRA BELA	10716.8	1943802.8	0.0	0.0	70.4	13029.9					70.4	13029.9	1956832.7	5.9
MA0088000	PA SANTA HELENA	62535.7	11535381.1	0.0	0.0	2741.6	508399.9			5061.1	936922.2	7802.8	1445322.0	12980703.1	
MA0089000	PA PURÃO DOS PIRRÓS	4222.8	708972.3	0.0	0.0							0.0	0.0	708972.3	
MA0090000	PA CASTELO	3001.8	508695.8	0.0	0.0							0.0	0.0	508695.8	
MA0091000	PA FLORESTAL/MARACASSUMÉ	35191.7	6496053.5	0.0	0.0	5523.2	1005662.3			6872.7	1253471.7	12395.8	2259134.0	8755187.6	
MA0092000	PA SANTO ANTONIO DO RIO CAXIAS	5395.5	771792.0	0.0	0.0							0.0	0.0	771792.0	
MA0093000	PA BABILÔNIA	2055.1	380313.8	0.0	0.0	3.6	666.4					3.6	666.4	380980.2	
MA0094000	PA ALTO ALEGRE	2879.2	445792.2	1112.5	163556.0					2192.6	321917.1	2192.6	321917.1	931265.3	21.0
MA0095000	PA INDIANA/SANTA MARIA	5090.6	942075.4	0.0	0.0	106.8	19766.8			6.8	1254.9	113.6	21021.7	963097.2	
MA0109000	PA LAGE COMPRIDA	1445.0	18124.1	0.0	0.0			4973.9	62273.5			4973.9	62273.5	80397.7	
MA0110000	PA PADRE CÍCERO/JASMINA	3703.0	660771.2	0.0	0.0							0.0	0.0	660771.2	
MA0111000	PA TRÊS LAGOAS	2687.7	498352.8	0.0	0.0	192.6	35873.2			365.1	67984.0	557.7	103857.3	602210.1	
MA0112000	PA CAAC	884.8	68338.2	44.0	3400.5	2.5	189.6					2.5	189.6	71928.2	
MA0113000	PA ALVORADA I	6967.8	1098975.2	0.0	0.0	209.2	21818.0			67.7	3514.1	276.9	25332.1	1124307.3	
MA0114000	PA ALVORADA II	8149.9	1511536.3	0.0	0.0	547.0	101933.3					547.0	101933.3	1613469.6	
MA0124000	PA SANTA MARIA DO CAPISTRANO	909.6	152712.5	0.0	0.0							0.0	0.0	152712.5	
MA0125000	PA CONCEIÇÃO/ARVOREDO	1164.9	38430.6	55.1	1817.0					177.4	5851.3	177.4	5851.3	46098.9	
MA0126000	PA PRAZERES	64.8	9694.5	191.7	28275.9					306.1	45961.5	306.1	45961.5	83931.9	
MA0127000	PA MACACOS	481.9	15897.0	2.6	85.6					30.5	1005.2	30.5	1005.2	16987.8	

MA0128000	PA BATUTA	1708.9	316251.6	0.0	0.0	0.7	133.2					0.7	133.2	316384.8	
MA0129000	PA VERONA	4459.8	814298.8	0.0	0.0	0.3	3.2					0.3	3.2	814302.1	0.0
MA0131000	PA ALMEIDA MATO GROSSO	317.9	44724.0	164.6	23158.2	926.2	130292.0			107.9	15178.8	1034.1	145470.8	213353.0	
MA0136000	PA QUADRA RENASCER II/COLONE	152.0	28120.6	0.0	0.0							0.0	0.0	28120.6	
MA0139000	PA VITAL BRASIL	1197.9	201118.9	0.0	0.0							0.0	0.0	201118.9	
MA0140000	PA ITAIGUARA	2175.5	406237.3	0.0	0.0	115.9	21644.9					115.9	21644.9	427882.1	
MA0141000	PA BONANZA	401.1	67345.6	0.0	0.0							0.0	0.0	67345.6	
MA0143000	PA TOCANTINS	415.4	69743.9	0.0	0.0							0.0	0.0	69743.9	
MA0144000	PA BOM VIVER	1488.8	249957.6	0.0	0.0							0.0	0.0	249957.6	
MA0145000	PA SANTA MARIA	405.3	68174.8	0.0	0.0	16.6	2790.3	955.4	169397.7	1523.7	256322.1	2495.7	428510.1	496684.9	42.4
MA0146000	PA GUARIDA	2511.1	464709.6	0.0	0.0	74.7	13818.0			18.8	3478.3	93.5	17296.3	482005.9	
MA0147000	PA GAMELEIRA	5063.8	766828.6	0.0	0.0	471.4	77449.8	1418.8	36622.2			1890.3	114072.0	880900.6	
MA0149000	PA CAXUXA	1459.9	134636.8	309.6	43870.1					1588.8	206948.9	1588.8	206948.9	385455.7	
MA0151000	PA CIGRA	21442.7	3138565.8	424.6	68394.3	1930.2	311188.0			297.9	51049.9	2228.1	362238.0	3569198.0	
MA0153000	PA OLHO D ÁGUA DO TURI/COLONE	1020.2	169633.1	0.0	0.0			154.0	14322.6			154.0	14322.6	183955.6	
MA0154000	PA PAU ROXO/VIGÁRIO	1006.3	87224.8	0.0	0.0							0.0	0.0	87224.8	264.0
MA0156000	PA SANTO ANTONIO	69.1	10324.4	3035.0	270653.9	144.3	25689.0	2332.1	267111.0			2476.5	292800.0	573778.3	
MA0158000	PA CONCEIÇÃO	1716.9	63962.8	84.9	3593.5					762.4	25896.1	762.4	25896.1	93452.4	
MA0159000	PE VIDÉU	206.6	33735.6	0.0	0.0	498.3	83181.9			531.0	89022.6	1029.4	172204.4	205940.0	
MA0161000	PE SÃO SIMÃO	827.2	139080.6	0.0	0.0	1499.5	251946.6			127.6	21423.9	1627.1	273370.5	412451.1	0.0
MA0162000	PA MATO GROSSO	146.1	24427.8	0.0	0.0			1293.7	133262.6			1293.7	133262.6	157690.4	
MA0163000	PA SIT	13435.4	2488395.1	0.0	0.0	105.5	19537.6			80.0	14864.4	185.5	34402.1	2522797.2	
MA0166000	PA NOVO MUNDO/CONQUISTA	1854.6	346602.9	0.0	0.0	872.8	163113.3			539.8	100878.3	1412.6	263991.7	610594.6	
MA0170000	PA PAVIO/VARJÃO	0.0	0.0	429.0	14151.8	0.8	125.5	2325.8	130791.7			2326.7	130917.2	145069.0	
MA0171000	PA PEDRA DE PAU	0.0	0.0	445.6	28091.5			2567.7	160196.8			2567.7	160196.8	188288.3	
MA0172000	PA JOÃO PALMEIRA/CASTÁLIA	5797.3	1036791.8	0.0	0.0	64.5	9668.1			28.5	4272.9	93.1	13941.0	1050732.8	

MA0173000	PA AÇAÍ	20133.0	3760251.6	0.0	0.0	2373.8	443599.2			914.1	170836.7	3287.9	614435.9	4374687.5	
MA0174000	PA BARRA LIMPA	1035.4	191618.3	0.0	0.0							0.0	0.0	191618.3	
MA0178000	PA SÃO PAULO	1207.6	177426.9	2873.6	421868.2					2480.6	364183.1	2480.6	364183.1	963478.1	
MA0179000	PA FAISA	11373.8	2104835.4	0.0	0.0	35.7	6608.9			158.2	29281.8	193.9	35890.6	2140726.1	
MA0180000	PA BOQUEIRÃO	428.9	62964.8	738.7	108452.9					2112.5	310132.9	2112.5	310132.9	481550.6	
MA0181000	PA BAIXÃO DO CEDRO	0.0	0.0	516.8	77420.9			1710.3	256227.0			1710.3	256227.0	333647.9	
MA0182000	PA APAGA FOGO	131.0	21994.8	0.0	0.0	282.1	46341.1	1340.7	172808.8			1622.8	219149.9	241144.7	
MA0183000	PA QUADRA SÃO JOÃO	861.0	144559.8	0.0	0.0	4.1	694.4			0.4	64.9	4.5	759.3	145319.0	
MA0184000	PA CACHIMBEIRO	7503.2	1134132.8	389.6	59247.4	10054.5	1512702.4	2227.3	63648.5			12281.7	1576350.9	2769731.1	
MA0185000	PE TAIM	77.0	11898.8	0.0	0.0	10.2	1366.2					10.2	1366.2	13265.0	
MA0186000	PA PORTUGAL	1606.0	265476.2	0.0	0.0	241.7	28287.6			44.2	5610.4	285.9	33898.1	299374.2	36.2
MA0187000	PA PLANALTO II	418.5	72056.9	0.0	0.0	9.7	1791.2					9.7	1791.2	73848.1	
MA0188000	PA INTERNACIONAL	879.5	162759.2	0.0	0.0	76.0	14069.9					76.0	14069.9	176829.1	
MA0189000	PA OLHO D ÁGUA DOS CARNEIROS	250.5	42054.6	0.0	0.0			0.6	94.7			0.6	94.7	42149.3	1.0
MA0190000	PA AREIAS	216.5	36340.4	0.0	0.0	0.3	54.4	628.6	45679.2			628.9	45733.6	82074.0	50.6
MA0198000	PA PLANALTO I	1194.4	221040.0	0.0	0.0	140.9	26071.4					140.9	26071.4	247111.4	
MA0201000	PA NOVO ORIENTE	1998.9	369913.5	0.0	0.0	204.6	37859.8					204.6	37859.8	407773.3	
MA0202000	PA CAXIXÍ	0.0	0.0	255.6	39121.1			2731.4	369881.8			2731.4	369881.8	409002.9	
MA0203000	PA PARAISO	752.0	110403.7	459.8	67500.0					1841.2	270304.1	1841.2	270304.1	448207.8	
MA0204000	PA SANTA AMÉLIA	4499.5	684809.3	379.0	57970.6	7495.7	1145619.7	1038.9	23740.1			8534.6	1169359.8	1912139.7	
MA0205000	PA MARACAJÁ III	1061.2	62988.3	255.1	31660.4					990.5	124029.3	990.5	124029.3	218678.0	
MA0206000	PA CENTRO NOVO	104.7	15364.4	120.1	17633.1					626.3	91944.3	626.3	91944.3	124941.8	
MA0207000	PA NOGUEIRA	181.0	26577.3	710.5	104305.7					1410.2	207024.5	1410.2	207024.5	337907.5	
MA0208000	PA BACURI	235.1	34520.7	366.8	53857.5					298.9	43876.4	298.9	43876.4	132254.6	
MA0209000	PA MINA DE PRATA	1032.0	77520.0	0.0	0.0	1.3	128.7	1305.5	22823.9			1306.9	22952.6	100472.7	
MA0210000	PA ANDIROBAL	1958.3	313626.4	0.0	0.0							0.0	0.0	313626.4	36.4
MA0212000	PA JAGUARANA/FLORESTA	0.0	0.0	1257.6	184635.5			3350.4	491869.2			3350.4	491869.2	676504.8	

MA0213000	PA LADEIRA VERMELHA COCO GRANDE	3.6	532.8	1505.6	221031.5			4568.4	670681.7			4568.4	670681.7	892246.0	
MA0214000	PA SANTANA MORRINHOS	0.0	0.0	607.2	89137.6			7014.3	1029762.2			7014.3	1029762.2	1118899.8	
MA0215000	PA PREGUIÇA	1649.3	276905.4	0.0	0.0							0.0	0.0	276905.4	
MA0216000	PA QUADRA 10 DE AGOSTO	947.6	159096.5	0.0	0.0	38.2	6410.6					38.2	6410.6	165507.1	
MA0217000	PA QUADRA BARRAQUINHA	1136.4	190786.6	0.0	0.0	54.2	9107.5					54.2	9107.5	199894.1	
MA0218000	PA QUADRA UNIÃO	843.2	104628.6	0.0	0.0							0.0	0.0	104628.6	
MA0219000	PA OLHO D ÁGUA	823.1	119672.9	162.6	23345.0	467.5	66002.3	482.4	70570.6	384.9	56175.6	1334.7	192748.5	335766.5	
MA0220000	PA GAIOLA GRANDE	316.1	52597.2	0.0	0.0	750.5	124269.3			56.2	9395.7	806.7	133665.1	186262.2	
MA0221000	PA MARACAJÁ I	2220.6	94229.7	226.9	27961.9					1159.7	119801.6	1159.7	119801.6	241993.2	
MA0222000	PA CALIFORNIA	5822.4	1077256.1	0.0	0.0	70.2	12983.0			46.8	8652.7	116.9	21635.7	1098891.8	
MA0223000	PA FLOR DO VALE	2791.1	431188.6	0.0	0.0	191.4	30312.5	460.3	27006.7	35.4	5942.4	687.1	63261.6	494450.2	938.1
MA0225000	PA OUTEIRO	18224.9	1401475.3	0.0	0.0	589.4	105717.7			668.5	122807.2	1257.9	228524.9	1630000.2	1027.1
MA0226000	PA BRAÇO GRANDE/LAGO AZUL	2415.5	447350.2	0.0	0.0	280.7	52055.9			303.2	56286.0	584.0	108341.9	555692.1	
MA0228000	PA BOCA DA MATA	2125.9	305614.9	619.0	88258.1	2287.3	324816.5	39.3	5720.0	295.8	41640.5	2622.4	372176.9	766050.0	
MA0229000	PA SÃO PATRÍCIO	914.7	132431.8	400.8	54586.0					194.2	19450.6	194.2	19450.6	206468.3	
MA0232000	PA SANTA RITA DOS MATıES	145.5	21362.2	486.3	71400.3					2086.1	306265.3	2086.1	306265.3	399027.8	
MA0233000	PA TABOCÃO	6247.2	1167012.9	0.0	0.0	3990.0	745687.7			40.4	7541.3	4030.3	753229.1	1920242.0	
MA0234000	PA 11 DE JUNHO	6348.4	1185775.1	0.0	0.0	2651.6	495479.8			87.3	16317.0	2738.9	511796.8	1697572.0	
MA0235000	PA 07 DE MAIO	10024.6	1871211.1	0.0	0.0	3550.1	663460.7			36.4	6798.8	3586.4	670259.5	2541470.6	
MA0236000	PA BRASILÂNDIA	6861.6	1280919.7	0.0	0.0	4156.5	776769.1			44.3	8287.1	4200.9	785056.2	2065975.9	
MA0237000	PA PALMARES	0.0	0.0	801.4	112364.6			1337.5	191922.4			1337.5	191922.4	304286.9	
MA0239000	PA GALO	3086.2	518145.6	0.0	0.0	394.1	66166.5					394.1	66166.5	584312.2	
MA0240000	PE REMANSO	1770.2	265199.5	92.9	13912.8	90.7	13585.0	3.2	477.8			93.9	14062.7	293175.1	
MA0245000	PE STA ZITA	456.8	76684.2	0.0	0.0							0.0	0.0	76684.2	
MA0248000	PE QUINTO BRAÇO	5071.2	938478.6	0.0	0.0	4.0	749.3			0.4	70.3	4.4	819.6	939298.2	
MA0249000	PE NOVA CONQUISTA	24693.6	4570945.3	0.0	0.0	346.3	64236.4			606.6	112789.9	952.9	177026.3	4747971.6	
MA0250000	PE IGARAPÉ GRANDE	15321.2	2835347.0	0.0	0.0	39.5	7318.9			8.7	1612.4	48.2	8931.4	2844278.3	

MA0251000	PE EBENEZIA	8685.1	1581363.8	0.0	0.0							0.0	0.0	1581363.8	
MA0253000	PE ANAJATIUA-ARRAIAL	302.7	48591.6	0.0	0.0	222.1	36909.9			132.2	21233.3	354.2	58143.2	106734.8	
MA0256000	PE VILA PINDARÉ	13691.8	2462229.8	0.0	0.0	434.3	80380.4			68.2	12624.1	502.6	93004.5	2555234.3	
MA0258000	PE GUAIRACÁ	11294.0	2090063.5	0.0	0.0	466.8	86378.0			32.4	5991.6	499.1	92369.7	2182433.2	
MA0260000	PE NOVA OLINDA	56484.9	10279976.9	0.0	0.0							0.0	0.0	10279976.9	
MA0266000	PE SAMBRA	78.4	14544.7	0.0	0.0	145.0	27083.8					145.0	27083.8	41628.5	
MA0267000	PE SANTA LUCIA	11561.7	2103443.0	0.0	0.0	13.1	2422.6			21.6	3995.6	34.7	6418.2	2109861.1	
MA0275000	PE JAMARI DOS PRETOS	9974.2	1780208.7	0.0	0.0	2466.9	456326.9			2238.0	414617.8	4704.9	870944.8	2651153.5	
MA0278000	PE ROSÁRIO/CENTRO DO BATISTA	5604.5	894967.1	0.0	0.0			463.1	16499.9			463.1	16499.9	911467.1	
MA0279000	PA BOM JESUS	265.0	44484.6	0.0	0.0							0.0	0.0	44484.6	
MA0280000	PA SANTANA DOS PRETOS	1287.9	216226.5	0.0	0.0	214.6	36037.2					214.6	36037.2	252263.7	
MA0281000	PA VARIG	18231.8	3327815.7	0.0	0.0	2530.7	452425.6			180.9	33799.4	2711.6	486224.9	3814040.6	
MA0282000	PA BOA VISTA	4919.4	886906.8	0.0	0.0					0.1	10.4	0.1	10.4	886917.3	
MA0283000	PA SÃO JORGE	4693.9	856352.8	0.0	0.0	139.1	25521.6					139.1	25521.6	881874.3	
MA0286000	PA QUADRA SÃO JOSÉ	1104.1	185361.0	0.0	0.0							0.0	0.0	185361.0	
MA0287000	PA QUADRA 5 DE OUTUBRO	406.3	61733.1	0.0	0.0	52.3	8141.3					52.3	8141.3	69874.4	
MA0288000	PA QUADRA NOVO HORIZONTE	684.9	114985.6	0.0	0.0							0.0	0.0	114985.6	
MA0289000	PA QUADRA JUVENAL GERÔNIMO	250.6	45972.0	0.0	0.0							0.0	0.0	45972.0	
MA0291000	PA QUADRA FEITOSA	444.3	82228.9	0.0	0.0							0.0	0.0	82228.9	
MA0292000	PA QUADRA SÃO BENEDITO	407.9	68474.4	0.0	0.0	15.5	2600.8					15.5	2600.8	71075.2	
MA0293000	PA PINGO DE OURO	1816.5	272178.4	0.0	0.0	22.4	3349.2			123.5	18498.7	145.8	21847.9	294026.4	
MA0294000	PA MONTE CASTELO	1197.5	179396.7	22.2	3321.2	27.4	4109.5					27.4	4109.5	186827.4	
MA0295000	PA BOM SOSSEGO	76.0	8946.3	0.0	0.0			137.8	5311.2			137.8	5311.2	14257.5	0.2
MA0297000	PA ALENCARINA	3079.2	569831.6	0.0	0.0	303.5	56159.3			100.4	18571.0	403.8	74730.3	644561.9	
MA0298000	PA PIRAPEMAS	1219.7	49532.3	187.4	27323.6					558.7	39468.3	558.7	39468.3	116324.2	
MA0301000	PA SÃO JOÃO DO VALE VERDE	2420.7	447979.8	0.0	0.0	35.1	6499.5			43.3	8009.6	78.4	14509.1	462488.9	

MA0302000	PA PORCO MORTO	3239.8	598222.6	0.0	0.0							0.0	0.0	598222.6	
MA0304000	PA REGALO/SÃO JOÃO	0.0	0.0	167.8	24631.1			2634.8	386818.7			2634.8	386818.7	411449.8	
MA0306000	PA MARAVILHA	816.3	122294.3	635.2	95158.2	104.3	15620.7	524.0	78507.7			628.3	94128.4	311580.9	
MA0308000	PA LAGO DO COCO	1202.3	176505.1	62.5	9168.9	31.9	4679.2			78.6	11540.0	110.5	16219.3	201893.4	
MA0309000	PA CHAPADINHA	245.9	36818.4	1704.7	250726.0	178.6	26752.6	1309.6	188428.1			1488.2	215180.7	502725.0	
MA0310000	PA CONCEIÇÃO DO SALAZAR	451.8	66328.7	630.7	92598.2					3223.0	473165.7	3223.0	473165.7	632092.6	
MA0311000	PA PONTA DÁGUA	485.4	68265.0	173.0	13890.9	1238.6	179690.2	3170.1	75889.8			4408.7	255580.0	337736.0	
MA0312000	PA SÃO BENEDITO I	1317.1	197470.0	0.0	0.0	12.7	1901.6			130.0	19479.2	142.7	21380.7	218850.7	
MA0314000	PA CIGANA/SANTA CATARINA	546.1	77097.3	499.3	62962.0	31.1	4562.2			731.8	100350.1	762.9	104912.3	244971.6	
MA0315000	PA ALTA FLORESTA	888.9	164765.9	0.0	0.0	30.0	5578.1			77.5	14444.0	107.5	20022.1	184788.0	
MA0316000	PA ALVORADA III	3618.6	491022.0	0.0	0.0	36.2	3601.7					36.2	3601.7	494623.7	
MA0317000	PA JABUTI	781.6	144644.1	4.8	890.0	12.7	2342.8			26.5	4911.1	39.2	7253.9	152788.1	
MA0318000	PA BOA ESPERANÇA	988.6	182945.1	0.0	0.0							0.0	0.0	182945.1	
MA0319000	PA JATOBÁ	4938.3	913884.7	0.0	0.0	31.8	5892.9					31.8	5892.9	919777.7	
MA0320000	PA CONCEIÇÃO ROSA	298.6	43843.3	224.0	32892.0	509.8	74841.6	528.3	77560.2	112.4	16500.7	1150.5	168902.5	245637.7	
MA0322000	PA AGROALEGRE	2895.2	405987.6	479.1	36250.9	0.0	2.4	98.8	8711.2	521.6	41114.0	620.4	49827.6	492066.2	
MA0326000	PA LAGO VERDE	190.0	17888.7	330.3	38520.2					270.5	32075.4	270.5	32075.4	88484.4	27.0
MA0327000	PA SÃO BENEDITO	1718.0	269933.9	19.9	2942.2	3474.7	547007.6	517.1	76372.8	551.8	88013.3	4543.6	711393.8	984269.8	
MA0329000	PCA CAJUÍ	178.7	26232.5	39.4	5791.0					0.0	0.4	0.0	0.4	32023.9	
MA0331000	PCA INGÁ	92.8	13621.6	101.8	14942.7					90.8	13331.8	90.8	13331.8	41896.2	
MA0333000	PA BOA UNIÃO	149.9	4952.9	30.2	4437.8			138.2	11908.4			138.2	11908.4	21299.1	
MA0334000	PA BANDEIRANTES	1607.8	279466.2	0.0	0.0	1196.3	202166.6			372.0	63116.9	1568.2	265283.5	544749.8	
MA0335000	PA ROQUE/SANTA TEREZA	2602.1	436873.0	0.0	0.0	160.9	27016.0					160.9	27016.0	463889.0	
MA0336000	PA TARUMÃ	387.4	4849.7	0.0	0.0	3.1	39.2	3360.8	42077.1			3363.9	42116.4	46966.1	
MA0337000	PA BREJO DA ILHA	0.0	0.0	1207.9	174326.7			2906.1	310969.4			2906.1	310969.4	485296.1	
MA0339000	PA ABELARDO RIBEIRO	3440.3	563877.2	0.0	0.0	3.2	378.3	322.5	38595.7			325.7	38974.0	602851.2	
MA0340000	PA LAGOA SECA	240.0	28107.5	190.2	27613.9					498.7	65571.9	498.7	65571.9	121293.3	
MA0341000	PA CENTRINHO/CARMO	131.7	19332.2	168.3	24705.7					258.3	37914.9	258.3	37914.9	81952.9	

MA0343000	PA SINCORÁ	0.0	0.0	0.0	0.0			1217.0	15237.4			1217.0	15237.4	15237.4	299.3
MA0344000	PA BELA VISTA	30.3	1614.7	0.0	0.0			875.8	49987.2			875.8	49987.2	51601.9	40.8
MA0345000	PA MATA DE ANA	256.7	28821.6	0.0	0.0							0.0	0.0	28821.6	
MA0346000	PA CIKEL	10672.6	1994121.5	0.0	0.0	1624.4	303577.1			10.8	2019.5	1635.2	305596.6	2299718.1	
MA0347000	PA SÃO JOSÉ DO LAGO AÇÚ	8567.2	579962.4	0.0	0.0			284.9	3567.2			284.9	3567.2	583529.5	2787.0
MA0348000	PA SANTA LÚCIA	0.0	0.0	1896.6	146787.0			4422.2	343438.8			4422.2	343438.8	490225.8	
MA0349000	PA SOL BRILHANTE	4517.2	711802.3	0.0	0.0	221.8	33233.7			6.6	986.5	228.4	34220.3	746022.5	
MA0353000	PA PRIMEIRO DE MAIO	1251.7	165709.9	45.4	5831.5	115.2	14616.1					115.2	14616.1	186157.5	
MA0355000	PA LAGOA DA CIGANA	2937.5	516104.3	0.0	0.0	27.2	4203.4			34.0	5129.4	61.2	9332.8	525437.1	
MA0357000	PA BRAÇO FORTE	0.0	0.0	752.8	110594.4			830.0	121868.6			830.0	121868.6	232462.9	41.7
MA0397000	PA GIKI	0.0	0.0	309.8	10221.3			2857.0	113954.6			2857.0	113954.6	124175.9	
MA0398000	PE VAMESA	1040.1	133393.2	0.0	0.0	564.7	84374.8	7982.6	184596.8	13.2	2208.1	8560.5	271179.7	404572.9	
MA0399000	PE FORTUNA	865.6	28555.1	130.4	4281.2	479.6	15663.4	1323.9	31519.1			1803.5	47182.6	80018.9	
MA0401000	PA SERRA NEGRA II	0.0	0.0	673.7	98912.1	1.2	173.6	2690.7	395016.9			2691.9	395190.5	494102.6	
MA0404000	PA SANTA MÔNICA	101.5	14894.1	697.4	102390.5					343.9	50490.3	343.9	50490.3	167774.9	
MA0406000	PA MATA FOME	2181.8	263774.6	1476.1	162406.8					2272.8	269676.3	2272.8	269676.3	695857.7	25.0
MA0408000	PA EDITH	2320.0	390589.0	0.0	0.0							0.0	0.0	390589.0	
MA0410000	PA GUAIBA/SANTA ANGÉLICA II	1664.1	307957.2	0.0	0.0	2.0	375.0			8.5	1565.6	10.5	1940.5	309897.8	
MA0411000	PA MARAJÓ/ SANTA ANGÉLICA III	1735.0	323094.5	0.0	0.0	94.7	17661.5			685.6	127814.4	780.4	145475.9	468570.4	
MA0415000	PA ROSA SARAIVA	8765.9	1622745.8	0.0	0.0	255.2	47251.8			336.6	62303.6	591.8	109555.4	1732301.2	
MA0416000	PA LIMÃO	402.0	59023.9	222.8	32713.2					1052.6	154537.8	1052.6	154537.8	246274.8	
MA0417000	PA REMY SOARES	570.6	85478.5	148.5	22253.0	691.9	103651.9	87.2	13066.9			779.1	116718.8	224450.3	
MA0419000	PA MARAJÁ DO VELOSO	462.9	69360.5	1535.7	227185.1					125.4	18654.8	125.4	18654.8	315200.4	
MA0420000	PA VILA NOVA DE ANA DIAS	4318.3	678542.1	0.0	0.0	136.0	22833.8					136.0	22833.8	701375.9	
MA0421000	PA LIVRAMENTO	3322.7	557853.8	0.0	0.0							0.0	0.0	557853.8	
MA0422000	PA OLHO D ÁGUA/TREZE DE MAIO	921.6	154734.5	0.0	0.0							0.0	0.0	154734.5	
MA0423000	PA CAMPONÊS	843.8	157638.7	0.0	0.0	388.1	72531.9			5.8	1076.4	393.9	73608.4	231247.1	

MA0427000	PA AMOR DA PÁTRIA	194.7	29170.4	397.5	59550.3	56.1	8398.6	530.6	79490.3			586.7	87888.8	176609.6	
MA0428000	PA SANTA ANGÉLICA	2622.1	485503.8	0.0	0.0	94.0	17445.1			132.0	24506.1	226.0	41951.3	527455.0	
MA0429000	PA SEGUNDA CONQUISTA	403.4	67798.5	0.0	0.0			310.2	54363.3			310.2	54363.3	122161.8	6.0
MA0431000	PA NOSSA SENHORA APARECIDA	4089.4	720008.7	0.0	0.0	683.3	107872.9					683.3	107872.9	827881.6	
MA0432000	PA RIACHÃO	306.3	18797.8	0.0	0.0			968.9	12159.7			968.9	12159.7	30957.5	22.2
MA0433000	PA SIMAÚMA	103.4	15676.9	0.0	0.0			740.0	18484.8			740.0	18484.8	34161.7	102.5
MA0435000	PA UNIÃO	345.2	57488.0	0.0	0.0	181.3	29775.5					181.3	29775.5	87263.4	
MA0437000	PA TAUÁ DO CENTRO/GAIOLA GRANDE	56.2	9359.7	0.0	0.0	53.5	8705.5	1476.5	184617.1	8.4	1260.8	1538.4	194583.4	203943.1	4.3
MA0438000	PA PIQUI	148.1	23319.8	8.8	1299.1	276.4	42215.2					276.4	42215.2	66834.0	3.9
MA0440000	PCA JOSÉ SARNEY COSTA	47.5	6979.4	26.1	3834.5					28.1	4130.1	28.1	4130.1	14944.0	
MA0443000	PA JORDÃO	1576.9	81932.3	40.5	3157.9					543.7	35289.9	543.7	35289.9	120380.0	
MA0444000	PA TERRAS BELAS	444.7	63517.6	0.0	0.0			31.1	562.3			31.1	562.3	64079.9	45.1
MA0445000	PA BOA ESPERANÇA DOS OLIVEIRAS	1161.7	190968.6	0.0	0.0	843.9	140312.7	1928.4	76976.0	45.5	7642.9	2817.9	224931.6	415900.2	281.9
MA0447000	PA NOSSA SENHORA DA CONCEIÇÃO	0.0	0.0	1487.4	117820.7			1588.9	128453.4			1588.9	128453.4	246274.1	
MA0448000	PA SULNORBRAZ/AGRO TERRA	3531.1	657398.2	14.2	2646.2	2057.2	384349.0			1509.0	281926.0	3566.3	666275.0	1326319.4	
MA0450000	PA PLANADA BOA VISTA	449.9	83267.7	0.0	0.0							0.0	0.0	83267.7	
MA0453000	PA SAGRADO CORAÇÃO DE JESUS	776.8	124535.5	0.0	0.0							0.0	0.0	124535.5	3.5
MA0454000	PA SÃO JOSÉ DA VITÓRIA	573.4	20745.7	29.1	1981.3					407.4	18984.7	407.4	18984.7	41711.7	
MA0456000	PA AMAZONIA	3719.5	694800.8	0.0	0.0	837.1	156436.7			46.7	8734.7	883.8	165171.4	859972.2	
MA0458000	PE SANTA CLARA	3089.3	571712.4	0.0	0.0	163.9	30326.3					163.9	30326.3	602038.7	
MA0462000	PA GURUPI	5673.0	1057282.9	0.0	0.0	3071.7	572023.7			54.1	10076.3	3125.8	582099.9	1639382.8	
MA0463000	PA CURITIBA	0.0	0.0	1059.1	81806.5			488.8	37751.3			488.8	37751.3	119557.8	
MA0464000	PA FLEXAS/GLEBA JURITI	1702.8	317537.2	0.0	0.0	597.4	111640.2					597.4	111640.2	429177.3	
MA0468000	PE MUTUTI	1293.0	239280.1	0.0	0.0	4.5	831.6			106.1	19639.4	110.6	20471.1	259751.2	
MA0477000	PE CAJUAL	1511.8	279764.9	0.0	0.0							0.0	0.0	279764.9	
MA0479000	PA PAU DE ESTOPA	967.9	31930.7	5.6	184.2					222.3	7332.7	222.3	7332.7	39447.6	

MA0480000	PA SERAFIM	0.0	0.0	766.8	112573.3			516.2	75780.4			516.2	75780.4	188353.7	
MA0481000	PA FLORESTA I	1209.3	184660.2	104.0	15871.1	54.5	8338.6			13.6	2072.3	68.1	10410.8	210942.2	
MA0483000	PA CAJUEIRO I	690.3	126700.4	92.0	16020.0	93.0	16705.3	451.9	27553.7			544.9	44259.0	186979.3	
MA0484000	PA SÃO RAIMUNDO	7311.2	1365665.4	0.0	0.0	2553.4	477157.9					2553.4	477157.9	1842823.3	
MA0485000	PE SITIO DOS ARRUDAS	0.0	0.0	187.8	4821.2			12692.1	283205.7			12692.1	283205.7	288026.9	
MA0486000	PE MUCUNÃ	530.1	79415.4	159.1	23829.4	257.9	38642.4	935.0	140071.3			1192.9	178713.7	281958.5	
MA0489000	PA DURVAL NETO	1342.3	197913.3	91.3	13532.6	367.1	54797.1	542.7	78034.2			909.8	132831.3	344277.2	
MA0490000	PA SANTO ANTONIO II	800.5	123662.5	312.3	46152.5	1271.0	198411.1	546.6	80289.5			1817.6	278700.6	448515.6	0.2
MA0493000	PA NOVA VIDA	2455.8	93801.2	781.0	32609.9					905.3	54840.5	905.3	54840.5	181251.6	
MA0502000	PA CHICO MENDES/BANANEIRAS	4778.8	808633.9	0.0	0.0	845.4	157372.1					845.4	157372.1	966006.0	
MA0503000	PA SANTA LUZIA	2474.0	327406.0	115.3	14784.1	54.1	4788.2	23.8	2887.7			77.9	7675.9	349866.0	
MA0504000	PA BOA VISTA I	0.0	0.0	1308.4	101058.4			983.9	75997.2			983.9	75997.2	177055.6	
MA0507000	PA ORCAISA	619.4	90949.2	630.3	92541.2					2867.0	420924.8	2867.0	420924.8	604415.2	
MA0508000	PA CASA BRANCA	1163.7	190628.0	0.0	0.0	837.7	136527.7			45.2	7361.2	882.9	143888.9	334516.9	
MA0510000	PA VENEZA	275.7	51534.3	0.0	0.0	104.9	19604.9					104.9	19604.9	71139.3	
MA0511000	PA SANTA ISABEL	2164.6	404488.0	0.0	0.0	477.5	89238.2					477.5	89238.2	493726.2	
MA0512000	PE JUÇARAL	37.6	5295.3	72.9	10250.5	131.5	18496.6			122.0	17169.8	253.5	35666.4	51212.2	
MA0520000	PA SOSSEGO	197.1	28938.9	433.2	63600.8					1113.9	163538.4	1113.9	163538.4	256078.1	
MA0521000	PA TINGIDOR	657.2	110337.3	0.0	0.0	3168.7	531992.3			1225.3	205715.9	4394.0	737708.2	848045.5	9.1
MA0523000	PA LAGO DA CARNAÚBA	9250.1	1512765.8	0.0	0.0							0.0	0.0	1512765.8	131.4
MA0524000	PA NOVA SALVAÇÃO	1308.1	193895.8	857.0	126939.2					3389.8	497293.4	3389.8	497293.4	818128.4	
MA0526000	PA ROSELI NUNES/BANANEIRAS	4359.3	810835.8	0.0	0.0	1950.0	352173.2					1950.0	352173.2	1163009.0	
MA0530000	PA SÃO JOÃO	0.0	0.0	229.5	17727.3			793.2	61266.4			793.2	61266.4	78993.6	
MA0538000	PE VERA CRUZ I	4944.5	915045.2	0.0	0.0	1511.8	277181.8			3978.9	736943.0	5490.8	1014124.9	1929170.1	
MA0539000	PCA BATALHA	218.4	17985.0	2.2	169.2							0.0	0.0	18154.1	
MA0540000	PA CIPOAL	20176.8	3735820.4	0.0	0.0	1440.8	268322.2			2495.6	464322.6	3936.4	732644.8	4468465.1	
MA0541000	PA JAGUARIBE	1939.4	329508.4	0.0	0.0							0.0	0.0	329508.4	

MA0542000	PE MANAUS	2875.5	532370.6	0.0	0.0	133.0	24615.3			129.6	24009.6	262.6	48625.0	580995.5	
MA0546000	PA QUADRA BOA VIDA	297.7	49974.6	0.0	0.0	19.8	3327.4					19.8	3327.4	53302.0	
MA0548000	PA VERA CRUZ	868.2	127463.1	803.4	116621.9	237.0	34798.5	1945.9	192398.8			2182.9	227197.3	471282.4	24.4
MA0549000	PA SÃO FÉLIX	699.1	102641.5	324.5	47645.3	224.1	32893.6	260.0	38168.3			484.0	71061.9	221348.7	
MA0553000	PCA VASSORAL	114.2	19170.8	0.0	0.0							0.0	0.0	19170.8	
MA0555000	PA XIXANDÁ	1950.9	310822.7	0.0	0.0	161.7	24526.7	1383.3	146921.7			1544.9	171448.5	482271.1	
MA0557000	PA BOI BAIANO	2126.7	303162.0	222.8	32631.5	0.0	2.0			269.2	39514.5	269.2	39516.6	375310.0	54.3
MA0562000	PA CIGANA	193.2	28359.7	280.4	41172.3	900.9	132259.5	1370.0	201132.2	66.6	9778.7	2337.5	343170.4	412702.4	
MA0565000	PA PADRE TRINDADE	150.3	15791.4	255.9	34965.7					1036.1	130443.7	1036.1	130443.7	181200.8	
MA0566000	PA NOVO HORIZONTE II	241.9	40613.6	0.0	0.0							0.0	0.0	40613.6	
MA0570000	PA PADRE JOSINO	549.8	77342.9	137.1	19281.2	427.0	60076.1			131.8	18545.9	558.9	78622.0	175246.1	
MA0572000	PA ANGICO	0.0	0.0	110.9	16275.8			3143.1	463672.2			3143.1	463672.2	479948.0	
MA0573000	PA MARGARIDA MARIA ALVES	1.2	95.8	785.1	60644.4			626.0	48351.7			626.0	48351.7	109091.9	
MA0574000	PA LAGOA DA FORTUNA	13964.7	2088807.3	837.5	125409.7	5287.6	791427.0	220.9	33088.4			5508.5	824515.5	3038732.4	
MA0577000	PA SARAMANDAIA	1439.0	214666.7	261.0	37991.2	47.0	6912.3	4.2	391.7	52.0	7499.4	103.3	14803.4	267461.4	
MA0580000	PA PADRE JOSIMO I	1207.2	223413.0	0.0	0.0							0.0	0.0	223413.0	
MA0581000	PA BOA ESPERANÇA III	3570.5	665209.8	0.0	0.0	979.4	182965.9					979.4	182965.9	848175.7	
MA0583000	PA COCALINHO	718.9	133040.1	0.0	0.0							0.0	0.0	133040.1	
MA0584000	PA QUIXABA	498.1	84536.1	0.0	0.0							0.0	0.0	84536.1	
MA0592000	PA ALVORADA IV	3442.0	636163.2	0.0	0.0	2.2	401.0					2.2	401.0	636564.2	
MA0593000	PE MARACUJÁ	207.3	34529.0	0.0	0.0	49.2	7415.5			88.1	13713.3	137.3	21128.7	55657.7	
MA0600000	PE LARANJAL	961.9	161494.0	0.0	0.0							0.0	0.0	161494.0	
MA0602000	PE PROMISSÃO	179.7	27257.5	0.0	0.0							0.0	0.0	27257.5	
MA0603000	PE RIO GRANDE	273.5	45912.5	0.0	0.0	43.9	7368.9			33.8	5673.9	77.7	13042.8	58955.3	
MA0604000	PA CANAÃ	837.3	143114.7	0.0	0.0							0.0	0.0	143114.7	
MA0605000	PA BOA VISTA II	595.4	108232.5	0.0	0.0							0.0	0.0	108232.5	
MA0606000	PA EL BETEL	311.5	57304.6	0.0	0.0							0.0	0.0	57304.6	
MA0607000	PA CRUZEIRO DO SUL	1000.1	167108.0	0.0	0.0	112.9	17950.4					112.9	17950.4	185058.3	

MA0608000	PA 1‖ DE JUNHO	286.4	48090.7	0.0	0.0	0.1	22.5					0.1	22.5	48113.2	
MA0609000	PA SUCESSO	420.6	77841.3	0.0	0.0							0.0	0.0	77841.3	
MA0610000	PA IPIRANGA	202.0	37382.1	0.0	0.0							0.0	0.0	37382.1	
MA0611000	PA MONTE LÍRIO	692.4	128135.8	0.0	0.0							0.0	0.0	128135.8	
MA0612000	PA TAMANDARÉ	462.6	77950.5	0.0	0.0							0.0	0.0	77950.5	
MA0614000	PA FÉ EM DEUS	584.0	98047.4	0.0	0.0							0.0	0.0	98047.4	
MA0615000	PA BOA ESPERANÇA I	897.5	150675.6	0.0	0.0							0.0	0.0	150675.6	
MA0618000	PA VALE DO IPÊ	355.6	61090.2	0.0	0.0	22.7	3729.3					22.7	3729.3	64819.5	
MA0619000	PA ALTO BOA VISTA	1652.1	307334.1	0.0	0.0	360.5	67368.8			93.1	17403.0	453.6	84771.8	392106.0	
MA0633000	PA SACO DANTAS GUARIBAS	2017.9	295695.0	19.2	2751.1	0.8	115.2	215.6	31647.6	647.3	94272.8	863.6	126035.6	424481.7	
MA0634000	PA TICO TICO	187.3	27499.9	26.9	3945.3	2.2	315.8			116.3	17079.8	118.5	17395.7	48840.8	
MA0635000	PA 6 DE MARÇO	314.1	58133.0	0.0	0.0							0.0	0.0	58133.0	
MA0640000	PA REFUGIO	2323.7	390112.4	0.0	0.0	437.1	73388.4	7.4	908.4			444.6	74296.8	464409.2	759.5
MA0642000	PA ALTAMIRA	0.0	0.0	1861.2	268141.1			1425.<br>1	209192.5			1425.1	209192.5	477333.5	
MA0644000	PA SOL NASCENTE	0.0	0.0	1157.8	81409.4			1996.<br>7	137154.5			1996.7	137154.5	218563.9	
MA0645000	PA GOVERNADOR LUIS ROCHA	0.0	0.0	1374.9	105238.3			1981.<br>5	146364.3			1981.5	146364.3	251602.6	
MA0646000	PA ÁGUA FRIA DO ITINGA	6291.0	1047235.9	0.0	0.0	1150.2	187479.2					1150.2	187479.2	1234715.1	
MA0647000	PA AGRÍCOLA 2002	2470.3	442066.7	0.0	0.0	2.8	440.6	946.2	116937.6	30.7	4940.4	979.7	122318.5	564385.2	
MA0648000	PA PIMENTA	281.6	52115.8	0.0	0.0							0.0	0.0	52115.8	
MA0649000	PA QUADRA DIAMANTE	102.0	17132.5	0.0	0.0							0.0	0.0	17132.5	
MA0650000	PA BELÉM	590.7	99169.2	0.0	0.0							0.0	0.0	99169.2	
MA0651000	PA CUTIA/BAIÚNA	5487.7	1011494.4	0.0	0.0							0.0	0.0	1011494.4	
MA0654000	PA VALE DO BEKAA	1002.8	50729.0	40.7	2053.9					253.9	18206.1	253.9	18206.1	70988.9	
MA0655000	PA BURITIATÁ	858.4	141740.1	0.0	0.0	15.1	2535.1					15.1	2535.1	144275.2	
MA0656000	PE BOCAINA	521.7	76585.8	62.3	9139.4					1.3	191.4	1.3	191.4	85916.6	
MA0660000	PE BACURITUBA I	0.0	0.0	0.0	0.0			167.5	20519.1			167.5	20519.1	20519.1	
MA0666000	PA DEUS É FIEL	1119.2	190724.5	0.0	0.0	216.6	7022.5					216.6	7022.5	197747.0	
MA0668000	PA EL SHADAY	877.9	158161.4	0.0	0.0	110.6	9129.1					110.6	9129.1	167290.5	

MA0685000	PA QUADRA SÃO JOÃO BATISTA	189.4	31797.3	0.0	0.0							0.0	0.0	31797.3	
MA0686000	PA QUADRA SÃO RAIMUNDO	203.7	34192.8	0.0	0.0							0.0	0.0	34192.8	
MA0688000	PE LARANJEIRAS / MURTURA	101.1	16562.3	0.0	0.0	12.3	1898.9			6.8	1145.5	19.1	3044.5	19606.7	
MA0692000	PA PAULO FREIRE	0.0	0.0	196.8	28859.9			1136.1	139776.2			1136.1	139776.2	168636.1	
MA0698000	PA VITÓRIA	0.0	0.0	606.7	61046.9			698.7	67737.0			698.7	67737.0	128783.9	
MA0699000	PA NOVA VITÓRIA	0.0	0.0	399.0	7221.9			155.9	7283.4			155.9	7283.4	14505.3	0.2
MA0700000	PA PASSO LIVRE	2353.8	439909.1	0.0	0.0	3289.9	614850.9			9.5	1768.4	3299.4	616619.3	1056528.4	
MA0701000	PA MINADOR	0.1	9.8	381.9	56194.4			1049.5	155789.1			1049.5	155789.1	211993.3	
MA0704000	PA 17 DE ABRIL	0.0	0.0	162.0	21723.2			1138.3	77863.5			1138.3	77863.5	99586.6	
MA0725000	PA FELIZ SÃO FRANCISCO	257.8	43282.9	0.0	0.0							0.0	0.0	43282.9	
MA0726000	PA BOM JESUS II	364.5	53517.5	141.5	20779.9	629.7	92445.0	284.6	41778.6			914.3	134223.6	208521.1	
MA0727000	PA SÃO VICENTE	219.8	40674.7	0.0	0.0							0.0	0.0	40674.7	
MA0729000	PA BEBE ÁGUA	239.4	40200.8	0.0	0.0							0.0	0.0	40200.8	
MA0730000	PA TRÊS PALMEIRAS	201.2	33781.7	0.0	0.0	4.3	725.8					4.3	725.8	34507.5	
MA0746000	PE BURITIRANA I	0.0	0.0	15417.9	729958.2			43380.5	1141540.5			43380.5	1141540.5	1871498.7	
MA0747000	PA RAIMUNDO PANELADA/SIMASA	5345.1	989164.8	0.0	0.0	1483.6	274554.9			166.4	30792.6	1650.0	305347.5	1294512.3	
MA0748000	PA SÃO JOÃO I	771.1	123328.3	0.0	0.0	5.9	880.8			38.1	5711.4	44.0	6592.2	129920.5	
MA0749000	PA IMPERIAL	2472.7	370432.1	5.5	817.3	24.6	3680.0					24.6	3680.0	374929.4	
MA0756000	PA OLGA BENÁRIO	0.0	0.0	877.8	76903.9			1777.1	192930.3			1777.1	192930.3	269834.2	
MA0757000	PA SÃO RAIMUNDO II	710.5	104314.4	20.0	2941.4					294.3	43209.0	294.3	43209.0	150464.7	6.4
MA0758000	PA JEOVAH	3174.8	533016.7	0.0	0.0							0.0	0.0	533016.7	
MA0767000	PA BRASILÂNDIA I	3176.9	165305.0	91.0	10095.7					963.8	103468.7	963.8	103468.7	278869.4	
MA0768000	PA SÃO JOSÉ / SATUBINHA	626.2	91538.6	0.0	0.0			1380.8	27987.8			1380.8	27987.8	119526.4	
MA0769000	PA SÃO BENEDITO DO ELCIAS	303.5	45146.0	272.1	40015.0					495.4	72829.8	495.4	72829.8	157990.8	
MA0771000	PA PADRE PAULO	3515.5	583119.2	0.0	0.0	179.2	23037.7	6.7	1118.6	23.7	3202.1	209.5	27358.5	610477.6	47.0
MA0772000	PA LAGOA DOS PATOS	1088.8	202276.3	0.0	0.0	177.4	33017.9			446.7	83157.7	624.1	116175.7	318451.9	
MA0773000	PA DALBAM	2471.5	414944.3	0.0	0.0							0.0	0.0	414944.3	

MA0775000	PA VIDA NOVA	0.0	0.0	1360.1	56405.7			4939.1	358440.5			4939.1	358440.5	414846.1	
MA0782000	PA MANGAUBA	165.3	27689.3	0.0	0.0	116.1	18576.5	3.2	200.7	17.3	2912.8	136.7	21690.0	49379.3	
MA0783000	PA CIGANA/BARRIGUDO/COLONE	144.1	24189.0	0.0	0.0	25.0	4201.0					25.0	4201.0	28390.0	
MA0800000	PA MUNDO NOVO	0.0	0.0	242.3	35577.4			1299.5	190775.7			1299.5	190775.7	226353.1	
MA0820000	PE BOA VISTA III	4378.7	743310.6	0.0	0.0	234.2	27018.5			190.3	23533.1	424.6	50551.6	793862.2	42.9
MA0821000	PE ENCRUSO	366.3	61502.9	0.0	0.0	3023.2	507566.2	177.4	29788.5	804.7	135105.1	4005.4	672459.8	733962.6	4.1
MA0834000	PA MONTE CRISTO	2574.4	389183.8	821.2	121785.8					3792.7	558788.3	3792.7	558788.3	1069757.9	
MA0835000	PA TAMBOR CENTRO VELHO	3268.9	511111.1	0.0	0.0	8.9	1249.2	5186.0	422617.9			5194.9	423867.0	934978.1	
MA0836000	PA DEUS PROTEJA	1619.1	300876.1	0.0	0.0	651.7	121790.6					651.7	121790.6	422666.7	
MA0840000	PA BARROCA DOS VEADOS	474.8	22395.4	19.7	1765.3					703.7	60056.5	703.7	60056.5	84217.2	
MA0842000	PA SÃO FRANCISCO I	1154.2	192112.2	0.0	0.0	1320.5	216629.9			201.2	33726.4	1521.7	250356.4	442468.5	77.9
MA0855000	PA CRISTINA ALVES	848.2	124513.3	278.1	40833.7	873.1	128178.5	2682.4	393803.2	130.8	19129.3	3686.3	541110.9	706457.9	
MA0859000	PA SÃO JOÃO DO ROSÁRIO	672.9	112193.5	0.0	0.0	1973.1	312327.7			1031.4	165097.1	3004.4	477424.8	589618.3	8.6
MA0860000	PA PRESIDENTE LULA	1782.7	331012.5	0.0	0.0	789.4	146516.1					789.4	146516.1	477528.6	
MA0864000	PA CAMPO VERDE / COLONE	300.5	50451.4	0.0	0.0							0.0	0.0	50451.4	
MA0865000	PA NOVA CANAÃ / COLONE	218.0	36600.8	0.0	0.0							0.0	0.0	36600.8	
MA0866000	PA SANTO ANTONIO III	255.1	42832.9	0.0	0.0							0.0	0.0	42832.9	
MA0869000	PA ALTO TURI III	23045.3	4265106.1	0.0	0.0	95.0	17589.0			35.3	6545.7	130.4	24134.6	4289240.8	
MA0870000	PA ALTO TURI II	19744.1	3653870.5	0.0	0.0	76.2	14112.8			12.5	2322.9	88.8	16435.6	3670306.1	
MA0871000	PA CENTRO DO ELIAS / BREJO DO LUCAS / COLONE	9273.4	1716143.8	0.0	0.0	87.4	16167.1			1.2	218.2	88.5	16385.2	1732529.1	
MA0872000	PA QUADRA ÁGUA AZUL / COLONE	4513.4	835255.9	0.0	0.0	257.9	47724.2			405.3	75005.2	663.2	122729.4	957985.3	
MA0873000	PA QUADRA 21 DE ABRIL / COLONE	5852.8	1083121.8	0.0	0.0	55.8	10329.6			48.6	9002.6	104.5	19332.2	1102454.0	
MA0874000	PA UNIÃO/SANTO ANTONIO / COLONE	6572.3	1216266.1	0.0	0.0	73.4	13590.8			119.4	22097.8	192.8	35688.6	1251954.7	
MA0876000	PA FORTAL/COLONE	270.2	50001.7	0.0	0.0							0.0	0.0	50001.7	
MA0877000	PA TERRA PROMETIDA / COLONE	738.2	136323.9	0.0	0.0							0.0	0.0	136323.9	
MA0878000	PA SÃO LUIS /	147.6	24784.2	0.0	0.0							0.0	0.0	24784.2	

	COLONE														
MA0879000	PA CENTRO DO PEDRO / BOM JESUS DA MATA/COLONE	4139.6	766074.1	0.0	0.0							0.0	0.0	766074.1	
MA0880000	PA VILA ESPERANÇA/COLON E	95.5	16026.0	0.0	0.0							0.0	0.0	16026.0	
MA0881000	PA QUADRA BETEL / COLONE	3613.9	668792.9	0.0	0.0	17.5	3247.2					17.5	3247.2	672040.1	
MA0882000	PA QUADRA CANAÃ/COLONE	2703.9	469788.4	0.0	0.0							0.0	0.0	469788.4	
MA0883000	PA QUADRA JERICÓ/COLONE	3068.4	530690.1	0.0	0.0							0.0	0.0	530690.1	
MA0884000	PA QUADRA BELÉM / COLONE	2666.6	486522.0	0.0	0.0							0.0	0.0	486522.0	
MA0885000	PA SANTA CLARA / COLONE	209.5	35173.3	0.0	0.0							0.0	0.0	35173.3	
MA0886000	PA ALTO ALEGRE / COLONE	576.0	94406.6	0.0	0.0							0.0	0.0	94406.6	
MA0887000	PA QUADRA BOA ESPERANÇA / COLONE	3627.8	671352.2	0.0	0.0							0.0	0.0	671352.2	
MA0888000	PA QUADRA SÃO FRANCISCO / COLONE	2844.9	526485.9	0.0	0.0	213.4	39484.2			357.9	66230.0	571.2	105714.2	632200.1	
MA0889000	PA QUADRA BRASIL / COLONE	4073.5	753850.9	0.0	0.0					0.9	170.7	0.9	170.7	754021.7	
MA0890000	PA QUADRA PEDRO ALVARES CABRAL / COLONE	2971.1	549825.4	0.0	0.0	74.3	13749.8			167.9	31052.0	242.2	44801.8	594627.2	60.2
MA0891000	PA QUADRA DUQUE DE CAXIAS / COLONE	4963.2	918490.8	0.0	0.0							0.0	0.0	918490.8	
MA0892000	PA REDENÇÃO / BELÉM / COLONE	7557.0	1399046.4	0.0	0.0	195.2	36136.1			875.5	162211.5	1070.7	198347.7	1597394.1	
MA0893000	PA QUADRA JERUSALÉM / COLONE	3278.5	606718.6	0.0	0.0							0.0	0.0	606718.6	
MA0894000	PA SÃO FRANCISCO / COLONE	177.9	29862.7	0.0	0.0	1.0	125.0	58.3	9789.0			59.3	9914.0	39776.7	
MA0896000	PA MORADA NOVA/CENTRO DOS PINHEIROS / COLONE	4632.3	788902.6	0.0	0.0							0.0	0.0	788902.6	
MA0897000	PA PEDREIRA II / INGAZAL / COLONE	1272.2	197612.6	0.0	0.0							0.0	0.0	197612.6	
MA0898000	PA CENTRO DOS MARTINS / AGRICOLÂNDIA / COLONE	12309.4	2277975.9	0.0	0.0	95.1	17602.2			34.4	6364.4	129.5	23966.6	2301942.5	
MA0901000	PA QUADRA NOVA / 13 DE JUNHO /	3132.7	560996.7	0.0	0.0							0.0	0.0	560996.7	

	COLONE												
MA0902000	PA QUADRA BENEDITO MENDES / QUADRA FORTALEZA / COLONE	6261.8	1158807.3	0.0	0.0					0.0	0.0	1158807.3	
MA0906000	PA QUADRA SÃO RAIMUNDO III / COLONE	13969.6	2592686.8	0.0	0.0	1343.3	250228.2	2843.6	530174.8	4186.9	780402.9	3373089.8	60.7
MA0909000	PA QUADRA SABIÁ / COLONE	4965.6	918935.2	0.0	0.0	122.1	22599.2	248.6	46011.8	370.8	68611.0	987546.2	
MA0911000	PA ITAMATARÉ / COLONE	18544.7	3435857.1	0.0	0.0	1179.1	219036.8	3467.8	645245.8	4647.0	864282.6	4300139.6	224.1
MA0912000	PA PINDORAMA / ARARIBÓIA / COLONE	28953.7	5356124.2	0.0	0.0	437.0	80314.1	1174.8	215739.8	1611.8	296054.0	5652178.2	
MA0913000	PA 15 DE JUNHO / IGARAPÉ AREIA / SÃO JOSE II / COLONE	7578.0	1402388.3	0.0	0.0	60.0	11108.5	2.2	403.5	62.2	11512.0	1413900.3	
MA0914000	PA FÉ EM DEUS / COLONIAL / COLONE	15030.5	2781467.9	0.0	0.0					0.0	0.0	2781467.9	
MA0915000	PA JACI / COLONE	6588.9	1219344.2	0.0	0.0	32.7	6055.4	86.7	16045.6	119.4	22101.0	1241445.2	
MA0916000	PA SÃO JOÃO / 16 DE OUTUBRO / COLONE	4147.3	767508.4	0.0	0.0					0.0	0.0	767508.4	
MA0917000	PA NOVA VIDA / SERPETIARA / COLONE	4573.3	846326.5	0.0	0.0	262.2	48523.8	126.3	23375.9	388.5	71899.7	918226.2	3.6
MA0918000	PA QUADRA CASTELO BRANCO / COLONE	4731.5	875607.4	0.0	0.0	41.4	7651.7	238.1	44063.3	279.5	51715.0	927322.4	9.8
MA0921000	PA SANTA TEREZA / COLONE	138.8	20320.7	0.0	0.0	14.6	1780.4			14.6	1780.4	22101.1	10.2
MA0924000	PA SÃO JUDAS TADEU	726.6	121985.7	0.0	0.0	0.6	100.1			0.6	100.1	122085.8	47.3
MA0925000	PA CONCEIÇÃO I	847.8	142331.1	0.0	0.0					0.0	0.0	142331.1	
MA0926000	PA SÃO PEDRO / COLONE	381.3	64015.0	0.0	0.0					0.0	0.0	64015.0	
MA0927000	PA 22 DE SETEMBRO / SANTA TEREZA / COLONE	26534.5	4828204.8	0.0	0.0	10.7	1977.3	5.7	1052.6	16.4	3029.9	4831234.6	
MA0928000	PA CAFÉ DA MATA / COLONE	2955.3	456928.6	0.0	0.0					0.0	0.0	456928.6	
MA0929000	PA MATA AZUL / COLONE	1793.4	329928.0	0.0	0.0	322.8	57354.3	308.2	55790.6	630.9	113144.9	443072.9	
MA0931000	PA GRACILÂNDIA / CIDELÂNDIA / COLONE	15192.5	2806920.2	0.0	0.0	22.5	4067.6	168.7	29579.7	191.2	33647.2	2840567.5	
MA0932000	PA NADIR / SÃO VICENTE / COLONE	23197.7	4293618.7	0.0	0.0	808.6	149745.1	5540.5	1026901.9	6349.2	1176647.0	5470265.8	

MA0933000	PA SÃO FRANCISCO II / COLONE	148.3	26136.2	0.0	0.0							0.0	0.0	26136.2	
MA0934000	PA FELIZ UNIÃO / COLONE	324.3	60011.1	0.0	0.0							0.0	0.0	60011.1	
MA0935000	PA NOSSA VITÓRIA / COLONE	241.2	40874.9	0.0	0.0							0.0	0.0	40874.9	
MA0939000	PA LEELAU /CAJUEIRO/COLONE	1087.2	201201.0	0.0	0.0	0.0	0.9					0.0	0.9	201201.9	
MA0940000	PA ABAIXADINHO / COLONE	1245.4	200493.7	0.0	0.0	285.1	48353.4	1760.4	57308.1	162.4	28229.8	2207.9	133891.2	334384.9	
MA0941000	PA UBINZAL / COLONE	15333.3	2528622.0	0.0	0.0	106.1	17956.9	1082.8	19521.7	63.6	10177.7	1252.6	47656.3	2576278.2	
MA0943000	PA SÃO JOSÉ	0.0	0.0	133.0	2935.4			1296.5	27008.0			1296.5	27008.0	29943.3	
MA0944000	PA TATAJUBA / 10 DE ABRIL / COLONE	4127.6	710315.8	0.0	0.0	1.2	197.0			1.1	188.2	2.3	385.2	710701.0	
MA0945000	PA VILA NOVA / ÁGUA BRANCA	15.4	2579.6	0.0	0.0	132.0	20436.2	531.3	77678.6			663.3	98114.8	100694.4	
MA0946000	PA MARACAÇUMÉ / RICOA	1590.4	267013.9	0.0	0.0			9.5	1593.8			9.5	1593.8	268607.7	74.1
MA0947000	PA JUSSARAL DO ANTONIO ROXO	808.9	95530.0	0.0	0.0			845.0	13537.1			845.0	13537.1	109067.1	
MA0948000	PA CUBA	365.5	61366.9	0.0	0.0	375.4	63024.3	3.6	604.3			379.0	63628.6	124995.5	447.3
MA0954000	PA MAPISA	7013.1	1297838.0	0.0	0.0	2139.4	395924.3			32.9	6090.6	2172.3	402014.9	1699852.9	
MA0956000	PA ALEGRE	0.0	0.0	114.8	8866.4			1880.7	139576.3			1880.7	139576.3	148442.7	
MA0959000	PA BOCA DA MATA II	478.4	70880.1	34.9	5128.4	23.8	3490.5			98.0	14385.0	121.8	17875.4	93883.9	
MA0961000	PA MONTE ALEGRE / DENDÊ	324.7	22563.2	283.2	35220.3					572.8	67940.4	572.8	67940.4	125723.9	
MA0962000	PE TATAJUBA	3157.9	580934.0	0.0	0.0	865.7	159187.5			1453.2	267271.3	2318.9	426458.8	1007392.8	44.7
MA0963000	PA APARECIDA DO MEARIM	123.2	18858.8	578.8	88112.6	1946.6	297933.7	1693.8	259016.7			3640.4	556950.4	663921.8	
MA0965000	PA JIQUIRI / SANTO AGOSTINHO	1109.3	123821.8	0.0	0.0	35.0	4807.8			7.6	1274.7	42.6	6082.5	129904.4	87.6
MA0968000	PA AGAYNARA / SEMBAL	1111.6	43409.1	76.9	3349.4					388.2	34286.2	388.2	34286.2	81044.7	
MA0969000	PA FLECHAL	14612.1	2701023.4	0.0	0.0	1384.2	256615.1			1188.0	220120.8	2572.2	476735.9	3177759.3	
MA0973000	PA VILA NOVA	496.9	92863.5	0.0	0.0	1502.4	280774.4					1502.4	280774.4	373637.9	
MA0974000	PA NÚCLEO A - I / COLONE	9644.7	1766294.2	0.0	0.0							0.0	0.0	1766294.2	
MA0975000	PA BOQUEIRÃO I / COLONE	370.7	62232.9	0.0	0.0	6.0	1003.8					6.0	1003.8	63236.6	
MA0976000	PA QUADRA PIMENTEIRA / COLONE	1547.8	276039.5	0.0	0.0							0.0	0.0	276039.5	
MA0977000	PA QUADRA 10 DE JANEIRO / COLONE	1261.4	233442.7	0.0	0.0							0.0	0.0	233442.7	

MA0978000	PA QUADRA NOVA PIMENTEIRA / COLONE	1249.6	229568.1	0.0	0.0							0.0	0.0	229568.1	
MA0979000	PA DIBOM I	3095.2	519655.7	0.0	0.0	11.5	1927.9	56.5	9484.1			68.0	11412.0	531067.7	287.5
MA0980000	PA DIBOM II	1562.2	262282.3	0.0	0.0	39.6	6649.0					39.6	6649.0	268931.3	
MA0981000	PA BOM JESUS III	359.4	54757.9	0.0	0.0	1564.5	191719.5			141.8	17567.2	1706.3	209286.7	264044.6	12.8
MA0982000	PA BOCA DA MATA I	2065.0	306197.2	0.0	0.0	336.5	56498.4	450.5	6962.7			787.0	63461.1	369658.3	306.2
MA0994000	PE TATAJUBAL	373.8	63381.8	0.0	0.0	9.8	1692.1					9.8	1692.1	65073.9	
MA0997000	PA SOBRADINHO	2249.1	342901.8	736.4	88076.2	10734.0	1642379.7	2023.8	270379.6			12757.7	1912759.3	2343737.3	
MA0998000	PA SÃO BARTOLOMEU / CIAMA	2752.8	509430.9	0.0	0.0	482.4	89277.3					482.4	89277.3	598708.1	
MA0999000	PA TORRÃO MUQUEM	489.3	82144.1	0.0	0.0							0.0	0.0	82144.1	
MA1002000	PA FÊNIX	7129.9	1318724.4	0.0	0.0	2153.4	400858.2			65.0	11993.2	2218.5	412851.4	1731575.9	
MA1003000	PA TERRA LIVRE	1244.3	232551.7	0.0	0.0	2018.6	377260.5			59.8	11173.3	2078.4	388433.7	620985.4	
MA1005000	PAE AGROEXTRATIVISTA BACURI	0.0	0.0	127.8	4217.5			5682.0	187449.3			5682.0	187449.3	191666.8	
MA1017000	PA MUTUM	0.0	0.0	0.0	0.0			67.3	10345.4			67.3	10345.4	10345.4	
MA1025000	PA CATINGUEIRO	669.6	112416.6	0.0	0.0							0.0	0.0	112416.6	
MA1030000	PA SÃO FRANCISCO/BOA VIAGEM	935.8	173187.2	0.0	0.0	286.5	53025.5					286.5	53025.5	226212.8	
MA1031000	PA RENASCER	146.4	24598.5	0.0	0.0	429.1	72043.4			37.7	6338.3	466.8	78381.7	102980.3	0.2
MA1032000	PA HORIZONTE AZUL	357.6	66832.4	0.0	0.0	1566.1	292684.9			16.8	3142.1	1582.9	295827.0	362659.3	
MA1033000	PA SÃO RAIMUNDO	1135.8	170161.6	163.3	24464.2	282.5	42327.5	977.2	146401.3			1259.8	188728.8	383354.6	
MA1042000	PA CRISTALINA/COLONE	229.5	42467.7	0.0	0.0							0.0	0.0	42467.7	
MA1043000	PA VILA SANTA MARIA/PIRATININGA	312.0	52375.5	0.0	0.0							0.0	0.0	52375.5	
MA1045000	PA LAGOA DA FLORESTA	1144.1	171399.3	191.7	28712.2	926.0	138725.1	38.5	5762.4			964.5	144487.5	344599.0	
MA1051000	PA OZIEL ALVES PEREIRA I	889.0	133181.5	0.0	3.6			0.5	77.0			0.5	77.0	133262.1	
MA1055000	PA VALE DO TAPUIO	0.0	0.0	1170.8	38623.7			8237.7	271762.4			8237.7	271762.4	310386.1	
MA1056000	PAE AGROEXTRATIVISTA BACURI I	181.9	21810.7	0.0	0.0	367.3	54948.2	278.6	10158.3			645.9	65106.4	86917.1	440.6

MA1057000	PAE AGROEXTRATIVISTA SANTANA RAPOSO III	398.4	66888.6	0.0	0.0	133.7	22454.6	211.8	9684.5	9.6	1606.5	355.1	33745.6	100634.2	93.0
MA1058000	PA NUCLEO BB	3306.3	611865.1	0.0	0.0							0.0	0.0	611865.1	
MA1060000	PA BREJO DO JOÃO	315.3	46626.1	42.6	4995.0	365.1	50224.9	2188.8	254282.7			2554.0	304507.6	356128.8	
MA1061000	PDS PETROLINA	605.9	101725.2	0.0	0.0							0.0	0.0	101725.2	
MA1062000	PDS CITEMA	9353.7	1745061.3	0.0	0.0	9088.7	1698265.0			4626.8	864480.9	13715.5	2562746.0	4307807.3	4.8
MA1063000	PAE VILA ADRIANA	504.1	92280.0	0.0	0.0							0.0	0.0	92280.0	
MA1064000	PDS TEMASA	1006.7	188146.2	0.0	0.0	3482.3	650811.6	1950.4	364437.2	1185.7	221589.6	6618.4	1236838.4	1424984.6	19.9
MA1065000	PA BACURIZINHO	400.3	67200.2	0.0	0.0							0.0	0.0	67200.2	
MA1066000	PDS SANTA BÁRBARA	5574.1	935829.8	0.0	0.0	0.0	4.3					0.0	4.3	935834.1	
MA1067000	PA SAO FELIX I	0.0	0.0	0.0	0.0			739.1	108514.0			739.1	108514.0	108514.0	
MA1068000	PA NOVA CANAA	0.0	0.0	123.5	18132.9			1456.7	213860.8			1456.7	213860.8	231993.7	
MA1069000	PA FRANCISCO ROMAO	3193.4	590977.4	0.0	0.0	961.9	178001.5					961.9	178001.5	768978.9	
MA1070000	PDS JOAO DO VALE I	1210.7	222370.0	0.0	0.0	7.5	1384.7					7.5	1384.7	223754.7	
MA1071000	PAE SANTA MARIA	544.9	91476.8	0.0	0.0							0.0	0.0	91476.8	
	Total in Maranhão	2065499.8	348825941.4	106208.1	12258927.3	248529.0	41586824.6	248694.3	17984500.8	179692.0	28376775.0	676915.2	87948100.4	449032969.1	17890.1
**MATO GROSSO**			0.0	0.0	0.0							0.0	0.0	0.0	
MT0011000	PAC PEIXOTO DE AZEVEDO	50198.0	8621720.1	0.0	0.0	8535.1	1462843.1					8535.1	1462843.1	10084563.2	
MT0012000	PAC CARLINDA	32411.2	5447537.4	0.0	0.0	1972.1	335496.4					1972.1	335496.4	5783033.8	8.0
MT0013000	PA BRAÇO SUL	125632.9	20694051.8	0.0	0.0	29673.6	4701459.3	329.4	51327.5	112.9	17679.8	30115.8	4770466.6	25464518.4	33.9
MT0014000	PA CAPÃO VERDE	3167.8	464715.6	0.0	0.0	122.3	17022.9	2495.9	181288.9			2618.3	198311.7	663027.3	
MT0015000	PA TUPÃ	2638.4	346990.4	0.0	0.0	180.5	26492.9	35.0	4924.3			215.5	31417.2	378407.6	
MT0016000	PA MORRINHO	0.0	0.0	2023.5	66754.7			110.7	3653.0			110.7	3653.0	70407.7	0.1
MT0017000	PA SARARÉ	13611.6	2030387.4	0.0	0.0	901.6	132639.8	3597.4	186523.7			4498.9	319163.5	2349550.9	
MT0018000	PA 1.500 ALQUEIRES	3259.9	486020.9	0.0	0.0	117.9	16287.6					117.9	16287.6	502308.5	
MT0019000	PA NOVA ALVORADA	3726.3	545848.3	0.0	0.0	458.5	66846.3					458.5	66846.3	612694.6	
MT0020000	PA PAPIRO	160.4	5291.9	0.0	0.0	0.7	23.8	1089.2	35932.3			1089.9	35956.1	41248.0	

MT0021000	PA NOVO MÉXICO	8600.5	1444518.3	0.0	0.0	1329.0	226622.2			1329.0	226622.2	1671140.5	
MT0022000	PA CORONEL ARI	7291.8	993476.1	0.0	0.0	259.5	35577.2	764.2	29398.5	1023.7	64975.6	1058451.7	
MT0023000	PA CANA BRAVA	20555.0	2706278.0	4924.2	240924.5	427.3	19934.3	9333.0	502488.2	9760.2	522422.4	3469624.9	25.1
MT0025000	PA HIJ	26080.4	4181102.2	0.0	0.0	3351.6	516727.1			3351.6	516727.1	4697829.3	
MT0026000	PA ETA	21895.9	3667229.2	0.0	0.0	1391.6	230250.7			1391.6	230250.7	3897479.8	57.6
MT0027000	PA TIRA SENTIDO	4768.2	547397.1	0.0	0.0	31.7	4482.5	1516.9	50041.9	1548.6	54524.4	601921.5	
MT0028000	PA JATOBAZINHO	0.0	0.0	13389.8	232601.1			1746.9	33229.4	1746.9	33229.4	265830.4	
MT0029000	PA FORMOSA	6608.3	928226.9	0.0	0.0	1358.4	185564.1	6649.2	253531.1	8007.6	439095.2	1367322.1	
MT0030000	PA GATO PRETO	0.0	0.0	4319.7	316611.0			3189.8	174735.6	3189.8	174735.6	491346.6	
MT0031000	PA SAFRA	0.0	0.0	20113.9	663140.7			9275.3	394350.9	9275.3	394350.9	1057491.6	
MT0033000	PA RIO DOS COCOS	3499.8	468576.2	0.0	0.0	1469.7	187766.8	1.4	211.5	1471.2	187978.3	656554.5	
MT0035000	PA CRUZEIRO DO NORTE	1676.9	235899.0	440.2	34571.6	122.3	18313.4	246.6	22170.4	368.9	40483.8	310954.4	
MT0037000	PA MARAGATOS	0.0	0.0	1695.0	49424.4			1032.9	34814.8	1032.9	34814.8	84239.2	2.8
MT0039000	PA ILHA DO COCO	0.0	0.0	898.8	28473.1			627.7	20707.5	627.7	20707.5	49180.6	
MT0040000	PA RIBERÃO DOS COCAIS	2062.9	76101.4	142.6	4743.6	38.8	3540.6	105.3	3474.6	144.1	7015.2	87860.2	
MT0041000	PA MELLO	3147.3	272678.6	0.0	0.0					0.0	0.0	272678.6	
MT0042000	PA MONTECHI	56.4	1860.6	568.8	18764.3			288.6	9521.6	288.6	9521.6	30146.5	
MT0043000	PA VINAGRE LOTE 31	2042.8	342966.1	0.0	0.0					0.0	0.0	342966.1	
MT0044000	PA CRUZEIRO DO SUL	14172.6	1990885.9	0.0	0.0	9198.1	723100.5	7605.8	706424.7	16803.9	1429525.3	3420411.1	23.5
MT0045000	PA LIBERDADE	2622.8	377291.2	0.0	0.0					0.0	0.0	377291.2	
MT0046000	PA SERRA NOVA I	17804.4	2649223.5	0.0	0.0	27.4	4078.8	2596.7	381638.1	2624.1	385716.9	3034940.5	
MT0047000	PA MIRASSOLZINHO	10978.7	1592784.5	141.7	18353.4	4669.3	656687.1	23374.5	758348.0	28043.8	1415035.1	3026173.1	285.6
MT0048000	PA PRESIDENTE	4071.3	283226.1	3375.0	111348.0			212.7	7107.6	212.7	7107.6	401681.7	46.6
MT0049000	PA RIO VERMELHO	0.0	0.0	1007.1	88479.1			1057.7	73388.7	1057.7	73388.7	161867.8	
MT0050000	PA SERRAGEM	20520.5	2569952.4	0.0	0.0	4673.0	419182.9			4673.0	419182.9	2989135.3	1.7
MT0051000	PA JACARÉ VALENTE	23769.3	3078711.7	0.0	0.0	2475.6	264863.2	5332.4	515531.2	7808.0	780394.5	3859106.1	
MT0052000	PA CANTA GALO	15640.0	663058.6	11585.3	455420.1	376.8	12431.0	21474.6	727988.5	21851.4	740419.5	1858898.3	

MT0053000	PA COQUEIRAL/QUEBÓ	6320.6	853416.5	0.0	0.0	531.6	60283.3	4809.3	204346.3	5340.9	264629.7	1118046.1	
MT0054000	PA NOROAGRO	0.0	0.0	1120.5	45993.2			416.7	22345.4	416.7	22345.4	68338.7	80.4
MT0055000	PA VOLTA GRANDE	11132.8	1035437.9	0.0	0.0	6740.6	568957.2	1673.0	127094.4	8413.6	696051.7	1731489.5	
MT0056000	PA MIRANDA ESTÂNCIA	15084.6	2675853.1	0.0	0.0	2669.8	487186.6			2669.8	487186.6	3163039.7	
MT0057000	PA PERSEVERANÇA PACUTINGA	71487.4	10951784.6	88.2	4208.8	33716.8	5191756.2	9219.2	215792.7	42936.0	5407548.8	16363542.2	18.9
MT0058000	PA TIBAGI	8412.6	1254310.9	24049.5	3544320.6	1165.8	176875.4	576.4	86891.8	1742.2	263767.2	5062398.7	
MT0060000	PA ELDORADO I	0.0	0.0	3319.4	86241.0			4126.7	106178.6	4126.7	106178.6	192419.6	66.1
MT0061000	PA PIAU	2813.0	421218.6	0.0	0.0					0.0	0.0	421218.6	
MT0062000	PA TRIANGULO	0.0	0.0	5296.0	143287.4			4119.2	122706.7	4119.2	122706.7	265994.0	
MT0063000	PA SERRINHA	11978.4	1821297.1	0.0	0.0	3570.5	543622.8	120.1	16425.9	3690.6	560048.7	2381345.8	
MT0064000	PA COUTINHO UNIÃO	7870.2	658724.8	2.0	64.7	32.8	3596.2	16.1	1468.2	48.9	5064.4	663853.9	
MT0065000	PA FICA FACA	1227.9	78970.2	7751.9	208595.3	106.2	14517.4	452.3	32010.6	558.5	46528.0	334093.5	14.0
MT0066000	PA PONTAL	9500.6	1180611.3	4.0	243.5	1633.3	85922.4	11271.5	994341.9	12904.8	1080264.2	2261119.0	
MT0067000	PA PIRACICABA	315.5	17949.3	5721.1	194109.3	281.0	18775.6	4380.8	153340.8	4661.8	172116.4	384175.0	
MT0068000	PA AZULONA GAMELEIRA	15817.0	1588802.0	5113.1	389168.1	16535.4	1854766.5	68779.9	2340824.5	85315.3	4195591.1	6173561.2	761.4
MT0069000	PA SANTO ANTONIO DA MATA AZUL	0.0	0.0	2551.7	49012.9	0.0	1.6	1271.8	71871.2	1271.8	71872.8	120885.7	
MT0070000	PA MARTINS I	3468.7	354244.1	0.0	0.0	270.2	33987.1	423.9	28610.6	694.2	62597.8	416841.9	
MT0071000	PA GRANJA	0.0	0.0	10221.8	337217.9			629.6	20768.9	629.6	20768.9	357986.8	2.9
MT0072000	PA BANDEIRANTES	5496.0	702574.9	0.0	0.0	914.4	77253.8	2172.5	320069.9	3087.0	397323.7	1099898.7	
MT0073000	PA XAVANTE FIGURA A	21.2	1541.0	7919.4	158853.9	80.9	6196.8	25098.8	669628.1	25179.7	675824.9	836219.7	
MT0074000	PA CHAPADINHA	21331.6	2481148.8	2392.4	73765.2	3089.6	271337.8	7011.0	202763.6	10100.6	474101.5	3029015.4	7.4
MT0075000	PA FARTURA	3628.8	119569.3	6079.5	157066.2	1316.4	44970.6	4582.8	148058.5	5899.2	193029.0	469664.6	
MT0076000	PA BOJUI	62950.9	11247683.0	0.0	0.0	36968.9	6659930.8	4.3	703.8	36973.2	6660634.6	17908317.7	
MT0077000	PA NOVA COTRIGUAÇU	1231.9	203912.9	0.0	0.0	27.8	4540.8			27.8	4540.8	208453.7	
MT0078000	PA NOVO HORIZONTE	5998.6	1010161.1	0.0	0.0	626.6	108864.4			626.6	108864.4	1119025.5	
MT0079000	PA AVAÍ	0.0	0.0	5373.7	239549.8			7360.9	377352.6	7360.9	377352.6	616902.3	
MT0080000	PA RONCADOR	4217.4	254756.2	1081.4	83972.2	533.5	17000.0	2852.1	194376.1	3385.6	211376.1	550104.6	

MT0081000	PA MANAH	56789.2	6056574.0	12399.8	822378.1	8496.7	908547.1	47171.2	1992713.3	55667.9	2901260.4	9780212.5	
MT0082000	PA MACIFE	5661.3	803056.2	1185.7	58163.2	395.9	57901.7	3321.5	220363.0	3717.4	278264.8	1139484.2	
MT0083000	PA SERRA NOVA II	0.0	0.0	5262.8	172561.3			2975.0	105073.8	2975.0	105073.8	277635.1	
MT0084000	PA RANCHO AMIGO	399.4	23134.0	5537.8	125662.8	231.0	15215.1	17924.4	436991.2	18155.3	452206.3	601003.1	
MT0085000	PA CARNAÚBA	1329.2	224059.6	0.0	0.0	297.7	51096.6			297.7	51096.6	275156.2	
MT0086000	PA CRISTO REI	4055.0	678921.4	0.0	0.0	292.2	47966.8			292.2	47966.8	726888.2	
MT0087000	PA UNIÃO DE TODOS	8602.8	1485232.1	0.0	0.0	3810.5	702405.6	34.7	5334.8	3845.2	707740.3	2192972.4	
MT0088000	PA COTREL	0.0	0.0	164.6	5429.8			700.6	23112.5	700.6	23112.5	28542.3	
MT0089000	PA PIRAPUTANGA	0.0	0.0	709.0	23389.1			3322.3	109603.3	3322.3	109603.3	132992.4	
MT0090000	PA PRAIA RICA	0.0	0.0	356.4	38167.9			523.2	55998.7	523.2	55998.7	94166.6	
MT0101000	PA VÃO GRANDE	84.7	11637.3	291.4	15937.3	1427.0	196147.2	421.5	55561.1	1848.5	251708.4	279283.0	147.8
MT0102000	PA OLARIA	1563.7	234265.2	0.0	0.0	12.1	1805.4			12.1	1805.4	236070.6	
MT0103000	PA CÓRREGO DA ONÇA	8533.9	1039835.5	4.3	634.3	157.2	22695.1			157.2	22695.1	1063164.8	
MT0104000	PA CORGÃO	1149.0	168688.0	0.0	0.0	5.7	834.4	526.6	77309.0	532.3	78143.4	246831.5	
MT0105000	PA MIRASSOLZINHO II	3616.6	532614.2	164.9	16306.5	797.9	120165.2	884.8	36668.8	1682.6	156834.0	705754.7	
MT0106000	PA COLONIA DOS MINEIROS	0.0	0.0	2818.5	92981.4			4619.7	151786.7	4619.7	151786.7	244768.1	
MT0107000	PA SÃO LUIZ	3314.3	399412.1	0.0	0.0	523.8	67531.5	2.2	313.5	526.0	67845.0	467257.1	
MT0108000	PA PROVIDÊNCIA - I	1466.5	14666.6	0.0	0.0			62.4	588.0	62.4	588.0	15254.6	
MT0109000	PA PROVIDÊNCIA III	9949.9	1478057.8	0.0	0.0	1761.2	247121.8	2875.0	248938.2	4636.2	496060.0	1974117.8	
MT0110000	PA NOVA CONQUISTA	11264.1	1695705.0	0.0	0.0	513.2	77388.9			513.2	77388.9	1773093.9	
MT0111000	PA BONJAGUÁ	643.9	108112.5	0.0	0.0					0.0	0.0	108112.5	
MT0112000	PA SANTA IZABEL	71669.8	8105466.6	2.7	210.9	11931.5	1062951.9	12237.4	1849252.8	24168.9	2912204.7	11017882.3	
MT0113000	PA CONFRESA/RONCADOR	1455.4	244523.7	0.0	0.0	201.4	34316.7			201.4	34316.7	278840.4	
MT0114000	PA SERRA NEGRA	3627.6	452102.8	0.0	0.0	1960.7	295253.0			1960.7	295253.0	747355.8	
MT0115000	PA CABIXI	4065.8	683373.3	0.0	0.0	357.1	62077.2			357.1	62077.2	745450.5	0.3
MT0116000	PA OURO BRANCO	2905.6	488130.5	0.0	0.0	67.0	11461.9			67.0	11461.9	499592.5	3.9
MT0117000	PA MONTE DAS OLIVEIRAS	4609.2	745548.9	0.0	0.0	660.4	103075.2	1632.1	252827.4	2292.5	355902.6	1101451.5	113.8

MT0118000	PA BELA VISTA	17692.2	2322189.4	0.0	0.0	2670.7	284928.3	1229.8	42127.2			3900.4	327055.5	2649244.9	5.8
MT0119000	PA MARIA TEREZA	3724.7	403559.8	0.0	0.0	459.0	36473.7					459.0	36473.7	440033.5	
MT0120000	PA PORTO ESPERANÇA	5992.3	835039.5	0.0	0.0	432.0	39935.4	2422.9	386614.2			2854.9	426549.6	1261589.1	
MT0121000	PA INDEPENDENTE II	19537.1	2401441.4	42.7	1463.9	73.4	2422.3					73.4	2422.3	2405327.6	
MT0122000	PA MACIFE II	10467.1	1687866.7	0.0	0.0	2829.3	441773.0	46.4	7106.8			2875.7	448879.9	2136746.5	
MT0123000	PA CACHOEIRA DA UNIÃO	3470.7	490755.6	0.0	0.0	16.5	2093.8					16.5	2093.8	492849.3	
MT0124000	PA VEREDA	7816.8	1178977.6	0.0	0.0	136.2	20734.2					136.2	20734.2	1199711.8	
MT0125000	PA YAMIN	6416.1	1076639.9	0.0	0.0	657.3	110010.8	75.5	12683.3			732.9	122694.1	1199334.0	
MT0126000	PA SÃO JOSÉ	93608.3	14246211.3	0.0	0.0	21913.8	3355711.1			313.7	47929.2	22227.5	3403640.3	17649851.6	126.6
MT0127000	PA TAPURAH/ITANHANGA	2205.9	354640.9	0.0	0.0	762.3	118356.9					762.3	118356.9	472997.8	
MT0128000	PA SÃO CRISTÓVÃO	1503.1	249948.0	0.0	0.0	29.2	4910.4	46.1	7059.1			75.4	11969.4	261917.5	
MT0129000	PA CASTANHAL	2617.2	431111.5	0.0	0.0	401.1	65417.0	260.2	42397.9			661.3	107815.0	538926.5	
MT0130000	PA ALVORADA	13308.7	1609284.2	0.0	0.0	2166.3	202917.9	800.7	67253.5			2967.0	270171.4	1879455.5	
MT0131000	PA INDEPENDENTE I	38107.4	5725956.4	0.0	0.0	13801.1	2106063.8	171.5	26138.3	56.7	8679.8	14029.3	2140881.9	7866838.3	
MT0132000	PA CACHIMBO	0.0	0.0	871.3	40307.1			2667.6	111338.7			2667.6	111338.7	151645.8	3220.3
MT0133000	PA QUILOMBO	2389.5	399785.5	0.0	0.0	298.2	49517.2					298.2	49517.2	449302.7	8.5
MT0134000	PA TAPAYUNA	4729.1	678398.4	0.0	0.0	258.6	37402.5	1113.7	58080.1			1372.3	95482.6	773881.0	
MT0136000	PA MACUCO	4137.5	577705.5	0.0	0.0	2089.9	287907.7	10464.8	827929.6			12554.7	1115837.3	1693542.8	
MT0137000	PA SANTA HELENA	11341.6	1691391.6	0.0	0.0	1344.8	193594.2	123.4	18100.5			1468.2	211694.6	1903086.2	
MT0138000	PA RIO ALEGRE	3731.5	119878.0	15639.1	471506.4	177.8	5509.1	10020.8	322268.8			10198.6	327777.9	919162.3	
MT0139000	PA PIRATININGA	13669.5	1991536.1	0.0	0.0	9664.3	1530316.3	6307.4	990502.9	17.5	3277.1	15989.3	2524096.4	4515632.5	
MT0140000	PA HORIZONTE II	0.0	0.0	5320.0	184180.1			11626.0	403036.9			11626.0	403036.9	587217.0	138.8
MT0141000	PA FORQUILHA DO RIO MANSO	3827.4	640874.0	0.0	0.0	623.1	101768.0					623.1	101768.0	742642.0	
MT0142000	PA COLONIA BOM JESUS	6545.5	1054699.9	12.4	2025.9	101.4	16276.3					101.4	16276.3	1073002.1	
MT0143000	PA ITAPORÃ DO NORTE	5575.1	851380.9	0.0	0.0	1153.0	176485.2					1153.0	176485.2	1027866.1	
MT0145000	PA IRACEMA	3693.6	519611.3	66.3	9329.9	77.6	10916.4	0.6	79.6			78.2	10996.0	539937.3	
MT0146000	PA GERALDO PEREIRA DE	0.0	0.0	0.0	0.0			362.1	52435.9			362.1	52435.9	52435.9	

	ANDRADE (MARIA IZABEL)												
MT0147000	PA MORRINHO DO TARUMÃ	610.1	20127.1	1.0	32.9	6.6	218.6			6.6	218.6	20378.6	
MT0148000	PA VALE DO BACURI	1223.3	179599.9	0.0	0.0					0.0	0.0	179599.9	
MT0149000	PA PARAISO	6234.1	1046644.7	0.0	0.0	1388.9	233182.0	633.3	106331.2	2022.2	339513.2	1386157.9	
MT0150000	PA RONDON	0.0	0.0	744.5	24559.9			111.5	3677.5	111.5	3677.5	28237.4	
MT0151000	PA SANTA LUZIA	0.0	0.0	15082.3	1643855.7			3623.8	343516.8	3623.8	343516.8	1987372.5	
MT0152000	PA SANTO IDELFONSO	1696.5	249056.4	8.2	1204.5	58.4	8577.7			58.4	8577.7	258838.6	
MT0153000	PA TERRA FORTE	11.2	819.3	1213.5	68741.2	1.2	93.9	31.0	2289.5	32.2	2383.4	71943.9	
MT0154000	PA PRIMAVERA	0.0	0.0	1369.4	66951.2			4.4	213.2	4.4	213.2	67164.4	
MT0155000	PA SÃO FRANCISCO	0.0	0.0	2131.9	74089.0			241.5	26149.7	241.5	26149.7	100238.7	
MT0156000	PA 28 DE OUTUBRO	1387.2	231966.0	0.0	0.0	254.5	41835.3			254.5	41835.3	273801.3	
MT0157000	PA ALTO PARAISO	2164.5	141356.0	2894.4	124039.7	801.1	66104.6	145.5	11128.8	946.6	77233.5	342629.2	
MT0158000	PA CARIMÃ	2106.1	313423.8	0.0	0.0	44.7	6251.4			44.7	6251.4	319675.2	
MT0159000	PA ARACATY	16654.5	1187463.2	1526.0	82288.2	6232.8	454311.8	5896.5	190107.1	12129.3	644418.9	1914170.3	
MT0160000	PA DOM PEDRO	744.7	101094.1	0.0	0.0	1419.3	209122.8	8634.6	500180.9	10053.9	709303.7	810397.8	
MT0162000	PA LARANJEIRA I	0.0	0.0	0.0	0.0			1193.6	39377.6	1193.6	39377.6	39377.6	
MT0163000	PA LARANJEIRA II	7237.4	1084198.3	0.0	0.0	120.3	18014.7	0.7	107.8	121.0	18122.5	1102320.8	
MT0164000	PA AERORANCHO	3683.0	107350.7	0.0	0.0	88.2	2854.8			88.2	2854.8	110205.5	
MT0165000	PA MARGARIDA ALVES	38.2	2400.4	371.6	12258.5	95.8	8900.1	15399.3	501710.0	15495.1	510610.1	525269.0	33.0
MT0166000	PA PAIOL	45126.3	6931042.8	0.0	0.0	10274.4	1615001.0	7888.7	1199980.7	18163.1	2814981.7	9746024.5	
MT0167000	PA MERCEDES BENS I E II	1142.9	166802.5	0.0	0.0	84.8	11788.7			84.8	11788.7	178591.2	
MT0168000	PA SANTA HELENA II	0.0	0.0	9691.7	456792.3			4802.3	296270.7	4802.3	296270.7	753063.1	
MT0169000	PA CANCELA	0.0	0.0	7084.6	258475.3			14298.1	610045.0	14298.1	610045.0	868520.3	
MT0170000	PA NOIDORINHO/VITÓRIA	16905.6	2234858.6	0.0	0.0	1242.3	111397.2	2206.6	323553.8	3449.0	434951.0	2669809.6	
MT0172000	PA MARGARIDA UNIÃO	116.8	16256.7	0.0	0.0	15.9	2222.7	167.1	3684.1	183.0	5906.8	22163.5	
MT0173000	PA BOCAINA	31322.0	4737180.5	0.0	0.0	14906.5	2271065.1	506.4	77079.4	15413.0	2348144.5	7085325.0	
MT0174000	PA SÃO JOSÉ UNIÃO	181.9	23599.2	0.0	0.0	128.7	17925.4	344.0	23825.7	472.7	41751.1	65350.3	

MT0176000	PA SÃO SEBASTIÃO	0.9	28.5	11163.3	376042.6	16.8	553.0	2426.3	87466.0			2443.0	88019.0	464090.2	
MT0179000	PA SANTA MARIA	11776.3	1565019.1	0.0	0.0	782.6	72966.7	384.1	16755.0			1166.7	89721.7	1654740.9	
MT0180000	PA TATUIBY	18297.7	2517826.9	0.0	0.0	4519.0	663762.0	15337.7	630083.3			19856.6	1293845.2	3811672.2	246.4
MT0181000	PA ANTONIO CONSELHEIRO	784.0	41938.2	0.0	0.0	321.0	24595.0	578.5	44593.4			899.5	69188.5	111126.7	
MT0183000	PA NOVA ESPERANÇA	0.0	0.0	848.2	19466.4	7.5	579.4	468.4	13268.5			475.9	13847.9	33314.3	13.7
MT0184000	PA LAGO DE PEDRA	615.0	86512.8	1533.8	215774.2			34.7	4882.5			34.7	4882.5	307169.5	
MT0185000	PA MÁRCIO PEREIRA	0.0	0.0	282.0	15551.3			717.9	29968.9			717.9	29968.9	45520.2	
MT0186000	PA FURNAS DO BURITI	6016.5	902276.1	0.0	0.0	540.4	81066.0					540.4	81066.0	983342.1	
MT0187000	PA SERINGAL	5367.0	820107.5	0.0	0.0	2102.7	321896.3					2102.7	321896.3	1142003.8	
MT0188000	PA RIO BORGES	2661.5	402962.0	0.0	0.0	730.5	111810.4					730.5	111810.4	514772.5	
MT0189000	PA BONANZA	2836.7	434851.4	38.3	5850.8	238.2	37619.4					238.2	37619.4	478321.6	
MT0190000	PA SANTA IRENE	9241.2	301398.8	1015.6	30012.3	3061.7	163160.4	180.1	5934.4	19.9	656.4	3261.7	169751.2	501162.3	
MT0191000	PA CAMPINAS	20497.2	3134463.7	0.0	0.0	10642.6	1629025.7	34.5	5284.4			10677.1	1634310.1	4768773.8	
MT0192000	PA PADOVANI	6354.9	618125.7	34.6	4669.3	47.1	5912.8					47.1	5912.8	628707.8	
MT0193000	PA SANTA ROSA	8233.2	1252777.4	0.0	0.0	934.1	132105.3					934.1	132105.3	1384882.8	
MT0194000	PA BOA VISTA	1136.6	170098.0	0.0	0.0	81.8	12516.1					81.8	12516.1	182614.1	
MT0195000	PA SÃO BENEDITO	1797.3	263863.4	0.0	0.0							0.0	0.0	263863.4	
MT0196000	PA SANTA ROSA I	324.5	25062.3	0.0	0.0	235.6	18199.9	349.2	26973.2			584.8	45173.0	70235.3	
MT0197000	PA JATOBÁ	100.7	7294.4	0.0	0.0	35.1	2626.2	2235.2	104342.7			2270.4	106968.9	114263.3	
MT0198000	PA RANCHO DA SAUDADE	18467.5	3284703.9	0.0	0.0	11955.8	2063936.1	325.5	53320.7			12281.3	2117256.8	5401960.7	48.9
MT0199000	PA JURUENA	13447.3	1930582.0	0.0	0.0	14563.8	1967572.8	2005.1	33587.1			16568.9	2001159.9	3931741.9	25.3
MT0200000	PA ENA	6842.0	1046689.1	0.0	0.0	681.5	104210.7					681.5	104210.7	1150899.7	
MT0201000	PA CALIFORNIA	13920.7	2318881.8	0.0	0.0	113.9	18605.0					113.9	18605.0	2337486.8	
MT0202000	PA VERANEIO	26869.5	4980052.9	0.0	0.0	7984.6	1491665.8	13.3	2491.3			7998.0	1494157.1	6474210.0	
MT0203000	PA SÃO PEDRO	822.2	125313.4	0.0	0.0	45.8	6979.3	9781.5	1269180.6			9827.3	1276159.9	1401473.4	
MT0204000	PE NOVA FLORESTA	5477.2	816469.4	0.0	0.0	1126.1	163916.1	231.8	34761.0			1357.9	198677.1	1015146.6	
MT0207000	PA RITINHA	2359.6	354009.8	42.2	6411.5	178.9	27351.6					178.9	27351.6	387773.0	
MT0209000	PA MOGIANA I	2528.7	385948.4	45.4	6948.5	473.1	72424.8					473.1	72424.8	465321.8	

MT0210000	PA FURNAS III	93.4	11371.0	18519.4	516085.6	237.5	26199.0	1352.5	103762.4	1590.0	129961.4	657418.0	2.8
MT0212000	PA JARAGUÁ	17119.4	2362365.5	0.0	0.0	4837.6	594685.2	6470.6	210701.2	11308.2	805386.4	3167751.9	87.6
MT0213000	PA BRASIL NOVO	4045.4	472009.8	0.0	0.0	2108.4	292370.5	1097.4	168000.5	3205.7	460370.9	932380.8	
MT0217000	PA IRIRIZINHO	0.0	0.0	587.3	18362.3			379.6	12361.4	379.6	12361.4	30723.7	
MT0219000	PA CAMPO LIMPO	0.0	0.0	2679.6	88398.6			2046.0	67482.6	2046.0	67482.6	155881.2	
MT0221000	PA SADIA III	1271.6	189553.6	0.0	0.0	470.7	69825.6	716.8	41533.6	1187.5	111359.2	300912.7	
MT0223000	PA RIOZINHO	1223.8	160404.3	1.1	145.9					0.0	0.0	160550.2	
MT0224000	PA COQUEIRO	0.0	0.0	1423.3	87524.1			51.6	6960.4	51.6	6960.4	94484.5	
MT0225000	PA DOIS IRMÃOS	0.0	0.0	0.0	0.0			2353.0	91783.9	2353.0	91783.9	91783.9	
MT0227000	PA VALE DO SOL	0.0	0.0	2636.0	160402.5			481.9	37364.6	481.9	37364.6	197767.1	
MT0228000	PA PADRE JOSIMO TAVARES	0.0	0.0	1957.3	64572.5			302.6	9984.1	302.6	9984.1	74556.5	8.9
MT0230000	PA ESTRELA DO ORIENTE	0.0	0.0	3701.8	121261.6			1352.3	40276.8	1352.3	40276.8	161538.4	
MT0231000	PA SANTO ANTONIO	3382.1	450302.0	0.0	0.0	4005.9	451846.4	2350.6	36924.4	6356.5	488770.8	939072.8	86.7
MT0232000	PA SANTA TEREZINHA II	21022.9	3030090.2	0.0	0.0	11030.4	1349182.2			11030.4	1349182.2	4379272.4	
MT0233000	PA BOA ESPERANÇA I, II E III	28332.5	4279169.4	0.0	0.0	8973.4	1351215.6	1507.5	94952.6	10480.8	1446168.1	5725337.5	
MT0234000	PA PINGOS DÁGUA	0.0	0.0	4141.1	136615.4			1191.7	39315.1	1191.7	39315.1	175930.5	
MT0235000	PA NOSSA SENHORA DO CARMO	12800.1	1973657.2	304.0	49837.3	950.8	154671.3	62.8	10230.2	1013.5	164901.4	2188396.0	
MT0236000	PA SÃO JOSÉ DA VILA RICA	10413.5	1710674.4	0.0	0.0	1359.1	221552.0			1359.1	221552.0	1932226.4	
MT0237000	PA IPE	30598.6	3377344.3	0.0	0.0	10483.3	890918.9	513.3	39647.4	10996.6	930566.3	4307910.5	
MT0239000	PA SANTO ANTONIO DO FONTOURA I	9684.1	588409.5	0.0	0.0	8687.6	573594.9	626.3	46590.4	9313.9	620185.3	1208594.8	343.6
MT0240000	PA SANTA CLARA	5273.1	470712.3	0.0	0.0	2926.3	216959.4	836.2	67963.8	3762.4	284923.2	755635.5	82.0
MT0241000	PA BRASIPAIVA I E II	19400.2	2921117.0	4527.9	655775.7	10.0	1517.5	1176.3	161314.7	1186.2	162832.1	3739724.8	11.0
MT0242000	PA MÃE MARIA	27114.3	4437477.7	0.0	0.0	8005.8	1313579.2	31.6	5148.2	8037.4	1318727.4	5756205.1	3.3
MT0243000	PA VALE DO SERINGAL	6668.4	1245746.6	0.0	0.0	8048.5	1504175.8			8048.5	1504175.8	2749922.4	1.2
MT0244000	PA VALE DO AMANHECER	2556.8	363871.3	374.6	12433.6	365.9	55774.0			365.9	55774.0	432078.9	
MT0247000	PA SANTA LUZIA I	3153.7	478490.8	14.0	2117.6	557.4	85326.9			557.4	85326.9	565935.3	
MT0248000	PA CRISTALMEL	11.0	362.8	2488.3	43152.5	272.8	8999.3	372.4	11921.0	645.2	20920.3	64435.6	

MT0249000	PA GUAPIRAMA	3449.8	527192.3	0.0	0.0	621.2	95099.9			621.2	95099.9	622292.2	
MT0250000	PA BOGORNI	1993.6	212423.2	0.0	0.0	305.2	25667.3			305.2	25667.3	238090.4	
MT0251000	PA SAO FRANCISCO II	1359.9	206701.4	11.9	1823.3	100.8	15424.8			100.8	15424.8	223949.5	
MT0252000	PA MOGIANA II	1585.5	296311.3	0.0	0.0	2884.4	539071.6	761.3	142280.0	3645.7	681351.6	977663.0	
MT0253000	PA ESCONDIDO	1181.2	180773.0	0.0	0.0					0.0	0.0	180773.0	
MT0256000	PA BOSMAJI	1924.4	294625.7	0.0	0.0	10.3	1577.6			10.3	1577.6	296203.3	
MT0257000	PA CARACOL	2748.3	420726.6	0.0	0.0	289.3	44290.1			289.3	44290.1	465016.7	
MT0258000	PA JULIETA II	646.9	26891.2	0.0	0.0	2.7	184.9	1879.7	68097.4	1882.4	68282.4	95173.6	
MT0262000	PA RIO BRANCO	1639.1	229327.6	19.3	2721.8	1.0	137.1	41.3	5806.6	42.2	5943.7	237993.1	
MT0263000	PA SANTO ANTONIO DA ALDEIA	10238.9	1530580.4	0.0	0.0	2145.4	325970.4	300.9	18869.0	2446.3	344839.4	1875419.8	
MT0264000	PA SÃO MANOEL	20917.2	3179160.2	0.0	0.0	6138.4	986312.3	236.1	39401.9	6374.5	1025714.2	4204874.4	96.2
MT0265000	PA PONTAL DO MARAPE	33.8	2608.5	742.7	26802.1	32.0	2474.6	899.9	31311.5	931.9	33786.1	63196.6	33.8
MT0268000	PA SANTA HELENA III	860.3	84183.4	301.4	35842.0	411.2	32496.0	3222.3	139211.2	3633.4	171707.2	291732.6	
MT0269000	PA SANTANA DO TAQUARAL	336.2	27235.4	0.0	0.0	144.7	12098.8	1643.5	131039.6	1788.2	143138.4	170373.8	
MT0274000	PA BARRANQUEIRA	2742.8	394434.6	0.0	0.0	149.6	22428.8			149.6	22428.8	416863.4	
MT0275000	PA SÃO SATURNINO	7902.8	1146588.3	0.0	0.0	1618.3	233941.4	679.5	95719.1	2297.8	329660.5	1476248.8	
MT0278000	PA ROSELI NUNES	13707.7	2116075.0	1169.0	182221.7	5060.5	789896.1			5060.5	789896.1	3088192.8	
MT0279000	PA SANTANA DA ÁGUA LIMPA	385.9	55727.6	0.0	0.0	175.1	25566.6	620.1	48364.5	795.2	73931.1	129658.7	
MT0282000	PA IPÊ ROXO	793.4	47950.4	43.1	2264.1	126.1	7548.3	7.4	242.5	133.5	7790.8	58005.3	
MT0292000	PA RIBEIRÃO DA GLÓRIA	0.0	0.0	1079.2	36218.5			714.1	24869.5	714.1	24869.5	61088.0	
MT0293000	PA ALMINHAS	0.0	0.0	1020.2	92848.5			322.8	27724.7	322.8	27724.7	120573.2	
MT0294000	PA TAMBORIL	811.9	136306.3	0.0	0.0	8.6	1447.7			8.6	1447.7	137754.0	
MT0295000	PA UNIÃO FLOR DA SERRA	33107.5	5783138.0	0.0	0.0	22245.5	3920149.3	7513.4	1395807.8	29759.0	5315957.1	11099095.1	
MT0297000	PA JAPURANÃ	9392.8	930252.3	0.0	0.0	818.7	119729.5	733.1	108600.1	1551.8	228329.6	1158581.8	
MT0299000	PA TRIUNFO	0.0	0.0	2944.8	53668.8			5791.7	149658.0	5791.7	149658.0	203326.7	
MT0300000	PA COLORADO	0.0	0.0	322.2	45332.8			64.3	9051.0	64.3	9051.0	54383.8	
MT0301000	PA ÁGUAS DA SERRA	2657.6	352863.2	106.9	15045.4	102.5	14423.7	237.0	30594.7	339.5	45018.4	412927.1	
MT0303000	PA 17 DE MARÇO	3370.0	565808.5	0.0	0.0	28.5	4779.9			28.5	4779.9	570588.4	

MT0304000	PA COLNIZA - II	31637.2	5858834.8	0.0	0.0	12310.2	2281840.1					12310.2	2281840.1	8140674.8	
MT0305000	PA COLNIZA-I	17995.3	3014723.2	0.0	0.0	8749.5	1475728.5					8749.5	1475728.5	4490451.8	
MT0306000	PA ESCOL SUL	8111.2	1483059.3	0.0	0.0	4164.9	771100.6					4164.9	771100.6	2254159.9	113.6
MT0307000	PA NATAL	2074.4	342122.0	0.0	0.0	220.8	35982.5					220.8	35982.5	378104.5	
MT0308000	PA SÃO GABRIEL	0.0	0.0	1306.3	43588.7	0.6	48.9	771.2	26019.0			771.9	26067.9	69656.6	
MT0309000	PA RAIZAMA	0.0	0.0	964.5	31819.2			573.1	18905.4			573.1	18905.4	50724.6	
MT0311000	PA CÓRREGO RICO	1966.1	293985.3	3743.6	193700.0	2079.3	317332.6	756.7	102154.0			2836.0	419486.6	907171.8	
MT0312000	PA PALOMA	0.0	0.0	0.0	0.0			3352.3	200896.1			3352.3	200896.1	200896.1	
MT0313000	PA CORIXO	394.1	19533.3	0.0	0.0	52.5	3645.1	842.4	49053.7			894.9	52698.9	72232.2	
MT0314000	PA SAPICUÁ	0.0	0.0	0.0	0.0			11405.4	755640.5			11405.4	755640.5	755640.5	
MT0315000	PA LIMOEIRO	9010.0	750906.2	0.0	0.0	3296.8	324188.5					3296.8	324188.5	1075094.7	48.1
MT0316000	PA SANTO ANTONIO DO FONTOURA III	877.5	123351.7	11.4	1496.5	61.8	8687.1	0.6	85.9			62.4	8772.9	133621.2	
MT0321000	PA JOÃO DE BARRO	1456.7	204926.5	1236.3	166535.2			115.2	16201.7			115.2	16201.7	387663.5	
MT0322000	PA JOÃO PESSOA	544.9	76658.4	34.6	4872.5	10.5	1480.9	124.2	17476.5			134.8	18957.4	100488.3	
MT0324000	PA TIETÊ	3764.9	267720.4	1063.8	45006.8	289.8	37144.3	123.9	11036.5			413.7	48180.8	360908.0	1.3
MT0325000	PA CARLOS MARIGHELA-PONTAL DA AREIA	0.0	0.0	991.3	38439.5			289.9	14495.1			289.9	14495.1	52934.6	
MT0326000	PA SANTO ANTONIO DO NORTE	1687.0	250405.6	0.0	0.0	411.3	59714.0					411.3	59714.0	310119.6	
MT0329000	PA LAGOA RICA	2987.0	438538.0	91.0	13363.2	618.6	90812.6					618.6	90812.6	542713.8	
MT0330000	PA CEDRO ROSA	10000.6	1633595.2	0.0	0.0	2444.9	398494.7					2444.9	398494.7	2032090.0	
MT0331000	PA SANTO ANTONIO DO BELEZA	4213.8	445351.8	1799.2	70722.1	372.9	45066.3	492.1	45053.0			865.0	90119.4	606193.3	
MT0339000	PA BELEZA	3012.2	325768.9	2460.1	68711.7	835.0	112940.4	1214.6	95624.6			2049.6	208565.0	603045.6	
MT0344000	PA SANTO ANTONIO DA FARTURA	4148.7	616141.9	49.5	7860.1	4392.2	715165.3	447.8	72979.1	13.8	2255.5	4853.9	790399.8	1414401.9	
MT0345000	PA CHACORORÉ	2691.2	490305.9	0.0	0.0	413.3	77004.4					413.3	77004.4	567310.3	
MT0346000	PA MONTE VERDE	1055.2	116731.2	391.1	19152.6	626.8	82308.4	951.3	34732.8			1578.1	117041.2	252925.0	
MT0347000	PA PONTAL DO GLÓRIA	18748.3	1952557.3	0.0	0.0	4641.9	422370.7	1812.5	221883.3			6454.4	644254.0	2596811.3	
MT0349000	PA SÃO VICENTE	40040.0	5925591.2	0.0	0.0	9407.3	1418962.7	61.5	9165.3			9468.8	1428128.0	7353719.2	
MT0356000	PA CACHIMBO II	12455.0	1884780.0	1087.0	172809.9	739.4	117188.5					739.4	117188.5	2174778.3	68.3

MT0360000	PA JURUENA I	0.0	0.0	577.1	12051.4			2426.2	61478.1			2426.2	61478.1	73529.5	
MT0361000	PA AGROANA/GIRAU	6.5	918.6	843.5	118665.6			486.3	68413.8			486.3	68413.8	187998.0	
MT0364000	PA WILSON MEDEIROS	1466.9	245187.7	0.0	0.0	509.1	85823.4					509.1	85823.4	331011.1	4.1
MT0366000	PA VALE DA ESPERANÇA	0.0	0.0	0.0	0.0	109.4	14145.6	5497.0	175096.0			5606.4	189241.6	189241.6	
MT0443000	PE JOÃO PONCE DE ARRUDA	4076.0	605684.5	0.0	0.0	280.9	42957.3					280.9	42957.3	648641.9	
MT0444000	PA FLORESTAN FERNANDES	1313.2	202637.0	0.0	0.0	897.2	138688.3					897.2	138688.3	341325.3	
MT0457000	PA POUSO ALEGRE	1134.2	164061.5	0.0	0.0	1.2	174.9					1.2	174.9	164236.5	
MT0458000	PA SANTO EXPEDITO	0.0	0.0	3029.6	143827.5			2239.4	100398.8			2239.4	100398.8	244226.4	
MT0468000	PA SANTA CÁSSIA	699.9	102753.7	65.7	9639.4	187.9	27588.8					187.9	27588.8	139981.9	
MT0470000	PA 04 DE OUTUBRO	125.8	17695.8	500.5	52979.1			124.4	11718.0			124.4	11718.0	82392.9	
MT0471000	PA SALETE STROZAK	0.0	0.0	1079.3	35605.7			3.6	117.2			3.6	117.2	35722.9	
MT0472000	PA DORCELINA FOLADOR	0.0	0.0	663.3	20228.6			3626.2	103870.0			3626.2	103870.0	124098.6	
MT0473000	PA FRANCISCO JOSÉ NASCIMENTO	758.0	75877.4	815.6	41799.7	178.5	12991.1	702.3	33800.9			880.8	46791.9	164469.1	
MT0477000	PE SALTINHO	51.5	7564.2	178.8	26224.6	197.0	20864.7	62.6	9058.8			259.6	29923.4	63712.3	
MT0478000	PA MARIA DE OLIVEIRA	1203.7	156026.2	107.1	14389.9	434.8	55862.9	4.0	589.4			438.8	56452.2	226868.3	
MT0479000	PA MARIA DE OLIVEIRA I	446.7	59070.6	5410.2	226682.7	1196.5	151969.9	2688.0	107696.9			3884.5	259666.7	545420.1	
MT0481000	PA CAETÉ	3307.4	534429.6	0.0	0.0	10620.4	1693223.2	2216.9	346440.4			12837.3	2039663.6	2574093.2	
MT0486000	PE VIDA NOVA	5677.0	823411.1	0.0	0.0	3102.9	432328.2	5118.3	317671.9			8221.2	750000.0	1573411.1	18.9
MT0491000	PA GUAPORÉ	0.0	0.0	503.4	10152.1	21.1	1632.3	4.4	338.7	43.2	3336.0	68.7	5307.0	15459.1	
MT0492000	PA NOSSA SENHORA APARECIDA	2906.7	475169.2	0.0	0.0	752.2	126376.3					752.2	126376.3	601545.5	
MT0494000	PA BARRA NORTE	6450.1	986399.1	230.5	35243.2	683.3	104593.6					683.3	104593.6	1126235.9	
MT0495000	PA JONAS PINHEIRO	5299.8	563930.5	0.0	0.0	947.7	77968.0	4181.2	457777.9			5128.9	535745.9	1099676.5	
MT0604000	PE RP	135.7	5891.5	1255.7	142385.9	312.2	15455.5	869.6	49371.1			1181.8	64826.6	213104.0	
MT0606000	PA SERRA DA ESPERANÇA	10167.2	978436.8	45.9	1810.6	396.3	32539.7	2.4	284.3			398.7	32824.0	1013071.5	2.2
MT0607000	PA GUERREIRO	1069.4	144133.6	0.0	0.0	30.6	4387.0	413.9	15773.8			444.5	20160.8	164294.4	
MT0608000	PA GAVIÃO	1055.6	152970.9	0.0	0.0	102.2	14227.6					102.2	14227.6	167198.5	
MT0609000	PA CECILIA ANTUNES	0.0	0.0	229.4	7568.8			4279.2	141169.8			4279.2	141169.8	148738.6	

MT0610000	PA NOSSA SENHORA DA ESPERANÇA	9468.6	1578799.7	0.0	0.0	1942.5	317222.3			1942.5	317222.3	1896022.0	
MT0615000	PA TEODOMIRO FERREIRA DOS SANTOS	0.0	0.0	569.3	32897.0			819.8	57666.1	819.8	57666.1	90563.1	
MT0616000	PA SANTA EMILIA	300.2	9981.2	347.4	11505.1	391.0	12920.5	63.8	2107.9	454.8	15028.4	36514.7	
MT0621000	PA RIO PRETO	2082.8	118090.0	78.1	2958.1	951.4	46533.4	1221.2	114906.1	2172.6	161439.6	282487.7	
MT0626000	PA TABAJARA	1.2	94.5	0.0	0.0	10.5	811.6	422.8	32656.3	433.3	33467.9	33562.5	
MT0627000	PA BOM SUCESSO	12495.8	2089776.6	0.0	0.0	4040.9	671329.9			4040.9	671329.9	2761106.4	261.3
MT0628000	PA RENASCER	0.0	0.0	153.7	1447.5			193.1	2420.5	193.1	2420.5	3867.9	
MT0629000	PA FLEXAS	0.0	0.0	8849.0	291928.9			4731.2	156083.8	4731.2	156083.8	448012.8	97.4
MT0634000	PA SADIA VALE VERDE	0.0	0.0	2930.1	173588.6			1850.4	119519.2	1850.4	119519.2	293107.8	
MT0635000	PA SANTA CRUZ	5779.3	639925.5	101.1	13319.3	858.4	91900.3	4.4	145.5	862.8	92045.9	745290.7	
MT0636000	PA RAIMUNDO ROCHA	0.0	0.0	6390.3	368625.3			3591.4	236561.3	3591.4	236561.3	605186.6	
MT0639000	PA SANTA CELIA	1276.3	214281.3	0.0	0.0					0.0	0.0	214281.3	
MT0640000	PA BELMONTE	14016.4	2348025.6	0.0	0.0	6506.0	1123336.7	2478.2	419245.0	8984.2	1542581.7	3890607.2	6.2
MT0641000	PA VALE DO ARINOS	0.0	0.0	804.8	26550.5			268.4	8855.3	268.4	8855.3	35405.8	
MT0642000	PA BR-070	0.0	0.0	28780.1	1008721.9	0.4	13.4	15635.3	613232.2	15635.7	613245.6	1621967.5	306.1
MT0643000	PE AGUIA	0.0	0.0	1144.1	130133.7			1414.1	136896.8	1414.1	136896.8	267030.5	
MT0644000	PE KARAJAS	0.0	0.0	2257.0	132876.8			4672.7	233168.9	4672.7	233168.9	366045.8	
MT0645000	PE BORORO	16.1	1244.8	605.7	27665.5	31.4	2421.7	979.1	57108.7	1010.5	59530.4	88440.7	125.6
MT0646000	PE SOCO	69.5	5338.6	1120.8	40475.2	114.2	8822.2	1165.5	57473.9	1279.7	66296.1	112109.9	96.2
MT0647000	PE XORORÓ	0.0	0.0	130.3	4936.4			999.9	38474.8	999.9	38474.8	43411.3	18.6
MT0648000	PE KADVEL	0.0	0.0	545.8	18789.7			1102.7	36378.9	1102.7	36378.9	55168.6	
MT0649000	PE XAVANTE - I	0.0	0.0	74.4	2454.6			17.6	581.5	17.6	581.5	3036.1	
MT0650000	PE TUPINAMBÁ	0.0	0.0	1226.0	56053.0			1155.8	53183.7	1155.8	53183.7	109236.7	80.7
MT0651000	PE TIJUCA	0.0	0.0	778.8	25693.1			160.9	5309.5	160.9	5309.5	31002.6	
MT0652000	PE TAMOIO	0.0	0.0	4002.1	151722.4			8608.3	293909.6	8608.3	293909.6	445632.0	65.7
MT0653000	PE FORQUILHA DO RIO ARRUDA	0.0	0.0	38.6	1961.5			76.9	5832.2	76.9	5832.2	7793.7	
MT0654000	PE TUCANO	0.0	0.0	862.6	48621.0			1279.1	87235.8	1279.1	87235.8	135856.8	

MT0655000	PE MANDIOCAL	0.0	0.0	438.5	14464.8			284.1	9373.2	284.1	9373.2	23837.9	
MT0656000	PE CANARIO	0.0	0.0	291.2	9606.4			216.4	7137.6	216.4	7137.6	16744.0	
MT0657000	PE BAKAIRI	0.0	0.0	686.3	17777.0			503.4	11066.1	503.4	11066.1	28843.1	0.2
MT0658000	PE JOÃO DE BARRO	720.5	66131.5	183.0	17473.9	696.5	57387.5	5.9	456.8	702.4	57844.4	141449.8	
MT0667000	PA RIBEIRÃO GRANDE - III	2.5	174.9	0.0	0.0	80.1	4751.3	1813.2	93533.6	1893.3	98284.8	98459.7	
MT0668000	PA KATIRA	0.0	0.0	3967.3	136848.3			2422.9	85875.1	2422.9	85875.1	222723.4	23.1
MT0683000	PE PERDIZ	0.0	0.0	279.2	9209.2			440.1	14519.9	440.1	14519.9	23729.1	
MT0684000	PE CARDEAL	29.5	2275.8	5491.3	275630.0	150.5	11625.2	9849.2	474860.0	9999.7	486485.2	764391.0	123.9
MT0686000	PE BELGA	0.0	0.0	197.5	6534.9			294.6	9719.1	294.6	9719.1	16253.9	
MT0687000	PE PAVÃO	202.1	13367.7	1070.0	36916.8	253.9	17579.0	1356.0	52674.6	1609.9	70253.6	120538.2	74.8
MT0688000	PE CURIO	2983.4	434799.3	53.8	7392.7	3690.5	524623.0	534.5	73460.3	4225.0	598083.4	1040275.3	12.5
MT0689000	PA CABAÇAS	0.0	0.0	622.1	20523.0			3997.1	131865.4	3997.1	131865.4	152388.4	
MT0693000	PE CASCAVEL	0.0	0.0	156.6	5167.1			690.8	22788.6	690.8	22788.6	27955.8	
MT0694000	PE PEDRA BRANCA	0.0	0.0	491.1	16201.2			697.3	23003.1	697.3	23003.1	39204.3	
MT0696000	PE BARREIRO E CANINANA	0.0	0.0	0.0	0.0			384.9	29730.3	384.9	29730.3	29730.3	
MT0697000	PE ATERRADO	0.0	0.0	327.2	10795.3			619.4	20433.2	619.4	20433.2	31228.5	
MT0698000	PE BURITI DO ATALHO	0.0	0.0	7301.5	240712.9			702.3	22420.7	702.3	22420.7	263133.6	
MT0701000	PA MESTRE	0.0	0.0	720.6	23773.3			335.0	12919.3	335.0	12919.3	36692.6	
MT0702000	PA FREI SERVACIO SCHULT	0.0	0.0	418.7	13813.6			951.1	31376.6	951.1	31376.6	45190.3	
MT0703000	PA SÃO BENTO	0.0	0.0	747.9	105710.0			2528.8	326778.0	2528.8	326778.0	432488.0	
MT0704000	PA VALE DO PRATA	0.0	0.0	3372.3	98811.8			801.1	33954.9	801.1	33954.9	132766.7	22.0
MT0705000	PA NOSSA SENHORA APARECIDA I	6200.0	340777.2	117.5	4800.2	775.0	54920.1	2119.4	109762.3	2894.4	164682.4	510259.8	
MT0706000	PA SANTA LUCIA	0.0	0.0	72.3	2384.0			153.5	5063.3	153.5	5063.3	7447.3	
MT0709000	PE CAPÃO VERDE I	0.0	0.0	258.9	8541.8			108.6	3583.4	108.6	3583.4	12125.3	
MT0711000	PE ÁGUA VERMELHA	77.3	10619.5	0.0	0.0	352.9	48085.1	582.8	61322.1	935.7	109407.2	120026.8	
MT0719000	PA FLOR DA MATA	0.0	0.0	1750.9	46097.5			830.5	67096.3	830.5	67096.3	113193.8	
MT0720000	PA SANTA FILOMENA	401.8	70937.5	0.0	0.0	87.5	15723.5	546.6	84923.8	634.1	100647.3	171584.8	

MT0724000	PA ALIANÇA	1202.4	184105.9	0.0	0.0	1219.7	186861.9	693.9	106235.0	1913.5	293096.9	477202.7	
MT0725000	PA CRISTALINO	580.8	89262.1	0.0	0.0	302.0	46281.2	1297.8	198715.7	1599.8	244996.9	334259.0	
MT0726000	PA CRISTALINO II	518.7	79451.7	0.0	0.0	156.8	24000.2	555.6	85058.7	712.3	109058.9	188510.6	
MT0727000	PA CRISTALINO - IV	0.0	0.0	472.7	36514.9			1364.6	105401.1	1364.6	105401.1	141916.0	
MT0728000	PA 26 DE JANEIRO	4436.1	727253.5	0.0	0.0	11537.0	1880227.6			11537.0	1880227.6	2607481.1	
MT0731000	PA ANTONIO SOARES	0.0	0.0	132.1	18577.3			1645.5	231483.5	1645.5	231483.5	250060.8	
MT0732000	PA FURNAS	2423.0	359927.2	0.0	0.0	2.1	294.6			2.1	294.6	360221.9	
MT0733000	PA NOSSA SENHORA DE FÁTIMA	1124.7	168636.9	0.0	0.0	199.1	30346.7			199.1	30346.7	198983.6	
MT0734000	PA PADRE JOSÉ TENCATE	479.8	67801.2	0.0	0.0	62.0	5008.4	1425.5	144331.9	1487.5	149340.3	217141.5	
MT0735000	PA UIRAPURU	957.6	140392.6	0.0	0.0	241.1	34126.9			241.1	34126.9	174519.5	
MT0736000	PA LOURIVAL D┤ ABIC	3602.4	590994.3	0.0	0.0	6169.4	1004662.5	60.0	9778.9	6229.4	1014441.4	1605435.7	
MT0737000	PA VIDA NOVA II	37105.6	5352609.6	701.9	101919.4	17934.9	2716064.5	150.1	7307.2	18085.0	2723371.7	8177900.8	431.8
MT0744000	PDS BORDOLÃNDIA	599.0	94730.3	0.0	0.0	197.6	31992.8			197.6	31992.8	126723.1	
MT0746000	PA SANTO ANTONIO	718.3	101046.4	67.3	9173.1	48.4	6815.7	445.1	62432.6	493.6	69248.3	179467.8	
MT0750000	PA 28 DE DEZEMBRO	2873.9	404305.6	2.5	358.0	403.0	56698.8			403.0	56698.8	461362.4	
MT0753000	PA MÁRTIRES DOS CARAJÁS	0.0	0.0	322.6	10643.6			4067.7	156752.7	4067.7	156752.7	167396.3	
MT0754000	PA PONTE DE BARRO	0.0	0.0	176.7	5830.2			4545.4	149953.7	4545.4	149953.7	155783.9	
MT0755000	PA FACÃO/BOM JARDIM	0.0	0.0	271.0	8940.2			188.2	6209.3	188.2	6209.3	15149.5	
MT0756000	PA DIOCESE DE DIAMANTINO/MT	2604.2	385716.3	0.0	0.0	1308.0	182265.6	1302.8	128410.1	2610.8	310675.7	696392.0	
MT0758000	PA MARUMBI	2068.4	344340.7	0.0	0.0	944.5	156377.4			944.5	156377.4	500718.1	
MT0760000	PA PINHEIRO VELHO	5777.2	857179.3	0.0	0.0	1044.2	153753.4	0.0	0.6	1044.2	153754.0	1010933.3	
MT0764000	PA SÃO JUDAS	619.8	98646.8	0.0	0.0	47.2	7563.8			47.2	7563.8	106210.6	
MT0768000	PA SANTO ANTONIO I	756.2	121407.2	0.0	0.0	6.1	999.0			6.1	999.0	122406.2	
MT0769000	PA SANTO ANTONIO II	6552.8	1016185.8	0.0	0.0	9364.8	1447526.6	6637.7	1017294.6	16002.6	2464821.2	3481007.0	
MT0776000	PA PLANALTO DO IRIRI	1808.6	338005.9	0.0	0.0	1096.6	204950.0	587.7	109830.4	1684.3	314780.4	652786.3	
MT0777000	PA ARAUNA	654.6	122099.4	0.0	0.0	496.6	92374.7	468.7	72670.1	965.3	165044.8	287144.2	
MT0778000	PA ARAUNA II	0.0	0.0	240.3	8357.4			102.3	7647.3	102.3	7647.3	16004.7	

MT0781000	PA VISTA ALEGRE	0.0	0.0	6241.2	200859.6			9836.8	310910.3	9836.8	310910.3	511769.9	
MT0782000	PA ARRAIAL SANTANA	303.8	46434.0	0.0	0.0	36.2	5543.9			36.2	5543.9	51977.9	
MT0789000	PA BOA ESPERANÇA I	3084.0	464626.1	0.0	0.0	679.8	103392.6			679.8	103392.6	568018.7	
MT0793000	PA CANÃA I	2211.3	317989.5	0.0	0.0	1914.1	274602.9	4076.8	146552.5	5990.9	421155.4	739144.9	
MT0794000	PA SÃO PEDRO/CAMBARA	3071.4	446095.2	51.1	7109.3	1426.9	199443.1	344.0	33263.5	1770.9	232706.5	685911.0	
MT0798000	PA EMA	7120.0	904639.9	613.6	74304.2	3718.4	432397.5			3718.4	432397.5	1411341.7	
MT0799000	PA PORTO VELHO	3723.4	568112.7	0.0	0.0	2789.5	426980.2			2789.5	426980.2	995093.0	
MT0807000	PA ZUMBI DOS PALMARES II	438.6	68059.6	0.0	0.0	2011.0	324935.0			2011.0	324935.0	392994.6	
MT0808000	PDS SONHO DE ANDERSON	0.0	0.0	3143.7	103709.6			2902.0	95738.4	2902.0	95738.4	199448.0	
MT0809000	PA MARIA BENVINDA DE AVILA SOARES	0.0	0.0	7153.9	257226.9	7.9	523.7	3279.3	334714.4	3287.2	335238.0	592465.0	
MT0810000	PA DOM OZORIO STOFELL	5636.3	699634.8	2360.7	158309.7	939.2	91299.8	1252.4	79300.5	2191.5	170600.3	1028544.8	178.4
MT0812000	PA SERRA VERDE	0.0	0.0	611.6	20063.2			2031.4	51833.3	2031.4	51833.3	71896.5	
MT0814000	PA MONTE AZUL	0.0	0.0	1033.1	65700.5			2968.2	413590.1	2968.2	413590.1	479290.6	
MT0815000	PA BRIDÃO BRASILEIRO	13785.2	1306596.8	0.0	0.0	4929.5	442220.5	45.3	4231.0	4974.7	446451.6	1753048.4	
MT0816000	PA MIURA	1594.6	178891.2	0.0	0.0	1010.7	144652.6			1010.7	144652.6	323543.8	
MT0817000	PA BARRA DO MARCO	503.7	75690.1	0.0	0.0	272.5	41701.6			272.5	41701.6	117391.7	
MT0818000	PA MEDALHA MILAGROSA	17955.9	3116414.0	0.0	0.0	9884.7	1744951.6	208.0	38871.4	10092.7	1783823.1	4900237.1	
MT0819000	PA PRIMOROSA	1381.4	49001.4	6514.6	234330.6	2153.1	105657.8	20417.4	658709.6	22570.5	764367.4	1047699.4	
MT0820000	PDS NOVO RENASCER	765.1	116630.7	0.0	0.0	185.1	28314.9			185.1	28314.9	144945.6	
MT0821000	PDS TERRA DE VIVER	353.0	54043.4	0.0	0.0	862.4	132028.5			862.4	132028.5	186071.9	
MT0822000	PDS FREI CRISPIN	325.6	49845.1	0.0	0.0	1337.7	204744.6			1337.7	204744.6	254589.7	
MT0824000	PDS OLGA BENARIO	443.2	67652.8	0.0	0.0	1014.1	155258.3			1014.1	155258.3	222911.1	
MT0827000	PA JAPURANOMANN	14284.6	2650622.6	0.0	0.0	14439.2	2697403.6			14439.2	2697403.6	5348026.2	
MT0835000	PA RECOMPENSA II	0.0	0.0	0.0	0.0			1199.0	39554.8	1199.0	39554.8	39554.8	
MT0837000	PDS SÃO PAULO	963.1	161364.7	0.0	0.0	1392.8	227098.8			1392.8	227098.8	388463.5	
MT0838000	PDS NOVA ALIANÇA	1331.2	203748.3	0.0	0.0	1100.3	168460.5			1100.3	168460.5	372208.7	

MT0840000	PDS NOVA CONQUISTA	1335.4	198843.0	0.0	0.0	1091.7	159917.4					1091.7	159917.4	358760.3	
MT0841000	PDS KENO	10036.3	1522800.5	0.0	0.0	12342.5	1889256.9					12342.5	1889256.9	3412057.4	
MT0845000	PA SANTA RITA	3.8	64.5	515.3	23785.0	426.8	12823.7	23812.0	1150249.9			24238.8	1163073.6	1186923.1	
MT0853000	PA GUATAPARA	0.0	0.0	2775.6	98206.4			4115.5	184641.1			4115.5	184641.1	282847.5	
MT0854000	PA PAU DALHO	0.0	0.0	0.0	0.0	682.5	93598.2	366.8	13783.6			1049.3	107381.8	107381.8	73.9
MT0855000	PA BARRA DO MARCO II	60.1	9001.6	0.0	0.0							0.0	0.0	9001.6	
MT0856000	PDS 12 DE OUTUBRO	2678.0	404671.5	0.0	0.0	3654.5	558834.9					3654.5	558834.9	963506.4	
MT0857000	PA SILVIO RODRIGUES	2859.0	357306.9	0.0	0.0	297.6	31919.7	1137.9	67129.9			1435.5	99049.5	456356.5	
Total in Mato Grosso		2325215.5	334118845.9	466772.6	22994767.4	716765.2	104271180.4	813100.5	45515010.9	577.7	83813.8	1530443.5	149870005.1	506983618.4	8581.0
**PARÁ**			0.0	0.0	0.0							0.0	0.0	0.0	
MB0001000	PA RIBEIRÃO DE FOGO	8216.2	1321811.7	0.0	0.0	187.3	30012.2					187.3	30012.2	1351824.0	
MB0002000	PA CASTANHAL ARARAS	3803.7	706693.9	0.0	0.0	1180.0	220072.7					1180.0	220072.7	926766.6	6.6
MB0003000	PA COLONIA VERDE BRASILEIRA	28411.8	4722399.4	0.0	0.0	2468.1	403129.9					2468.1	403129.9	5125529.3	2.9
MB0004000	PA TUERE	53799.0	10044700.4	0.0	0.0	48090.6	8986435.5			778.2	145440.2	48868.8	9131875.7	19176576.1	
MB0005000	PA JONCON/3 IRMÃOS	15916.6	2670321.2	0.0	0.0	1318.6	218517.6	4698.8	788281.3			6017.4	1006798.9	3677120.1	85.7
MB0006000	PA JACUNDA	9457.7	1752014.7	0.0	0.0	1791.5	332298.9					1791.5	332298.9	2084313.6	1256.0
MB0007000	PA INGÁ	2881.0	475239.4	0.0	0.0	2923.1	487325.6	3.6	624.8			2926.7	487950.4	963189.7	
MB0008000	PA RIO DA ESQUERDA	28644.3	5329527.8	0.0	0.0	11208.3	2091692.7					11208.3	2091692.7	7421220.5	
MB0009000	PA INDEPENDENCIA	1756.2	321148.2	0.0	0.0	47.9	8799.4					47.9	8799.4	329947.7	
MB0010000	PA ARRAIAPORÃ	3322.3	535364.5	0.0	0.0	543.5	88536.4					543.5	88536.4	623900.9	
MB0011000	PA CARAJAS II E III	64059.7	11622847.7	0.0	0.0	13315.3	2200737.2	2123.6	152626.3			15438.9	2353363.4	13976211.1	
MB0012000	PA GELADINHO/MURU MURU	33042.3	6120588.6	0.0	0.0	2751.1	512122.1			4.9	922.6	2756.0	513044.7	6633633.3	
MB0013000	PA SERENO	17871.9	3306379.9	0.0	0.0	1958.9	365114.8					1958.9	365114.8	3671494.7	
MB0014000	PA CINZEIRO	4820.1	894193.0	0.0	0.0	1056.1	196741.5					1056.1	196741.5	1090934.5	120.4
MB0015000	PA CARAJAS/TAMBORIL	12971.3	2406366.0	0.0	0.0	1945.4	362266.3					1945.4	362266.3	2768632.3	112.3
MB0016000	PA SÃO JORGE	3529.4	653555.5	0.0	0.0	313.7	52948.0	212.9	39782.8			526.6	92730.8	746286.3	
MB0017000	PA MENINA MOÇA	2105.5	350719.7	0.0	0.0	159.9	26105.2					159.9	26105.2	376824.9	

MB0018000	PA ITAIPAVAS/LOTE-143	4026.7	743425.8	0.0	0.0	145.4	24519.6			145.4	24519.6	767945.4	
MB0019000	PA LAGOA BONITA	18124.2	3354667.9	0.0	0.0	432.9	80210.9			432.9	80210.9	3434878.7	
MB0020000	PA ABÓBORA	12972.2	2400626.4	0.0	0.0	190.8	35303.2			190.8	35303.2	2435929.6	
MB0021000	PA PAULO FONTELES	15095.5	2795215.7	0.0	0.0	1447.3	268162.0			1447.3	268162.0	3063377.7	
MB0022000	PA GROTÃO DOS CABOCLOS	39117.3	6941033.1	0.0	0.0	1871.8	317111.5			1871.8	317111.5	7258144.6	
MB0023000	PA BOCA DO CARDOSO	16546.1	3077879.8	0.0	0.0	487.7	91074.0			487.7	91074.0	3168953.7	
MB0024000	PA VENEZA	9290.0	1719316.7	0.0	0.0	929.8	172197.6			929.8	172197.6	1891514.4	
MB0025000	PA TUCUMA	364613.7	67386372.6	0.0	0.0	38706.0	7082518.0			38706.0	7082518.0	74468890.6	11.7
MB0026000	PA JUSSAMA	5057.6	824236.7	0.0	0.0	578.2	94225.2	609.9	99396.0	1188.1	193621.2	1017857.9	
MB0027000	PA BELA VISTA	14744.7	2346663.4	0.0	0.0	1666.1	268102.5			1666.1	268102.5	2614765.9	10.9
MB0028000	PA ARRAIAS	2378.1	393972.6	0.0	0.0	297.4	49823.2	2296.2	385497.3	2593.6	435320.5	829293.1	
MB0029000	PA CINTURAO VERDE I E II	69762.8	12994058.7	0.0	0.0	19712.3	3680661.1			19712.3	3680661.1	16674719.8	23.3
MB0030000	PA VERMELHO E PRETO	4251.1	786714.9	0.0	0.0	41.4	7666.1			41.4	7666.1	794381.0	
MB0031000	PA CENTRO DA MATA	3977.5	664042.0	0.0	0.0	386.4	63665.1			386.4	63665.1	727707.1	
MB0032000	PA ITAIPAVAS/LOTE 142	8324.9	1540604.9	0.0	0.0	50.4	9328.7			50.4	9328.7	1549933.7	
MB0033000	PA JOÃO CANUTO	2204.3	366080.3	0.0	0.0	2007.0	327291.6			2007.0	327291.6	693372.0	
MB0034000	PA MARAJOARA	4259.4	788244.0	0.0	0.0	116.7	21592.6			116.7	21592.6	809836.6	
MB0035000	PA PALMEIRAS	20145.0	3751467.9	0.0	0.0	7420.7	1385854.9			7420.7	1385854.9	5137322.8	
MB0036000	PA PROPASA/DOIS IRMÃOS	22027.4	3664791.1	0.0	0.0	1427.2	232753.0	2598.6	426473.8	4025.8	659226.7	4324017.9	
MB0037000	PA RIO GELADO	7242.8	1353598.4	0.0	0.0	5184.9	969012.8			5184.9	969012.8	2322611.2	
MB0038000	PA SÃO JOSÉ DOS TRÊS MORROS	1757.4	292101.0	0.0	0.0	649.8	120894.6			649.8	120894.6	412995.6	
MB0039000	PA SAGITARIO	1885.6	350306.7	0.0	0.0	544.6	101675.9			544.6	101675.9	451982.6	
MB0040000	PA AGUA FRIA	7441.1	1347706.4	0.0	0.0	459.3	80262.7			459.3	80262.7	1427969.1	
MB0041000	PA CACHOEIRA PRETA	2899.9	539087.4	0.0	0.0	471.4	88026.2			471.4	88026.2	627113.6	
MB0042000	PA IPIRANGA	3615.4	669070.7	0.0	0.0	19.5	3606.4			19.5	3606.4	672677.0	
MB0043000	PA JUSSARA	2909.2	538372.3	0.0	0.0					0.0	0.0	538372.3	
MB0044000	PA GAMELEIRA	37825.1	7030640.0	0.0	0.0	2241.6	418081.7			2241.6	418081.7	7448721.7	

MB0045000	PA VALE FORMOSO	3347.2	619426.7	0.0	0.0	5.6	1027.7					5.6	1027.7	620454.4	
MB0046000	PA RIO BRANCO	7783.1	1454509.1	0.0	0.0	4176.5	780541.4					4176.5	780541.4	2235050.5	
MB0047000	PA INGÁ II	7717.5	1195610.1	0.0	0.0	598.2	100095.8	65.6	4085.5			663.8	104181.3	1299791.3	
MB0048000	PA INGÁ III	875.3	90752.7	0.0	0.0	222.3	25706.6	9053.8	394210.0			9276.2	419916.6	510669.3	
MB0049000	PA JACARE	2936.0	544668.6	0.0	0.0	142.8	26658.6			0.5	90.8	143.3	26749.4	571418.0	
MB0050000	PA RIO VERMELHO	5254.8	982001.5	0.0	0.0	945.5	176712.1					945.5	176712.1	1158713.5	
MB0051000	PA ELDORADO	7768.5	1451315.9	0.0	0.0	1072.6	200458.5					1072.6	200458.5	1651774.5	
MB0052000	PA CASTANHEIRA	4790.7	895339.4	0.0	0.0	1041.3	194607.2					1041.3	194607.2	1089946.6	
MB0053000	PA TANCREDO NEVES	18294.5	3410927.9	0.0	0.0	3597.7	672169.4					3597.7	672169.4	4083097.3	
MB0054000	PA COLONIA S. JOSE DO XINGU	41959.5	7819859.2	0.0	0.0	15434.7	2883781.2	184.0	34390.7			15618.7	2918171.9	10738031.1	
MB0055000	PA APERTAR DA HORA	3802.6	632778.2	0.0	0.0	574.1	94106.6	0.5	78.0			574.6	94184.5	726962.7	
MB0056000	PA ITACAIUNAS-AÇU	3066.6	573049.2	0.0	0.0	1294.2	241835.8					1294.2	241835.8	814885.1	
MB0057000	PA CEDRINHO	2429.4	450138.4	0.0	0.0	316.8	59012.9					316.8	59012.9	509151.3	
MB0058000	PA FORTALEZA I E II	6099.6	1129342.6	0.0	0.0	265.9	49419.4					265.9	49419.4	1178762.0	
MB0059000	PA JOVENCRELANDIA I E II	4603.3	855995.6	0.0	0.0	2663.5	497091.2					2663.5	497091.2	1353086.8	
MB0060000	PA ALDEIA I E II	9635.1	1613917.5	0.0	0.0	55.0	9052.5					55.0	9052.5	1622970.0	
MB0061000	PA LONTRA	4436.8	732502.6	0.0	0.0	314.5	52801.0					314.5	52801.0	785303.5	
MB0062000	PA PECOSA	7622.3	1167425.7	0.0	0.0	471.7	77044.0					471.7	77044.0	1244469.7	12.5
MB0063000	PA ARAGUAXIM II	23481.6	2727816.0	0.0	0.0	12842.7	1096665.4	4468.6	347129.5			17311.3	1443794.8	4171610.8	
MB0064000	PA SÃO DOMINGOS	2713.9	452187.6	0.0	0.0	146.1	23889.3					146.1	23889.3	476076.8	
MB0065000	PA TUPÃ-CIRETÃ - LOTE 160	4347.6	804569.2	0.0	0.0	28.3	5241.4					28.3	5241.4	809810.6	
MB0066000	PA PARAUAPEBAS I	2886.1	531845.2	0.0	0.0	54.7	9445.0					54.7	9445.0	541290.2	
MB0067000	PA PALMARES	3723.3	692530.7	0.0	0.0	1456.8	272131.4					1456.8	272131.4	964662.0	
MB0068000	PA ESTRELA DO PARÁ	1967.5	364824.1	0.0	0.0	91.3	16928.6	88.3	16503.4			179.7	33431.9	398256.0	
MB0069000	PA JACIRA II	1881.9	317932.7	0.0	0.0	41.1	7419.4					41.1	7419.4	325352.1	
MB0070000	PA SANTO ANTONIO	3604.0	599004.0	0.0	0.0	424.7	69366.4					424.7	69366.4	668370.4	
MB0071000	PA ITAIPAVAS II - LOTE 134	4306.2	742591.9	0.0	0.0	40.8	7046.4					40.8	7046.4	749638.3	

MB0072000	PA ITAIPAVAS III-LOTE 147	4205.4	778242.8	0.0	0.0							0.0	0.0	778242.8	
MB0073000	PA ITACAIUNAS	4102.1	764558.5	0.0	0.0	1444.9	269924.2					1444.9	269924.2	1034482.7	6.3
MB0074000	PA SANTA CRISTINA	8260.8	1363250.7	0.0	0.0	738.5	120583.5					738.5	120583.5	1483834.2	
MB0075000	PA MATA AZUL I	3654.6	597750.2	0.0	0.0	699.6	114073.4					699.6	114073.4	711823.6	
MB0076000	PA ANGELIN	9405.3	1752838.3	0.0	0.0	5028.1	939536.9			826.7	154473.7	5854.8	1094010.6	2846848.9	
MB0077000	PA CURRAL DE PEDRAS	5624.8	915100.9	0.0	0.0	320.7	53727.6					320.7	53727.6	968828.4	34.6
MB0078000	PA CANARANA	12707.9	2132002.2	0.0	0.0	829.5	138269.7	183.9	30877.5			1013.4	169147.2	2301149.4	2.3
MB0079000	PA REDENÇÃO	2326.7	432167.2	0.0	0.0	458.8	85531.3			209.4	39061.1	668.2	124592.5	556759.7	
MB0080000	PA AGRISA	8886.0	1354398.4	0.0	0.0	610.9	93107.6					610.9	93107.6	1447506.1	108.6
MB0081000	PA PARAUAPEBAS II	2912.3	538615.1	0.0	0.0	20.8	3841.8					20.8	3841.8	542456.9	
MB0082000	PA MARIA LUIZA	4335.5	651981.9	0.0	0.0	140.3	20996.2					140.3	20996.2	672978.1	6.4
MB0083000	PA MATA VERDE	4281.2	696834.4	0.0	0.0	659.0	107282.3					659.0	107282.3	804116.8	
MB0084000	PA SÃO SEBASTIÃO - LOTE 86	1960.5	320239.1	0.0	0.0	741.8	120924.4	89.7	14626.4			831.5	135550.8	455789.9	
MB0085000	PA 17 DE ABRIL	17077.5	2951681.9	0.0	0.0	250.4	41444.2					250.4	41444.2	2993126.1	
MB0086000	PA NOVO ARAGUAIA - LOTE 03	8162.0	1366844.1	0.0	0.0	1317.0	216518.1	562.2	94264.0			1879.2	310782.0	1677626.1	21.9
MB0088000	PA SUDOESTE	26337.0	4916463.4	0.0	0.0	11712.6	2188773.5			18.1	3376.4	11730.7	2192150.0	7108613.4	
MB0089000	PA GROTÃO	4680.4	779017.4	0.0	0.0	9.7	1574.7					9.7	1574.7	780592.0	
MB0090000	PA AGROPECUS	35978.0	5560495.8	0.0	0.0	5885.7	941948.6	1262.1	34670.9			7147.8	976619.5	6537115.3	
MB0091000	PA EMBAUBAL	2070.7	352284.6	0.0	0.0	187.2	30608.8					187.2	30608.8	382893.4	55.6
MB0092000	PA SÃO FRANCISCO	6865.1	1278125.1	0.0	0.0	729.5	136291.4					729.5	136291.4	1414416.5	
MB0093000	PA CAMPOS ALTOS	8299.4	1475566.1	0.0	0.0	607.5	100274.1					607.5	100274.1	1575840.2	
MB0094000	PA UBA	4164.2	770634.0	0.0	0.0	55.3	10236.8					55.3	10236.8	780870.8	
MB0095000	PA SANTA MARIA DO PONTAL	5099.8	948854.0	0.0	0.0	291.1	54398.6					291.1	54398.6	1003252.6	
MB0096000	PA SUÇUAPARA	7663.9	1255007.8	0.0	0.0	839.3	136782.8	193.3	31506.0			1032.6	168288.8	1423296.5	
MB0097000	PA RECANTÃO	2772.4	509107.1	0.0	0.0	173.2	31458.4					173.2	31458.4	540565.5	
MB0098000	PA TOCANTINS	643.3	118955.7	0.0	0.0			1463.0	263116.6			1463.0	263116.6	382072.3	
MB0099000	PA MONTES BELOS	6041.4	1120326.9	0.0	0.0	3723.4	689699.4			184.6	34302.5	3908.0	724001.9	1844328.8	
MB0100000	PA NAZARÉ	11465.1	1850156.4	0.0	0.0	807.8	131917.5					807.8	131917.5	1982073.9	

MB0101000	PA JEQUIÉ	1433.5	264884.9	0.0	0.0	1031.9	168216.5					1031.9	168216.5	433101.4	
MB0102000	PA DIACUI	2917.4	543044.2	0.0	0.0	34.9	6514.7			55.7	10406.2	90.6	16920.9	559965.1	
MB0103000	PA INAJÁ	5534.6	792326.3	0.0	0.0	123.8	13706.3	816.2	21268.9			940.0	34975.3	827301.5	
MB0104000	PA BURITIRANA	36520.9	6807614.0	0.0	0.0	7199.1	1345105.2					7199.1	1345105.2	8152719.2	
MB0105000	PAE PRAIALTA PIRANHEIRA	16664.0	3111198.8	0.0	0.0	5956.9	1113093.0			2.1	389.4	5959.0	1113482.4	4224681.2	957.3
MB0106000	PA GAÚCHA	4515.0	720436.8	0.0	0.0	228.8	36764.1					228.8	36764.1	757201.0	
MB0107000	PA PEDRO MONTEIRO DA SILVA	1157.5	214209.8	0.0	0.0	5.0	922.3					5.0	922.3	215132.1	
MB0108000	PA ESCALADA DO NORTE	1884.0	311075.5	0.0	0.0	1078.1	175832.7					1078.1	175832.7	486908.2	
MB0109000	PA MANAH	4457.0	689229.0	0.0	0.0	1816.0	292500.1	1972.0	61550.4			3788.0	354050.5	1043279.5	
MB0110000	PA BARRA MANSA	5747.7	961313.6	0.0	0.0	386.6	63893.4					386.6	63893.4	1025206.9	
MB0111000	PA RIO PRETO	27218.7	4462898.6	0.0	0.0	8442.4	1376834.6	332.9	54665.4			8775.3	1431500.0	5894398.6	
MB0112000	PA ESCALADA DO NORTE II LOTE 28	10244.7	1715691.5	0.0	0.0	1695.6	277945.5					1695.6	277945.5	1993637.0	0.0
MB0113000	PA PRIMAVERA E OUTROS	3610.7	602249.9	0.0	0.0	420.8	68911.4	333.0	55902.3			753.8	124813.7	727063.6	
MB0114000	PA ARRAIAPORÃ II	4991.0	836588.5	0.0	0.0	35.3	5765.2					35.3	5765.2	842353.7	
MB0115000	PA RAIMUNDO CORREIA - LOTE 81	4669.3	766654.1	0.0	0.0	653.4	103209.1					653.4	103209.1	869863.1	6.0
MB0116000	PA MAGDALENA NICOLINA RIVETTI	8281.8	1392821.5	0.0	0.0	820.5	137651.6					820.5	137651.6	1530473.1	
MB0117000	PA TRÊS RIOS	2275.5	328256.0	0.0	0.0	147.6	23850.8	2769.7	83471.7			2917.2	107322.4	435578.5	
MB0118000	PA MILHOMEM	1335.5	219562.1	0.0	0.0	179.8	28277.6					179.8	28277.6	247839.6	10.8
MB0119000	PA PE JOSIMO TAVARES	26141.8	4284190.0	0.0	0.0	20488.6	3074445.3	10439.1	878966.2			30927.7	3953411.5	8237601.5	547.6
MB0120000	PA POÇO RICO	6926.5	1281816.4	0.0	0.0	35.6	6595.2					35.6	6595.2	1288411.6	
MB0121000	PA OZIEL PEREIRA	19272.4	3244396.3	0.0	0.0	265.8	43407.5					265.8	43407.5	3287803.9	
MB0122000	PA PROGRESSO	15781.4	2674602.5	0.0	0.0	947.0	154494.3					947.0	154494.3	2829096.8	
MB0123000	PA BENFICA	7945.2	1477715.0	0.0	0.0	1529.0	285283.2					1529.0	285283.2	1762998.2	
MB0124000	PA LUCIANA	74353.2	13431235.5	0.0	0.0	24428.1	4303195.8	302.5	52068.2			24730.5	4355264.0	17786499.5	
MB0125000	PA LIMÃO	6916.6	1271294.9	0.0	0.0	362.2	63338.2					362.2	63338.2	1334633.1	
MB0127000	PA AÇAIZAL	2720.4	443345.0	0.0	0.0	579.2	94394.6					579.2	94394.6	537739.6	46.4
MB0128000	PA PATAUÁ	6523.5	1216069.7	0.0	0.0	371.2	69362.1					371.2	69362.1	1285431.8	

MB0129000	PA LINDOESTE	9874.5	1834579.5	0.0	0.0	2193.4	409637.4					2193.4	409637.4	2244216.9	
MB0130000	PA ANGICAL	3041.3	495632.7	0.0	0.0	413.1	67329.5					413.1	67329.5	562962.2	
MB0131000	PA JOSE MARTINS PESSOA	2805.6	521560.0	0.0	0.0	1048.9	195840.1					1048.9	195840.1	717400.1	
MB0132000	PA COCALÃNDIA	2916.7	542995.4	0.0	0.0	713.8	133304.1			393.2	73427.2	1107.0	206731.4	749726.8	
MB0133000	PA JAGUATIARA	2395.0	446069.7	0.0	0.0	661.2	123458.5					661.2	123458.5	569528.2	
MB0134000	PA BARREIRO COCAL	4217.1	780415.4	0.0	0.0	15.2	2805.4					15.2	2805.4	783220.8	
MB0135000	PA MORESCHI	4028.9	752958.0	0.0	0.0	381.1	71229.2					381.1	71229.2	824187.2	
MB0136000	PA LAJEDO	4566.2	845837.4	0.0	0.0	803.2	149243.0					803.2	149243.0	995080.4	
MB0137000	PA PARAISO DO ARAGUAIA	3959.7	694815.3	0.0	0.0	1442.3	253838.9					1442.3	253838.9	948654.2	
MB0138000	PA 1║ DE MARÇO	9327.0	1735000.4	0.0	0.0	1492.8	278770.2					1492.8	278770.2	2013770.6	23.0
MB0139000	PA SÃO PAULO DAS CACHOEIRAS	6873.9	1275221.2	0.0	0.0	387.0	72022.2			13.1	2449.2	400.1	74471.4	1349692.7	
MB0146000	PA INDIAPORÃ	2561.9	423915.1	0.0	0.0	224.8	36668.7					224.8	36668.7	460583.8	
MB0147000	PA AGROPECUS II	2002.1	200613.2	0.0	0.0	613.4	98725.5					613.4	98725.5	299338.7	
MB0148000	PA ARAXÁ	1231.8	199347.6	0.0	0.0	161.1	26257.0	251.5	19172.7			412.7	45429.7	244777.3	
MB0150000	PA ENTRE RIOS I LOTES 76	2444.8	368342.7	0.0	0.0	568.8	86509.5	1366.1	99101.4			1934.9	185610.9	553953.6	
MB0151000	PA TRAVESSÃO	18743.1	3070702.4	0.0	0.0	1751.4	285543.4					1751.4	285543.4	3356245.8	
MB0152000	PA MURIÇOCA	1182.3	199914.7	0.0	0.0	158.7	26629.0					158.7	26629.0	226543.7	
MB0153000	PA DIUTÁ	4620.6	856657.4	0.0	0.0	542.9	101119.4	27.9	5216.3			570.8	106335.7	962993.1	
MB0155000	PA FRUTÃO	15174.3	2832161.9	0.0	0.0	2950.8	551420.8					2950.8	551420.8	3383582.6	
MB0156000	PA SERRA AZUL	2908.7	543614.3	0.0	0.0	878.4	164170.1					878.4	164170.1	707784.4	
MB0157000	PA PRIMAVERA DO ARAGUAIA	2889.2	539528.5	0.0	0.0	643.2	120192.0					643.2	120192.0	659720.4	
MB0158000	PA BOA ESPERANÇA	1949.9	363592.7	0.0	0.0	408.6	76196.1					408.6	76196.1	439788.8	
MB0159000	PA VOLTA DO TAPIRAPÉ	15559.3	2904931.8	0.0	0.0	5454.8	1019356.3					5454.8	1019356.3	3924288.1	
MB0160000	PA ESCADA ALTA	3229.3	597621.9	0.0	0.0	394.9	73075.8					394.9	73075.8	670697.7	
MB0161000	PA SANTA CRUZ	1416.6	230303.2	0.0	0.0	456.5	74398.7	19.8	3223.0			476.3	77621.6	307924.8	
MB0163000	PA PIMENTEIRA	2793.0	520396.4	0.0	0.0	475.9	88896.4					475.9	88896.4	609292.8	
MB0164000	PA PENSÃO DA ONÇA	3893.7	726933.5	0.0	0.0	2891.1	540157.3			23.2	4333.1	2914.2	544490.4	1271423.9	
MB0166000	PA SÃO BRAZ	2071.9	387204.4	0.0	0.0	1001.0	187077.4					1001.0	187077.4	574281.8	

MB0167000	PA DOS BANDEIRANTES	3712.9	693832.2	0.0	0.0	933.9	174536.2					933.9	174536.2	868368.4	
MB0168000	PA CONCEIÇÃO	27565.7	4468474.7	0.0	0.0	8839.4	1440457.8	51.3	8367.2			8890.7	1448825.0	5917299.7	
MB0169000	PA HERMINIO BRITO	20036.4	3244761.9	0.0	0.0	6062.1	987896.1	240.2	39152.2			6302.3	1027048.3	4271810.2	
MB0170000	PA VOLTA GRANDE	9503.4	1774471.0	0.0	0.0	4494.3	839751.8					4494.3	839751.8	2614222.8	
MB0171000	PA SÃO RAIMUNDO	963.3	160544.9	0.0	0.0	12.7	2080.9					12.7	2080.9	162625.8	
MB0172000	PA LANA	1853.6	344365.5	0.0	0.0	527.0	98477.4					527.0	98477.4	442842.9	
MB0173000	PA BARRO PRETO	1928.1	360348.6	0.0	0.0	516.5	96523.7					516.5	96523.7	456872.3	13.8
MB0174000	PA CALIFORNIA	1195.9	223465.8	0.0	0.0	1017.2	190091.3					1017.2	190091.3	413557.1	
MB0175000	PA DO MEIO	2925.2	545170.9	0.0	0.0	470.4	87874.4					470.4	87874.4	633045.3	
MB0176000	PA GAMELEIRA AÇÚ	2750.6	512092.9	0.0	0.0	572.0	106774.7					572.0	106774.7	618867.6	
MB0177000	PA GRANDE VITORIA	2781.9	509413.1	0.0	0.0	1203.5	213681.9	642.3	112408.7			1845.8	326090.6	835503.7	80.8
MB0178000	PA TERRA ROXA	2153.3	402426.6	0.0	0.0	938.8	175428.8					938.8	175428.8	577855.4	
MB0181000	PA ENTRE RIOS II LOTE 79	269.7	28307.8	0.0	0.0	135.4	19314.8	4911.4	350245.9			5046.8	369560.7	397868.4	
MB0182000	PA ENTRE RIOS III LOTE 80	1322.3	201542.1	0.0	0.0	66.5	10143.3	2677.7	358761.8			2744.2	368905.1	570447.2	
MB0183000	PA MANUELITO	2113.6	393430.1	0.0	0.0	395.1	73722.6					395.1	73722.6	467152.7	
MB0184000	PA SERRA QUEBRADA	1652.3	307778.5	0.0	0.0	471.2	88036.1					471.2	88036.1	395814.6	1.3
MB0185000	PA SANTA AMÉLIA	1142.5	213497.6	0.0	0.0	737.0	137732.0			4.6	852.9	741.6	138584.9	352082.5	3.5
MB0186000	PA CIGANA	1271.2	236952.4	0.0	0.0	583.6	108916.8			40.9	7650.9	624.5	116567.7	353520.1	
MB0187000	PA SAO GABRIEL	1443.4	268830.1	0.0	0.0	800.1	149447.8			134.4	25028.9	934.4	174476.7	443306.9	
MB0188000	PA CROÁ	1569.6	290689.3	0.0	0.0	53.6	9943.3					53.6	9943.3	300632.6	
MB0189000	PA OITO BARRACAS	3430.4	640687.0	0.0	0.0	373.0	69702.0					373.0	69702.0	710388.9	
MB0190000	PA RIO MAR	3473.2	566541.0	0.0	0.0	255.9	41696.4					255.9	41696.4	608237.5	12.5
MB0191000	PA SÃO JOSÉ DO ARAGUAIA	4098.8	739911.9	0.0	0.0	102.3	17381.0					102.3	17381.0	757292.9	79.3
MB0192000	PA MANOEL DOS REIS	4369.7	788937.2	0.0	0.0	16.0	2604.2					16.0	2604.2	791541.4	
MB0193000	PA FRANCISCO NUNES TEIXEIRA	9307.3	1591860.5	0.0	0.0	228.9	38106.8					228.9	38106.8	1629967.3	
MB0194000	PA DJALMA CASTRO	12805.5	2114730.0	0.0	0.0	1188.1	193960.5					1188.1	193960.5	2308690.5	
MB0195000	PA CARLOS PEREIRA NUNES	2606.8	482419.0	0.0	0.0	258.5	47846.0					258.5	47846.0	530265.0	259.0
MB0196000	PA VINAGRE	2279.4	425999.3	0.0	0.0	861.1	160931.3					861.1	160931.3	586930.7	28.3

MB0197000	PA RIO PRETO MALHA II	2914.4	544512.6	0.0	0.0	705.4	131829.1					705.4	131829.1	676341.7	
MB0198000	PA GOIANOS	3549.7	663411.3	0.0	0.0	1597.5	298553.4					1597.5	298553.4	961964.7	56.0
MB0200000	PA CASTANHEIRA II	3456.5	563311.8	0.0	0.0	319.3	52037.2					319.3	52037.2	615349.0	
MB0201000	PA CRISTO REI	2632.5	487689.4	0.0	0.0	665.5	123736.8					665.5	123736.8	611426.2	
MB0202000	PA BETH	1819.4	339342.7	0.0	0.0	182.1	34001.2					182.1	34001.2	373343.9	
MB0203000	PA BELO HORIZONTE	3451.1	638874.3	0.0	0.0	76.7	14235.3					76.7	14235.3	653109.6	
MB0204000	PA ARRAIALANDIA	4953.6	923100.0	0.0	0.0	1380.6	257802.8					1380.6	257802.8	1180902.8	
MB0206000	PA RIO ITACOROA	2516.3	465937.5	0.0	0.0	1433.5	265658.9			92.0	17168.3	1525.6	282827.2	748764.7	0.9
MB0208000	PA SANTA FÉ	2362.1	439893.9	0.0	0.0	674.6	126046.6			125.7	23492.5	800.3	149539.1	589433.0	
MB0209000	PA LA ESTANÇIA	2779.3	516630.1	0.0	0.0	544.7	101230.1					544.7	101230.1	617860.2	
MB0210000	PA CONQUISTA	2069.8	385706.0	0.0	0.0	680.4	127158.7					680.4	127158.7	512864.7	
MB0211000	PA MURAJUBA	1703.3	317712.1	0.0	0.0	682.9	127623.1					682.9	127623.1	445335.2	
MB0212000	PA PRINCESA	2226.8	416006.7	0.0	0.0	696.9	130199.2					696.9	130199.2	546205.9	85.9
MB0213000	PA TARTARUGA	2621.7	489918.1	0.0	0.0	981.4	183177.3					981.4	183177.3	673095.4	108.8
MB0214000	PA IGUAÇU	904.9	168890.3	0.0	0.0	167.5	31299.5					167.5	31299.5	200189.8	
MB0217000	PA BARREIRA BRANCA	3362.9	602172.8	0.0	0.0	63.4	10345.0					63.4	10345.0	612517.8	
MB0218000	PA VALE DO MUCURA	9899.9	1647351.4	0.0	0.0	904.4	147475.9					904.4	147475.9	1794827.3	
MB0219000	PA DOURADA	2147.6	397634.6	0.0	0.0	50.8	9448.7					50.8	9448.7	407083.4	
MB0220000	PA VOLTINHA DO ITACAIUNAS	885.3	165404.7	0.0	0.0	515.3	96301.9	73.8	13795.9			589.1	110097.8	275502.4	69.3
MB0221000	PA BRASILESPANHA	3354.4	620758.9	0.0	0.0	235.3	43550.9					235.3	43550.9	664309.8	
MB0222000	PA BRASIL NOVO	1953.7	365126.0	0.0	0.0	555.5	103813.1					555.5	103813.1	468939.1	
MB0223000	PA ALTO BONITO	2781.9	519896.0	0.0	0.0	780.7	145875.9					780.7	145875.9	665771.9	0.0
MB0224000	PA JATOBÁ	1838.5	341326.6	0.0	0.0	144.4	26925.9					144.4	26925.9	368252.5	
MB0225000	PA SAPUCAIA	3079.3	573698.8	0.0	0.0	160.8	30047.2					160.8	30047.2	603745.9	
MB0226000	PA SÃO SEBASTIÃO DO XINGÚ	8837.4	1644300.6	0.0	0.0	3611.1	672508.7					3611.1	672508.7	2316809.4	
MB0227000	PA MARIA RITA	2684.7	464497.5	0.0	0.0	127.8	20902.4					127.8	20902.4	485399.9	
MB0228000	PA PIMENTA	5393.4	849788.8	0.0	0.0	872.9	133914.9	138.6	6631.6			1011.5	140546.5	990335.3	
MB0229000	PA PAU BRASIL	15423.7	2520288.7	0.0	0.0	5690.5	940089.2					5690.5	940089.2	3460377.9	5.9

MB0230000	PA AIRTON SENA	9562.9	1564638.9	0.0	0.0	4124.2	679094.8					4124.2	679094.8	2243733.7	
MB0231000	PA RIO CRISTALINO	11635.8	1896743.2	0.0	0.0	4191.9	686658.6					4191.9	686658.6	2583401.8	
MB0232000	PA NICOBRAN	20182.0	3322351.2	0.0	0.0	7952.9	1298389.8					7952.9	1298389.8	4620741.0	
MB0233000	PA RIO CAMPO ALEGRE	10212.0	1663871.0	0.0	0.0	3586.1	580883.9					3586.1	580883.9	2244754.9	
MB0234000	PA PAJEÚ	2090.3	389129.9	0.0	0.0	622.9	116387.2			12.2	2279.5	635.1	118666.8	507796.6	
MB0235000	PA SANTA IZABEL	1816.5	337171.3	0.0	0.0	483.6	90054.2			19.3	3581.6	502.9	93635.8	430807.0	
MB0236000	PA CODESPAR	10277.7	1709908.5	0.0	0.0	133.4	21786.0					133.4	21786.0	1731694.5	
MB0237000	PA INAJA II	10178.2	1606343.0	0.0	0.0	810.3	110240.0	0.2	17.6			810.5	110257.5	1716600.5	
MB0238000	PA EXPEDITO RIBEIRO	1895.6	334962.5	0.0	0.0	239.7	40083.5	396.1	73221.9			635.8	113305.4	448268.0	
MB0239000	PA BRASIL 500	8104.6	1341373.9	0.0	0.0	348.8	56891.6	3.4	561.7			352.2	57453.3	1398827.2	
MB0240000	PA NOVO MUNDO	17739.1	2926952.1	0.0	0.0	1762.5	287642.2					1762.5	287642.2	3214594.3	
MB0241000	PA ALTO BONITO DO AXIXÁ	5656.5	1057134.8	0.0	0.0	978.8	182929.7					978.8	182929.7	1240064.6	
MB0242000	PA RAINHA	3666.3	679263.3	0.0	0.0	846.7	157598.4					846.7	157598.4	836861.7	6.4
MB0244000	PA BOM JARDIM DA VOLTINHA	1973.9	368856.4	0.0	0.0	992.4	185474.1					992.4	185474.1	554330.6	22.9
MB0245000	PA SÃO GERALDO	5182.7	965017.8	0.0	0.0	865.4	161654.6			4.1	770.0	869.5	162424.6	1127442.4	
MB0246000	PA MOÇA BONITA	2763.0	515374.7	0.0	0.0	658.0	122939.4					658.0	122939.4	638314.1	
MB0247000	PA NOVA ITAPERUNA	2030.9	378239.9	0.0	0.0	712.4	133101.1					712.4	133101.1	511341.0	
MB0248000	PA CASARÃO	2937.3	545640.5	0.0	0.0	136.4	25487.9			0.0	7.3	136.4	25495.3	571135.7	
MB0249000	PA BOA ESPERANÇA DO BURGO	3878.6	723315.1	0.0	0.0	897.1	167631.5					897.1	167631.5	890946.6	
MB0250000	PA SABINO SÃO PEDRO	2355.4	434748.7	0.0	0.0	148.9	27005.9					148.9	27005.9	461754.6	
MB0251000	PA TERRA NOVA	5572.3	1031195.6	0.0	0.0	167.0	30913.1					167.0	30913.1	1062108.7	
MB0252000	PA BOM LUGAR	2358.9	436544.3	0.0	0.0	0.1	21.8					0.1	21.8	436566.1	
MB0253000	PA TIRA CATINGA	1591.0	294433.6	0.0	0.0							0.0	0.0	294433.6	
MB0255000	PA CABANAGEM	1695.1	316703.5	0.0	0.0	771.0	144056.7					771.0	144056.7	460760.3	
MB0256000	PA CÍCO	6509.3	1216376.2	0.0	0.0	4793.0	895730.0					4793.0	895730.0	2112106.2	
MB0257000	PA MARAVILHA	7497.6	1401225.7	0.0	0.0	2736.9	511505.1					2736.9	511505.1	1912730.8	
MB0258000	PA CARLOS FONSECA	4203.6	778741.6	0.0	0.0	227.5	42315.2					227.5	42315.2	821056.8	
MB0259000	PA CAJARANA	1032.6	192937.7	0.0	0.0	464.7	86822.8					464.7	86822.8	279760.5	

MB0260000	PA SANTA MARIA DO ITACAIUNAS	1704.6	318575.7	0.0	0.0	701.3	131063.3					701.3	131063.3	449639.0	
MB0262000	PA CONTINENTAL	2430.6	451604.1	0.0	0.0	336.2	62783.6					336.2	62783.6	514387.7	
MB0263000	PA LAGO VERMELHO	4225.3	787911.4	0.0	0.0	1548.4	289297.2					1548.4	289297.2	1077208.6	
MB0264000	PA MONTEPIO	5918.8	1094374.0	0.0	0.0	150.3	27813.8					150.3	27813.8	1122187.8	
MB0265000	PA BRASILIA	5349.3	988839.3	0.0	0.0	97.9	17795.0					97.9	17795.0	1006634.3	
MB0266000	PA CHICO MENDES II	1452.5	269778.0	0.0	0.0	449.2	83817.6			609.9	113774.3	1059.1	197591.9	467369.9	
MB0267000	PA NOSSA SENHORA DO PERPÉTUO SOCORRO	1488.1	275108.1	0.0	0.0	143.0	26384.3					143.0	26384.3	301492.4	
MB0268000	PA CHICO MENDES I	870.0	161914.7	0.0	0.0	341.2	63753.7			928.1	173363.3	1269.3	237116.9	399031.7	
MB0269000	PA ALEGRIA	3149.6	587946.7	0.0	0.0	427.3	79850.8					427.3	79850.8	667797.5	59.0
MB0270000	PA MÃE MARIA	2619.0	484977.8	0.0	0.0	683.7	126715.3					683.7	126715.3	611693.1	6.9
MB0271000	PA ESTRELA DO NORTE	4079.4	760357.3	0.0	0.0	1097.4	204924.3					1097.4	204924.3	965281.6	
MB0272000	PA BOA SORTE	1194.7	217370.1	0.0	0.0	694.1	125784.7			33.8	6194.2	728.0	131979.0	349349.1	
MB0273000	PA CAPSS	18114.5	2990357.0	0.0	0.0	1476.2	240858.2					1476.2	240858.2	3231215.2	
MB0274000	PA CASA BRANCA	1805.0	336315.2	0.0	0.0	1116.9	208670.2					1116.9	208670.2	544985.3	
MB0275000	PA CHIBIL	2114.7	348088.5	0.0	0.0	472.3	77116.3	94.3	15788.5			566.5	92904.7	440993.2	
MB0276000	PA SANTA EUDOXIA	2940.8	490464.7	0.0	0.0	73.4	12015.2					73.4	12015.2	502479.8	
MB0277000	PA BARRA DO CEDRO	542.5	100956.0	0.0	0.0	449.0	83885.8					449.0	83885.8	184841.8	
MB0278000	PA MARINGÁ	3774.2	652766.0	0.0	0.0	387.8	63715.3					387.8	63715.3	716481.4	49.0
MB0279000	PA MIGUEL GOMES DA SILVA	1372.6	227741.9	0.0	0.0	73.4	12051.3					73.4	12051.3	239793.2	
MB0280000	PA LAGO AZUL	2532.5	468987.8	0.0	0.0	232.7	43220.0	705.9	126612.9			938.6	169832.8	638820.6	86.1
MB0281000	PA BOQUEIRÃO	2556.8	465109.3	0.0	0.0	106.3	19361.7	22.0	1830.9			128.4	21192.5	486301.8	
MB0282000	PA PEDRA PRETA	2431.6	386345.1	0.0	0.0	160.8	24531.8					160.8	24531.8	410876.9	
MB0283000	PA UIRAPURÚ	1944.3	363375.8	0.0	0.0	1128.3	210869.4					1128.3	210869.4	574245.1	
MB0284000	PA RANCHARIA	1738.7	324149.6	0.0	0.0	1388.0	259097.8					1388.0	259097.8	583247.4	
MB0286000	PA VISTA ALEGRE	1500.4	279123.4	0.0	0.0	517.7	96678.8			80.9	15107.3	598.6	111786.1	390909.5	
MB0287000	PA ACAPU	969.1	180247.0	0.0	0.0	51.2	9484.0			0.7	126.5	51.8	9610.5	189857.5	
MB0288000	PA VALE DA SERRA	2051.2	379976.4	0.0	0.0	109.0	20176.7	25.4	4744.3			134.4	24921.0	404897.4	

MB0289000	PA UNIAO DA VITORIA	3807.8	703440.4	0.0	0.0	352.6	64838.5			352.6	64838.5	768278.9
MB0290000	PA JERONIMO NUNES LACERDA	3651.5	673715.7	0.0	0.0	106.0	18926.9			106.0	18926.9	692642.7
MB0291000	PA MARIA DE LOURDES RODRIGUES	4154.8	768885.8	0.0	0.0	30.0	5544.5			30.0	5544.5	774430.3
MB0292000	PA LARANJEIRAS	5517.7	1029430.3	0.0	0.0	1773.9	331451.0			1773.9	331451.0	1360881.4
MB0293000	PA RIO PARA	3255.0	607949.2	0.0	0.0	2168.8	405216.6			2168.8	405216.6	1013165.8
MB0294000	PA RIO CINZA	4313.6	804521.8	0.0	0.0	1302.3	243382.3			1302.3	243382.3	1047904.1
MB0295000	PA RIO NEGRO	2996.1	559831.1	0.0	0.0	1393.0	260328.2			1393.0	260328.2	820159.3
MB0296000	PA ARAPARI	9408.6	1756264.3	0.0	0.0	4816.1	900077.0			4816.1	900077.0	2656341.3
MB0297000	PA EMÍDIO BATISTA DE MOURA	11466.8	2122054.6	0.0	0.0	269.3	49828.8			269.3	49828.8	2171883.4
MB0298000	PA 21 DE ABRIL	1705.1	315609.6	0.0	0.0	69.8	12954.1			69.8	12954.1	328563.7
MB0299000	PA VALENTIM SERRA	1986.8	369259.9	0.0	0.0	301.3	56289.4			301.3	56289.4	425549.3
MB0306000	PA BEIRA RIO	1307.1	244292.6	0.0	0.0	563.8	105362.4			563.8	105362.4	349655.0
MB0307000	PA BURGO	3142.5	584049.2	0.0	0.0	451.8	84294.5			451.8	84294.5	668343.7
MB0308000	PA CARIMÃ	1454.7	270719.5	0.0	0.0	625.3	116853.3			625.3	116853.3	387572.8
MB0309000	PA BELO MIRAR	3297.8	613241.5	0.0	0.0	194.1	36130.9			194.1	36130.9	649372.4
MB0310000	PA NOSSA SENHORA DAS GRAÇAS	3395.6	630789.9	0.0	0.0	169.8	31668.1			169.8	31668.1	662458.1
MB0311000	PA BOCA DO LAGO	2906.2	542437.7	0.0	0.0	466.3	87132.3			466.3	87132.3	629570.0
MB0313000	PA BORRACHEIRA	2143.6	400619.5	0.0	0.0	424.2	79282.1			424.2	79282.1	479901.6
MB0314000	PA ARICA	1653.7	300013.6	0.0	0.0	0.7	121.0			0.7	121.0	300134.6
MB0315000	PA MINEIRO PRETO	6668.1	1246082.0	0.0	0.0	3277.2	612445.6			3277.2	612445.6	1858527.6
MB0316000	PA ALTO PACAJÁ	9429.6	1762292.6	0.0	0.0	4030.2	753196.7			4030.2	753196.7	2515489.3
MB0317000	PA BOCA LARGA	4893.6	914571.5	0.0	0.0	2072.5	387331.2			2072.5	387331.2	1301902.7
MB0318000	PA JACARÉ-AÇÚ	6163.4	1151876.9	0.0	0.0	5362.0	1002105.6			5362.0	1002105.6	2153982.5
MB0319000	PA JOSÉ CIRILO GOMES	7137.6	1333409.4	0.0	0.0	3042.6	568548.4			3042.6	568548.4	1901957.8
MB0320000	PA RIO PRETO	3809.2	711030.0	0.0	0.0	2113.0	394800.4			2113.0	394800.4	1105830.4
MB0321000	PA CASCALHO	1540.4	287688.4	0.0	0.0	1128.5	210793.6	0.0	3.5	1128.5	210797.0	498485.5
MB0322000	PA NOVA VIDA	1912.1	357035.7	0.0	0.0	1076.5	201193.7			1076.5	201193.7	558229.4

MB0323000	PA CASTANHAL RATO II	1357.6	246431.3	0.0	0.0	115.1	20338.5					115.1	20338.5	266769.8	
MB0324000	PA SANTA MARIA	1258.2	235143.5	0.0	0.0	892.5	166803.0			7.4	1383.9	899.9	168186.9	403330.4	
MB0325000	PA SANTA LIDUINA	1113.5	207891.8	0.0	0.0	785.0	146687.1			11.8	2201.5	796.8	148888.6	356780.4	
MB0326000	PA IOLANDA	2385.6	444857.2	0.0	0.0	1048.0	195815.7			4.5	838.7	1052.5	196654.3	641511.5	
MB0327000	PA SÃO JOSE	4266.6	789487.6	0.0	0.0	235.4	43567.3					235.4	43567.3	833054.8	
MB0328000	PA PAU FERRADO	2830.9	513293.5	0.0	0.0	119.1	20261.8					119.1	20261.8	533555.4	
MB0329000	PA BEIJA FLOR II	635.8	118828.1	0.0	0.0	144.9	27083.4			385.3	72017.5	530.3	99100.8	217929.0	
MB0330000	PA AMAPÁ I	1936.0	360196.7	0.0	0.0	819.7	153123.6			607.6	113540.9	1427.3	266664.5	626861.2	
MB0331000	PA ALTO AMAZONAS	1321.2	246778.9	0.0	0.0	1081.9	202129.6			8.2	1537.3	1090.2	203666.9	450445.8	
MB0332000	PA BARRAGEIRA	1263.9	235532.7	0.0	0.0	1391.0	259868.7			319.1	59619.4	1710.2	319488.2	555020.9	
MB0333000	PA CACHOEIRINHA	845.1	157911.5	0.0	0.0	376.9	70432.5			30.6	5717.1	407.5	76149.6	234061.1	
MB0334000	PA AMAPA II	1356.0	252062.2	0.0	0.0	583.1	108883.6			578.5	108113.2	1161.6	216996.8	469059.1	
MB0335000	PA SÃO GERALDO DO REPARTIMENTO	1144.5	213895.1	0.0	0.0	952.7	178046.9			0.1	21.1	952.8	178068.0	391963.1	
MB0336000	PA NOVO MUNDO DO ITUPIRANGA	985.6	183987.1	0.0	0.0	255.4	47729.5					255.4	47729.5	231716.6	
MB0337000	PA BOA ESPERANÇA DO ARAGUAIA	2591.7	479626.8	0.0	0.0	121.1	22419.3					121.1	22419.3	502046.0	
MB0338000	PA UNIÃO	1887.3	351328.8	0.0	0.0	1039.7	194245.8					1039.7	194245.8	545574.6	
MB0339000	PA JERUZALÉM	1898.3	352893.3	0.0	0.0	713.2	133243.6					713.2	133243.6	486136.9	
MB0340000	PA SANTA TEREZINHA	2274.5	386844.3	0.0	0.0	181.9	30187.2					181.9	30187.2	417031.5	13.3
MB0341000	PA SAMAUMA	1407.2	261397.5	0.0	0.0	143.5	26814.5					143.5	26814.5	288212.0	
MB0342000	PA MARRECAS DO ARAGUAIA	3019.4	437596.6	0.0	0.0	445.3	47570.1	120.1	11093.5			565.5	58663.6	496260.2	
MB0343000	PA CUXIÚ	274.7	51337.7	0.0	0.0	220.9	41286.0					220.9	41286.0	92623.7	
MB0344000	PA JOSE PINHEIRO LIMA	3068.6	570913.3	0.0	0.0	920.7	172024.4					920.7	172024.4	742937.7	
MB0345000	PA JURUNA	5976.5	1114136.8	0.0	0.0	6145.4	1147763.0			89.5	16720.1	6234.9	1164483.1	2278619.9	
MB0346000	PA PALMARES SUL	12680.1	2356680.3	0.0	0.0	2926.3	546336.9					2926.3	546336.9	2903017.3	
MB0347000	PA ALCOBAÇA	84440.4	15654471.8	0.0	0.0	15782.4	2933784.0	13.7	2533.7	2694.6	499122.8	18490.7	3435440.4	19089912.3	95.1
MB0351000	PA IGARAPE DO RATO	1967.3	365574.9	0.0	0.0	222.0	41373.8					222.0	41373.8	406948.7	
MB0352000	PA ARAÇATUBA CARAJÁS	1355.4	251248.8	0.0	0.0	60.6	11329.3					60.6	11329.3	262578.1	
MB0353000	PA 4 DE JUNHO	1036.3	192997.2	0.0	0.0	157.7	29469.5					157.7	29469.5	222466.7	

MB0354000	PA JARDIM	1778.4	330945.3	0.0	0.0	451.3	84289.2					451.3	84289.2	415234.5
MB0355000	PA FÊNIX	1431.5	242513.8	0.0	0.0	22.5	3700.1					22.5	3700.1	246213.9
MB0356000	PA JOSÉ DUTRA DA COSTA	2694.7	502666.8	0.0	0.0	187.1	34973.7			44.7	8349.0	231.9	43322.7	545989.5
MB0358000	PA NOSSA SENHORA APARECIDA	3164.6	587768.9	0.0	0.0	240.2	44705.4					240.2	44705.4	632474.4
MB0359000	PA NOVA VITORIA	5493.8	1022711.6	0.0	0.0	373.7	69789.5			98.9	18461.7	472.6	88251.2	1110962.8
MB0360000	PA UNIDOS PARA VENCER	1808.2	337391.3	0.0	0.0	310.1	57933.2					310.1	57933.2	395324.5
MB0361000	PA PIQUIÁ	4216.7	780356.1	0.0	0.0	270.5	50053.5					270.5	50053.5	830409.5
MB0363000	PA PIRASSUNUNGA	297.1	55243.5	0.0	0.0	187.7	34979.0	5.6	989.0			193.4	35968.0	91211.5
MB0364000	PA POUSO ALEGRE	835.3	155807.5	0.0	0.0	298.9	55856.7					298.9	55856.7	211664.3
MB0365000	PA SANTA RITA	887.6	165381.7	0.0	0.0	63.6	11883.8					63.6	11883.8	177265.6
MB0366000	PA POUSO BONITO	873.5	162485.9	0.0	0.0	443.8	82907.8			99.9	18653.1	543.7	101560.9	264046.7
MB0367000	PA BOA SORTE II	572.4	106534.3	0.0	0.0	321.2	59895.1			9.6	1793.0	330.9	61688.1	168222.4
MB0368000	PA SANTA MARTA	899.1	166902.1	0.0	0.0	278.7	51969.7			38.1	7114.0	316.9	59083.7	225985.8
MB0369000	PA 8 DE MARÇO	539.3	100136.2	0.0	0.0	28.5	5326.0			955.6	178511.3	984.1	183837.3	283973.5
MB0370000	PA NOSSA SENHORA DA GUIA	1857.6	345662.2	0.0	0.0	190.2	35507.1			198.5	37029.2	388.6	72536.3	418198.5
MB0371000	PA VALE DO ARATAU	1350.8	251903.4	0.0	0.0	416.8	77863.4			241.3	45077.3	658.0	122940.7	374844.1
MB0372000	PA NOVA UNIÃO	737.8	137330.5	0.0	0.0	20.9	3914.1			638.5	119321.4	659.4	123235.5	260566.0
MB0373000	PA COCALÂNDIA II	1090.9	203729.8	0.0	0.0	556.2	103936.8					556.2	103936.8	307666.6
MB0374000	PA PARAUNA	792.3	148030.4	0.0	0.0	89.2	16664.1			1097.1	205022.9	1186.2	221687.0	369717.4
MB0375000	PA REUNIDAS	2486.1	463973.8	0.0	0.0	650.6	121568.0			1176.0	219758.7	1826.6	341326.7	805300.5
MB0376000	PA PALMEIRA JUSSARA	845.1	157057.0	0.0	0.0	117.0	21813.5					117.0	21813.5	178870.6
MB0377000	PA CONSPEL	807.9	149508.4	0.0	0.0	241.5	44695.2					241.5	44695.2	194203.6
MB0378000	PA JOÃO LANARI DO VAL	3986.3	383668.9	0.0	0.0	680.0	45676.3					680.0	45676.3	429345.2
MB0379000	PA CABANOS	2734.5	462164.5	0.0	0.0	175.6	28674.1					175.6	28674.1	490838.6
MB0380000	PA BELO VALE	1138.4	211905.2	0.0	0.0	157.4	29393.1					157.4	29393.1	241298.3
MB0381000	PA CASTANHAL ALMESCÃO	3906.1	730016.0	0.0	0.0	699.5	130730.0					699.5	130730.0	860746.0
MB0382000	PA NOVO PROJETO	0.0	0.0	0.0	0.0			1716.1	31500.3			1716.1	31500.3	31500.3
MB0384000	PA MATA AZUL II	1965.5	320319.5	0.0	0.0	132.8	21649.9					132.8	21649.9	341969.3

MB0385000	PA IRMÃ DULCE	4182.0	702115.4	0.0	0.0	175.8	29511.8					175.8	29511.8	731627.3	
MB0387000	PA AQUARIUS CRUZEIRO DO SUL	2720.9	506516.7	0.0	0.0	287.1	53609.0			1053.5	196864.1	1340.6	250473.1	756989.8	
MB0388000	PA BELO HORIZONTE II	1854.7	344070.6	0.0	0.0	529.6	98907.4					529.6	98907.4	442978.0	
MB0389000	PA PEDRA DE AMOLAR	703.2	130129.2	0.0	0.0	51.7	9576.3					51.7	9576.3	139705.5	
MB0390000	PA ZUMBI DOS PALMARES	2039.9	379982.3	0.0	0.0	1170.7	218664.9			565.9	105750.0	1736.6	324414.9	704397.2	
MB0391000	PA SOL NASCENTE	1174.7	217398.1	0.0	0.0	39.4	7283.8					39.4	7283.8	224681.9	
MB0392000	PA GABRIEL PIMENTA	2562.0	474803.8	0.0	0.0	599.3	111727.8					599.3	111727.8	586531.5	
MB0394000	PA JOÃO VAZ	2510.1	464456.0	0.0	0.0	138.0	25432.5	123.6	22880.9			261.7	48313.4	512769.4	
MB0395000	PA LIBERDADE	4839.5	903145.4	0.0	0.0	768.7	143654.6					768.7	143654.6	1046800.0	
MB0396000	PA BOM JESUS	1445.9	236316.1	0.0	0.0	76.5	12474.3					76.5	12474.3	248790.4	
MB0397000	PA SANTA LUCIA	1172.7	218957.3	0.0	0.0	79.0	14768.6					79.0	14768.6	233726.0	
MB0398000	PA ESTRELA DALVA	1488.4	278098.3	0.0	0.0	1259.3	235349.8					1259.3	235349.8	513448.1	
MB0401000	PA GROTÃO DO SEVERINO	746.6	138326.1	0.0	0.0	16.4	3029.5					16.4	3029.5	141355.6	
MB0402000	PA TALISMÃ	668.9	124972.7	0.0	0.0	317.6	59348.7					317.6	59348.7	184321.4	
MB0404000	PA CANUDOS	2567.0	456166.2	0.0	0.0	174.6	28630.4					174.6	28630.4	484796.6	
MB0405000	PA TARUMÃ	4023.6	747282.9	0.0	0.0	381.4	70949.7					381.4	70949.7	818232.6	
MB0406000	PA ESPERANÇA	2028.6	346425.1	0.0	0.0	235.5	38633.2					235.5	38633.2	385058.3	
MB0407000	PA LIMEIRA	1205.2	223729.9	0.0	0.0	137.7	25602.2					137.7	25602.2	249332.1	
MB0408000	PA ANTARES	5577.5	1038588.6	0.0	0.0	1454.9	271861.8					1454.9	271861.8	1310450.4	
MB0409000	PA PARAGOMINAS	1553.3	259719.3	0.0	0.0	47.0	7716.7					47.0	7716.7	267436.0	
MB0411000	PA CARUMBÉ	2715.9	447244.9	0.0	0.0	136.4	22226.9					136.4	22226.9	469471.7	
MB0412000	PA UNIÃO DO ANGICO	2619.5	427542.0	0.0	0.0	183.0	29829.0					183.0	29829.0	457371.0	
MB0413000	PA MUTUÃ	3267.9	534215.1	0.0	0.0	331.4	54342.5					331.4	54342.5	588557.7	
MB0416000	PA COCALINHO	0.0	0.0	0.0	0.0			1373.8	12941.5			1373.8	12941.5	12941.5	
MB0417000	PA RAINHA DA PAZ	3011.1	562185.0	0.0	0.0	541.6	101190.1			369.8	69117.2	911.5	170307.3	732492.3	
MB0418000	PA GAVIÃO	8060.7	1502350.7	0.0	0.0	2009.6	375428.7			404.0	75467.2	2413.6	450896.0	1953246.6	
MB0419000	PA MANTENHA	1068.9	199437.6	0.0	0.0	101.7	19007.0					101.7	19007.0	218444.6	
MB0420000	PA CACHOEIRA PRETA II	5349.7	998727.5	0.0	0.0	1775.5	331740.8					1775.5	331740.8	1330468.3	11.3

MB0422000	PA PADRE JOSIMO TAVARES II	845.3	157304.5	0.0	0.0	174.6	32611.8					174.6	32611.8	189916.4	32.9
MB0423000	PA REINO ENCANTADO	1205.9	224624.7	0.0	0.0	83.9	15681.6			1037.1	193756.0	1121.0	209437.6	434062.3	
MB0424000	PA DOMINGOS OLIVEIRA BEZERRA	4930.1	808195.8	0.0	0.0	1896.4	312816.7					1896.4	312816.7	1121012.5	
MB0425000	PA SÃO SEBASTIÃO DO CRISTALINO	3200.6	521926.4	0.0	0.0	1583.8	258113.9					1583.8	258113.9	780040.3	
MB0426000	PA SÃO PEDRO II	3197.8	521144.4	0.0	0.0	2309.5	376373.5					2309.5	376373.5	897517.9	
MB0427000	PA PIONEIRA	1262.1	234584.4	0.0	0.0	419.6	78272.6			354.2	65957.2	773.7	144229.8	378814.2	
MB0428000	PA RIBEIRÃO DAS PEDRAS	1317.0	245597.9	0.0	0.0	428.6	80052.9			270.9	50609.4	699.5	130662.3	376260.2	
MB0430000	PA ARIZONA	11008.3	1826941.8	0.0	0.0	2300.6	387538.5					2300.6	387538.5	2214480.3	57.8
MB0431000	PA CONSOLAÇÃO	0.0	0.0	0.0	0.0			4823.3	233626.0			4823.3	233626.0	233626.0	
MB0432000	PA PANORAMA	3452.1	572398.4	0.0	0.0	130.1	21224.7					130.1	21224.7	593623.1	
MB0433000	PA CRISTO VIVE	4131.2	673495.3	0.0	0.0	2545.5	414837.1	85.4	13914.9			2630.9	428752.0	1102247.3	
MB0434000	PA SERRA GRANDE	3831.6	625174.8	0.0	0.0	2066.8	336822.4					2066.8	336822.4	961997.1	
MB0435000	PA NOVA INDEPENDÊNCIA	2955.3	482571.5	0.0	0.0	3105.5	506114.4					3105.5	506114.4	988686.0	
MB0436000	PA ITAMARATY	5700.5	929162.5	0.0	0.0	1910.5	311346.9					1910.5	311346.9	1240509.4	
MB0437000	PA ÁGUA BOA	704.4	115204.5	0.0	0.0							0.0	0.0	115204.5	
MB0438000	PA SUMAUMA II	3277.2	612197.6	0.0	0.0	1796.4	335674.5					1796.4	335674.5	947872.0	39.1
MB0439000	PA CAMPOS VERDES	69.4	760.8	0.0	0.0	97.7	8223.6	4460.5	46231.2			4558.2	54454.8	55215.6	
MB0440000	PA BOM FUTURO	804.3	149870.1	0.0	0.0	2123.5	396308.7			9.3	1726.5	2132.7	398035.2	547905.3	
MB0441000	PA SUNIL	1329.2	248413.9	0.0	0.0	1027.2	191968.8			1.3	241.6	1028.5	192210.4	440624.3	
MB0442000	PA JORDÃO	1585.5	296310.6	0.0	0.0	1444.2	269898.2					1444.2	269898.2	566208.8	
MB0443000	PA CARAJÁS	2295.5	429014.7	0.0	0.0	2802.9	523841.6					2802.9	523841.6	952856.2	
MB0444000	PA VIDA NOVA	1962.0	365611.4	0.0	0.0	1091.3	203813.5			0.0	1.3	1091.3	203814.8	569426.2	
MB0445000	PA BARRACA DO MEIO	2857.4	530796.8	0.0	0.0	1520.2	283948.1					1520.2	283948.1	814744.9	
MB0446000	PA UXI	5328.3	990516.7	0.0	0.0	2625.5	490290.6					2625.5	490290.6	1480807.3	
MB0448000	PA PEDRA BRANCA	13488.7	2508608.1	0.0	0.0	5162.1	964454.1					5162.1	964454.1	3473062.3	
MB0450000	PA RIO BANDEIRA	2026.7	377131.0	0.0	0.0	8800.5	1643515.6			320.7	59924.5	9121.3	1703440.2	2080571.2	
MB0451000	PA SANTIAGO	1639.8	306470.0	0.0	0.0	1765.0	329867.7			313.9	58671.1	2079.0	388538.8	695008.8	
MB0452000	PA NOVA DESCOBERTA	3160.5	583957.4	0.0	0.0	93.8	17276.5					93.8	17276.5	601233.9	

MB0453000	PA LIMPEZA	3690.9	683043.9	0.0	0.0	83.5	15446.8					83.5	15446.8	698490.7	
MB0454000	PA MAL ARRUMADO	949.5	175711.3	0.0	0.0	40.8	7545.2					40.8	7545.2	183256.4	
MB0455000	PA JOSÉ INOCÊNCIO NERES	65.5	8113.4	0.0	0.0	148.1	12155.2	3788.6	48084.6			3936.7	60239.8	68353.2	
MB0456000	PA ÁGUA BRANCA	2328.4	434471.9	0.0	0.0	905.1	169103.6					905.1	169103.6	603575.5	
MB0458000	PA COLÔNIA PODEROSA	5436.1	886028.3	0.0	0.0	1627.2	265185.8	422.6	68872.1			2049.8	334057.9	1220086.2	
MB0459000	PA OESTE	4369.9	815144.7	0.0	0.0	1450.6	271061.9					1450.6	271061.9	1086206.7	
MB0460000	PA RIO CURURUÍ	5865.0	1096112.3	0.0	0.0	22976.8	4294126.4			2570.6	480426.9	25547.4	4774553.3	5870665.6	
MB0461000	PA PETRONÍLIO ALVES BATISTA	5609.1	914122.6	0.0	0.0	8943.2	1457479.2					8943.2	1457479.2	2371601.8	
MB0462000	PA NOVA JERUZALÉM	577.1	106791.5	0.0	0.0							0.0	0.0	106791.5	
MB0463000	PA SÃO VICENTE	2494.9	465474.9	0.0	0.0	250.5	46819.8			2800.8	523422.5	3051.3	570242.3	1035717.2	
MB0467000	PA HAMILTON CORDEIRO CELSO	9429.4	1539635.2	0.0	0.0	4454.3	729176.3	55.8	9100.9			4510.1	738277.2	2277912.4	
MB0469000	PA CENTRO NOVO	2545.2	471010.7	0.0	0.0	164.2	30383.9					164.2	30383.9	501394.6	
MB0472000	PA BACURIZINHO	1133.7	211885.6	0.0	0.0	242.0	45233.0					242.0	45233.0	257118.6	
MB0473000	PA SOL NASCENTE	1494.8	277727.3	0.0	0.0	1434.7	267336.3			33.7	6274.2	1468.4	273610.5	551337.7	
MB0474000	PA CLÉZINHO	3413.8	634314.1	0.0	0.0	1251.0	233735.4					1251.0	233735.4	868049.4	
MB0475000	PA NOSSA SENHORA APARECIDA II	95.2	2544.5	0.0	0.0	117.0	8462.0	1925.9	47675.4			2042.9	56137.4	58681.9	
MB0476000	PA TIRADENTES	199.3	27481.9	0.0	0.0	73.9	11783.1	4032.9	69188.2			4106.8	80971.3	108453.2	
MB0477000	PA ARARANDEUA	13137.9	2444125.0	0.0	0.0	13284.7	2482416.3			968.0	180870.9	14252.7	2663287.2	5107412.2	
MB0478000	PA IRMÃ DOROTY	361.1	67483.9	0.0	0.0	2603.9	486646.5			127.8	23876.6	2731.7	510523.1	578007.0	
MB0480000	PA COSME E DAMIÃO	1435.1	243241.4	0.0	0.0	112.2	18302.1					112.2	18302.1	261543.4	
MB0481000	PA SANTA CECILIA	426.3	79252.1	0.0	0.0	507.7	94653.4					507.7	94653.4	173905.5	
MB0483000	PA CENTRO NOVO II	3514.0	650308.7	0.0	0.0	100.0	18500.8					100.0	18500.8	668809.5	
MB0484000	PA SANTA ELIZA	3344.7	556289.3	0.0	0.0	1.4	234.8					1.4	234.8	556524.1	
MB0485000	PA MORADA NOVA	9421.4	1754459.0	0.0	0.0	629.9	117670.8			4760.9	889459.0	5390.8	1007129.8	2761588.8	
MB0486000	PA MORADA NOVA II	6006.6	1119639.0	0.0	0.0	817.7	152744.8			4622.3	863666.6	5440.0	1016411.4	2136050.4	
MB0489000	PA DEUS É PAZ	6921.1	1122851.3	0.0	0.0	848.8	138055.7					848.8	138055.7	1260907.1	
MB0491000	PA POMBAL	36892.9	6161669.1	0.0	0.0	49355.8	8373677.3	4320.9	550158.8			53676.7	8923836.1	15085505.2	242.0
MB0492000	PA FELICIDADE	5003.6	934817.2	0.0	0.0	282.1	52719.3					282.1	52719.3	987536.5	

MB0493000	PA 1║ DE MAIO	2380.8	444944.3	0.0	0.0	490.0	91576.2					490.0	91576.2	536520.5	
MB0497000	PA RECANTO DA IARA	218.4	10187.0	0.0	0.0	159.0	25387.9	1393.0	24748.9			1552.0	50136.7	60323.8	
MB0499000	PA ANTONIO NONATO	2338.1	435601.1	0.0	0.0	2182.8	407765.9			53.1	9921.2	2235.9	417687.1	853288.2	
MB0500000	PA UNIÃO DA VITÓRIA	599.7	111512.4	0.0	0.0	412.2	77012.8					412.2	77012.8	188525.2	
MB0503000	PA PROGRESSO	1361.6	225580.0	0.0	0.0	97.1	15867.0					97.1	15867.0	241447.0	
MB0504000	PA BARRA MANSA	7420.0	1386728.4	0.0	0.0	8302.1	1551579.5			0.0	2.0	8302.1	1551581.5	2938309.9	
MB0505000	PA 26 DE MARÇO	8222.4	1524279.8	0.0	0.0	1959.2	365262.6					1959.2	365262.6	1889542.5	
MB0506000	PA MAMUI	1375.4	255873.7	0.0	0.0	628.7	117371.9					628.7	117371.9	373245.7	
MB0508000	PA UNIAO BATENTE	3422.8	571221.1	0.0	0.0	507.8	82818.3					507.8	82818.3	654039.4	
MB0509000	PA PRATA	1615.6	298977.0	0.0	0.0	1172.6	217007.9					1172.6	217007.9	515984.9	
MB0511000	PE BACABAL GRANDE	2214.3	410035.0	0.0	0.0	199.4	37076.6					199.4	37076.6	447111.6	
MB0513000	PA BAGUA	4823.5	847169.8	0.0	0.0	699.0	114045.9					699.0	114045.9	961215.7	
MB0514000	PA AGUAS CLARAS	0.0	0.0	0.0	0.0			1453.0	67154.0			1453.0	67154.0	67154.0	
MB0515000	PA SANTA MARIANA	1586.4	263606.5	0.0	0.0	242.5	39590.0					242.5	39590.0	303196.5	
MB0516000	PA SANTA PAULA	2794.7	519918.4	0.0	0.0	1836.7	343129.3			1126.3	210454.0	2963.0	553583.4	1073501.8	10.8
MB0517000	PA SAO SEBASTIAO	1184.5	220013.2	0.0	0.0	1232.2	230234.9			409.4	76488.3	1641.6	306723.3	526736.5	
MB0518000	PA PEDRO ALCANTARA	4625.7	762234.5	0.0	0.0	587.6	95759.0					587.6	95759.0	857993.5	
MB0519000	PA BELAUTO	16686.7	3089354.0	0.0	0.0	8540.0	1594842.9					8540.0	1594842.9	4684196.9	
MB0520000	PA RAINHA II	1946.8	361726.2	0.0	0.0	662.9	123815.8			0.0	4.1	662.9	123819.9	485546.1	
MB0520001	PA SALVADOR ALLENDE PIRATININGA	2396.4	444824.5	0.0	0.0	4448.3	829461.9			420.1	78333.3	4868.4	907795.1	1352619.6	
MB0524000	PA PIROSCA	4039.4	640331.4	0.0	0.0	202.9	33062.9					202.9	33062.9	673394.3	
MB0525000	PA PEDRO LAURINDO DA SILVA	2086.1	388342.0	0.0	0.0	1396.7	260819.8					1396.7	260819.8	649161.8	
PA0006000	PA MIRITIPITANGA	3189.5	591355.4	0.0	0.0	175.5	32587.1			21.0	3906.1	196.6	36493.2	627848.6	
PA0016000	PA MANOEL CRESCÊNCIO DE SOUZA	18322.2	3390700.0	0.0	0.0	524.4	97045.9			22.4	4147.2	546.8	101193.0	3491893.0	
PA0017000	PA COLONIA REUNIDAS	4161.0	771105.9	0.0	0.0	197.8	36822.8			3.6	667.4	201.4	37490.2	808596.1	
PA0019000	PA CONCREIN	4004.8	743147.1	0.0	0.0	67.5	12621.2			11.5	2154.0	79.1	14775.3	757922.3	

PA0027000	PA UNIÃO I	13490.9	2496633.3	0.0	0.0	141.3	26149.2			2.9	533.8	144.2	26683.0	2523316.3	
PA0028000	PA SAO PEDRO	9809.8	1815395.5	0.0	0.0	0.7	130.9					0.7	130.9	1815526.4	
PA0030000	PA BENEDITO ALVES BANDEIRA	6097.8	1128514.0	0.0	0.0	1911.7	353788.3			295.8	54742.7	2207.6	408530.9	1537045.0	
PA0055000	PA BARCELONA	1619.3	299663.1	0.0	0.0							0.0	0.0	299663.1	
PA0063000	PA PARAGOMINAS/FAISCÃO	11693.1	2151265.7	0.0	0.0	1117.9	207340.5			145.4	26837.2	1263.2	234177.6	2385443.3	
PA0066000	PA SANTA MARIA I E II	2323.6	425016.2	0.0	0.0	2003.3	356903.4	74.7	13688.6	50.5	8922.2	2128.4	379514.2	804530.4	
PA0068000	PA JOAO BATISTA	3193.9	591059.7	0.0	0.0	1.1	199.9					1.1	199.9	591259.6	
PA0069000	PA SURUBIJU	3431.4	602413.1	0.0	0.0	739.0	122241.3			184.6	30515.1	923.6	152756.4	755169.5	
PA0080000	PA BOM JESUS	7597.3	1405959.3	0.0	0.0	578.0	106972.2			19.7	3651.5	597.8	110623.8	1516583.1	
PA0081000	PA CIDAPAR 1¬ PARTE	148317.7	27469021.3	0.0	0.0	39098.1	7269454.2			69010.2	12823592.9	108108.3	20093047.1	47562068.4	573.0
PA0082000	PA CIDAPAR 3¬ PARTE	44584.7	8244621.8	0.0	0.0	3083.0	569272.7			5990.2	1110176.2	9073.2	1679448.9	9924070.7	166.3
PA0085000	PA ITABOCAL	39337.2	7279751.1	0.0	0.0	223.4	41349.0			1668.3	308736.7	1891.7	350085.7	7629836.8	
PA0088000	PA CIDAPAR 2¬ PARTE	19067.6	3530129.2	0.0	0.0	444.9	82405.6			2158.9	400365.5	2603.8	482771.1	4012900.3	
PA0097000	PA TROPICÁLIA	3098.0	574036.7	0.0	0.0	1152.0	214314.0			146.4	27227.2	1298.4	241541.3	815577.9	
PA0105000	PA MINAS PARÁ	9835.9	1820576.8	0.0	0.0	424.2	78593.4			49.0	9089.4	473.1	87682.8	1908259.5	
PA0150000	PA ARAPUA SIMEIRA	58073.9	10758768.6	0.0	0.0	2255.4	418703.6			8771.5	1630567.4	11026.9	2049270.9	12808039.5	
PA0154000	PA CAMAPUÃ	5541.7	1026682.8	0.0	0.0	1443.6	269004.4			350.0	65249.5	1793.6	334253.8	1360936.6	
PA0163000	PA MARAVILHA	7291.1	1351470.3	0.0	0.0	1031.1	192016.0	50.8	9462.5	60.7	11308.4	1142.5	212786.8	1564257.2	
PA0164000	PA FLORESTA GURUPI I	32600.7	5832186.4	0.0	0.0	6528.7	1152360.8			2965.0	520623.8	9493.7	1672984.7	7505171.1	0.7
PA0165000	PA VALE DO BACABA	13822.9	2558471.4	0.0	0.0	93.1	17229.5			0.0	0.5	93.1	17229.9	2575701.3	
PA0166000	PA DEL REY	7278.3	1348037.0	0.0	0.0	1293.7	240379.1			136.9	25456.6	1430.6	265835.7	1613872.7	
PA0167000	PA SERRA NEGRA	2435.2	450849.9	0.0	0.0	80.0	14809.2			8.0	1496.9	88.0	16306.1	467156.0	
PA0168000	PA CANDIRU	9067.3	1677995.6	0.0	0.0	9.4	1748.1					9.4	1748.1	1679743.7	
PA0171000	PA ÁGUIA	7469.8	1389673.4	0.0	0.0	574.4	107316.7			436.7	81588.0	1011.1	188904.7	1578578.1	
PA0179000	PA PARAGONORTE	28414.9	5265494.7	0.0	0.0	2621.4	487925.3			1414.5	263577.2	4036.0	751502.6	6016997.2	
PA0180000	PA RIO JABUTI	15200.5	2815879.2	0.0	0.0	669.3	123151.8			21.6	3985.2	690.9	127136.9	2943016.2	
PA0193000	PA PARANOA	14626.3	2712031.3	0.0	0.0	1672.8	311517.1			1164.6	216923.9	2837.5	528441.1	3240472.4	

PA0194000	PA ÁGUA AZUL	3193.4	592095.3	0.0	0.0	97.5	18126.3			832.1	155080.4	929.6	173206.7	765302.0	
PA0210000	PA CALMARIA I	5139.4	958394.0	0.0	0.0	3917.1	730465.5			236.0	44016.1	4153.2	774481.7	1732875.7	
PA0211000	PA CALMARIA II	7248.7	1348540.8	0.0	0.0	3764.1	701111.9	1466.8	271683.8	395.4	73664.9	5626.3	1046460.7	2395001.5	
PA0212000	PA OLHO D AGUA I	5728.5	1060792.2	0.0	0.0	1541.2	285695.6	2352.4	435672.1	27.7	5129.2	3921.3	726496.9	1787289.1	
PA0224000	PA LUIZ LOPES SOBRINHO	3447.2	637939.4	0.0	0.0	982.7	181851.5			92.1	17052.5	1074.8	198904.0	836843.4	
PA0225000	PA CRISTAL	1362.7	252174.3	0.0	0.0	22.6	4183.9					22.6	4183.9	256358.2	
PA0229000	PA ENALCO	10058.1	1871099.3	0.0	0.0	1208.7	225193.7			7.5	1402.3	1216.3	226595.9	2097695.2	0.4
PA0230000	PA IMPERASSU	1910.1	353484.8	0.0	0.0	17.8	3295.6					17.8	3295.6	356780.4	
PA0248000	PA CUPIÚBA	1350.6	249945.8	0.0	0.0							0.0	0.0	249945.8	
PA0250000	PA JOÃO BATISTA II	1825.7	333881.7	0.0	0.0	289.7	51941.4					289.7	51941.4	385823.1	
PA0251000	PA CANAÃ	3805.3	704459.5	0.0	0.0	116.0	21464.0					116.0	21464.0	725923.5	
PA0253000	PA TRÊS IRMÃOS	2411.7	448779.0	0.0	0.0	1003.5	187050.8			57.3	10685.5	1060.9	197736.3	646515.2	
PA0254000	PA TIMBORANA	3109.2	577486.9	0.0	0.0	133.0	24775.1					133.0	24775.1	602262.0	
PA0255000	PA RIO ACAMPAMENTO	5900.8	1099819.5	0.0	0.0	578.5	108093.0					578.5	108093.0	1207912.5	
PA0256000	PA NOVA CONQUISTA	1342.5	249163.1	0.0	0.0	55.6	10372.0					55.6	10372.0	259535.2	
PA0257000	PA OLHO D ÁGUA II	2292.7	427543.7	0.0	0.0	4769.7	889449.1			980.7	182871.8	5750.3	1072320.9	1499864.6	63.5
PA0258000	PA RIO DAS CRUZES	3375.2	630096.6	0.0	0.0	421.2	78721.7			107.7	20126.8	528.9	98848.5	728945.1	
PA0259000	PA ESPERANÇA	4238.1	784501.0	0.0	0.0	1070.0	198081.0			286.2	52981.8	1356.2	251062.8	1035563.7	
PA0261000	PCA MÁRTIRES DE ABRIL	283.9	49686.9	0.0	0.0	124.4	21762.8					124.4	21762.8	71449.7	
PA0267000	PA PIRAMIDE	184.2	34094.9	0.0	0.0	53.5	9902.1			1.9	357.4	55.4	10259.5	44354.4	
PA0268000	PA BACABAL	2036.5	377729.5	0.0	0.0	254.5	47338.0			7.9	1466.7	262.4	48804.7	426534.2	
PA0269000	PA PROGRESSO	2811.8	523548.9	0.0	0.0	879.4	163894.1			36.8	6861.2	916.2	170755.3	694304.2	
PA0270000	PA NOVA SANTA MARIA	736.5	136296.4	0.0	0.0	22.2	4116.3			144.3	26701.0	166.5	30817.3	167113.7	
PA0271000	PA TAPERUSSU	3316.3	610656.1	0.0	0.0	793.1	145529.5			54.3	9906.6	847.3	155436.1	766092.2	
PA0272000	PA VALE DO MOJU	12523.4	2334791.5	0.0	0.0	7245.4	1351089.9	243.8	45462.8	515.9	96209.2	8005.1	1492762.0	3827553.5	80.3
PA0273000	PA INÁCIA	2205.6	408166.6	0.0	0.0	314.3	58159.8			138.2	25572.8	452.5	83732.6	491899.2	
PA0274000	PA JARARACA	1142.1	211351.2	0.0	0.0	87.1	16110.3			151.5	28042.8	238.6	44153.1	255504.2	
PA0275000	PAE SÃO JOÃO BATISTA	0.0	0.0	0.0	0.0	390.8	68382.8					390.8	68382.8	68382.8	41.1

PA0276000	PAE NOSSA SENHORA DO LIVRAMENTO	0.0	0.0	0.0	0.0	180.6	31604.4					180.6	31604.4	31604.4	23.2
PA0278000	PCA ELIZABETE TEIXEIRA	27.9	4876.1	0.0	0.0	43.8	7662.2					43.8	7662.2	12538.3	
PA0286000	PA MANDACARU	3856.3	716382.6	0.0	0.0	486.4	90540.0			44.9	8368.3	531.3	98908.3	815290.9	
PA0287000	PA DIAMANTINA II	1931.7	357937.5	0.0	0.0	324.8	60509.3			4.4	811.4	329.2	61320.8	419258.2	
PA0288000	PA ALTA FLORESTA	2413.9	449296.0	0.0	0.0	1309.2	244101.5			294.5	54914.1	1603.7	299015.6	748311.5	
PA0289000	PA TERRA NOVA	5109.4	943284.4	0.0	0.0	611.8	113014.2			14.2	2580.3	626.0	115594.5	1058878.9	89.3
PA0290000	PA SUÇUARANA	3760.1	701587.0	0.0	0.0	461.4	86238.8					461.4	86238.8	787825.8	
PA0291000	PAE ILHA DAS ONÇAS	43.5	7605.5	0.0	0.0	7304.7	1278315.6	22.3	3894.1			7326.9	1282209.7	1289815.2	367.6
PA0292000	PAE JOÃO PILATOS	290.5	50839.6	0.0	0.0	3372.0	590091.8					3372.0	590091.8	640931.4	90.9
PA0293000	PAE SANTO ANTONIO	0.0	0.0	0.0	0.0	1316.7	230414.1					1316.7	230414.1	230414.1	13.6
PA0294000	PAE NOSSA SENHORA DE NAZARÉ	18.0	3146.9	0.0	0.0	923.9	161682.1			30.7	5371.7	954.6	167053.7	170200.7	25.9
PA0295000	PAE NOSSA SENHORA DO PERPETUO SOCORRO	167.4	29302.1	0.0	0.0	2515.1	440148.4			45.6	7973.4	2560.7	448121.9	477424.0	19.2
PA0296000	PAE ILHA SANTA ROSA	12.6	2207.8	0.0	0.0	353.6	61871.4					353.6	61871.4	64079.2	18.5
PA0298000	PAE ILHA VIÇOSA	0.0	0.0	0.0	0.0	327.0	57233.2					327.0	57233.2	57233.2	23.0
PA0299000	PAE ILHA ARAPIRANGA	142.4	24922.9	0.0	0.0	3325.5	581959.6	7.5	1318.9			3333.0	583278.5	608201.5	148.2
PA0300000	PAE SANTA MARIA	0.0	0.0	0.0	0.0	674.8	118095.2					674.8	118095.2	118095.2	55.4
PA0301000	PAE SANTO AFONSO	1051.3	183982.5	0.0	0.0	1495.8	261757.7	13.0	2266.8	11.6	2021.4	1520.3	266045.8	450028.3	123.3
PA0302000	PAE NOSSA SENHORA DAS GRAÇAS	222.9	39006.8	0.0	0.0	1584.0	277199.1			47.3	8275.0	1631.3	285474.1	324480.9	40.7
PA0303000	PAE SANTO ANTONIO II	209.5	36667.9	0.0	0.0	662.7	115975.0	10.8	1888.9			673.5	117863.9	154531.8	338.6
PA0304000	PAE SÃO FRANCISCO DE ASSIS	0.0	0.0	0.0	0.0	431.5	75506.8	6.1	1070.5			437.6	76577.3	76577.3	41.5
PA0305000	PAE SÃO RAIMUNDO	551.6	96532.2	0.0	0.0	1910.1	334269.5			16.7	2917.6	1926.8	337187.2	433719.3	106.7
PA0306000	PA PAULO FONTELES	297.7	52094.8	0.0	0.0	547.1	95748.6					547.1	95748.6	147843.4	
PA0307000	PA ALTO BONITO	2034.9	378861.7	0.0	0.0	317.2	59269.3			75.4	14082.1	392.6	73351.4	452213.0	
PA0308000	PA SANTA PAULA I	352.5	65228.4	0.0	0.0							0.0	0.0	65228.4	
PA0309000	PAE ILHA MAMANGAL	61.9	10833.0	0.0	0.0	2423.2	424056.7					2423.2	424056.7	434889.7	71.0

PA0310000	PAE ILHA URUBUOCA	0.0	0.0	0.0	0.0	311.5	54517.4					311.5	54517.4	54517.4	25.1
PA0311000	PAE ILHA LONGA	0.0	0.0	0.0	0.0	158.6	27761.0					158.6	27761.0	27761.0	19.7
PA0312000	PAE ILHA SUMAUMA	198.9	34810.1	0.0	0.0	4059.9	710490.8					4059.9	710490.8	745301.0	331.9
PA0313000	PAE ILHA GRANDE - PACAJAI	7669.3	1421561.9	0.0	0.0	12641.9	2353659.8	734.3	136579.0	14194.4	2644247.0	27570.6	5134485.9	6556047.8	1206.0
PA0314000	PAE ILHA BUÇU	17.9	3137.5	0.0	0.0	331.1	57948.4					331.1	57948.4	61085.9	110.6
PA0315000	PAE ILHA MUCURA	0.0	0.0	0.0	0.0	393.7	68904.7					393.7	68904.7	68904.7	38.4
PA0316000	PAE ILHA ARAPARI	41.3	7227.9	0.0	0.0	2686.8	470197.3	10.0	1758.7	27.9	4874.1	2724.7	476830.1	484058.0	103.1
PA0317000	PAE ILHA MOCAJUBA	81.1	14186.2	0.0	0.0	37.7	6597.4			17.8	3110.6	55.5	9708.0	23894.2	23.4
PA0318000	PA NOVO JAUARA	1224.7	226641.3	0.0	0.0	206.9	38294.1					206.9	38294.1	264935.4	28.9
PA0319000	PA ARAXITEUA	1172.8	216811.8	0.0	0.0	293.6	54111.5					293.6	54111.5	270923.3	
PA0320000	PA FLOR DE MINAS	2323.4	429969.7	0.0	0.0	276.1	51092.2			3.9	725.5	280.0	51817.6	481787.3	
PA0321000	PAE ILHA SÃO MATEUS	26.6	4647.5	0.0	0.0	1658.2	290183.4					1658.2	290183.4	294830.9	34.9
PA0322000	PAE ILHA GRANDE CAMETÁ	2.4	424.4	0.0	0.0	6691.8	1171065.8					6691.8	1171065.8	1171490.2	712.1
PA0323000	PAE NOSSA SENHORA DA PAZ	847.9	148375.7	0.0	0.0	1315.5	230218.7					1315.5	230218.7	378594.4	18.2
PA0324000	PAE SÃO JOÃO BATISTA II	749.0	131083.3	0.0	0.0	2430.8	425398.6					2430.8	425398.6	556481.9	56.0
PA0325000	PAE ILHA UMARITUBA	0.0	0.0	0.0	0.0	707.7	131967.3			0.1	12.9	707.7	131980.2	131980.2	35.2
PA0326000	PAE ILHA DO PAULO	0.0	0.0	0.0	0.0	346.3	60610.8					346.3	60610.8	60610.8	41.5
PA0327000	PAE ILHA DO TESO	0.0	0.0	0.0	0.0	1018.7	178267.5			1.1	192.6	1019.8	178460.1	178460.1	32.8
PA0328000	PAE ILHA RAQUEL	12.6	2350.0	0.0	0.0	1577.0	294088.0					1577.0	294088.0	296438.1	27.8
PA0329000	PAE ILHA CAETÉ	0.0	0.0	0.0	0.0	1133.4	211350.2					1133.4	211350.2	211350.2	8.4
PA0330000	PAE ILHA CUXIPIARI	0.0	0.0	0.0	0.0	3498.1	612162.6					3498.1	612162.6	612162.6	176.9
PA0331000	PAE ILHA JACARÉ XINGU	0.0	0.0	0.0	0.0	861.4	150749.0	1.7	299.5			863.1	151048.5	151048.5	30.9
PA0332000	PAE ILHA JAITUBA	0.0	0.0	0.0	0.0	673.0	117767.3					673.0	117767.3	117767.3	216.9
PA0333000	PAE ILHA JOROCA	4.7	819.0	0.0	0.0	1933.7	338406.0					1933.7	338406.0	339225.0	253.4
PA0334000	PAE ILHA MANOEL RAIMUNDO	0.0	0.0	0.0	0.0	1066.2	186586.6					1066.2	186586.6	186586.6	74.2
PA0335000	PAE ILHA DO CARMO	0.0	0.0	0.0	0.0	75.5	13210.1					75.5	13210.1	13210.1	0.3
PA0337000	PAE ILHA GAMA	0.0	0.0	0.0	0.0	89.1	15590.5					89.1	15590.5	15590.5	39.3
PA0338000	PAE ILHA GUAJARÁ	0.0	0.0	0.0	0.0	392.6	73211.6					392.6	73211.6	73211.6	19.4

PA0339000	PAE ILHA CACOAL	0.0	0.0	0.0	0.0	330.8	57891.5					330.8	57891.5	57891.5	98.6
PA0340000	PAE ILHA JARACUERA	0.0	0.0	0.0	0.0	572.8	100240.9					572.8	100240.9	100240.9	59.3
PA0341000	PAE ILHA MAPEUÁ	0.0	0.0	0.0	0.0	355.0	62121.3					355.0	62121.3	62121.3	27.8
PA0342000	PAE ILHA MOIRABA	0.0	0.0	0.0	0.0	229.9	40235.1					229.9	40235.1	40235.1	8.0
PA0343000	PAE ILHA TABATINGA DO CARAPAJÓ	0.0	0.0	0.0	0.0	420.5	74404.7			6.8	1233.1	427.3	75637.8	75637.8	74.3
PA0344000	PAE ILHA PARURU	0.0	0.0	0.0	0.0	3585.1	627393.3			139.3	24380.2	3724.4	651773.6	651773.6	90.6
PA0345000	PAE ILHA CARIPETUBA	1061.5	185763.9	0.0	0.0	2584.2	452228.3	1.1	188.9	25.3	4434.7	2610.6	456851.9	642615.8	102.6
PA0346000	PAE ILHA URUÁ I	62.8	10987.1	0.0	0.0	1516.3	265354.9					1516.3	265354.9	276342.1	44.7
PA0347000	PAE ILHA PIQUIARANA	145.1	25395.2	0.0	0.0	623.4	109093.6					623.4	109093.6	134488.8	16.6
PA0348000	PAE ILHA XIPAIÁ	195.9	36172.5	0.0	0.0	997.3	182330.4	23.9	1380.0	18.5	3415.9	1039.7	187126.3	223298.8	24.5
PA0349000	PAE ILHA GRANDE BELÉM	0.0	0.0	0.0	0.0	745.7	130504.2	103.4	18094.7			849.1	148598.8	148598.8	34.4
PA0350000	PAE ILHA JUTUBA	0.0	0.0	0.0	0.0	453.2	79305.4					453.2	79305.4	79305.4	36.5
PA0351000	PAE ILHA MURUTUCU	0.0	0.0	0.0	0.0	826.9	144711.4					826.9	144711.4	144711.4	22.2
PA0352000	PAE ILHA PAQUETÁ	0.0	0.0	0.0	0.0	720.6	126108.3					720.6	126108.3	126108.3	45.2
PA0353000	PAE ILHA SANTANA	1039.3	192338.9	0.0	0.0	3442.1	636991.7			31.4	5802.4	3473.4	642794.1	835133.0	148.9
PA0354000	PAE ILHA JUPATITUBA	0.0	0.0	0.0	0.0	21.7	3794.1					21.7	3794.1	3794.1	24.8
PA0355000	PAE ILHA URUÁ	0.0	0.0	0.0	0.0	408.4	71469.5			24.4	4271.7	432.8	75741.2	75741.2	91.4
PA0356000	PAE ILHA SANTARÉM	0.0	0.0	0.0	0.0	4113.7	719890.3			25.6	4481.1	4139.3	724371.4	724371.4	95.7
PA0357000	PAE ILHA CASTANHAL	0.0	0.0	0.0	0.0	1114.1	194962.1			119.0	20820.9	1233.0	215783.0	215783.0	122.8
PA0358000	PAE ILHA UNIÃO	0.0	0.0	0.0	0.0	614.7	107569.4					614.7	107569.4	107569.4	74.6
PA0359000	PAE ILHA DAS CINZAS	0.0	0.0	0.0	0.0	1922.5	336440.3			995.7	174243.1	2918.2	510683.4	510683.4	382.6
PA0360000	PAE ILHA PALHETA	13.2	2314.9	0.0	0.0	373.4	65345.8					373.4	65345.8	67660.7	24.6
PA0361000	PAE ILHA DAS PRACUUBINHAS	3.2	567.4	0.0	0.0	356.4	62378.4			447.7	78340.1	804.1	140718.4	141285.9	
PA0362000	PAE ILHA CHIQUEIRO	0.0	0.0	0.0	0.0	2464.2	431239.7	9.4	1637.8	5.5	963.5	2479.1	433841.0	433841.0	113.3
PA0363000	PAE ILHA CAMPUMPEMA	0.0	0.0	0.0	0.0	204.2	35736.4			0.0	1.4	204.2	35737.8	35737.8	88.5
PA0364000	PAE ILHA URUTAÍ PARTE	0.0	0.0	0.0	0.0	5239.3	916873.6			5292.4	926173.4	10531.7	1843047.0	1843047.0	780.1
PA0365000	PAE ILHA MURUMURU	0.0	0.0	0.0	0.0	1431.1	250449.5			2.7	479.8	1433.9	250929.2	250929.2	204.4

PA0366000	PAE ILHA GOIABAL	0.0	0.0	0.0	0.0	1087.4	190303.0			13.3	2328.4	1100.8	192631.4	192631.4	68.4
PA0367000	PAE ILHA BACURI	0.0	0.0	0.0	0.0	2032.9	355755.8			18.1	3168.1	2051.0	358923.9	358923.9	81.5
PA0368000	PAE ILHA SANTO AMARO	0.0	0.0	0.0	0.0	2368.7	414523.6			1717.6	300573.6	4086.3	715097.2	715097.2	302.4
PA0369000	PAE ILHA PIQUIARANA MIRIM	56.7	9927.8	0.0	0.0	488.5	85495.7					488.5	85495.7	95423.5	28.6
PA0370000	PA PALHETA	6058.4	1121163.6	0.0	0.0	142.4	26345.8			436.9	80847.9	579.2	107193.7	1228357.3	
PA0371000	PAE ILHA SACAJÓS GRANDE	0.0	0.0	0.0	0.0	3222.8	563992.9	9.2	1608.2			3232.0	565601.0	565601.0	240.1
PA0372000	PAE ILHA PRAIA GRANDE	64.8	11915.3	0.0	0.0	4351.4	773019.7	727.2	135275.8			5078.7	908295.5	920210.8	300.7
PA0373000	PAE ILHA MURUJUCÁ MIRI	6.4	1126.1	0.0	0.0	1925.7	339264.3	51.1	9529.8	183.3	32079.2	2160.1	380873.3	381999.4	101.0
PA0374000	PAE ILHA ATURIÁ GRANDE	0.0	0.0	0.0	0.0	405.1	70888.0					405.1	70888.0	70888.0	67.6
PA0375000	PAE ILHA DAMIÃO	0.0	0.0	0.0	0.0	3810.6	669622.2	573.8	106906.9			4384.4	776529.0	776529.0	195.1
PA0376000	PAE ILHA CAÍ GRANDE	0.0	0.0	0.0	0.0	558.5	97736.8					558.5	97736.8	97736.8	86.0
PA0377000	PAE ILHA JARIMBU	146.7	25666.2	0.0	0.0	8201.5	1457064.0					8201.5	1457064.0	1482730.2	251.9
PA0378000	PAE ILHA PANACAUERA-MIRI	147.5	25807.7	0.0	0.0	3791.9	663591.2			2.4	422.4	3794.4	664013.6	689821.3	164.3
PA0379000	PAE ILHA DO CUMBU	3.6	628.2	0.0	0.0	1362.9	238504.0			50.4	8821.1	1413.3	247325.2	247953.4	79.7
PA0380000	PAE ILHA PAULISTA	1.4	253.7	0.0	0.0	1563.2	273562.8					1563.2	273562.8	273816.6	55.1
PA0381000	PAE ILHA CONCEIÇÃO	0.0	0.0	0.0	0.0	4711.8	824563.8					4711.8	824563.8	824563.8	67.4
PA0382000	PAE ILHA MUTIRÃO	216.6	37905.3	0.0	0.0	1446.2	253084.9					1446.2	253084.9	290990.2	29.6
PA0383000	PA AREIA BRANCA	1253.4	232517.8	0.0	0.0	165.7	30875.8			5.8	1088.2	171.5	31964.1	264481.9	
PA0384000	PA LUIZ INÁCIO	20590.1	3825885.8	0.0	0.0	9740.9	1814753.7			4612.1	858074.9	14353.0	2672828.6	6498714.4	
PA0385000	PAE ILHA COMPRIDA	0.0	0.0	0.0	0.0	15.4	2692.2					15.4	2692.2	2692.2	17.0
PA0386000	PAE ILHA MAUBA	11.8	2059.7	0.0	0.0	807.0	141232.1			14.8	2590.4	821.8	143822.5	145882.2	58.6
PA0388000	PAE COMPLEXO MARACUJÁ	14.7	2575.5	0.0	0.0	731.3	127969.0			14.0	2455.1	745.3	130424.1	132999.6	30.0
PA0389000	PAE ILHA ANUERÁ	3.6	630.3	0.0	0.0	210.8	36881.8					210.8	36881.8	37512.1	82.7
PA0390000	PAE ILHA DE MELGAÇO	1338.5	240629.5	0.0	0.0	5762.4	1037996.2	1945.6	352869.0	4677.8	844379.4	12385.7	2235244.6	2475874.1	33.4
PA0391000	PAE ILHA MUJIRUM	187.2	34546.8	0.0	0.0	7105.3	1270518.6			7878.7	1413722.6	14984.0	2684241.2	2718788.0	158.1
PA0393000	PA NAZARE	2643.6	489349.3	0.0	0.0	825.0	152806.0			1647.2	305140.4	2472.1	457946.4	947295.7	
PA0394000	PAE TRÊS IRMÃS	0.0	0.0	0.0	0.0	517.6	90578.2	327.9	57382.2	0.0	3.4	845.5	147963.8	147963.8	91.8

PA0395000	PAE ILHA COROA NOVA	0.0	0.0	0.0	0.0	671.3	117478.2					671.3	117478.2	117478.2	81.8
PA0396000	PAE ILHA MARINTEUA	3.3	584.6	0.0	0.0	343.3	60071.6	10.7	1874.5			354.0	61946.1	62530.7	32.6
PA0397000	PAE ILHA PACUÍ	0.0	0.0	0.0	0.0	760.5	133087.0					760.5	133087.0	133087.0	46.8
PA0398000	PAE ILHA ITANDUBA	0.0	0.0	0.0	0.0	780.9	136657.0	74.3	13004.2			855.2	149661.2	149661.2	46.7
PA0399000	PAE ILHA ITAUNA	0.0	0.0	0.0	0.0	755.3	132176.2					755.3	132176.2	132176.2	146.7
PA0400000	PAE ILHA AJARAÍ	0.0	0.0	0.0	0.0	345.3	60423.9					345.3	60423.9	60423.9	23.5
PA0401000	PAE ILHA SANTANA DE CAMETÁ	0.0	0.0	0.0	0.0	2546.9	445704.5	7.2	1260.2			2554.1	446964.7	446964.7	84.0
PA0402000	PA NOVA VIDA	1998.9	372044.2	0.0	0.0	404.3	75223.0			1665.4	310445.5	2069.7	385668.5	757712.6	
PA0403000	PA GLEBINHA	709.1	131956.8	0.0	0.0	43.3	8056.5			1007.8	187788.8	1051.1	195845.3	327802.1	
PA0404000	PAE ILHA NOVA	0.0	0.0	0.0	0.0	227.9	39880.0					227.9	39880.0	39880.0	18.5
PA0405000	PAE ILHA JUPATITUBA DE CURRALINHO	666.7	119695.2	0.0	0.0	9964.6	1798014.8	75.3	14039.3			10039.9	1812054.1	1931749.3	191.5
PA0406000	PAE ILHA CANATICU	0.0	0.0	0.0	0.0	1061.1	197871.5			62.1	11587.3	1123.2	209458.8	209458.8	10.7
PA0407000	PAE ILHA DO MUTUM	4.7	819.5	0.0	0.0	2749.5	483228.1			1517.8	267393.3	4267.3	750621.4	751440.9	670.1
PA0408000	PAE ILHA DO FURO GRANDE	6.5	1135.4	0.0	0.0	1311.4	229493.3			0.1	14.3	1311.5	229507.6	230643.1	3.3
PA0409000	PAE ILHA ITABOCA	7.9	1385.5	0.0	0.0	2224.8	389338.8					2224.8	389338.8	390724.3	32.3
PA0410000	PAE ILHA PINDOBAL GRANDE	42.9	7506.5	0.0	0.0	3688.5	645488.1					3688.5	645488.1	652994.6	207.9
PA0411000	PA JUTAÍ MIRIM	1734.5	320980.8	0.0	0.0	22.7	4192.9			435.1	80512.5	457.7	84705.5	405686.2	
PA0412000	PAE ILHA COROCA	0.0	0.0	0.0	0.0	1852.7	345484.8					1852.7	345484.8	345484.8	32.9
PA0413000	PAE ILHA PAQUETÁ II	0.0	0.0	0.0	0.0	676.4	118375.2					676.4	118375.2	118375.2	94.2
PA0414000	PAE ILHA BOA VISTA	13.0	2275.9	0.0	0.0	835.8	146271.4			75.3	13176.7	911.1	159448.1	161724.1	0.1
PA0415000	PAE ILHA CHAVES	0.0	0.0	0.0	0.0	882.3	154402.1			0.0	0.2	882.3	154402.3	154402.3	81.4
PA0416000	PAE ILHA LARANJA	21.5	3763.6	0.0	0.0	1515.4	265201.0					1515.4	265201.0	268964.5	28.4
PA0417000	PAE ILHA SÃO JOÃO	0.0	0.0	0.0	0.0	737.6	129079.9			48.5	8492.0	786.1	137571.9	137571.9	
PA0418000	PAE ILHA TUCUPI GRANDE	0.0	0.0	0.0	0.0	1429.4	250151.0					1429.4	250151.0	250151.0	83.7
PA0419000	PAE ILHA SANTO ANTÔNIO III	0.0	0.0	0.0	0.0	1277.2	223503.1					1277.2	223503.1	223503.1	88.0
PA0430000	PA PATAUATEUA	2408.0	445381.9	0.0	0.0	322.8	59731.6			26.1	4839.3	349.0	64570.9	509952.8	
PA0431000	PA FE EM DEUS	380.9	70493.1	0.0	0.0					3.7	686.7	3.7	686.7	71179.8	

PA0433000	PA PEDRO SOUZA	1058.7	196282.6	0.0	0.0	68.3	12700.2			25.4	4713.7	93.7	17413.8	213696.4	
PA0436000	PAE ILHA SANTA MARIA	0.0	0.0	0.0	0.0	1034.8	181090.9			93.7	16393.7	1128.5	197484.6	197484.6	53.4
PA0437000	PAE ILHA MARITUBINHA	0.0	0.0	0.0	0.0	4311.5	754504.5			0.0	0.6	4311.5	754505.1	754505.1	63.3
PA0438000	PAE ILHA ATATAZINHO	35.6	6237.5	0.0	0.0	1886.3	330101.5			26.7	4669.5	1913.0	334771.0	341008.5	109.0
PA0439000	PAE ILHA CAJUUBINHA	0.0	0.0	0.0	0.0	1992.4	348675.5					1992.4	348675.5	348675.5	98.4
PA0441000	PAE ILHA SANTA CATARINA	0.0	0.0	0.0	0.0	379.5	70769.0			16.0	2974.7	395.5	73743.6	73743.6	
PA0442000	PAE ILHA TRACUATEUA	71.4	13317.0	0.0	0.0	826.4	154098.7					826.4	154098.7	167415.7	46.2
PA0443000	PAE ILHA BELA PATRIA	5.9	1096.5	0.0	0.0	389.8	72682.6			12.0	2236.2	401.8	74918.7	76015.2	0.7
PA0444000	PAE ILHA SÃO RAIMUNDO	148.9	27767.9	0.0	0.0	2784.6	519269.5	6.5	1208.8	12.3	2284.8	2803.3	522763.2	550531.1	13.8
PA0445000	PAE ILHA SORVA	4.8	890.6	0.0	0.0	1402.0	261441.4			27.8	5192.2	1429.8	266633.6	267524.2	
PA0447000	PAE ILHA SÃO JOÃO I	164.7	30711.2	0.0	0.0	3680.3	686295.6	145.6	27144.6	14.6	2720.7	3840.4	716160.9	746872.1	61.2
PA0448000	PAE ILHA CALHEIRA	195.3	36414.6	0.0	0.0	2156.5	402152.3					2156.5	402152.3	438566.9	21.3
PA0449000	PAE ILHA MOSSORO	0.0	0.0	0.0	0.0	753.6	140519.3	0.0	2.0			753.6	140521.2	140521.2	1.1
PA0450000	PAE ILHA SAMANAJÓS	3.6	672.5	0.0	0.0	2566.3	478557.4					2566.3	478557.4	479229.9	
PA0451000	PAE ILHA ARARAS	0.0	0.0	0.0	0.0	258.8	45290.1					258.8	45290.1	45290.1	79.8
PA0452000	PAE ILHA SAO PEDRO E BARBOSA	7.0	1227.4	0.0	0.0	678.8	118794.9			2.0	354.1	680.9	119149.0	120376.4	116.6
PA0453000	PAE ILHA GRANDE DO LAGUNA	8468.8	1536111.7	0.0	0.0	60091.8	11021811.4	4368.8	342467.1	103625.9	18967553.7	168086.5	30331832.2	31867943.9	731.6
PA0454000	PAE ILHA SANTA MARIA I	0.0	0.0	0.0	0.0	766.0	134050.9			333.0	58273.0	1099.0	192323.9	192323.9	38.5
PA0455000	PAE ILHA MACUJUBIM	512.3	89901.7	0.0	0.0	4138.8	725076.5			7017.5	1229226.0	11156.3	1954302.5	2044204.2	423.7
PA0456000	PAE ILHA ATURIÁ	41.4	7239.2	0.0	0.0	5642.4	987527.0			7520.8	1316498.2	13163.2	2304025.2	2311264.4	212.7
PA0457000	PAE ILHA DOS MACACOS	8361.8	1556079.1	0.0	0.0	41924.2	7788115.9			71941.8	13381862.1	113866.0	21169978.0	22726057.1	3568.8
PA0458000	PA RIO BUJARU	7183.1	1329312.5	0.0	0.0	811.7	150214.4			67.5	12485.0	879.2	162699.4	1492011.9	
PA0459000	PA TARIRATEUA	2522.1	465829.7	0.0	0.0	603.9	111646.9			310.0	56990.4	913.9	168637.3	634467.0	
PA0461000	PAE ILHA CARARUÁ-GRANDE	18.7	3278.2	0.0	0.0	5560.6	964292.2	388.2	46686.6	854.0	143745.1	6802.8	1154723.9	1158002.1	139.7
PA0462000	PAE LUZ DA VIDA	0.0	0.0	0.0	0.0	2801.7	490296.4			499.3	87382.9	3301.0	577679.3	577679.3	290.0
PA0464000	PAE ILHA JURUPARI	165.9	30216.0	0.0	0.0	4175.0	755265.0			15.7	2769.5	4190.7	758034.5	788250.5	5.2
PA0465000	PAE ILHA TAQUARI	130.4	23578.9	0.0	0.0	4966.6	872623.8	492.1	90365.7	687.0	120292.8	6145.7	1083282.3	1106861.2	376.6

PA0466000	PAE ILHA ITAPERA	319.7	58833.2	0.0	0.0	4749.6	854207.4	563.0	104856.7			5312.6	959064.1	1017897.2	64.3
PA0467000	PAE ILHA URUÁ II	5.0	940.4	0.0	0.0	2758.8	514452.7					2758.8	514452.7	515393.0	46.2
PA0468000	PAE ILHA SANTA APOLONIA	59.4	11080.6	0.0	0.0	1501.6	276807.2	159.0	9859.6			1660.6	286666.8	297747.4	54.6
PA0469000	PAE ILHA PANACU	0.0	0.0	0.0	0.0	3841.7	676300.7	1120.3	207857.9			4961.9	884158.5	884158.5	63.7
PA0470000	PAE ILHA SAPATEIRO	0.0	0.0	0.0	0.0	219.4	38400.0					219.4	38400.0	38400.0	45.1
PA0471000	PAE SANTA ROSA DO MARACATI	52.4	9166.6	0.0	0.0	2882.4	504423.3					2882.4	504423.3	513589.9	63.5
PA0472000	PAE ILHA SANTA MARIA II	0.0	0.0	0.0	0.0	836.8	146443.2					836.8	146443.2	146443.2	58.8
PA0473000	PAE ILHA SANTA MARIA III	0.0	0.0	0.0	0.0	68.1	11923.9					68.1	11923.9	11923.9	17.5
PA0474000	PAE ILHA MARIANA	0.0	0.0	0.0	0.0	222.4	38927.1					222.4	38927.1	38927.1	38.6
PA0475000	PAE ILHA NOSSA SENHORA DO LIVRAMENTO I	50.7	9455.3	0.0	0.0	2077.1	387330.1					2077.1	387330.1	396785.3	0.2
PA0477000	PAE ILHA ENTRE-ILHAS	0.0	0.0	0.0	0.0	22.6	3954.7					22.6	3954.7	3954.7	14.5
PA0478000	PAE ILHA JACAREUÁ	6.8	1227.2	0.0	0.0	439.0	76924.3					439.0	76924.3	78151.5	609.2
PA0479000	PAE ILHA BIRIBATUBA	4.3	756.0	0.0	0.0	222.0	38851.4					222.0	38851.4	39607.4	52.9
PA0480000	PAE ILHA DO MEIO	4.0	693.6	0.0	0.0	11485.3	2009931.8			14286.8	2500196.8	25772.2	4510128.6	4510822.2	13.2
PA0481000	PAE ILHA MARACUJÁ I	0.0	0.0	0.0	0.0	9729.0	1702578.7			11814.4	2067519.8	21543.4	3770098.5	3770098.5	24.7
PA0482000	PAE ILHA RASA	0.0	0.0	0.0	0.0	1827.3	319781.4			651.6	114028.8	2478.9	433810.2	433810.2	64.6
PA0483000	PAE ILHA DO TELES	0.0	0.0	0.0	0.0	2198.2	384689.0			1243.0	217517.9	3441.2	602206.9	602206.9	71.2
PA0484000	PAE ILHA CALDEIRÃO	0.0	0.0	0.0	0.0	2273.7	397898.4	474.5	83037.3	5139.1	899348.2	7887.3	1380283.9	1380283.9	103.4
PA0485000	PAE ILHA CONCEIÇÃO I	83.7	14642.7	0.0	0.0	8660.3	1515559.7			3836.0	671304.3	12496.4	2186864.0	2201506.6	4.3
PA0486000	PAE ILHA SALVADOR	1.7	298.4	0.0	0.0	13451.0	2353931.6			12221.8	2138808.4	25672.8	4492740.0	4493038.5	15.3
PA0487000	PAE ILHA QUEIMADA	183.1	32566.2	0.0	0.0	24246.1	4243333.5	20000.4	3500065.7	36895.9	6456959.5	81142.3	14200358.8	14232925.0	4141.0
PA0488000	PAE ILHA DOS CARÁS	0.0	0.0	0.0	0.0	6583.0	1152033.1			6056.3	1059849.5	12639.3	2211882.6	2211882.6	674.1
PA0489000	PAE ILHA PANEMA	0.8	134.1	0.0	0.0	2676.7	468414.6	1.4	252.2	4323.6	756628.4	7001.7	1225295.1	1225429.2	536.2
PA0490000	PAE ILHA JURUPARI I	604.3	105225.6	0.0	0.0	9194.8	1587375.5	6364.9	657952.8	19991.8	3488114.0	35551.5	5733442.3	5838667.9	2577.7
PA0491000	PAE ILHA BAIANO	0.0	0.0	0.0	0.0	2060.7	360620.6			1529.7	267699.5	3590.4	628320.1	628320.1	237.9
PA0492000	PAE ILHA TRAMBIOCA	1881.2	329213.6	0.0	0.0	5038.6	881748.4	43.1	7535.1	99.6	17435.6	5181.3	906719.1	1235932.7	382.2
PA0493000	PA LUIS CARLOS	1275.2	235989.8	0.0	0.0							0.0	0.0	235989.8	

	PRESTES														
PA0494000	PA MARIA BONITA	1252.6	231801.9	0.0	0.0	39.1	7231.0			45.1	8353.2	84.2	15584.2	247386.1	
PA0495000	PAE ILHA PIRARUAIA	22.2	3876.6	0.0	0.0	489.5	85665.2	9.4	1638.3			498.9	87303.4	91180.1	75.4
PA0496000	PAE ILHA IOIÁS	0.0	0.0	0.0	0.0	211.7	37040.9					211.7	37040.9	37040.9	67.8
PA0497000	PAE ILHA DO CABO DICO	0.0	0.0	0.0	0.0	418.9	73299.1			117.7	20596.4	536.5	93895.6	93895.6	
PA0498000	PAE TIRIRICA	215.2	37661.9	0.0	0.0	2413.4	422338.8	36.0	6303.8			2449.4	428642.7	466304.6	182.1
PA0499000	PAE ILHA DO BOI	0.0	0.0	0.0	0.0	2438.4	426713.2					2438.4	426713.2	426713.2	132.5
PA0500000	PAE ILHA JAPIIM SECO	0.0	0.0	0.0	0.0	2073.9	362931.4					2073.9	362931.4	362931.4	132.1
PA0501000	PAE ILHA MURUMURU I	80.3	14044.9	0.0	0.0	5159.0	902827.4					5159.0	902827.4	916872.3	98.2
PA0502000	PAE ILHA DO TANGARAZINHO	31.5	5509.2	0.0	0.0	1820.8	318647.3					1820.8	318647.3	324156.6	21.0
PA0503000	PAE ILHA FURO MUANÁ	17.5	3061.3	0.0	0.0	2070.4	362325.3			91.1	15943.1	2161.5	378268.3	381329.6	37.4
PA0504000	PAE COMPLEXO BATUQUE	0.0	0.0	0.0	0.0	161.2	28206.9					161.2	28206.9	28206.9	115.6
PA0505000	PAE ILHA MIRITIAPINA	19.8	3467.8	0.0	0.0	5531.2	967955.1			12910.6	2259357.8	18441.8	3227313.0	3230780.8	376.2
PA0506000	PAE ILHA ITUQUARA	0.0	0.0	0.0	0.0	6822.8	1193992.1			21738.1	3804174.2	28561.0	4998166.3	4998166.3	375.4
PA0507000	PA ABRIL VERMELHO	6304.1	1166556.5	0.0	0.0	478.3	88054.2			0.9	161.9	479.2	88216.1	1254772.6	
PA0508000	PAE ILHA PRACAXI	439.8	76965.9	0.0	0.0	5233.4	915958.6			4716.1	825482.5	9949.5	1741441.1	1818407.0	1135.9
PA0509000	PAE ILHA BUIUSSU	0.0	0.0	0.0	0.0	8385.0	1467374.1			7686.5	1345140.3	16071.5	2812514.5	2812514.5	1453.8
PA0510000	PAE ILHA LIMÃO	347.0	60717.7	0.0	0.0	14606.1	2556072.7			25210.0	4411754.4	39816.2	6967827.1	7028544.8	628.3
PA0511000	PAE ILHA PEREIRA	21.3	3722.8	0.0	0.0	3185.1	557387.5			5331.4	932994.4	8516.5	1490381.9	1494104.7	603.8
PA0512000	PAE ILHA SANTO AMARO II	533.2	93313.5	0.0	0.0	5299.0	927330.5			4687.0	820229.3	9986.1	1747559.7	1840873.3	553.8
PA0513000	PAE ILHA JEJUTEUA	1554.0	287580.1	0.0	0.0	1066.8	197427.8			1528.0	282764.2	2594.8	480192.0	767772.1	32.7
PA0514000	PAE ILHA BOM SAMARITANO	601.3	111365.4	0.0	0.0	5387.2	999595.8			91.5	16946.6	5478.7	1016542.4	1127907.8	101.5
PA0515000	PAE ILHA PRACUUBA-GRANDE	542.1	99987.8	0.0	0.0	2948.7	539425.8	74.9	13865.2	132.4	24121.5	3156.1	577412.6	677400.4	40.6
PA0516000	PAE ILHA CARIÁ-GUAJARÁ	1066.7	197400.4	0.0	0.0	2724.9	504275.7			180.1	33332.4	2905.0	537608.1	735008.6	136.6
PA0517000	PAE ILHA SÃO RAIMUNDO II	658.1	122025.8	0.0	0.0	5215.7	925551.9	834.9	41804.3	18.0	3349.0	6068.6	970705.2	1092731.0	174.5
PA0518000	PAE ILHA CENTRAL	7178.8	1337273.8	0.0	0.0	21542.4	4012792.3	1924.7	358720.9	500.4	93270.0	23967.4	4464783.3	5802057.1	402.2
PA0519000	PAE BAIXO ANAJÁS I	24.1	4216.6	0.0	0.0	5450.6	953857.8			9064.8	1586345.7	14515.4	2540203.5	2544420.1	970.1

PA0520000	PAE BAIXO ANAJÁS II	3145.7	553088.9	0.0	0.0	34140.2	5977713.7			62532.5	10948941.3	96672.7	16926655.1	17479744.0	1943.9
PA0521000	PE BORBA GATO	3518.7	654454.0	0.0	0.0	4435.1	826961.9			553.2	103160.4	4988.4	930122.3	1584576.3	
PA0522000	PE PINDORAMA	2927.2	543524.0	0.0	0.0	946.7	176276.6			97.6	18180.1	1044.2	194456.8	737980.7	
PA0523000	PE ITABIRA	226.4	41420.2	0.0	0.0	252.1	45065.1			24.6	4417.1	276.6	49482.2	90902.4	0.0
PA0524000	PE MOCAJUBA	496.5	90596.1	0.0	0.0	149.1	26574.6					149.1	26574.6	117170.7	
PA0525000	PAE ILHA CHARAPUCU	404.3	70754.4	0.0	0.0	66133.2	11573307.5	49.7	8700.0	122300.9	21402652.3	188483.8	32984659.8	33055414.2	7765.2
PA0526000	PAE ILHA DO PARÁ	0.0	0.0	0.0	0.0	17070.4	2987315.8			23658.4	4140216.8	40728.8	7127532.5	7127532.5	2171.0
PA0527000	PAE ILHA TERÊ	0.0	0.0	0.0	0.0	4287.9	750381.2			62.9	11007.3	4350.8	761388.4	761388.4	81.8
PA0529000	PAE ILHA PAQUETÁ III	1.5	260.0	0.0	0.0	248.9	43557.1					248.9	43557.1	43817.1	30.7
PA0530000	PAE ILHA ARARAIM	0.0	0.0	0.0	0.0	674.2	117976.5					674.2	117976.5	117976.5	83.8
PA0531000	PAE ILHA SARACÁ	0.0	0.0	0.0	0.0	1200.3	210051.1					1200.3	210051.1	210051.1	90.3
PA0532000	PAE ILHA TATUOCA-JAPIIM	3021.2	528717.9	0.0	0.0	18052.7	3159221.8			47.0	8217.1	18099.7	3167438.8	3696156.7	540.9
PA0533000	PE ITAPEVA	1046.2	191810.6	0.0	0.0	494.8	88112.7	530.3	92842.3	220.1	40091.5	1245.1	221046.5	412857.1	17.0
PA0534000	PAE ILHA UMARITUBA JUACARA	0.0	0.0	0.0	0.0	2229.4	390487.9	28.3	4956.3			2257.7	395444.1	395444.1	68.1
PA0535000	PAE ILHA ANAJAI	395.2	72742.5	0.0	0.0	921.1	163044.5					921.1	163044.5	235786.9	63.8
PA0536000	PAE ILHA VITÓRIA	0.0	0.0	0.0	0.0	1359.3	237900.8	14.0	2457.6	497.7	87102.5	1871.1	327460.8	327460.8	129.7
PA0537000	PAE ILHA ANGAPIJO	0.0	0.0	0.0	0.0	753.6	131882.3	10.1	1764.4			763.7	133646.6	133646.6	95.9
PA0538000	PAE ILHA URUBUQUARA	44.3	8201.8	0.0	0.0	2310.8	426529.1			398.9	73812.8	2709.7	500341.9	508543.7	255.5
PA0539000	PAE ILHA CONCEICAO DE MOCAJUBA	23.1	4040.7	0.0	0.0	1266.9	221699.0	1.5	269.7			1268.4	221968.6	226009.4	239.8
PA0540000	PAE ILHA GRANDE DE VISEU	0.2	28.5	0.0	0.0	1140.7	199627.3	5.0	875.6	6.0	1053.9	1151.8	201556.8	201585.3	248.8
PA0541000	PAE ILHA TAUARE	6.0	1056.1	0.0	0.0	1819.6	318432.7	26.5	4643.8	3.5	609.9	1849.6	323686.3	324742.5	219.1
PA0542000	PAE ILHA CURUPIRA	0.0	0.0	0.0	0.0	951.9	166578.7					951.9	166578.7	166578.7	12.5
PA0543000	PAE ILHA TURUÇU	0.0	0.0	0.0	0.0	1804.8	315836.9					1804.8	315836.9	315836.9	142.0
PA0544000	PAE ILHA MACHADO	0.0	0.0	0.0	0.0	1905.9	333525.2					1905.9	333525.2	333525.2	51.4
PA0555000	PAE ILHA MACACAA	11.2	1964.8	0.0	0.0	6603.3	1155581.7			3.0	523.2	6606.3	1156104.9	1158069.8	187.2
PA0556000	PAE ILHA ACAJUI	13.1	2285.4	0.0	0.0	812.8	142241.3					812.8	142241.3	144526.7	58.0
PA0557000	PAE ILHA CUPIJO MIRIM	0.0	0.0	0.0	0.0	1307.7	228839.0			564.8	98841.1	1872.5	327680.1	327680.1	77.2

PA0559000	PA SOROROCA	583.3	107941.7	0.0	0.0	107.5	19676.2	194.8	35285.9	26.2	4851.5	328.5	59813.5	167755.2	
PA0560000	PA CANDEUA	372.7	68888.5	0.0	0.0	225.6	40416.3	86.3	15122.4	7.9	1387.1	319.9	56925.8	125814.3	
PA0561000	PA MARIAHI	2098.4	388043.2	0.0	0.0	755.6	139190.2			9.9	1829.7	765.5	141019.8	529063.0	
PA0562000	PA CARLOS LAMARCA	940.9	174117.5	0.0	0.0	2.1	391.7			4.0	737.0	6.1	1128.7	175246.2	
PA0563000	PA SAO LOURENCO	804.6	146923.7	0.0	0.0	433.2	76736.0			13.6	2388.8	446.8	79124.8	226048.5	3.7
PA0564000	PA FORTALEZA	1049.2	190164.6	0.0	0.0	764.8	136239.7			62.4	11005.9	827.2	147245.6	337410.3	32.0
PA0565000	PA VALE DO ARIACAUA	6400.2	1184321.5	0.0	0.0	1678.1	310710.7			482.2	89360.2	2160.3	400070.9	1584392.4	
PA0567000	PAE ILHA JAPICHAUA	5421.3	978797.9	0.0	0.0	12910.9	2278818.8			29903.6	5286966.0	42814.5	7565784.8	8544582.7	663.4
PA0569000	PAE ILHA SETUBAL	0.0	0.0	0.0	0.0	170.4	31769.5					170.4	31769.5	31769.5	1.0
PA0570000	PAE ILHA SOBERANA	0.0	0.0	0.0	0.0	294.7	54955.3					294.7	54955.3	54955.3	
PA0571000	PAE ILHA MUTUNQUARA	0.0	0.0	0.0	0.0	10154.9	1777105.7			5125.4	896950.4	15280.3	2674056.1	2674056.1	881.0
PA0572000	PAE ILHA ARANAI	114.9	20113.0	0.0	0.0	4900.7	857616.4			9568.2	1674427.4	14468.8	2532043.8	2552156.9	622.3
PA0573000	PAE ILHA QUANGUERA	0.0	0.0	0.0	0.0	92.8	17304.8			17.1	3188.1	109.9	20492.9	20492.9	0.5
PA0574000	PAE ILHA BITUBA	0.0	0.0	0.0	0.0	112.7	21025.5					112.7	21025.5	21025.5	1.7
PA0576000	PAE ILHA CAPITEUA	0.0	0.0	0.0	0.0	101.3	17729.9					101.3	17729.9	17729.9	26.4
PA0580000	PAE ILHA SOROROCA	21.9	3832.8	0.0	0.0	459.7	80453.8			23.6	4136.7	483.4	84590.5	88423.3	35.8
PA0582000	PAE ILHA PURURE	3135.8	559195.3	0.0	0.0	3648.6	640953.8			7851.7	1382205.5	11500.4	2023159.3	2582354.7	447.5
PA0583000	PAE ILHA MUTUTI	320.0	56005.6	0.0	0.0	20720.2	3626036.5			27502.7	4812965.1	48222.9	8439001.6	8495007.2	774.4
PA0585000	PA PAIXAO	2098.0	388249.9	0.0	0.0	8.4	1548.1			48.1	8897.6	56.4	10445.7	398695.6	
PA0586000	PA GALILEIA	2981.8	551813.3	0.0	0.0	30.2	5582.5			27.2	5030.4	57.3	10612.9	562426.2	
PA0587000	PA ALTO ALEGRE	1624.5	300639.0	0.0	0.0	33.9	6271.1			64.1	11859.4	98.0	18130.6	318769.6	
PA0588000	PA JERICO	2887.5	534365.4	0.0	0.0	34.1	6305.2			38.0	7034.6	72.1	13339.8	547705.2	
PA0589000	PA MACARANDUBA	1250.0	229952.5	0.0	0.0	2.1	374.5			11.9	2088.6	14.1	2463.1	232415.6	
PA0590000	PAE ILHA MARAJOZINHO	18.7	3267.8	0.0	0.0	746.2	130582.2			667.5	116805.9	1413.6	247388.1	250655.9	236.8
PA0591000	PAE ILHA ARARAMA I	248.5	43484.7	0.0	0.0	1909.1	334083.9			6475.9	1133282.4	8385.0	1467366.3	1510851.0	368.4
PA0592000	PAE ILHA CAJUUNA	105.4	18453.5	0.0	0.0	1654.8	289597.6			1143.2	200067.0	2798.1	489664.6	508118.1	308.6
PA0593000	PAE ILHA JURARA	0.0	0.0	0.0	0.0	1032.2	180634.2			1220.1	213513.6	2252.3	394147.8	394147.8	168.2
PA0597000	PAE ILHA QUATI	0.0	0.0	0.0	0.0	380.1	66522.1					380.1	66522.1	66522.1	19.2

PA0598000	PAE ILHA PERIQUITÃO	0.0	0.0	0.0	0.0	105.3	18433.6					105.3	18433.6	18433.6	9.1
PA0599000	PAE ILHA PROVIDENCIA	0.0	0.0	0.0	0.0	491.9	86081.2			14.2	2484.3	506.1	88565.5	88565.5	12.9
PA0650000	PAE ILHA SANTA LUZIA	0.0	0.0	0.0	0.0	1986.4	347612.5			138.4	24223.4	2124.8	371835.9	371835.9	28.4
PA0651000	PAE ILHA BUIUSSU DO ATATA	0.0	0.0	0.0	0.0	676.0	118296.8			9.7	1698.4	685.7	119995.2	119995.2	51.2
PA0652000	PAE ILHA CIPOTEUA	0.0	0.0	0.0	0.0	127.8	22365.6					127.8	22365.6	22365.6	14.6
PA0653000	PAE ILHA BOM SUCESSO	7.1	1240.8	0.0	0.0	704.8	123346.9			16.0	2795.7	720.8	126142.6	127383.3	15.8
PA0655000	PAE ILHA CAMALEOES	0.0	0.0	0.0	0.0	2107.5	230860.0	1044.9	114454.4	326.2	35728.5	3478.6	381042.9	381042.9	332.1
PA0656000	PAE ILHA POAMPE	0.0	0.0	0.0	0.0			382.4	41884.4			382.4	41884.4	41884.4	239.3
PA0657000	PAE ILHA CRAJURU	30.0	5587.1	0.0	0.0	206.3	38463.2					206.3	38463.2	44050.3	
PA0658000	PAE ILHA ITABOCA II	17.5	3060.9	0.0	0.0	300.3	52550.2					300.3	52550.2	55611.2	40.0
PA0659000	PAE ILHA AZEITE	71.0	12425.5	0.0	0.0	75.1	13145.8					75.1	13145.8	25571.3	23.5
PA0660000	PAE ILHA SANTA CRUZ	0.0	0.0	0.0	0.0	299.7	52443.5					299.7	52443.5	52443.5	38.2
PA0661000	PAE ILHA JACAREZINHO	1281.9	224335.4	0.0	0.0	8546.8	1495685.7			19626.0	3434548.1	28172.8	4930233.8	5154569.2	2383.0
PA0663000	PAE ILHA ARAJAPANEMA II	0.0	0.0	0.0	0.0	50.1	9338.3			6.0	1126.8	56.1	10465.1	10465.1	6.5
PA0664000	PAE ILHA ITAPUPANA	0.0	0.0	0.0	0.0	157.7	29414.4					157.7	29414.4	29414.4	
PA0665000	PAE ILHA ARICURA	0.0	0.0	0.0	0.0	307.6	53825.4					307.6	53825.4	53825.4	86.3
PA0666000	PAE ILHA GRANDE DE GURUPA	586.6	102655.9	0.0	0.0	73060.7	12785627.9	13919.1	2435843.5	204287.8	35750357.3	291267.6	50971828.7	51074484.5	4337.5
PA0667000	PAE ILHA SANTA BARBARA	0.0	0.0	0.0	0.0	124.8	21839.3			805.5	140957.2	930.3	162796.6	162796.6	115.7
PA0668000	PAE ILHA GURUPAI	0.0	0.0	0.0	0.0	3573.2	625302.4			2499.8	437456.4	6072.9	1062758.8	1062758.8	193.2
PA0669000	PAE ILHA DO CORRE	1680.3	299493.7	0.0	0.0	5481.3	960072.2			12591.4	2206048.8	18072.7	3166121.0	3465614.7	1143.0
PA0679000	PAE ILHA GUARIBAS	1148.1	200925.5	0.0	0.0	4765.3	833927.3			7160.7	1253128.5	11926.0	2087055.8	2287981.3	201.7
PA0680000	PAE ILHA NAZARE	267.2	46755.5	0.0	0.0	5996.1	1049314.9			5403.0	945530.8	11399.1	1994845.7	2041601.2	428.8
SM0001000	PIC MONTE ALEGRE	214581.3	20842326.3	0.0	0.0	75732.5	10693291.0	64840.5	6409526.5	61393.7	10495120.1	201966.6	27597937.6	48440263.9	24.1
SM0004000	PA NOVO HORIZONTE	7108.5	1232756.2	0.0	0.0	3299.6	587831.3	3.9	727.3	610.1	110166.6	3913.5	698725.1	1931481.3	
SM0005000	PA ITUQUI	5035.3	700552.9	0.0	0.0	4579.1	772453.2	6.8	238.5	6490.7	1174782.0	11076.6	1947473.7	2648026.6	1.0
SM0006000	PA SURUBIM	88800.3	16547444.0	0.0	0.0	114022.0	21292380.5			203.6	37994.8	114225.6	21330375.3	37877819.2	
SM0008000	PA CRISTALINO	4963.8	919830.9	0.0	0.0	4010.2	745825.1			553.4	102906.8	4563.6	848731.8	1768562.8	

SM0009000	PA ASSURINI	26372.0	4870693.2	0.0	0.0	10738.5	1975501.7	546.5	95963.0	8929.1	1638935.3	20214.1	3710400.0	8581093.2	3153.9
SM0010000	PA RIO DO PEIXE	8082.9	1504085.8	0.0	0.0	14238.7	2653451.1			3391.1	632348.1	17629.8	3285799.2	4789885.0	
SM0011000	PA RIO CUPARI	8960.7	1596340.6	0.0	0.0	21048.2	3826359.8			6714.5	1195383.5	27762.6	5021743.3	6618083.8	
SM0012000	PA TAPAJÓS	6753.5	1253295.6	0.0	0.0	10671.4	1988261.2			4992.2	930768.5	15663.7	2919029.7	4172325.3	
SM0013000	PA CAMPO VERDE	9540.1	1611587.9	0.0	0.0	536.2	88529.4			14351.2	2357823.2	14887.3	2446352.7	4057940.5	
SM0014000	PA NOVA FRONTEIRA	8272.9	1370986.4	0.0	0.0	7896.2	1312826.4					7896.2	1312826.4	2683812.8	
SM0016000	PA MOJU I E II	21262.6	3946555.3	0.0	0.0	19619.4	3653965.1			94005.1	17516136.8	113624.5	21170101.9	25116657.3	
SM0019000	PA UIRAPURU	13875.5	2354045.6	0.0	0.0	7817.9	1280259.8			273.0	44638.0	8090.9	1324897.8	3678943.4	
SM0020000	PA TUTUÍ SUL	7440.8	1389787.9	0.0	0.0	10606.0	1982108.3			2274.2	425025.7	12880.2	2407134.0	3796921.9	
SM0021000	PA GROTAO DA ONCA	2311.5	419758.5	0.0	0.0	431.1	74428.8			928.3	163750.1	1359.4	238178.9	657937.5	
SM0022000	PA RIO TRAIRÃO	4293.9	796339.5	0.0	0.0	9912.3	1845167.9			1836.2	341222.4	11748.5	2186390.3	2982729.8	
SM0023000	PA CANOÉ	5842.7	1088212.7	0.0	0.0	3165.6	590441.2			1672.4	312012.7	4838.0	902453.9	1990666.5	
SM0024000	PA ARARAQUARA	1637.7	304943.0	0.0	0.0	1530.4	285352.0			1086.2	202542.8	2616.6	487894.8	792837.8	
SM0025000	PA CRISTALINO II	2674.5	496371.7	0.0	0.0	3207.9	597536.5			335.8	62500.7	3543.8	660037.2	1156408.9	
SM0026000	PA PILAO POENTE	7355.2	1279932.5	0.0	0.0	2300.5	393647.2			5014.6	850160.5	7315.1	1243807.7	2523740.2	
SM0027000	PA SANTA JULIA	12016.9	2032221.8	0.0	0.0	10571.9	1840851.1	80.3	15002.7			10652.2	1855853.8	3888075.6	
SM0028000	PA BRASIL NOVO	5352.4	997381.8	0.0	0.0	1764.6	329571.0					1764.6	329571.0	1326952.8	
SM0029000	PA RIO BONITO	3822.1	710093.3	0.0	0.0	4270.1	794692.3			485.0	90648.1	4755.2	885340.4	1595433.7	
SM0030000	PA BUERU	1005.0	186622.3	0.0	0.0	934.0	173996.2			1048.8	195226.2	1982.9	369222.3	555844.6	
SM0031000	PA TAPERA VELHA	4035.4	749318.9	0.0	0.0	3228.2	601441.3			5374.7	1001362.7	8602.9	1602803.9	2352122.9	
SM0032000	PA CORTA CORDA	8405.1	1562070.9	0.0	0.0	63226.5	11788755.2			6527.1	1216583.3	69753.6	13005338.4	14567409.3	12.2
SM0033000	PA CRUZEIRÃO	11264.3	1857206.4	0.0	0.0	22629.4	3704731.0			1421.4	232792.5	24050.7	3937523.4	5794729.8	
SM0034000	PA SOCÓ I	13587.1	2310786.9	0.0	0.0	3533.0	655488.0	65.5	8812.1	5391.3	1002476.0	8989.8	1666776.0	3977563.0	493.4
SM0035000	PA PARAÍSO	4900.8	854747.5	0.0	0.0	25844.1	4729111.7			33865.8	6219795.7	59709.9	10948907.4	11803655.0	
SM0036000	PA BOM JARDIM	52770.5	9805825.3	0.0	0.0	11700.4	2185398.4			28197.9	5266963.8	39898.2	7452362.3	17258187.6	
SM0037000	PA RIO ARATAU	43760.4	8139823.5	0.0	0.0	1560.8	291364.2			24308.0	4541890.1	25868.8	4833254.3	12973077.8	
SM0038000	PA JACARÉ	2237.9	418020.6	0.0	0.0	25099.0	4690557.6					25099.0	4690557.6	5108578.2	4.5
SM0039000	PA MIRITITUBA	10662.4	1962406.4	0.0	0.0	14406.5	2667962.7			229.3	42539.6	14635.8	2710502.2	4672908.6	

SM0040000	PA PILAO POENTE II E III	6292.7	1091923.1	0.0	0.0	1998.9	337245.7			3304.2	555361.3	5303.1	892607.0	1984530.0	
SM0041000	PA ALTO PARÁ	1698.8	316201.9	0.0	0.0	42350.2	7897360.1			986.7	184004.9	43336.9	8081365.0	8397566.9	
SM0043000	PA NOVA ESPERANÇA	4476.1	682757.2	0.0	0.0	19.9	3519.1			28.0	5079.3	47.9	8598.4	691355.6	
SM0044000	PA PLACAS	11370.5	2076151.5	0.0	0.0	16641.2	3067683.5			5046.3	926111.1	21687.6	3993794.5	6069946.0	
SM0045000	PA RIO DAS PEDRAS	18702.3	3252949.2	0.0	0.0	15298.8	2708653.1			2953.7	512303.2	18252.5	3220956.2	6473905.4	
SM0046000	PA CURUÁ-UNA	3165.8	580768.5	0.0	0.0	7986.7	1473330.9			6073.0	1130619.0	14059.7	2603949.9	3184718.4	
SM0047000	PA AREIA	6321.8	1087751.7	0.0	0.0	13341.4	2332742.7			324.8	60632.0	13666.2	2393374.7	3481126.3	
SM0048000	PA IGARAPE FLORES	12645.9	2361065.3	0.0	0.0	9064.8	1693608.2			415.5	77642.9	9480.4	1771251.2	4132316.5	
SM0049000	PA PENETECAUA	12576.5	2347087.5	0.0	0.0	10131.9	1892149.8			36.5	6819.2	10168.4	1898969.0	4246056.6	
SM0050000	PA ACARAI	1144.2	213127.8	0.0	0.0	3826.9	714806.3			417.7	77983.9	4244.5	792790.2	1005918.0	
SM0051000	PA LARANJAL	8006.5	1492946.7	0.0	0.0	4718.4	878906.4			0.4	80.5	4718.9	878987.0	2371933.7	
SM0052000	PA CAMPOS DE PILAR	2867.1	378016.5	0.0	0.0	119.6	15007.1	182.1	14428.0	473.7	49598.9	775.3	79034.1	457050.6	
SM0053000	PA YPIRANGA	8789.6	1565317.2	0.0	0.0	22589.6	4022488.7			185.9	34536.0	22775.5	4057024.7	5622341.9	
SM0054000	PA TRAJAP	720.6	130236.6	0.0	0.0	2978.2	555015.6	1430.0	262501.0	217.6	40650.2	4625.8	858166.8	988403.4	235.7
SM0055000	PA CAMPOS DO POPÓ	6490.9	769129.1	0.0	0.0	1087.8	120313.1			499.1	59223.9	1586.9	179537.0	948666.1	
SM0057000	PA SÃO BENEDITO	4366.1	810831.2	0.0	0.0	6184.1	1151268.4			51.5	9598.7	6235.7	1160867.1	1971698.3	
SM0058000	PA RESSACA	10696.5	1873659.8	0.0	0.0	5721.5	1001080.7			9848.8	1718990.9	15570.3	2720071.5	4593731.4	165.9
SM0059000	PA MORRO DAS ARARAS	11082.6	1880757.4	0.0	0.0	3186.3	538160.6			6270.3	1037081.9	9456.6	1575242.5	3455999.9	88.2
SM0060000	PA ITAPUAMA	8616.5	1610342.8	0.0	0.0	8596.1	1606521.2	87.6	16379.9	1918.5	358553.0	10602.2	1981454.0	3591796.9	19.9
SM0061000	PA JURAUA	1798.6	334926.0	0.0	0.0	1409.3	262820.0			882.0	164472.5	2291.3	427292.5	762218.5	
SM0062000	PA ARAPARI	3248.0	574997.1	0.0	0.0	2569.1	436714.7			3082.8	514975.6	5651.9	951690.4	1526687.4	9.3
SM0064000	PA TUTUI NORTE	2528.6	455955.6	0.0	0.0	21441.6	3974810.0			588.4	109321.2	22030.1	4084131.2	4540086.8	
SM0065000	PA ÁREA CUMINÁ E TROMBETAS	7508.4	1393908.1	0.0	0.0	14437.4	2691337.1	594.6	108000.0	1347.6	251251.3	16379.6	3050588.5	4444496.6	1190.5
SM0066000	PDS IGARAPÉ DO ANTA E CURUÁ UMA	241.6	45058.0	0.0	0.0	2307.2	430245.4			8036.7	1498680.2	10343.9	1928925.6	1973983.6	
SM0067000	PDS ANAPU I	5461.2	955048.3	0.0	0.0	7138.7	1263237.5			11413.7	1999379.1	18552.3	3262616.7	4217665.0	
SM0069000	PDS ANAPU III	209.6	39072.4	0.0	0.0	12702.3	2368941.9			11345.0	2115911.5	24047.3	4484853.4	4523925.9	
SM0070000	PDS ANAPU IV	597.9	111602.2	0.0	0.0	3354.9	626801.2			2187.0	408565.8	5541.8	1035367.0	1146969.2	
SM0073000	PDS CUPARI	653.0	108263.6	0.0	0.0	14083.0	2502229.0			15263.1	2839522.6	29346.1	5341751.6	5450015.2	

SM0074000	PDS DIVINÓPOLIS	2384.1	443975.1	0.0	0.0	22539.7	4144276.0			4562.4	845994.4	27102.1	4990270.3	5434245.4	
SM0075000	PDS NOVO MUNDO	778.0	144448.2	0.0	0.0	17040.3	3021546.8			12176.0	2181247.2	29216.4	5202794.0	5347242.2	
SM0099000	PDS SERRA AZUL	3821.6	669420.0	0.0	0.0	27944.5	4747584.9	4849.8	790365.7	42178.9	7477333.4	74973.2	13015284.0	13684703.9	
SM0101000	PDS ADEMIR FEDERICCE	13141.3	2452042.7	0.0	0.0	214762.3	40059807.4			5246.4	978356.0	220008.8	41038163.4	43490206.1	7.9
SM0104000	PAE LAGO GRANDE	115260.3	17882535.7	0.0	0.0	56210.2	10279921.2	9430.3	445225.0	84265.8	15284787.2	149906.3	26009933.4	43892469.1	11397.2
SM0105000	PA MARIPÁ	6209.0	477517.1	0.0	0.0	2326.7	81864.5	2447.7	63500.5	52.1	894.6	4826.5	146259.6	623776.7	521.2
SM0106000	PA BAIXÃO	6942.0	585163.6	0.0	0.0	3018.5	226643.2	1671.0	47116.2	99.2	8166.0	4788.7	281925.4	867089.0	336.3
SM0107000	PA MORIÇOCA	624.3	57131.5	0.0	0.0	250.2	19858.7			11.0	849.5	261.1	20708.1	77839.6	
SM0108000	PA VAI QUEM QUER	1151.4	132583.4	0.0	0.0	872.2	113261.2			30.0	3279.8	902.2	116541.0	249124.5	
SM0109000	PA MIRITI	9679.9	1493195.4	0.0	0.0	2521.0	411563.5			463.3	64289.0	2984.3	475852.6	1969048.0	
SM0110000	PA TERRA PRETA E OLHO D'ÁGUA	2100.3	225591.6	0.0	0.0	1324.6	97806.3	6.2	263.7	39.6	3206.5	1370.4	101276.5	326868.0	
SM0111000	PA CURUMU	1301.7	203294.3	0.0	0.0	135.0	21197.5	94.0	14323.5			229.0	35521.1	238815.4	129.8
SM0112000	PA CAMBURÃO I	11954.0	1746627.8	0.0	0.0	499.7	50375.3			458.6	44403.6	958.3	94778.8	1841406.6	
SM0113000	PAE JURUTI VELHO	23978.4	4447402.4	0.0	0.0	35282.5	6575112.5	312.3	21356.4	26168.3	4878665.5	61763.1	11475134.5	15922536.9	7313.8
SM0114000	PA URUCURITUBA	5111.3	943578.1	0.0	0.0	7684.4	1426956.2			2349.1	437651.9	10033.5	1864608.1	2808186.2	50.0
SM0115000	PA SANTA CRUZ	3874.5	717598.5	0.0	0.0	16267.8	3030704.2			1630.4	303978.1	17898.1	3334682.2	4052280.7	65.7
SM0116000	PA DANIEL DE CARVALHO	5197.6	964788.7	0.0	0.0	13779.5	2565680.8			371.4	69265.5	14150.9	2634946.2	3599734.9	730.1
SM0117000	PA BRASÍLIA LEGAL	13738.9	2536178.2	0.0	0.0	10135.0	1871427.5			563.6	104883.8	10698.6	1976311.3	4512489.5	1279.0
SM0118000	PDS BRASÍLIA	7617.1	1228482.5	0.0	0.0	11671.8	1870770.2	555.1	85930.6			12226.9	1956700.8	3185183.3	
SM0119000	PA CAMBURÃO II	2391.0	392366.8	0.0	0.0	526.7	84788.9			1036.1	164851.9	1562.8	249640.8	642007.6	
SM0120000	PA PORÃO	2704.7	419074.2	0.0	0.0	37.4	5696.3	45.9	7639.2	1.8	271.1	85.1	13606.6	432680.7	
SM0121000	PAE MISSIONÁRIO RUFINO	2157.3	286690.3	0.0	0.0	219.1	33166.7	302.8	4377.8	3382.9	493485.4	3904.8	531029.9	817720.2	2.1
SM0122000	PAE VALE DO SALGADO	12137.9	1636097.5	0.0	0.0	1216.7	197749.2	751.3	14719.8	3574.1	534490.7	5542.2	746959.8	2383057.3	274.8
SM0123000	PDS MALOCA	11304.5	1764420.2	0.0	0.0	8612.8	1401980.2	175.2	3355.7	11311.0	1822812.1	20099.0	3228147.9	4992568.2	153.0
SM0125000	PDS VALE DO JAMANXIM	11829.2	1934182.8	0.0	0.0	76408.0	12932441.2					76408.0	12932441.2	14866624.0	584.0
SM0126000	PDS ÁGUA PRETA	1941.5	358996.2	0.0	0.0	18426.8	3429542.4	14.7	2748.8	4471.8	825525.2	22913.3	4257816.3	4616812.6	
SM0127000	PDS CASTANHEIRA	1374.8	251140.1	0.0	0.0	2640.3	487231.3			21800.1	4050590.8	24440.4	4537822.1	4788962.2	
SM0128000	PA RENASCER	3798.2	708574.2	0.0	0.0	1911.2	356765.4			2870.2	535774.3	4781.5	892539.7	1601113.9	

SM0129000	PA CUPUZAL	4714.9	878525.1	0.0	0.0	288.0	53745.4			6299.5	1176670.5	6587.4	1230415.9	2108941.0	
SM0130000	PA TERRA PARA PAZ	3702.1	690818.4	0.0	0.0	22684.3	4232522.9			37776.5	7048840.2	60460.9	11281363.1	11972181.5	
SM0131000	PA JAMARY	15271.5	2609756.0	0.0	0.0	11939.9	2183668.2	178.6	4054.8	4236.6	770091.6	16355.1	2957814.6	5567570.6	58.9
SM0135000	PA CRISTO REI	7597.1	734290.5	0.0	0.0							0.0	0.0	734290.5	
SM0136000	PA REPARTIMENTO	23925.0	4433708.1	0.0	0.0	6995.1	1301800.5			6596.0	1228719.4	13591.1	2530519.9	6964228.0	
SM0137000	PA MAMURU	13962.7	2467199.4	0.0	0.0	2445.0	453540.8	531.6	26126.5	5060.2	916721.5	8036.8	1396388.8	3863588.1	121.0
SM0138000	PA CURUMU II	13477.9	2497863.4	0.0	0.0	2778.3	516974.0	11.5	144.5	6328.4	1174011.9	9118.2	1691130.4	4188993.7	505.2
SM0139000	PA CIPOAL	25998.3	4624690.6	0.0	0.0	6625.5	1155652.1			2238.2	378061.7	8863.8	1533713.8	6158404.4	
SM0140000	PA VALE DO AÇAÍ	14575.1	2530520.1	0.0	0.0	283.9	51639.0	185.4	2321.5	2991.7	537527.0	3461.0	591487.5	3122007.6	791.5
SM0141000	PAE EIXO FORTE	8412.5	1254015.0	0.0	0.0	4411.7	766701.5	861.4	125547.0	3527.4	627718.1	8800.5	1519966.6	2773981.6	5.2
SM0143000	PA ITAQUERA I	10368.5	1619906.8	0.0	0.0	14084.3	2514777.3	689.9	25019.1	7402.5	1350944.4	22176.7	3890740.9	5510647.7	459.8
SM0144000	PDS RENASCER II	302.4	56397.4	0.0	0.0	27302.9	5091451.0			9781.6	1824078.8	37084.6	6915529.8	6971927.1	
SM0145000	PA CURUÁ	15154.5	2639073.3	0.0	0.0	47926.4	8532111.9	12919.1	325079.0	12777.6	2286276.0	73623.1	11143467.0	13782540.3	4935.3
SM0146000	PDS PARAÍSO	3560.7	455786.8	0.0	0.0	94882.3	14352247.3	27489.7	1681293.9	26211.8	3866461.2	148583.9	19900002.4	20355789.2	840.4
SM0147000	PA VIRA VOLTA	1765.0	271968.3	0.0	0.0	1161.9	199367.2	3079.9	275214.9	462.2	84424.2	4704.0	559006.3	830974.7	1184.6
SM0148000	PA ACOMEC	1799.3	334772.3	0.0	0.0	5365.2	1001714.2	3494.4	628859.0	1308.0	244336.5	10167.5	1874909.8	2209682.1	2650.2
SM0149000	PDS LIBERDADE	64548.0	12037513.7	0.0	0.0	104698.1	19561487.7			74516.6	13925343.4	179214.7	33486831.1	45524344.8	99.9
SM0150000	PDS NELSON DE OLIVEIRA	890.6	145745.0	0.0	0.0	3643.7	594805.2					3643.7	594805.2	740550.3	84.1
SM0151000	PDS TERRA NOSSA	40063.4	6858545.0	0.0	0.0	107269.3	18895218.7	2459.5	454222.3			109728.8	19349441.0	26207986.1	
SM0153000	PAE REGIÃO DO CUÇARI	9708.8	1784288.7	0.0	0.0	16373.5	3010909.6	13.1	2424.5	2641.3	488528.0	19028.0	3501862.1	5286150.7	382.8
SM0154000	PDS MÁRIO BRAULE PINTO DA SILVA	0.0	0.0	0.0	0.0	4868.4	907851.2			2145.2	400038.6	7013.6	1307889.8	1307889.8	
SM0155000	PDS ESPERANÇA DO TRAIRÃO	6100.5	1133334.3	0.0	0.0	18497.5	3449211.9			197.4	36890.4	18694.9	3486102.4	4619436.6	
SM0156000	PDS NOVA UNIÃO	7818.9	1444756.0	0.0	0.0	3662.0	673375.6			162.7	30107.4	3824.7	703483.0	2148239.0	11.5
SM0157000	PA ARIXI	1104.9	185218.7	0.0	0.0	4641.5	797408.2			9.3	1690.9	4650.8	799099.0	984317.7	
SM0162000	PAE CUÇARU	447.2	5598.5	0.0	0.0	340.9	4268.1	264.2	3307.6	4.7	59.3	609.8	7635.0	13233.5	126.8
SM0163000	PAE ILHAS REUNIDAS	0.0	0.0	0.0	0.0			598.0	7487.0			598.0	7487.0	7487.0	1793.7
SM0164000	PAE PACOVAL/PRACOBAL	0.0	0.0	0.0	0.0			1523.2	19070.3			1523.2	19070.3	19070.3	800.9

SM0165000	PAE NAZARÉ	1051.7	28138.3	0.0	0.0	383.3	4799.0	3372.7	56693.6	50.3	630.2	3806.3	62122.7	90261.0	111.4
SM0166000	PAE CURRALINHO	0.0	0.0	0.0	0.0			1015.6	12714.9			1015.6	12714.9	12714.9	61.0
SM0167000	PAE IGARAPE DO CUÇARI	0.0	0.0	0.0	0.0	4.9	60.8	596.0	7462.1	3.2	40.0	604.1	7562.9	7562.9	0.2
SM0168000	PAE CUIEIRAS	0.0	0.0	0.0	0.0			257.6	3225.3			257.6	3225.3	3225.3	79.0
SM0169000	PAE COSTA DO AMAZONAS	41.7	521.9	0.0	0.0	9.7	121.6	1196.5	14980.2	3.5	43.5	1209.7	15145.3	15667.2	266.1
SM0170000	PAE ARITAPERA	0.0	0.0	0.0	0.0	4.5	55.8	9514.8	119125.6			9519.3	119181.4	119181.4	13425.3
SM0171000	PA ANAPUZINHO	8890.6	1653500.2	0.0	0.0	3143.2	586953.2			6105.6	1140304.3	9248.8	1727257.4	3380757.6	
SM0172000	PDS SANTA CLARA	783.0	131561.1	0.0	0.0	23376.2	4188208.9			17.9	3034.1	23394.1	4191243.1	4322804.2	
SM0173000	PAE PIAPÓ	0.0	0.0	0.0	0.0			400.4	5013.1			400.4	5013.1	5013.1	24.7
SM0174000	PAE SÃO DIOGO	81.7	1023.4	0.0	0.0			694.5	8695.1			694.5	8695.1	9718.5	12.1
SM0175000	PDS LIBERDADE I	15649.6	2920392.6	0.0	0.0	238119.5	44424275.3			197623.2	36868468.4	435742.7	81292743.7	84213136.3	8.5
SM0176000	PAE URUCURITUBA	0.0	0.0	0.0	0.0			2873.2	35972.8			2873.2	35972.8	35972.8	32422.0
SM0177000	PDS HORIZONTE NOVO	1449.4	270288.6	0.0	0.0	10116.3	1863101.8	9.7	1800.8	10075.6	1808673.8	20201.6	3673576.5	3943865.1	453.2
SM0179000	PAE SALVAÇÃO	0.0	0.0	0.0	0.0			10157.9	127176.3			10157.9	127176.3	127176.3	7616.2
SM0180000	PAE ATUMÃ	0.0	0.0	0.0	0.0	5.9	73.6	13546.2	169598.7	0.1	1.1	13552.2	169673.3	169673.3	16350.2
SM0181000	PA MACANÃ I	3484.7	584402.8	0.0	0.0	1044.6	190666.3			21572.9	3706099.3	22617.5	3896765.5	4481168.3	
SM0182000	PA MACANÃ II	2817.2	488987.0	0.0	0.0	19422.1	3505848.2			9172.5	1642629.6	28594.6	5148477.8	5637464.8	
SM0183000	PDS ITATA	5732.5	952527.3	0.0	0.0	68669.6	11282255.7			30882.9	5168710.2	99552.6	16450965.9	17403493.3	
SM0185000	PAE MADALENA	0.0	0.0	0.0	0.0			9907.7	124044.1			9907.7	124044.1	124044.1	4968.6
SM0186000	PAE TAPARÁ	278.1	6044.2	0.0	0.0	646.7	11404.4	6072.8	76031.5	100.5	2481.2	6820.0	89917.1	95961.3	3236.5
SM0187000	PAC ARAIPACUPU	17114.7	3144973.8	0.0	0.0	12658.7	2311618.4			461.1	84897.8	13119.8	2396516.2	5541490.0	459.2
SM0188000	PDS NOVA BRASILIA II	1286.8	238936.6	0.0	0.0	3907.0	728556.7			502.4	93690.7	4409.4	822247.5	1061184.1	
SM0189000	PA NOSSA SENHORA DE FATIMA	1069.2	198299.1	0.0	0.0	3115.2	580103.1			56.0	10390.8	3171.3	590493.9	788793.0	
SM0190000	PA RIO CIGANO	794.3	146438.7	0.0	0.0	1406.2	249673.5					1406.2	249673.5	396112.2	
SM0191000	PDS ÁGUA AZUL	6777.6	1190631.3	0.0	0.0	22915.3	3809408.7	7.2	1174.0			22922.5	3810582.8	5001214.1	207.6
SM0192000	PDS BOA VISTA DO CARACOL	3195.0	596568.1	0.0	0.0	19540.5	3651600.2			18.2	3407.3	19558.7	3655007.5	4251575.6	
SM0193000	PAC NOVA ALTAMIRA	1498.2	109288.4	0.0	0.0	283.1	7601.3	1064.9	37044.5	48.8	1182.5	1396.7	45828.3	155116.7	11.5

SM0194000	PAC CAUÇU B E BALANÇA	1734.2	131495.6	0.0	0.0	652.5	27315.4	1176.6	70612.6	52.1	4021.4	1881.2	101949.5	233445.0	
SM0195000	PAE PARANA DE BAIXO	0.0	0.0	0.0	0.0			5257.4	65822.7			5257.4	65822.7	65822.7	3683.5
SM0196000	PAE TRES ILHAS	0.0	0.0	0.0	0.0			7407.2	92737.9			7407.2	92737.9	92737.9	4307.5
SM0197000	PAE MARIA TEREZA	27.1	4593.1	0.0	0.0			641.3	8029.6	137.9	3312.3	779.3	11342.0	15935.1	620.7
SM0198000	PAE COSTA FRONTEIRA	0.0	0.0	0.0	0.0			11771.4	147377.6			11771.4	147377.6	147377.6	3903.8
SM0199000	PAE PARU	0.0	0.0	0.0	0.0			2535.0	31738.4			2535.0	31738.4	31738.4	12059.3
SM0200000	PAE CACOAL GRANDE	0.0	0.0	0.0	0.0			9013.1	112843.7			9013.1	112843.7	112843.7	613.3
SM0201000	PAE SAO PEDRO	0.0	0.0	0.0	0.0			3764.4	48087.1			3764.4	48087.1	48087.1	5604.5
SM0203000	PAE PAITUNA	108.4	1356.8	0.0	0.0	43.1	539.8	575.2	7202.0			618.4	7741.8	9098.6	1828.4
SM0204000	PAE REGIAO DOS LAGOS	512.1	27407.7	0.0	0.0	210.3	7511.9	275.6	3450.4	0.1	1.6	486.0	10964.0	38371.7	107.6
SM0205000	PAE ACARA-AÇU	0.0	0.0	0.0	0.0			1641.5	20551.7			1641.5	20551.7	20551.7	731.1
SM0206000	PAC BELA TERRA I	6114.5	1133838.3	0.0	0.0	2930.7	544921.9			1810.2	337080.5	4740.9	882002.4	2015840.7	
SM0207000	PAC BELA TERRA II	8301.3	1540965.9	0.0	0.0	1275.3	237142.0			1417.0	263899.6	2692.3	501041.6	2042007.6	
SM0208000	PDS ARTHUR FALEIRO	1945.6	362238.1	0.0	0.0	506.2	94361.2			20257.8	3777613.7	20764.0	3871974.9	4234213.0	
SM0209000	PDS AVELINO RIBEIRO	1278.8	238284.3	0.0	0.0	955.5	178184.1			19427.5	3622777.5	20383.0	3800961.6	4039246.0	
SM0210000	PAE PARANA DONA ROSA	0.0	0.0	0.0	0.0			18751.9	234773.4			18751.9	234773.4	234773.4	3380.5
SM0211000	PAE SANTA RITA	0.0	0.0	0.0	0.0			4717.0	59057.1			4717.0	59057.1	59057.1	7248.2
SM0212000	PAE VALHA-ME DEUS	0.0	0.0	0.0	0.0			1900.0	23788.1			1900.0	23788.1	23788.1	1579.6
SM0213000	PAE SALÉ	1013.3	24955.1	0.0	0.0	39.8	725.0	11311.0	177982.5	310.6	5775.5	11661.3	184483.0	209438.1	14408.2
SM0214000	PAE BALAIO	342.3	61307.0	0.0	0.0	36.8	6840.8	6868.9	116222.9	3.6	669.8	6909.3	123733.6	185040.6	5959.5
SM0215000	PDS MÃE MENININHA	3823.4	623392.5	0.0	0.0	15004.3	2445467.1	21.2	3460.2			15025.6	2448927.3	3072319.8	
SM0216000	PDS ESPERANÇA	2020.4	329267.5	0.0	0.0	15989.5	2605820.7					15989.5	2605820.7	2935088.2	
SM0217000	PA ESPERANÇA	8065.8	1324421.4	0.0	0.0	6408.6	1047127.0	451.3	74226.2			6859.8	1121353.2	2445774.5	26.8
SM0218000	PAC ANANIZAL	1026.7	190287.5	0.0	0.0	301.2	56125.1			1120.6	208780.8	1421.7	264905.9	455193.3	
SM0219000	PAC ITAPECURU	1848.4	323930.9	0.0	0.0	624.7	116133.9			2248.7	414864.1	2873.4	530998.0	854928.9	35.7
SM0220000	PAC IRIPIXI	1379.3	255636.1	0.0	0.0	58.1	10794.8			995.5	185378.6	1053.6	196173.4	451809.5	2.5
SM0221000	PAC MONTE MURIA	2086.6	387742.6	0.0	0.0	1210.3	225519.6			1555.7	289962.1	2766.0	515481.7	903224.3	

SM0222000	PAE ARAMANAÍ	1885.1	349569.3	0.0	0.0	1347.0	250574.7			99.9	18596.2	1446.8	269170.9	618740.3	0.0
SM0223000	PAE PINDOBAL	1639.6	303651.5	0.0	0.0	3862.9	718620.4			2544.9	473953.0	6407.8	1192573.4	1496224.9	55.1
SM0224000	PAE NHAMUNDA	2.5	383.9	0.0	0.0			5683.3	75345.4			5683.3	75345.4	75729.3	6207.4
SM0225000	PAE CACHOERY	6.5	1145.1	0.0	0.0			10343.1	129495.1	86.5	1083.6	10429.6	130578.6	131723.7	26271.0
SM0226000	PAE ITUQUI	2993.2	37504.5	0.0	0.0	1200.3	15211.3	2324.2	33202.4	133.4	1669.6	3657.9	50083.3	87587.8	14334.7
SM0227000	PAE CHICANTÃ	70.9	10862.5	0.0	0.0	44.4	8264.2	6883.8	380316.7	81.0	14321.2	7009.2	402902.2	413764.7	534.9
SM0228000	PAE NOSSA SENHORA DO PERPÉTUO SOCORRO	0.0	0.0	0.0	0.0	0.0	0.4	3297.6	41286.2	0.0	0.1	3297.7	41286.8	41286.8	1944.3
SM0229000	PAE ANEMA	0.0	0.0	0.0	0.0			2471.4	30941.3			2471.4	30941.3	30941.3	50.5
SM0230000	PAE JACARECAPÁ	945.5	121517.6	0.0	0.0	239.5	22889.3					239.5	22889.3	144406.8	239.5
SM0231000	PAE JAQUARÁ	1533.6	221440.0	0.0	0.0	281.5	40267.4			9.4	1428.2	290.9	41695.6	263135.6	3.7
SM0232000	PAE ALDEIA	2329.8	318226.3	0.0	0.0	312.5	33456.4	345.7	4328.1			658.2	37784.5	356010.8	1096.3
SM0233000	PAC BOM SOSSEGO	28038.1	4947118.8	0.0	0.0	27292.4	5053528.1			31529.3	5834179.6	58821.8	10887707.6	15834826.4	1228.6
SM0234000	PA PARAISO DO NORTE	15382.8	2869782.7	0.0	0.0	91034.2	16986489.6			6552.7	1222359.1	97586.9	18208848.7	21078631.4	
SM0235000	PAC SÃO SEBASTIÃO DO TUTUÍ	1762.9	327425.1	0.0	0.0	33999.3	6328857.2			4065.0	758173.2	38064.3	7087030.4	7414455.5	
SM0236000	PAC NOVA UNIÃO	3796.9	705320.7	0.0	0.0	25455.3	4745930.1			2350.1	438024.9	27805.4	5183955.0	5889275.8	
SM0237000	PDS OURO BRANCO	5973.5	1114460.8	0.0	0.0	43927.5	8195611.8			4675.6	872189.6	48603.1	9067801.3	10182262.1	
SM0238000	PAC OURO BRANCO I	3955.0	737724.0	0.0	0.0	35641.3	6647933.4			211.2	39384.4	35852.5	6687317.8	7425041.9	
SM0239000	PAC OURO BRANCO II	2534.2	473360.7	0.0	0.0	33436.9	6240984.4			1022.0	190720.3	34459.0	6431704.6	6905065.4	
SM0241000	PDS LARANJAL	2369.7	442185.4	0.0	0.0	31678.5	5913000.3					31678.5	5913000.3	6355185.7	280.8
SM0242000	PDS PIMENTAL	1555.7	263116.2	0.0	0.0	4650.0	773520.4			21.7	3600.8	4671.7	777121.2	1040237.3	162.5
SM0246000	PDS IRMÃ DOROTY	2568.7	479007.9	0.0	0.0	25308.6	4719544.6			2630.4	490519.5	27939.0	5210064.1	5689072.0	
SM0249000	PEAEX ARUA	1645.9	305072.7	0.0	0.0	15501.0	2888410.8			6907.5	1285353.3	22408.5	4173764.1	4478836.8	24.2
SM0250000	PEAEX VISTA ALEGRE	856.2	158575.2	0.0	0.0	4574.2	850562.2			290.5	54054.0	4864.6	904616.2	1063191.4	
SM0251000	PAE CURUÁ II	5857.6	1065977.0	0.0	0.0	31081.0	5688160.7	203.3	27497.1	7384.8	1345105.4	38669.0	7060763.2	8126740.2	605.1
SM0252000	PEAEX MAJARI	1867.2	283528.3	0.0	0.0	17608.8	2993558.5	625.1	34226.5	18699.8	2816074.3	36933.7	5843859.3	6127387.6	
SM0254000	PEAEX CURUMUCURI	43265.8	6659041.6	0.0	0.0	34304.9	6346381.3	4231.8	298527.2	39110.9	7186358.1	77647.6	13831266.5	20490308.1	2317.5

SM0255000	PEAS REPARTIMENTO	227.6	42297.1	0.0	0.0	2305.2	429040.4			841.2	156703.2	3146.4	585743.7	628040.8	
SM0256000	PEAEX MARIAZINHA ARACATI	410.6	76530.1	0.0	0.0	8355.2	1557671.6			1556.1	290041.1	9911.3	1847712.7	1924242.8	
SM0259000	PEAEX PRUDENTE MONTE SINAI	922.2	171631.4	0.0	0.0	1436.9	267809.5			1927.4	359349.2	3364.3	627158.7	798790.1	46.4
SM0260000	PAE MONTANHA E MANGABAL	643.7	119658.8	0.0	0.0	53120.4	9907400.7	308.1	57573.5	54.4	9990.4	53482.8	9974964.6	10094623.4	346.5
	Total in Pará	5467122.2	954645936.1	0.0	0.0	4671760.3	835805342.7	479400.3	31990533.6	2458890.0	441743939.6	7610050.6	1309539815.9	2264185752.0	316424.7
**RONDÔNIA**			0.0	0.0	0.0							0.0	0.0	0.0	
RO0006000	PIC OURO PRETO	398342.7	66820256.7	0.0	0.0	38432.5	6392530.3	1552.6	279164.3	10.1	1645.7	39995.2	6673340.3	73493597.1	925.6
RO0007000	PIC Pe.ADOLPHO ROHL	302424.8	51161883.2	0.0	0.0	18006.9	2960665.6	5268.0	959666.9	261.4	46674.5	23536.3	3967006.9	55128890.1	1480.6
RO0008000	PAD MARECHAL DUTRA	346470.6	58576238.1	0.0	0.0	124498.6	20857354.3	6493.0	1104329.6			130991.6	21961683.9	80537922.0	848.0
RO0009000	PIC SIDNEY GIRÃO	53694.8	8947233.1	0.0	0.0	12787.8	2092368.6					12787.8	2092368.6	11039601.7	
RO0010000	PIC GY PARANÁ	402320.5	61925654.8	0.0	0.0	48308.3	7175562.3	24404.5	3315038.4			72712.8	10490600.7	72416255.5	1632.2
RO0011000	PIC PAULO ASSIS RIBEIRO	260198.0	38833243.6	0.0	0.0	39504.5	5862607.0	0.7	103.2			39505.2	5862710.3	44695953.9	
RO0012000	PAD BURAREIRO	210322.1	36322984.0	0.0	0.0	78552.3	13583182.8	5458.7	997270.2	10.4	1693.1	84021.4	14582146.1	50905130.1	
RO0013000	PA URUPÁ	39435.5	6501995.3	0.0	0.0	1313.3	212539.2	189.3	30830.1	9.0	1471.8	1511.7	244841.1	6746836.5	490.7
RO0014000	PA MACHADINHO	116195.4	19326077.4	0.0	0.0	96006.5	16000189.9	537.4	95040.9			96543.9	16095230.8	35421308.2	
RO0015000	PA BOM PRINCÍPIO	67465.6	11382682.8	0.0	0.0	14606.6	2487676.4	157.5	27473.3	1777.6	307553.5	16541.7	2822703.2	14205386.0	159.5
RO0016000	PA CUJUBIM	29614.4	4957091.6	0.0	0.0	12342.2	2060124.0					12342.2	2060124.0	7017215.6	
RO0017000	PA SÃO FELIPE	16934.5	2837253.3	0.0	0.0	816.0	135786.0					816.0	135786.0	2973039.2	
RO0018000	PA VITÓRIA DA UNIÃO	18027.4	2762032.2	0.0	0.0	3281.7	486317.7					3281.7	486317.7	3248349.8	
RO0019000	PA VALE DO JAMARI	31169.8	5258479.4	0.0	0.0	19569.2	3266169.9					19569.2	3266169.9	8524649.3	303.7
RO0020000	PA DJARU UARU	22939.3	3560294.8	0.0	0.0	4323.0	474582.4	1374.1	128429.4			5697.1	603011.8	4163306.6	
RO0021000	PA RIO PRETO DO CANDEIAS	20683.2	3469886.5	0.0	0.0	13050.5	2204252.7					13050.5	2204252.7	5674139.2	
RO0022000	PA ZEFERINO	7232.2	1147024.4	0.0	0.0	321.6	52176.2					321.6	52176.2	1199200.6	
RO0023000	PA PYRINEOS	4342.7	726760.3	0.0	0.0	188.3	31089.0					188.3	31089.0	757849.3	76.9
RO0024000	PA TANCREDO NEVES	28284.0	4699979.9	0.0	0.0	375.0	61854.8					375.0	61854.8	4761834.6	569.2
RO0025000	PA ITAPIREMA	5892.7	989073.9	0.0	0.0	146.8	24636.4					146.8	24636.4	1013710.3	123.2
RO0026000	PA JATUARANA	31940.8	5266211.1	0.0	0.0	9214.2	1505342.2					9214.2	1505342.2	6771553.3	

RO0027000	PA MARCOS FREIRE	7474.5	1217417.5	0.0	0.0	322.7	51890.0	2853.9	450468.1			3176.6	502358.1	1719775.6	
RO0028000	PA VERDE SERINGAL	8210.0	1233876.5	0.0	0.0	3006.4	442310.1	3170.6	417750.0			6177.0	860060.1	2093936.6	
RO0029000	PA BURITI	23372.4	3875716.8	0.0	0.0	2224.4	366291.9					2224.4	366291.9	4242008.7	
RO0030000	PA TARUMÃ	2939.8	485928.6	0.0	0.0	189.7	30936.6					189.7	30936.6	516865.2	47.6
RO0031000	PA NOVA CONQUISTA	16362.0	2388382.9	0.0	0.0	3453.6	444389.1	1054.2	108149.1			4507.8	552538.3	2940921.2	
RO0032000	PA RIBEIRÃO GRANDE	5732.5	902057.5	0.0	0.0	929.7	150110.0	751.5	117557.6			1681.1	267667.6	1169725.1	
RO0033000	PA CACHOEIRA	8319.8	1423042.5	0.0	0.0	782.2	133725.5					782.2	133725.5	1556768.0	
RO0034000	PA EMBURANA	2290.6	382690.4	0.0	0.0	293.3	48216.3	281.7	46372.3			575.0	94588.6	477279.0	
RO0035000	PA VÁRZEA ALEGRE	6838.4	1034724.9	0.0	0.0	1035.1	157532.1					1035.1	157532.1	1192257.0	
RO0036000	PA NOVA FLORESTA	1798.6	325065.5	0.0	0.0	2448.3	441978.2	169.3	31560.7			2617.6	473539.0	798604.5	
RO0037000	PA COLINA VERDE	24076.8	4333008.5	0.0	0.0	2980.2	541898.7	1046.5	192707.2	12.6	2341.9	4039.3	736947.8	5069956.3	
RO0038000	PA MASSANGANA	18630.6	3345833.3	0.0	0.0	6670.2	1201303.7	5048.8	909392.9			11719.0	2110696.6	5456529.8	
RO0042000	PA ADRIANA	1748.1	263353.3	0.0	0.0	189.5	28548.3					189.5	28548.3	291901.5	
RO0043000	PA RIO ALTO	35655.7	6118784.4	0.0	0.0	8092.1	1379829.3	10.8	1942.2			8102.9	1381771.5	7500555.9	
RO0044000	PA PIRAJUI	88.4	13592.3	0.0	0.0	23.9	3739.6	1379.5	216438.1			1403.4	220177.7	233770.0	
RO0045000	PA CANAÃ	2511.0	421238.2	0.0	0.0	549.3	91521.7					549.3	91521.7	512759.9	11.2
RO0046000	PA ELI MOREIRA	2511.5	383497.0	0.0	0.0	41.3	6293.7					41.3	6293.7	389790.7	
RO0047000	PA CUJUBIM II	1852.1	321660.2	0.0	0.0	960.3	168353.9					960.3	168353.9	490014.2	
RO0048000	PA SÃO FRANCISCO	3433.2	565825.8	0.0	0.0	4437.7	730066.9					4437.7	730066.9	1295892.7	
RO0049000	PA AMÉRICO VENTURA	2417.2	398589.9	0.0	0.0	412.0	67851.3					412.0	67851.3	466441.3	4.2
RO0050000	PA SANTA CATARINA	2355.8	386157.6	0.0	0.0	132.6	21608.4					132.6	21608.4	407766.0	
RO0051000	PA RIO BRANCO	4345.4	708194.3	0.0	0.0	382.3	62308.9					382.3	62308.9	770503.2	
RO0052000	PA SANTA CRUZ	34105.6	5893624.3	0.0	0.0	20548.4	3609053.5	522.2	92579.7			21070.6	3701633.2	9595257.6	
RO0053000	PA SANTA MARIA	6718.2	1129261.6	0.0	0.0	3957.0	675591.2					3957.0	675591.2	1804852.8	
RO0054000	PA TABAJARA	10178.2	1708923.6	0.0	0.0	3856.2	646847.3					3856.2	646847.3	2355770.9	
RO0055000	PA PEDRA REDONDA	9447.8	1545551.1	0.0	0.0	2771.0	455640.0					2771.0	455640.0	2001191.0	1.1
RO0056000	PA GUARAJUS	3523.7	525180.7	0.0	0.0	666.4	94759.6					666.4	94759.6	619940.3	
RO0057000	PA UNIÃO	18935.2	3129691.7	0.0	0.0	7229.0	1180800.4					7229.0	1180800.4	4310492.1	

RO0058000	PA LAJES	38867.7	6541987.8	0.0	0.0	23590.4	4012800.9	2.2	352.2			23592.6	4013153.1	10555140.9	
RO0059000	PA LAGOA NOVA	2647.3	436013.0	0.0	0.0	121.0	19756.0					121.0	19756.0	455769.0	
RO0061000	PA PALMARES	8212.8	1354051.3	0.0	0.0	1349.6	220774.1	187.1	30498.7			1536.7	251272.8	1605324.2	
RO0062000	PA SANTA CATARINA EXPANSÃO I	672.6	110132.4	0.0	0.0	27.6	4490.4					27.6	4490.4	114622.8	
RO0063000	PA SANTA MARIA II	19341.7	3355349.3	0.0	0.0	18856.2	3272668.6					18856.2	3272668.6	6628017.9	73.2
RO0064000	PA ZUMBI	1803.6	315131.3	0.0	0.0	170.3	30724.0					170.3	30724.0	345855.4	
RO0065000	PA CHICO MENDES	1553.5	253534.5	0.0	0.0	229.1	37360.0	342.7	55853.3			571.8	93213.3	346747.8	43.1
RO0066000	PA PRIMAVERA	5574.3	909614.9	0.0	0.0	454.5	74231.2					454.5	74231.2	983846.0	
RO0067000	PA MARGARIDA ALVES	7393.6	1218888.1	0.0	0.0	4292.2	699992.0	213.7	35318.0			4505.9	735310.0	1954198.1	
RO0068000	PA CHICO MENDES II	1441.3	236074.8	0.0	0.0	209.4	34145.0	330.7	53982.4			540.1	88127.4	324202.2	14.6
RO0069000	PA 14 DE AGOSTO	453.8	74828.9	0.0	0.0	24.3	4067.3			14.2	2387.2	38.6	6454.5	81283.4	
RO0070000	PA MARIA JOSÉ RIQUE	3293.6	555091.0	0.0	0.0	216.7	36835.0			5.6	918.8	222.4	37753.8	592844.8	
RO0071000	PA PROZOLINA	1346.3	249659.9	0.0	0.0	33.6	6200.0					33.6	6200.0	255860.0	
RO0072000	PA MENEZES FILHO	26149.6	4453667.9	0.0	0.0	6478.3	1112364.7	101.2	18887.2			6579.5	1131251.9	5584919.8	
RO0073000	PA MANOEL SOUZA CARDOSO	797.7	129884.6	0.0	0.0	196.0	31962.4					196.0	31962.4	161847.0	
RO0074000	PA OZIEL DOS CARAJAS	1023.5	166417.7	0.0	0.0	766.8	124964.6					766.8	124964.6	291382.3	3.2
RO0075000	PA PALMA ARRUDA	14450.1	2382018.6	0.0	0.0	6031.6	998034.8					6031.6	998034.8	3380053.5	34.7
RO0076000	PA JOSÉ CARLOS	1188.6	193716.2	0.0	0.0	409.2	66687.6					409.2	66687.6	260403.8	
RO0077000	PA CONCEIÇÃO	6170.0	1076601.7	0.0	0.0	489.1	85402.5					489.1	85402.5	1162004.2	
RO0078000	PA RENASCER	7426.4	1308654.8	0.0	0.0	7998.7	1409097.9					7998.7	1409097.9	2717752.7	
RO0079000	PA AGOSTINHO BECKER	8967.1	1554135.3	0.0	0.0	4555.2	794379.0					4555.2	794379.0	2348514.4	
RO0080000	PA CHICO MENDES III	2476.5	398300.2	0.0	0.0	230.4	37529.9	273.0	44485.8			503.3	82015.7	480315.9	
RO0081000	PA PEDRA DO ABISMO	10705.8	1760651.7	0.0	0.0	1904.4	311821.4					1904.4	311821.4	2072473.1	
RO0082000	PA LAGOA AZUL	6956.4	1139941.2	0.0	0.0	1370.8	224410.5					1370.8	224410.5	1364351.6	
RO0083000	PA CEARA	3059.2	463023.5	0.0	0.0	388.3	59250.8	663.3	100560.4			1051.7	159811.2	622834.7	
RO0086000	PA SÃO DOMINGOS	15006.5	2448122.1	0.0	0.0	4005.7	652866.0					4005.7	652866.0	3100988.1	
RO0087000	PA SÃO PEDRO	7918.4	1297577.2	0.0	0.0	1147.4	187166.0					1147.4	187166.0	1484743.2	

RO0088000	PA REVIVER	12670.9	2109157.9	0.0	0.0	1801.8	298596.4					1801.8	298596.4	2407754.3	
RO0094000	PA JOSÉODON	6710.7	1120148.6	0.0	0.0	2216.2	366163.3	1101.3	191941.2			3317.5	558104.5	1678253.1	
RO0095000	PA ZENON	7895.9	1458974.6	0.0	0.0	3863.0	720556.7					3863.0	720556.7	2179531.3	
RO0096000	PA FILADELFIA	1247.1	209894.7	0.0	0.0	752.1	126686.6	604.7	63526.1			1356.8	190212.7	400107.3	
RO0097000	PA SANTA VITORIA	428.4	68665.9	0.0	0.0	58.1	9213.3					58.1	9213.3	77879.2	
RO0098000	PA AGUINEL DIVINO	4805.3	819102.7	0.0	0.0	1439.0	251823.7					1439.0	251823.7	1070926.4	
RO0099000	PA RIO MADEIRA	2196.6	379540.5	0.0	0.0	5569.5	974724.0	296.4	15202.6			5865.9	989926.6	1369467.1	81.3
RO0100000	PA ALIANÇA	14555.5	2345055.5	0.0	0.0	8370.5	1435042.1	110.2	18827.7			8480.7	1453869.7	3798925.3	2.0
RO0101000	PA CACHOEIRA DE SAMUEL	1169.3	193698.5	0.0	0.0	497.8	81911.6					497.8	81911.6	275610.1	
RO0102000	PA CEDRO JEQUITIBÁ	4990.0	824655.2	0.0	0.0	1231.4	201792.8					1231.4	201792.8	1026448.0	
RO0103000	PA ANTONIO PEREIRA NERI	1739.4	284019.2	0.0	0.0	323.4	52740.0					323.4	52740.0	336759.2	165.4
RO0104000	PA AMIGOS DO CAMPO	5357.0	879687.8	0.0	0.0	2160.7	352837.4					2160.7	352837.4	1232525.2	
RO0106000	PA RONCAUTO	1272.3	215562.0	0.0	0.0	210.5	36324.9					210.5	36324.9	251886.9	
RO0107000	PA ASA DO AVIÃO	2334.0	388798.0	0.0	0.0	194.6	31826.3					194.6	31826.3	420624.2	
RO0108000	PA RIO CONSUELO	2605.4	449240.7	0.0	0.0	938.6	165073.4					938.6	165073.4	614314.1	
RO0109000	PA SANTA HELENA	11025.5	1826195.0	0.0	0.0	1022.2	167431.3					1022.2	167431.3	1993626.3	
RO0110000	PA MARIA MENDES	1350.5	220681.6	0.0	0.0	365.5	59591.4					365.5	59591.4	280273.0	
RO0111000	PA NELSON ALVES	827.6	137097.1	0.0	0.0	110.0	18013.1					110.0	18013.1	155110.2	
RO0112000	PA JANDAIRA	766.5	126314.2	0.0	0.0	251.2	41026.7					251.2	41026.7	167340.9	
RO0113000	PA JOANA D ARC I	8809.7	1498985.6	0.0	0.0	13157.1	2233168.7	7.2	580.4			13164.3	2233749.1	3732734.7	0.0
RO0114000	PA JOANA D ARC II	3587.1	619985.3	0.0	0.0	11128.2	1911855.7	20.9	1613.0			11149.1	1913468.7	2533454.0	0.0
RO0115000	PA JOANA D ARC III	5001.3	835307.9	0.0	0.0	19156.2	3235383.6	11.2	1967.3			19167.4	3237350.9	4072658.9	1.4
RO0116000	PA FRANCISCO JOAO	9605.0	1579787.9	0.0	0.0	3930.3	640967.5					3930.3	640967.5	2220755.4	
RO0117000	PA FLORIANO MAGNO	11311.7	1861020.2	0.0	0.0	4941.8	806092.6			174.3	28407.3	5116.1	834500.0	2695520.2	
RO0118000	PA ESMOSINA PINHO	8586.7	1432535.0	0.0	0.0	2883.8	479608.8			48.9	7973.6	2932.8	487582.4	1920117.4	
RO0119000	PA IVO INACIO	7381.1	1212076.1	0.0	0.0	5263.1	859054.3					5263.1	859054.3	2071130.5	
RO0120000	PA NOVA VIDA	7109.7	1167130.5	0.0	0.0	1474.6	240388.9					1474.6	240388.9	1407519.5	
RO0121000	PA SÃO JOSÉ DO BURITIS	8212.1	1356040.5	0.0	0.0	916.6	150006.4					916.6	150006.4	1506046.8	

RO0122000	PA SÃO PAULO	4025.0	657582.5	0.0	0.0	778.3	126846.9					778.3	126846.9	784429.5	
RO0123000	PA NILSON CAMPOS	6930.7	1138123.6	0.0	0.0	5588.3	912573.2					5588.3	912573.2	2050696.8	
RO0125000	PA ORIENTE	7548.7	1281262.5	0.0	0.0	2114.1	366716.1					2114.1	366716.1	1647978.5	
RO0126000	PA EDMILSON PASTOR	86.8	16123.6	0.0	0.0	329.1	61428.3					329.1	61428.3	77551.9	
RO0127000	PA PADRE EZEQUIEL	3406.8	567757.1	0.0	0.0	2566.6	418505.0	62.7	8731.5			2629.3	427236.5	994993.6	
RO0129000	PA PARAISO DAS ACÁCIAS	505.7	88985.5	0.0	0.0	77.2	13607.3					77.2	13607.3	102592.8	
RO0130000	PDS CERNAMBI	7343.9	1199024.4	0.0	0.0	7477.1	1218949.7					7477.1	1218949.7	2417974.2	16.2
RO0131000	PA JATOBÁ	4872.0	795141.0	0.0	0.0	1881.0	306601.4					1881.0	306601.4	1101742.4	
RO0132000	PCA NOVO ALVORECER	695.6	116186.3	0.0	0.0	29.4	4797.6					29.4	4797.6	120983.9	
RO0133000	PA BOM PRINCÍPIO II	2365.1	395448.0	0.0	0.0	51.0	8419.6					51.0	8419.6	403867.6	
RO0134000	PA BELA VISTA	1191.4	191560.4	0.0	0.0	556.4	90468.5					556.4	90468.5	282028.9	0.8
RO0135000	PA SERRA GRANDE	5304.9	923425.2	0.0	0.0	2116.7	371051.6	67.2	11757.7			2183.8	382809.3	1306234.5	
RO0136000	PA SÃO FRANCISCO DO GUAPORÉ	4193.3	685195.1	0.0	0.0	125.4	22046.5					125.4	22046.5	707241.6	
RO0139000	PA MARTIM PESCADOR	14177.9	2106373.0	0.0	0.0	1266.1	161781.7	2753.1	420530.0	0.1	8.9	4019.2	582320.6	2688693.6	25.8
RO0140000	PA GOGÓ DA ONÇA	19459.6	3265606.2	0.0	0.0	4516.5	757103.3					4516.5	757103.3	4022709.5	
RO0141000	PA CAUTARINHO	12397.4	2087488.6	0.0	0.0	3960.5	672955.1					3960.5	672955.1	2760443.6	
RO0142000	PA SAGRADA FAMÍLIA	4079.2	708510.5	0.0	0.0	988.1	173984.9					988.1	173984.9	882495.3	
RO0143000	PDS NAZARÉ E BOA VITÓRIA	789.2	137012.5	0.0	0.0	2725.1	477012.7					2725.1	477012.7	614025.3	158.1
RO0146000	PA IGARAPÉ DAS ARARAS	7456.4	1215928.1	0.0	0.0	7987.8	1302050.9					7987.8	1302050.9	2517978.9	
RO0147000	PA MARECHAL RONDON	5801.2	946641.3	0.0	0.0	5669.2	923967.1					5669.2	923967.1	1870608.4	
RO0148000	PA IGARAPÉ AZUL	5864.6	955758.1	0.0	0.0	4747.3	773667.0					4747.3	773667.0	1729425.1	
RO0149000	PA ROSANA LECY	1379.5	224824.2	0.0	0.0	1624.5	264737.2					1624.5	264737.2	489561.4	
RO0150000	PA PAU BRASIL	8673.3	1419466.7	0.0	0.0	6208.7	1011967.4					6208.7	1011967.4	2431434.2	
RO0151000	PA IGARAPE TAQUARA	10858.6	1775697.2	0.0	0.0	8716.0	1420962.6			46.1	7510.2	8762.1	1428472.7	3204169.9	
RO0152000	PA PAU DARCO	9771.5	1594766.3	0.0	0.0	7968.6	1299074.3					7968.6	1299074.3	2893840.6	
RO0153000	PA RIBEIRÃO	6591.1	1075552.3	0.0	0.0	6309.4	1028318.8					6309.4	1028318.8	2103871.1	
RO0156000	PDS PORTO SEGURO	482.5	78967.0	0.0	0.0	981.2	160375.1					981.2	160375.1	239342.1	27.7
RO0157000	PA MIGRANTES	1648.7	272830.0	0.0	0.0	368.7	60721.4			6.3	1027.1	375.0	61748.5	334578.5	

RO0158000	PA ALTAMIRA	7389.6	1219693.1	0.0	0.0	2542.2	416156.8			2542.2	416156.8	1635849.9	
RO0159000	PA SANTA ELISA	5992.9	1019151.6	0.0	0.0	846.8	141782.7	241.1	43293.5	1087.8	185076.1	1204227.7	
RO0161000	PA PAULO FREIRE	1288.1	206217.3	0.0	0.0	577.3	93851.9			577.3	93851.9	300069.2	
RO0162000	PA ANTONIO CONSELHEIRO	511.9	83426.4	0.0	0.0	445.6	72613.0			445.6	72613.0	156039.4	
RO0163000	PAF JEQUITIBÁ	18871.6	3203063.7	0.0	0.0	118313.9	20095972.4			118313.9	20095972.4	23299036.1	0.0
RO0164000	PDS ROLIM DE MOURA DO GUAPORÉ	825.9	127112.0	0.0	0.0	1003.7	169120.1	699.5	53677.1	1703.3	222797.2	349909.2	31.3
RO0165000	PA BOA ESPERANÇA	283.8	23881.7	0.0	0.0	32.8	1631.6	174.6	5963.5	207.4	7595.1	31476.8	
RO0166000	PA RIO SÃO PEDRO	1261.0	188569.4	0.0	0.0	646.8	92281.8	83.5	2136.6	730.3	94418.4	282987.8	
RO0167000	PA LAMARQUINHA	405.4	66205.9	0.0	0.0	86.2	14059.8			86.2	14059.8	80265.7	
RO0168000	PDS DOM XAVIER REY	213.4	37085.6	0.0	0.0	3300.0	572437.5	23025.8	3975318.8	26325.8	4547756.3	4584841.9	3663.9
RO0170000	PA NOVO PROGRESSO	404.0	66626.9	0.0	0.0	50.2	8183.3			50.2	8183.3	74810.2	
RO0171000	PA LAMARCA	798.9	130204.8	0.0	0.0	218.3	35582.1			218.3	35582.1	165786.9	
RO0172000	PA FLOR DO AMAZONAS 1	4023.1	708805.8	0.0	0.0	6770.9	1193326.0			6770.9	1193326.0	1902131.8	
RO0173000	PA FLOR DO AMAZONAS 2	1628.6	286910.1	0.0	0.0	3535.8	623190.9			3535.8	623190.9	910101.0	
RO0174000	PA FLOR DO AMAZONAS 3	2682.3	472681.9	0.0	0.0	4418.1	778739.2			4418.1	778739.2	1251421.2	
RO0175000	PA NORTE SUL	3674.1	601546.6	0.0	0.0	1391.4	227110.5			1391.4	227110.5	828657.2	
RO0176000	PA NORTE SUL I	833.5	135919.3	0.0	0.0	495.4	80734.0			495.4	80734.0	216653.3	
RO0177000	PA PORTO MURTINHO	1360.4	235674.4	0.0	0.0	353.6	62144.7			353.6	62144.7	297819.1	
RO0178000	PA FLOR DO AMAZONAS 4	2245.9	394775.8	0.0	0.0	3573.9	629791.5			3573.9	629791.5	1024567.4	
RO0179000	PA CAMPO NOVO	526.7	86629.3	0.0	0.0	116.1	19030.5			116.1	19030.5	105659.8	
RO0180000	PA RABO DO TAMANDUÁ	3180.7	525155.0	0.0	0.0	803.7	131592.8			803.7	131592.8	656747.8	
RO0181000	PA 13 DE OUTUBRO	446.1	73090.7	0.0	0.0	70.7	11536.6			70.7	11536.6	84627.2	
RO0182000	PA UNIÃO I	1713.9	262081.3	0.0	0.0	249.9	38238.1			249.9	38238.1	300319.4	
RO0183000	PA CALADINHO	585.9	86915.9	0.0	0.0	222.1	30603.5			222.1	30603.5	117519.5	53.0
RO0188000	PA SANTA BÁRBARA	279.3	46000.8	0.0	0.0	0.9	143.4			0.9	143.4	46144.2	
RO0195000	PA VALE ENCANTADO	1474.6	246807.1	0.0	0.0	1513.4	246685.0			1513.4	246685.0	493492.2	
RO0216000	PA MARANATA	3805.7	574336.1	0.0	0.0	1774.3	271623.4			1774.3	271623.4	845959.5	

RO0217000	PA ZÉ BENTÃO	3393.4	511331.5	0.0	0.0	2250.7	344513.6					2250.7	344513.6	855845.1	
RO0219000	PA SANTA IZABEL	175.2	30879.8	0.0	0.0	75.7	13341.2					75.7	13341.2	44221.0	
RO0221000	PA RIO AZUL	2000.3	350988.4	0.0	0.0	498.8	87839.9					498.8	87839.9	438828.3	
RO0222000	PA RIO AZUL II	1852.9	326588.2	0.0	0.0	523.7	92315.2					523.7	92315.2	418903.4	
RO0227000	PA BELO HORIZONTE	4814.9	796749.1	0.0	0.0	13259.3	2207161.8					13259.3	2207161.8	3003910.9	
RO0228000	PA MACACO PRETO	877.8	152193.4	0.0	0.0	661.1	115623.3	60.4	10144.0			721.5	125767.2	277960.6	
RO0231000	PA DOIS DE JULHO	1551.0	254317.0	0.0	0.0	2002.6	326561.7					2002.6	326561.7	580878.7	
RO0232000	PA ALBERICO CARVALHO	1159.2	174630.8	0.0	0.0							0.0	0.0	174630.8	
RO0233000	PA MARANATA II	860.8	129905.1	0.0	0.0	1.0	150.4					1.0	150.4	130055.5	
RO0234000	PA ALZIRA AUGUSTO MONTEIRO	1425.8	214790.3	0.0	0.0							0.0	0.0	214790.3	
Total in Rondônia		3280973.5	539907815.5	0.0	0.0	1054360.8	175314649.3	93158.7	14687413.2	2376.6	409613.5	1149896.1	190411676.0	730319491.5	11069.5
**RORAIMA**			0.0	0.0	0.0							0.0	0.0	0.0	
RR0003000	PAD ANAUÁ	86711.2	16107010.1	0.0	0.0	112108.8	20931216.9	438.0	81854.4	20623.9	3828552.7	133170.7	24841624.1	40948634.2	
RR0006000	PA JATAPU	71766.0	13355431.7	0.0	0.0	65524.9	12242184.8			17336.5	3239295.9	82861.4	15481480.8	28836912.4	
RR0007000	PA PAREDÃO	14521.0	2215238.2	0.0	0.0	58242.4	9019979.3	186.5	28555.6	3683.1	588775.1	62111.9	9637309.9	11852548.2	
RR0008000	PA EQUADOR	1122.1	209599.6	0.0	0.0	9469.7	1769781.4					9469.7	1769781.4	1979380.9	
RR0009000	PA TEPEQUÉM	4227.9	704470.2	0.0	0.0	25074.8	4340464.0	1898.2	348080.1	5664.4	948707.2	32637.4	5637251.4	6341721.5	12.3
RR0010000	PA JUNDIÁ	1600.1	297522.5	0.0	0.0	336.9	62823.7	1056.0	191617.9	10228.9	1901556.0	11621.8	2155997.6	2453520.2	79.6
RR0011000	PA LADEIRÃO	1329.0	247618.7	0.0	0.0	16763.1	3131373.4	365.6	68328.3	26.1	4868.9	17154.7	3204570.6	3452189.4	
RR0013000	PA INTEGRAÇÃO	10802.7	2014935.4	0.0	0.0	16088.9	3006626.0			170.5	31856.5	16259.4	3038482.4	5053417.8	
RR0014000	PA RR-170	10620.6	1978442.9	0.0	0.0	16782.2	3136192.7	8472.1	1582409.7	5020.1	938178.0	30274.3	5656780.5	7635223.4	
RR0015000	PA ITA	2381.3	442795.6	0.0	0.0	4381.2	818689.2	318.4	59503.8	3129.3	584623.3	7828.9	1462816.3	1905611.9	
RR0016000	PA JAPÃO	7518.7	1324255.9	0.0	0.0	5892.3	984059.9	264.9	49509.9	7287.9	1311336.2	13445.2	2344906.1	3669162.0	
RR0017000	PA NOVO PARAISO	503.7	94127.9	0.0	0.0	1852.3	346176.2	5773.8	1079064.2	1729.0	323130.1	9355.1	1748370.5	1842498.4	
RR0018000	PA CUJUBIM	3157.4	530866.9	0.0	0.0	4151.4	771409.8	539.6	52451.9	6341.5	1180835.0	11032.5	2004696.7	2535563.6	
RR0020000	PA VILA NOVA	5096.4	816981.8	0.0	0.0	10149.9	1644608.5	57.1	9304.4	6.2	1014.8	10213.2	1654927.7	2471909.5	
RR0021000	PA SERRA DOURADA	6662.0	1242769.1	0.0	0.0	12038.1	2247855.6	8.6	1610.4	6284.5	1172330.3	18331.1	3421796.3	4664565.4	

RR0022000	PA QUITAUAÚ	6950.1	1059265.1	0.0	0.0	7027.5	1078669.5			3041.5	464047.3	10069.0	1542716.8	2601982.0	
RR0023000	PA SÃO JOSE	1783.1	304472.0	0.0	0.0	323.7	53742.4			2204.8	364044.9	2528.5	417787.3	722259.3	
RR0024000	PA SAMAUMA	19388.3	3107716.5	0.0	0.0	29457.2	4759696.3	624.0	44113.6	136.4	22233.3	30217.7	4826043.2	7933759.6	13.5
RR0025000	PA TABOCA	5551.4	1037395.0	0.0	0.0	7996.4	1494440.2	18.4	2135.7	7463.4	1394838.2	15478.2	2891414.1	3928809.0	
RR0026000	PA MARANHÃO	6878.4	1090291.1	0.0	0.0	7130.3	1157051.6	21.8	3913.3	5043.1	792161.8	12195.2	1953126.7	3043417.7	
RR0027000	PA ESPERANÇA	1484.6	277394.4	0.0	0.0	2062.6	385469.7			370.8	69286.3	2433.4	454756.0	732150.3	
RR0028000	PA RIO DIAS	1707.7	319154.7	0.0	0.0	3625.9	676686.5	7.8	296.4	3960.4	734333.0	7594.1	1411315.9	1730470.6	
RR0029000	PA UNIÃO	4527.2	846009.3	0.0	0.0	9194.6	1718308.1	6.5	1209.8	4155.1	776541.3	13356.2	2496059.2	3342068.5	
RR0030000	PA BOM SUCESSO	7212.5	1344153.8	0.0	0.0	11414.1	2132847.2			695.2	129924.7	12109.4	2262771.9	3606925.7	
RR0031000	PA CAXIAS	9802.6	1825844.6	0.0	0.0	14012.4	2618368.6			11095.6	2073478.7	25107.9	4691847.3	6517692.0	
RR0032000	PA SÃO LUIZÃO	5087.0	949672.1	0.0	0.0	5035.1	940985.7			2782.6	520039.6	7817.7	1461025.4	2410697.5	
RR0033000	PA MASSARANDUBA	10491.0	1759216.8	0.0	0.0	20910.7	3476508.9	642.7	119662.6	4375.3	787339.7	25928.7	4383511.3	6142728.1	
RR0034000	PA VILENA	1599.6	298945.4	0.0	0.0	2471.6	461919.5	827.5	154651.1	4546.0	849604.3	7845.1	1466174.9	1765120.3	
RR0035000	PA BOM JESUS	1759.5	286187.6	0.0	0.0	11815.9	1866264.6	2315.3	356146.5	1729.0	268524.6	15860.3	2490935.7	2777123.3	5.6
RR0036000	PA AMAJARI	1061.7	162545.1	0.0	0.0	14194.3	2179275.5	2230.2	341742.2	693.3	106537.9	17117.8	2627555.6	2790100.8	355.9
RR0037000	PA NOVA AMAZONIA	0.0	0.0	0.0	0.0	51.4	6013.1	35216.9	948434.9	1.1	123.3	35269.4	954571.4	954571.4	177.7
RR0038000	PA JACAMIM	288.9	50402.2	0.0	0.0	587.4	109202.8			1666.3	309034.1	2253.7	418236.9	468639.1	
RR0039000	PA TATAJUBA	2011.1	375860.9	0.0	0.0	13064.1	2441544.8			7709.3	1440791.5	20773.4	3882336.3	4258197.1	
RR0040000	PA AJARANI	7382.2	1241455.8	0.0	0.0	82059.7	14160027.6	91.1	15771.1	34871.8	5917482.7	117022.6	20093281.4	21334737.2	
RR0041000	PA PAU RAINHA	671.3	125455.1	0.0	0.0	10690.7	1997980.5	6564.5	1226833.6	468.2	87494.5	17723.3	3312308.6	3437763.7	
RR0042000	PA CUPIUBA	761.4	142295.3	0.0	0.0	7259.1	1356658.4	400.9	74925.9	5224.5	976414.0	12884.6	2407998.3	2550293.7	
RR0043000	PA CASTANHEIRA	353.4	66055.4	0.0	0.0	5479.8	1024125.4	1511.0	282397.9	4488.3	838815.6	11479.2	2145339.0	2211394.4	
RR0044000	PA ANGELIN	361.6	67580.7	0.0	0.0	1249.6	233528.9	53.2	9947.2	3939.8	736311.3	5242.6	979787.3	1047368.1	
RR0045000	PA SERINGUEIRA	1327.9	248173.3	0.0	0.0	10167.1	1900137.1	974.8	182187.7	623.5	116519.8	11765.4	2198844.6	2447017.9	
RR0046000	PA JATOBA	1689.2	315694.1	0.0	0.0	11954.5	2234180.0			5936.4	1109457.4	17890.9	3343637.4	3659331.5	
RR0049000	PA RENASCER	80.3	9280.2	0.0	0.0	221.5	27381.4	2097.3	22532.3	149.5	18482.4	2468.3	68396.0	77676.2	
RR0051000	PA PAU- BRASIL	68.7	12841.8	0.0	0.0	20721.9	3872708.8	3948.8	737985.4	4349.9	812960.5	29020.6	5423654.7	5436496.5	
RR0052000	PA TERRA NOVA	1265.5	234983.5	0.0	0.0	2023.9	376734.8			145.8	26849.1	2169.7	403583.9	638567.5	

RR0053000	PA SERRA TALHADA	697.8	130414.3	0.0	0.0	1215.4	227136.9			1335.7	249634.9	2551.1	476771.8	607186.1	
RR0054000	PA ARCO-ÍRIS	348.5	59861.0	0.0	0.0	8518.9	1556489.4	1310.0	244803.3	5736.8	1063262.9	15565.7	2864555.6	2924416.7	
RR0055000	PA TALISMÃ	133.1	23901.9	0.0	0.0	1661.1	310214.8	421.7	78807.8	513.2	95909.3	2595.9	484931.8	508833.7	
RR0056000	PA NOVA FLORESTA	649.3	114155.7	0.0	0.0	6766.2	1206331.4	183.4	34051.5	1033.8	173165.7	7983.4	1413548.5	1527704.2	
RR0073000	PA ALTO ARRAIA	85.8	7777.8	0.0	0.0	555.3	70250.9	1124.4	18819.6	382.5	42987.2	2062.1	132057.7	139835.5	
	Total in Roraima	331480.7	59476518.9	0.0	0.0	689776.5	122564323.0	79971.1	8453659.9	218400.8	39423692.0	988148.3	170441674.9	229918193.8	644.6
**TOCANTINS**			0.0	0.0	0.0							0.0	0.0	0.0	
TO0004000	PA AMARRIO	0.0	0.0	1403.3	46295.4			967.5	31916.3			967.5	31916.3	78211.7	
TO0005000	PA SÃO JOÃO	0.0	0.0	1308.4	51844.2			2775.7	132332.4			2775.7	132332.4	184176.5	
TO0006000	PA CACHOEIRA	0.0	0.0	3376.8	111400.7			1399.7	46174.8			1399.7	46174.8	157575.5	
TO0007000	PA CAMARÃO	1027.6	153940.7	212.7	31862.0	224.4	33616.3	89.3	13383.6			313.7	46999.9	232802.6	
TO0008000	PA MONTES ALTOS	353.8	46827.7	29.4	4407.2	113.7	17033.0					113.7	17033.0	68268.0	
TO0009000	PA ALEGRE	1650.7	269009.2	0.0	0.0	8.3	1346.1					8.3	1346.1	270355.3	
TO0010000	PA SÃO PEDRO	0.0	0.0	3739.1	123353.1			1808.5	59663.6			1808.5	59663.6	183016.8	
TO0011000	PA GROTÃO	1685.9	173686.9	13.5	2025.3	31.0	4641.9					31.0	4641.9	180354.2	
TO0012000	PA BARROCA	503.9	16624.7	620.5	20470.2	217.6	7180.0	1327.2	43784.0			1544.8	50964.0	88058.9	
TO0013000	PA SÃO SALVADOR	0.0	0.0	843.0	40573.1			874.4	45530.3			874.4	45530.3	86103.4	
TO0014000	PA SERRA TAQUARUÇU	0.0	0.0	421.4	15090.8			1574.4	53953.9			1574.4	53953.9	69044.6	
TO0015000	PA LAGOÃO	0.0	0.0	7518.8	422664.0	0.0	0.6	3930.2	244230.7			3930.2	244231.3	666895.3	
TO0016000	PA JUARINA	26961.6	4519599.5	0.0	0.0	382.8	66750.5	3428.5	561743.6			3811.3	628494.1	5148093.6	7.1
TO0017000	PA PONTAL	590.4	86054.5	37.7	5646.7	218.2	32670.1					218.2	32670.1	124371.4	
TO0018000	PA TOBASA	1629.7	304578.6	0.0	0.0	85.3	15940.6	194.5	36323.3			279.8	52264.0	356842.6	
TO0019000	PA SANTO ANTÔNIO	0.0	0.0	2534.6	83617.8			909.2	29993.7			909.2	29993.7	113611.5	
TO0020000	PA BAIAO	0.0	0.0	1345.4	48234.4			4567.3	180612.5			4567.3	180612.5	228846.9	
TO0021000	PA SÃO JORGE	1871.1	234757.4	43.0	5285.4	84.2	12615.1					84.2	12615.1	252657.8	
TO0022000	PA PRAIA NORTE	2942.5	441222.9	714.6	107058.4	507.5	76021.9	363.6	54476.3			871.1	130498.1	678779.5	7.8
TO0023000	PA SANTA CRUZ II	9985.9	1866112.2	0.0	0.0	242.7	45362.1	314.7	58816.3			557.4	104178.4	1970290.6	0.1

TO0024000	PA CAMARÃO II	3067.4	459531.3	173.8	26033.3	837.7	125494.4	112.0	16779.7	949.7	142274.0	627838.6	
TO0025000	PA TRÊCHO SECO	844.2	154615.0	0.0	0.0					0.0	0.0	154615.0	
TO0026000	PA JUARI	4755.4	736209.6	0.0	0.0	47.9	7825.8			47.9	7825.8	744035.5	
TO0027000	PA ESPERANTINA	1031.8	192839.7	0.0	0.0	17.2	3222.6	558.9	104462.1	576.2	107684.7	300524.4	
TO0028000	PA JENIPAPO	1271.6	212570.9	0.0	0.0					0.0	0.0	212570.9	
TO0029000	PA ARAGUAIALA	164.5	30752.7	0.0	0.0	26.0	4855.9	2035.7	380458.3	2061.7	385314.1	416066.9	15.2
TO0031000	PA BANDEIRANTES	847.6	112988.6	0.0	0.0					0.0	0.0	112988.6	
TO0032000	PA PENHA	0.0	0.0	18415.5	644947.1			12634.4	529668.7	12634.4	529668.7	1174615.8	10.7
TO0034000	PA REVOLUÇÃO	0.0	0.0	900.1	30398.7			1607.7	53969.2	1607.7	53969.2	84367.9	
TO0035000	PA BOA ESPERANÇA	1799.4	336291.1	0.0	0.0	140.7	26303.9	509.2	94643.2	650.0	120947.1	457238.2	19.6
TO0036000	PA LAGO PRETO	383.8	71724.1	0.0	0.0			2072.6	387355.8	2072.6	387355.8	459079.9	52.3
TO0037000	PA OURO VERDE	5476.4	1022370.6	0.0	0.0	255.2	47687.3			255.2	47687.3	1070057.9	
TO0038000	PA BANDEIRANTE	0.0	0.0	852.9	65877.1			192.5	14872.5	192.5	14872.5	80749.6	
TO0039000	PA DOIS RIACHOS	4915.2	825219.0	0.0	0.0	78.7	13220.3			78.7	13220.3	838439.3	4.5
TO0040000	PA ALMECEGAS	0.0	0.0	1257.7	45467.7			609.0	33454.1	609.0	33454.1	78921.8	182.7
TO0041000	PA RIO PRETO	4173.0	428312.2	0.0	0.0	867.9	70411.6	4851.2	377962.5	5719.2	448374.1	876686.3	
TO0042000	PA MULATOS	2413.7	451097.0	0.0	0.0	125.7	23499.8			125.7	23499.8	474596.8	
TO0043000	PA AREIAS	0.0	0.0	734.5	24230.2			1981.4	65365.4	1981.4	65365.4	89595.7	
TO0044000	PA CABECEIRA DO PRATA	0.0	0.0	182.1	6007.5			1.2	40.9	1.2	40.9	6048.4	
TO0045000	PA CAIAPOZINHO	0.0	0.0	436.2	15666.6			1645.7	67484.0	1645.7	67484.0	83150.6	
TO0046000	PA MURICIZAL	5364.9	900184.9	0.0	0.0	110.5	18454.9			110.5	18454.9	918639.9	
TO0047000	PA ANDORINHA	3564.7	583643.0	0.0	0.0	169.8	28463.1			169.8	28463.1	612106.0	
TO0048000	PA CAJUEIRO	976.7	159169.7	76.9	12531.6	126.6	20624.2	860.2	140188.8	986.8	160813.0	332514.3	
TO0049000	PA CUPIM	0.0	0.0	1133.8	165563.2	0.5	75.3	4212.7	594755.3	4213.2	594830.6	760393.8	
TO0050000	PA MATA BONITA I	194.2	29116.1	0.0	0.0					0.0	0.0	29116.1	1.6
TO0052000	PA SURUBIM	0.0	0.0	351.5	11597.0			1349.8	44529.2	1349.8	44529.2	56126.2	
TO0054000	PA UNIÃO	0.0	0.0	418.0	5338.4			1233.3	20166.6	1233.3	20166.6	25505.0	
TO0055000	PA EXTREMA	2319.2	377963.6	0.0	0.0	241.0	39283.0			241.0	39283.0	417246.5	

TO0056000	PA BICO DO PAPAGAIO	228.3	42664.0	0.0	0.0			688.1	122749.5			688.1	122749.5	165413.5	24.6
TO0058000	PA TOCANTINS	0.0	0.0	0.0	0.0			1162.9	215116.1			1162.9	215116.1	215116.1	40.1
TO0068000	PA ÁGUA LIMPA	734.7	136149.3	0.0	0.0	43.9	8213.1					43.9	8213.1	144362.4	
TO0069000	PA CAPELINHA	0.0	0.0	1409.3	46494.4			273.6	9027.4			273.6	9027.4	55521.7	
TO0070000	PA NOVA VIDA	477.8	77560.3	0.0	0.0	1.0	155.4					1.0	155.4	77715.7	
TO0071000	PA RETIRO	0.0	0.0	401.6	11807.5			788.2	30274.2			788.2	30274.2	42081.7	307.2
TO0072000	PA RONCA	2760.8	449928.4	38.4	6260.0	603.0	98265.2	114.2	18606.5			717.1	116871.7	573060.1	
TO0073000	PA SÃO JORGE II	752.7	84822.6	13.7	2057.1	99.7	14940.7					99.7	14940.7	101820.4	
TO0074000	PA JACUBINHA	0.0	0.0	639.5	53302.9			5679.8	346324.0			5679.8	346324.0	399626.9	
TO0076000	PA SUDAN	4131.9	675636.7	0.0	0.0	0.1	4.7	1394.9	108384.7			1395.0	108389.4	784026.1	
TO0077000	PA FILADÉLFIA	3197.3	536786.4	0.0	0.0							0.0	0.0	536786.4	
TO0078000	PA TAMBORIL	1154.9	188209.0	112.2	18287.9	34.0	5543.2	2703.2	440545.7			2737.2	446088.9	652585.8	
TO0079000	PA RIACHINHO	2958.6	482165.6	0.0	0.0	108.6	17703.6	9.7	1582.1			118.3	19285.7	501451.3	
TO0080000	PA COLORADO	2486.9	405282.9	0.0	0.0	90.7	14787.5					90.7	14787.5	420070.4	
TO0081000	PA CASA DO MORRO	1749.1	285049.0	0.0	0.0	191.4	31188.1					191.4	31188.1	316237.1	
TO0083000	PA REIS	4215.9	632530.8	206.2	30904.3	653.0	97908.2	705.4	108946.3			1358.4	206854.5	870289.5	
TO0084000	PA PORTELA	450.5	84187.2	0.0	0.0	65.7	12276.6	590.1	108896.4			655.8	121173.0	205360.2	0.2
TO0085000	PA RIO PRATA	0.0	0.0	2124.5	70086.3			527.2	17393.4			527.2	17393.4	87479.7	
TO0086000	PA ARAGUAMINAS	299.0	9865.4	503.8	16620.9	28.3	934.1	1555.4	51314.1			1583.8	52248.2	78734.6	
TO0087000	PA LOROTY	3092.9	422332.0	6431.1	386072.8	3986.6	558872.4	25821.2	787890.8			29807.7	1346763.2	2155168.1	291.7
TO0088000	PA SÃO PAULO	0.0	0.0	2180.9	351075.8			2607.4	418658.9			2607.4	418658.9	769734.7	
TO0089000	PA SÃO JOSÉ	3102.9	579309.2	0.0	0.0							0.0	0.0	579309.2	
TO0090000	PA SÃO FRANCISCO	1196.4	179231.2	81.0	12127.4	522.0	78194.3					522.0	78194.3	269552.9	
TO0091000	PA VENTURA	7152.6	1162755.5	39.0	5982.2	499.7	79598.0			30.2	4029.7	529.9	83627.7	1252365.4	
TO0092000	PA UNIÃO II	0.0	0.0	595.2	19630.5			689.3	22739.1			689.3	22739.1	42369.5	
TO0093000	PA SANTA MARTA	2288.9	373016.1	0.0	0.0	281.7	45908.4					281.7	45908.4	418924.5	
TO0094000	PA MANTIQUEIRA	1473.5	240130.4	0.0	0.0	443.5	72282.4					443.5	72282.4	312412.8	
TO0095000	PA BAVIERA	3126.9	515646.6	0.0	0.0	1471.8	240295.8					1471.8	240295.8	755942.4	

TO0096000	PA NOSSA SENHORA APARECIDA	2099.9	325443.5	0.0	0.0	135.3	22282.1					135.3	22282.1	347725.7	
TO0097000	PA ATANASIO	2504.7	400060.5	0.0	0.0	368.1	59868.0					368.1	59868.0	459928.6	22.6
TO0098000	PA MARCOS FREIRE	2485.8	398299.0	0.0	0.0	200.3	32471.4					200.3	32471.4	430770.4	
TO0099000	PA PADRE JOSIMO	1523.6	248301.6	0.0	0.0	86.4	14073.3	2.6	426.9			89.0	14500.2	262801.8	
TO0100000	PA DONA EUNICE	2242.2	365087.6	0.0	0.0	192.7	31350.0					192.7	31350.0	396437.6	0.0
TO0101000	PA GAMELEIRA	0.0	0.0	5514.5	103426.0	0.0	0.6	4511.4	89452.3			4511.4	89452.9	192879.0	
TO0102000	PA BARONESA	0.0	0.0	375.7	12392.8			1223.7	40371.1			1223.7	40371.1	52764.0	
TO0103000	PA VERA CRUZ	2033.4	215515.6	0.0	0.0	207.7	16986.5					207.7	16986.5	232502.2	
TO0104000	PA AGUA BRANCA	2076.5	97569.2	3275.9	109515.4	96.3	3177.4	25110.1	846503.2			25206.4	849680.6	1056765.2	
TO0105000	PA SANTA HELENA	525.9	98288.0	0.0	0.0	23.9	4465.1					23.9	4465.1	102753.1	1.1
TO0106000	PA CARACOL	1369.5	196977.6	4736.6	145255.3	2174.8	309438.2	8738.4	246900.9			10913.2	556339.1	898572.0	43.3
TO0107000	PA PALMEIRAS	1066.6	118153.4	670.0	40484.6	170.0	23763.0	125.1	9373.2			295.1	33136.3	191774.2	
TO0108000	PA ÁGUA FRIA	0.0	0.0	434.5	14334.2			785.5	27091.4			785.5	27091.4	41425.6	
TO0109000	PA GROTA DO LAGE	1994.1	322103.9	0.0	0.0	43.8	7349.9	877.2	93805.4	15.0	2513.4	935.9	103668.6	425772.5	
TO0110000	PA SÃO MIGUEL	0.0	0.0	314.4	5592.9			2104.2	39195.2			2104.2	39195.2	44788.0	
TO0111000	PA MUTIRÃO	1628.4	282749.6	0.0	0.0							0.0	0.0	282749.6	
TO0112000	PA TRANSARAGUAIA	836.5	136330.0	232.4	37876.3	26.2	4267.0	722.9	117817.8			749.1	122084.7	296290.9	
TO0113000	PA PROFESSORA DJANIRA	1409.5	229712.8	0.0	0.0							0.0	0.0	229712.8	
TO0114000	PA SANTA CLARA	161.8	11281.9	2082.2	84714.8	357.2	32854.7	9717.4	400721.4			10074.6	433576.1	529572.8	26.3
TO0115000	PA SÃO LUIZ	0.0	0.0	2901.2	96906.1			922.8	36975.3			922.8	36975.3	133881.4	
TO0116000	PA BOA SORTE	1088.7	193671.8	265.3	43233.0			55.1	9091.6			55.1	9091.6	245996.4	
TO0117000	PA RANCHO ALEGRE	1085.6	162637.9	8.6	1399.3	232.2	34857.3	300.7	47224.7			532.9	82082.0	246119.2	
TO0118000	PA VINICIUS	1066.7	111149.8	265.0	41809.2	104.4	12729.3	1749.9	279744.5			1854.2	292473.8	445432.8	
TO0120000	PA FIRMEZA	0.0	0.0	364.8	12128.2			779.0	27260.0			779.0	27260.0	39388.1	
TO0124000	PA DOIS CORAÇıES	2817.5	468443.1	0.0	0.0	281.7	46138.5					281.7	46138.5	514581.6	
TO0125000	PA PIRARUCU	0.0	0.0	3910.9	132003.5			2513.5	87128.6			2513.5	87128.6	219132.1	
TO0126000	PA TRÊS PODERES	0.0	0.0	1684.7	41992.6			430.7	29508.3			430.7	29508.3	71500.9	63.0
TO0127000	PA MARINGA	3079.2	511315.6	12.2	1984.2	176.1	28698.6					176.1	28698.6	541998.5	

TO0128000	PA TRÊS IRMÃOS	4373.9	712193.1	24.1	3928.0	290.7	47371.9			290.7	47371.9	763493.0	
TO0129000	PA SÃO SILVESTRE	2445.9	382036.5	12.5	2017.5	45.2	7369.5			45.2	7369.5	391423.5	
TO0130000	PA SANTA JULIANA	2548.3	398362.4	57.2	8757.0	414.3	62183.8	9.3	1520.0	423.6	63703.8	470823.2	
TO0131000	PA CANAÃ	1121.2	208189.2	0.0	0.0					0.0	0.0	208189.2	
TO0132000	PA PETRÔNIO	621.0	101199.1	0.0	0.0					0.0	0.0	101199.1	
TO0133000	PA NAJÁ	678.6	106793.3	2.9	455.0	2.0	309.5			2.0	309.5	107557.8	
TO0134000	PA FAVEIRA	0.0	0.0	244.8	18688.5			976.7	68944.9	976.7	68944.9	87633.4	35.6
TO0135000	PA REAL	1029.8	94548.4	0.0	0.0	12.7	1397.4			12.7	1397.4	95945.8	
TO0136000	PA ESTRELA DALVA	0.0	0.0	774.7	25116.6			1176.0	45439.4	1176.0	45439.4	70556.0	
TO0138000	PA PERICATU	0.0	0.0	3244.9	92167.5			3503.3	109209.4	3503.3	109209.4	201376.9	
TO0139000	PA BARRANCO DO MUNDO	0.0	0.0	915.4	49670.2			3918.0	146875.5	3918.0	146875.5	196545.7	0.1
TO0140000	PA TRÊS LAGOAS	0.0	0.0	1302.4	44581.7			603.0	41756.6	603.0	41756.6	86338.3	
TO0141000	PA PRIMOGÊNITO	0.0	0.0	1423.1	54761.8			1830.3	96027.3	1830.3	96027.3	150789.0	
TO0142000	PA TARUMÃ	94.1	11047.2	3657.8	80003.1	816.9	84691.9	3722.4	106799.0	4539.3	191491.0	282541.2	
TO0143000	PA REUNIDAS	14136.3	2322286.2	0.0	0.0	2231.5	363779.8	108.2	17630.9	2339.6	381410.6	2703696.9	0.4
TO0144000	PA PROVIDÊNCIA	11731.5	1907833.2	0.0	0.0	206.8	34253.3			206.8	34253.3	1942086.5	
TO0146000	PA COCAL	1547.5	230878.3	5.1	767.5	116.0	17382.1	205.5	30785.9	321.5	48168.1	279813.8	
TO0147000	PA SANTA LUZIA	0.0	0.0	712.8	55945.6			879.2	112185.7	879.2	112185.7	168131.3	
TO0148000	PA PEDRA BRANCA	555.7	90367.6	89.5	14571.9	1542.5	251363.9	1214.8	188371.6	2757.3	439735.5	544675.0	
TO0149000	PA ARAGUAIA	0.0	0.0	1121.0	44719.0	562.2	76663.0	803.1	36093.4	1365.3	112756.4	157475.4	119.5
TO0150000	PA VITORIA RÉGIA	4455.6	726124.1	0.0	0.0	1993.0	324801.3	1585.3	258354.8	3578.3	583156.0	1309280.1	20.8
TO0151000	PA ALEGRIA	0.0	0.0	35.1	601.9			74.1	1272.2	74.1	1272.2	1874.1	
TO0152000	PA COSTA RICA	2447.5	398873.0	19.9	3239.8	1416.6	230869.6	2724.5	444012.2	4141.1	674881.8	1076994.7	
TO0153000	PA TOLEDO II	1.6	221.3	607.6	21303.8	320.3	43326.1	899.9	41834.9	1220.2	85161.0	106686.0	20.8
TO0154000	PA MUIRAQUITAN	45.8	4260.0	1920.3	18153.3	501.3	68216.4	2573.6	26597.1	3074.9	94813.6	117226.8	
TO0155000	PA CONSOLAÇÃO	0.0	0.0	1266.9	42515.4			784.5	35890.1	784.5	35890.1	78405.5	
TO0156000	PA CALIFÓRNIA	985.0	93313.6	246.7	23352.3	1418.0	200428.2	263.2	9342.1	1681.2	209770.3	326436.2	
TO0157000	PA RECANTO	1653.0	122603.6	0.0	0.0	434.3	31856.4			434.3	31856.4	154460.0	

TO0158000	PA CAÇADOR	1816.8	145144.3	0.0	0.0	1209.7	93547.4					1209.7	93547.4	238691.7	
TO0159000	PA LIMEIRA	2940.0	390602.7	0.0	0.0	455.6	45640.0					455.6	45640.0	436242.7	2.6
TO0160000	PA 1░DE JANEIRO	0.0	0.0	1217.7	66552.1			2893.1	167794.0			2893.1	167794.0	234346.1	
TO0162000	PA NOVA ESTRELA	623.5	115862.1	0.0	0.0			1283.1	232607.3			1283.1	232607.3	348469.4	1.3
TO0163000	PA BURITIS	1988.1	319867.4	7.7	1159.6	187.9	28160.9					187.9	28160.9	349188.0	
TO0164000	PA BOA SORTE II	884.6	125510.0	39.5	5925.7	24.7	3702.4					24.7	3702.4	135138.2	
TO0165000	PA SANTA BARBARA	983.6	145132.1	14.9	2237.7	72.2	10822.6					72.2	10822.6	158192.4	
TO0166000	PA PINGO D ÁGUA	1600.1	298827.5	0.0	0.0			1494.3	275227.5			1494.3	275227.5	574055.1	15.2
TO0167000	PA SÃO ROQUE	2118.8	344875.9	0.0	0.0	3.3	539.2					3.3	539.2	345415.1	
TO0168000	PA ÁGUA FRIA II	0.0	0.0	2626.1	89170.5			2999.0	126194.7			2999.0	126194.7	215365.2	
TO0171000	PA SÃO LUCAS	859.3	135954.8	18.5	2950.9	46.9	7055.2					46.9	7055.2	145960.9	
TO0172000	PA ALTO BONITO	334.8	62350.6	0.0	0.0							0.0	0.0	62350.6	
TO0173000	PA PIRACEMA	4519.0	661378.0	331.3	46359.7	3827.6	541613.0	1318.9	103225.4			5146.5	644838.3	1352576.0	
TO0174000	PA TOLEDO I	0.0	0.0	1115.6	36803.5			89.6	2954.3			89.6	2954.3	39757.8	
TO0175000	PA MANCHETE	214.9	22180.6	8689.4	157431.4	1863.9	222033.7	13575.9	370280.1			15439.8	592313.7	771925.7	241.8
TO0176000	PA TALISMÃ	0.0	0.0	2496.4	82354.7			1622.8	53537.5			1622.8	53537.5	135892.2	
TO0177000	PA LAGOA DA ONÇA	314.6	23448.6	9288.9	483333.7	27.9	3172.6	7711.7	365690.6			7739.6	368863.2	875645.5	
TO0178000	PA DALILA	4144.6	676624.2	0.0	0.0	387.2	63112.6					387.2	63112.6	739736.8	
TO0179000	PA REMANSO	0.0	0.0	249.3	6284.6			1922.7	61172.7			1922.7	61172.7	67457.2	18.7
TO0180000	PA RECREIO	0.0	0.0	676.7	97944.3			681.1	69567.6			681.1	69567.6	167511.9	
TO0181000	PA INHUMA	3349.1	545915.2	0.0	0.0	428.1	69775.0			32.6	5307.5	460.7	75082.6	620997.8	
TO0182000	PA BANANAL	0.0	0.0	1230.9	40606.1			1001.6	33044.4			1001.6	33044.4	73650.5	2.0
TO0183000	PA RESTINGA	51.4	9611.7	0.0	0.0	0.0	4.1	283.9	53062.0			283.9	53066.1	62677.8	
TO0184000	PA VITORIA	2440.7	397397.4	0.0	0.0	78.0	12709.1					78.0	12709.1	410106.4	
TO0185000	PA SOLEDADE	802.2	115985.7	482.3	32239.4	159.9	25898.0	873.3	40804.0			1033.2	66702.0	214927.2	
TO0186000	PA AMIGOS DA TERRA	0.0	0.0	1502.8	243731.5			7306.5	1175891.7			7306.5	1175891.7	1419623.2	
TO0187000	PA BARRA BONITA	2477.8	209449.0	43.3	3582.0	210.9	16288.3			64.0	4940.4	274.8	21228.7	234259.7	
TO0188000	PA PROGRESSO	8016.1	766661.2	0.0	0.0	73.2	11890.3					73.2	11890.3	778551.5	

TO0189000	PA CRISTAL	0.0	0.0	1057.1	34873.0			2762.1	93557.1	2762.1	93557.1	128430.1	23.4
TO0190000	PA CRISTO REI	959.9	161571.1	0.0	0.0	47.1	8172.1			47.1	8172.1	169743.2	
TO0191000	PA SANTA FÉ	888.6	69260.6	0.0	0.0	3.7	289.5			3.7	289.5	69550.1	
TO0192000	PA SÃO JOÃO BATISTA	1219.9	81942.1	0.0	0.0	150.4	5179.1			150.4	5179.1	87121.2	
TO0193000	PA FLORESTA	0.0	0.0	941.2	47673.9			2015.9	84841.5	2015.9	84841.5	132515.4	
TO0194000	PA ENTRE RIOS	0.0	0.0	921.1	35036.4			2233.7	82344.9	2233.7	82344.9	117381.3	
TO0195000	PA CAPIVARA	0.0	0.0	860.8	23789.9			875.1	27883.8	875.1	27883.8	51673.8	
TO0196000	PA BREJINHO	0.0	0.0	1426.7	47066.8			1253.0	41337.1	1253.0	41337.1	88403.9	
TO0197000	PA ITIMIRIM	0.0	0.0	1238.0	40842.0			1153.9	38067.6	1153.9	38067.6	78909.6	
TO0198000	PA COIMBRA	0.0	0.0	4147.2	144357.6			1068.7	35862.3	1068.7	35862.3	180219.9	
TO0199000	PA SANTA TEREZA	0.0	0.0	546.4	18026.5			1220.0	40247.1	1220.0	40247.1	58273.6	
TO0200000	PA TALISMÃ II	0.0	0.0	1445.3	47680.7			1165.7	38457.0	1165.7	38457.0	86137.7	4.5
TO0201000	PA CONQUISTA	2205.3	190029.1	0.0	0.0	9.0	725.2			9.0	725.2	190754.3	
TO0202000	PA PÉ DO MORRO	0.0	0.0	462.1	5249.7			1088.9	23808.0	1088.9	23808.0	29057.7	
TO0203000	PA SÃO JUDAS TADEU	1730.0	219589.2	5615.2	216249.9	749.0	94604.1	16796.3	777815.3	17545.3	872419.4	1308258.5	1.6
TO0204000	PA CORREGO FUNDO	0.0	0.0	824.5	27199.0			1327.0	43776.5	1327.0	43776.5	70975.5	
TO0205000	PA NOVO PLANO	0.0	0.0	85.8	1993.5			1173.9	32041.1	1173.9	32041.1	34034.5	
TO0206000	PA PALMEIRINHA	0.0	0.0	837.4	34450.5			237.7	17297.1	237.7	17297.1	51747.5	
TO0207000	PA PAU D ARCO	0.0	0.0	1228.9	41927.7			426.4	14839.5	426.4	14839.5	56767.3	
TO0208000	PA PROGRESSO II	0.0	0.0	394.9	13127.5			890.5	32646.3	890.5	32646.3	45773.8	
TO0209000	PA BELENZINHO	363.5	19670.1	1623.3	16666.2	50.1	6880.9	563.4	5668.2	613.6	12549.1	48885.4	
TO0210000	PA LONTRA	451.8	30505.5	18.3	172.6	65.2	8462.0	1070.0	11725.1	1135.2	20187.1	50865.2	
TO0211000	PA MATA AZUL	4489.2	639816.3	25.3	3911.3	377.2	56061.4			377.2	56061.4	699789.0	
TO0212000	PA SÃO SEBASTIÃO	2190.4	361187.0	0.0	0.0	70.1	11430.3			70.1	11430.3	372617.4	
TO0213000	PA SETECENTOS	604.5	43354.4	317.4	3128.5	243.8	31365.7	2517.6	27104.2	2761.3	58469.9	104952.8	
TO0214000	PA BABAÇU	777.2	117368.5	5.7	847.9	234.4	35115.7			234.4	35115.7	153332.1	
TO0215000	PA CHOBÓ	0.0	0.0	303.9	10025.1			2693.6	88860.6	2693.6	88860.6	98885.7	

TO0216000	PA CARAIBINHA	587.4	87995.3	15.5	2322.7	54.5	8157.5	601.6	90119.7	656.0	98277.2	188595.2	
TO0266000	PA BANDEIRANTE II	617.9	100701.6	3.8	619.5					0.0	0.0	101321.1	
TO0267000	PA SOSSEGO	468.2	87443.7	0.0	0.0					0.0	0.0	87443.7	
TO0268000	PA ITACOLOMI	1019.8	78770.9	0.0	0.0					0.0	0.0	78770.9	
TO0269000	PA MAMÉDIO	277.6	10154.5	5.2	250.0	2.0	273.1			2.0	273.1	10677.6	
TO0270000	PA SÃO JOÃO II	566.5	84870.8	35.8	5368.1	21.8	3259.6	832.3	124683.2	854.0	127942.8	218181.7	
TO0271000	PA FORMOSA	0.0	0.0	253.1	31581.1			4539.9	398061.3	4539.9	398061.3	429642.3	13.2
TO0272000	PA RETIRO II	0.0	0.0	19.4	1495.6			1051.8	80436.0	1051.8	80436.0	81931.7	
TO0273000	PA TABULEIRO	0.0	0.0	42.7	3301.8			502.2	38792.3	502.2	38792.3	42094.1	
TO0274000	PA BELA VISTA	0.0	0.0	447.0	63348.4			861.7	122453.9	861.7	122453.9	185802.3	
TO0275000	PA PACIÊNCIA	0.0	0.0	224.1	6583.0			2310.3	69569.2	2310.3	69569.2	76152.2	1.4
TO0276000	PA CACHOEIRINHA	654.3	62244.9	0.0	0.0	4.2	539.2	3.5	33.0	7.7	572.2	62817.2	
TO0277000	PA SENHOR DO BONFIM	0.0	0.0	75.6	712.4	39.3	5114.5	1560.3	29562.5	1599.6	34677.0	35389.3	
TO0278000	PA PONTÃO	298.5	55789.7	0.0	0.0					0.0	0.0	55789.7	
TO0279000	PA BASTI─O VELHO	24.0	4481.2	0.0	0.0			406.6	75976.4	406.6	75976.4	80457.6	
TO0280000	PA REMANSINHO	0.0	0.0	1376.5	26758.3			15389.4	311921.5	15389.4	311921.5	338679.8	81.8
TO0281000	PA ARLINDO	0.0	0.0	1615.6	53299.5			1116.3	36827.9	1116.3	36827.9	90127.4	
TO0282000	PA REMANSÃO	0.0	0.0	201.5	6648.5			3049.9	100616.7	3049.9	100616.7	107265.2	
TO0284000	PA SÃO GABRIEL	3449.0	566403.1	0.0	0.0	1031.9	168663.0			1031.9	168663.0	735066.2	
TO0286000	PA BREJÃO	0.0	0.0	4.8	158.5			652.6	43431.5	652.6	43431.5	43589.9	
TO0287000	PA VALE DA SERRA DA CONCEIÇÃO	161.6	25718.1	16.0	2613.7	20.5	3219.6	1515.9	246782.0	1536.3	250001.5	278333.4	
TO0288000	PA TAPIRASSU	0.0	0.0	735.5	24765.2			991.5	38673.3	991.5	38673.3	63438.4	
TO0289000	PA PIABA	0.0	0.0	493.8	16290.2			791.5	26112.5	791.5	26112.5	42402.7	
TO0290000	PA SERTÃOZINHO	0.0	0.0	463.9	15303.9			651.0	21477.0	651.0	21477.0	36780.9	
TO0291000	PA DESTILARIA	0.0	0.0	1219.4	185153.5			684.4	96672.2	684.4	96672.2	281825.7	
TO0292000	PA JURANDI BELIZÁRIO	0.0	0.0	0.0	0.0			2089.8	366162.1	2089.8	366162.1	366162.1	104.0
TO0293000	PA SÃO BENTO	173.6	27507.6	377.3	60401.6	27.7	4231.9	2213.2	360293.8	2240.9	364525.7	452434.8	
TO0294000	PA VOLTA DO RIO	0.0	0.0	1762.6	59866.7			4101.1	140811.9	4101.1	140811.9	200678.6	1.9

TO0295000	PA DUAS CABECEIRAS	0.0	0.0	252.0	19464.7			965.7	74590.6	965.7	74590.6	94055.3	
TO0296000	PA TURRÃO	0.0	0.0	489.5	37812.2			188.4	14548.9	188.4	14548.9	52361.1	
TO0297000	PA TUCUMIRIM	2954.1	400203.2	0.0	0.0	479.3	40426.6	792.9	117128.6	1272.3	157555.2	557758.5	
TO0298000	PA ARRAIAS	1232.5	190556.4	0.0	0.0	154.4	24713.4			154.4	24713.4	215269.8	
TO0299000	PA CHAPADA VERMELHA	0.0	0.0	456.1	16277.6			2014.0	80171.6	2014.0	80171.6	96449.2	
TO0300000	PA PAULO FREIRE I e II	0.0	0.0	1308.0	41746.4	2.3	73.8	3327.0	106711.0	3329.3	106784.8	148531.2	
TO0301000	PA OZIEL ALVES PEREIRA	1362.5	198218.0	2.7	424.9	309.7	46391.1	90.1	13942.9	399.8	60334.1	258976.9	
TO0302000	PA ESTRELA	603.4	98338.1	52.9	8625.1	559.9	91240.5	159.6	26009.5	719.5	117250.0	224213.2	
TO0303000	PA VARGEM GRANDE	1924.9	63503.6	147.8	4875.3	110.1	3632.7	61.4	2026.7	171.6	5659.5	74038.4	
TO0304000	PA ZÉ PEREIRA	0.0	0.0	894.5	28490.5			1352.4	41080.5	1352.4	41080.5	69571.0	0.7
TO0305000	PA COBERTÃO	0.0	0.0	1179.5	38911.1			1596.8	52677.7	1596.8	52677.7	91588.8	
TO0306000	PA VITÓRIA I	0.0	0.0	756.5	24957.6			1258.2	42291.9	1258.2	42291.9	67249.5	
TO0307000	PA VALE VERDE	0.0	0.0	851.6	86250.0			902.9	73418.9	902.9	73418.9	159669.0	
TO0308000	PA 2 DE JANEIRO	0.0	0.0	535.5	87266.7			1400.8	228179.3	1400.8	228179.3	315446.0	
TO0309000	PA ALTO ALEGRE	185.9	23999.8	78.0	891.6	448.7	61350.5	1251.7	37520.7	1700.3	98871.2	123762.6	14.8
TO0310000	PA BURITIRANA	698.9	102521.7	81.5	11862.6	999.3	144726.9	6.9	950.8	1006.2	145677.7	260062.0	1.1
TO0312000	PA SANTO ANTONIO BOM SOSSEGO	0.0	0.0	37.3	1035.6			1399.6	43892.8	1399.6	43892.8	44928.4	
TO0313000	PA SÃO JOSÉ BELA VISTA	0.0	0.0	7.1	129.4			1752.6	53788.1	1752.6	53788.1	53917.5	
TO0314000	PA BARRA DO RIO ARRAIAS	0.0	0.0	249.8	19296.0			326.3	25207.2	326.3	25207.2	44503.3	
TO0315000	PA SANTA CLARA II	0.0	0.0	13.4	440.4			441.8	17622.5	441.8	17622.5	18063.0	
TO0316000	PA POÇO AZUL	0.0	0.0	1183.1	39032.1			421.5	13906.9	421.5	13906.9	52939.0	
TO0317000	PA FORTALEZA	334.4	54500.1	0.0	0.0					0.0	0.0	54500.1	
TO0319000	PA ALEGRIA II	0.0	0.0	71.3	1527.8			1833.1	35022.4	1833.1	35022.4	36550.3	
TO0320000	PA PIEDADE	0.0	0.0	420.1	13393.8			848.5	39485.4	848.5	39485.4	52879.2	
TO0321000	PA TAUARI	0.0	0.0	883.0	29255.6			2528.7	83571.9	2528.7	83571.9	112827.5	
TO0322000	PA IPÊ AMARELO	13.0	2114.8	255.7	41679.6	0.1	16.0	1092.1	177972.7	1092.2	177988.7	221783.1	
TO0323000	PA COCAL II	0.0	0.0	722.2	6803.5			1343.0	12651.5	1343.0	12651.5	19455.0	

TO0324000	PA SOLIDÁRIO	1264.6	205948.5	8.2	1331.1	22.1	3597.3			22.1	3597.3	210876.9	
TO0325000	PA SANTA RITA	0.0	0.0	431.2	14223.8			514.2	16439.0	514.2	16439.0	30662.7	
TO0326000	PA PADRE JOSIMO I E II	0.0	0.0	1884.2	62160.6			6262.0	205770.6	6262.0	205770.6	267931.1	
TO0327000	PA MARÍLIA	2612.3	259712.2	0.0	0.0	76.2	6538.8	156.9	3866.4	233.1	10405.2	270117.4	
TO0328000	PA ORLÂNDIA	726.8	81116.8	0.0	0.0	32.0	2614.8			32.0	2614.8	83731.6	
TO0329000	PA UNIVERSO	0.0	0.0	181.3	5981.5			1022.5	33730.8	1022.5	33730.8	39712.3	
TO0330000	PA 20 MIL	984.6	160444.0	0.0	0.0					0.0	0.0	160444.0	
TO0332000	PA BARRA MANSA	375.3	24726.8	334.8	3262.3	815.5	60758.5	4863.0	69231.9	5678.5	129990.4	157979.5	
TO0333000	PA SÃO JOSÉ I	0.0	0.0	1929.0	68654.4			6447.0	306985.0	6447.0	306985.0	375639.4	6.6
TO0334000	PA PIRARUCU I	0.0	0.0	747.3	24652.7			1160.7	38290.9	1160.7	38290.9	62943.6	
TO0335000	PA 1║ DE MAIO	636.4	69058.5	660.1	28900.7	1995.4	288683.8	876.2	43770.0	2871.6	332453.8	430412.9	135.3
TO0336000	PA PADRE EXPEDITO	508.7	21959.2	175.1	5867.4	286.1	9570.6	290.6	9587.1	576.7	19157.6	46984.2	
TO0337000	PA CHE GUEVARA	15.6	512.0	605.8	16607.9			775.5	24638.7	775.5	24638.7	41758.7	
TO0338000	PA BACURI	548.4	80742.3	41.9	6269.9	773.6	115884.5			773.6	115884.5	202896.7	
TO0339000	PA MORRO DAS NEVES	0.0	0.0	205.0	7338.4			645.1	27179.0	645.1	27179.0	34517.4	
TO0352000	PA RENASCER	0.0	0.0	1539.4	50786.3			646.0	21310.4	646.0	21310.4	72096.6	
TO0353000	PA CHAVE DE OURO	366.8	59776.6	28.2	4597.5	44.1	7194.3			44.1	7194.3	71568.3	
TO0354000	PA SANTA JULIA	0.0	0.0	392.4	15738.5			1397.3	65888.1	1397.3	65888.1	81626.6	
TO0355000	PA GROTA DE PEDRA	18.5	2536.4	517.1	15995.9	114.5	15648.2	1655.9	59752.2	1770.4	75400.4	93932.7	
TO0358000	PA MATÃO	0.0	0.0	166.0	5476.5			1120.6	36969.1	1120.6	36969.1	42445.6	
TO0359000	PA CAMPO DA MISSA	355.2	27443.0	101.7	7854.1	138.0	10658.1	597.2	46125.4	735.1	56783.5	92080.6	1.8
TO0370000	PA ARAGUAIA I	5922.4	797767.7	9894.0	523050.4	1659.8	235891.9	6707.0	267503.3	8366.8	503395.2	1824213.3	
TO0371000	PA SANTA RITA II	0.0	0.0	626.4	5900.6			2603.0	24519.9	2603.0	24519.9	30420.5	
TO0372000	PA LAGO DA UMBAÚBA	5.4	944.0	0.0	0.0			2966.3	526422.4	2966.3	526422.4	527366.5	84.7
TO0373000	PA PRINCIPADO DO CARMO	0.0	0.0	653.8	31271.3			5300.4	406411.1	5300.4	406411.1	437682.4	
TO0374000	PA TABOCA	0.0	0.0	318.4	11737.3			382.3	25159.6	382.3	25159.6	36896.9	
TO0375000	PA NOSSA SENHORA DE FÁTIMA	0.0	0.0	134.9	4450.7			868.5	28650.7	868.5	28650.7	33101.4	

TO0376000	PA SANTA TEREZA I	0.0	0.0	88.9	3640.0			2175.0	121478.1			2175.0	121478.1	125118.1	
TO0377000	PA SANTO ONOFRE	0.0	0.0	22.1	730.0			2466.0	81353.1			2466.0	81353.1	82083.1	
TO0378000	PA MALHADA DA PEDRA	0.0	0.0	1262.0	81184.1			1814.2	137440.7			1814.2	137440.7	218624.8	
TO0379000	PA PONTAL I	0.0	0.0	51.3	2325.6			1957.0	82484.1			1957.0	82484.1	84809.8	
TO0380000	PA PONTAL II	0.0	0.0	306.8	10191.0	0.0	0.4	2782.8	119045.8			2782.8	119046.2	129237.2	
TO0381000	PA BONANZA	111.2	8336.4	305.0	2872.7	507.9	64240.1	1925.4	18618.5			2433.3	82858.6	94067.7	
TO0382000	PA FIRMEZA I	0.0	0.0	454.4	7869.4			5462.2	171798.1			5462.2	171798.1	179667.5	
TO0383000	PA BOM SUCESSO	0.0	0.0	1187.2	39503.7			38.3	1318.7			38.3	1318.7	40822.3	26.1
TO0384000	PA LAGO VERDE	0.0	0.0	2091.6	76525.8			2295.4	104240.7			2295.4	104240.7	180766.4	
TO0385000	PA PEDRA DE AMOLAR	1245.2	154303.8	74.7	3881.9	139.7	14918.2	1760.6	65417.7			1900.3	80335.9	238521.5	
TO0386000	PA SANTA HELENA II	1151.7	214569.7	0.0	0.0	301.5	56352.6					301.5	56352.6	270922.3	
TO0387000	PA MACAÚBA	199.6	25209.7	3190.9	89863.1	2165.1	245005.5	1012.4	73427.7			3177.5	318433.2	433506.0	41.4
TO0389000	PA ONALÍCIO BARROS	0.0	0.0	3986.4	70791.5	403.9	40435.1	3055.2	62382.4			3459.2	102817.5	173609.0	41.3
TO0390000	PA BOM JESUS	0.0	0.0	868.4	28646.9			1625.1	53612.6			1625.1	53612.6	82259.5	
TO0391000	PA ESTRELA DO ARAGUAIA	499.8	18766.0	637.2	21507.2	1783.5	238613.9	2115.8	112355.6			3899.3	350969.4	391242.7	79.9
TO0392000	PA NOVA CANAÃ	88.6	11859.8	836.9	35315.0	1393.2	173556.4	5519.5	127218.1			6912.7	300774.4	347949.3	6.2
TO0394000	PA CANTO DE BARRO	568.0	50088.8	41.9	3343.2	138.9	8987.3	810.8	41974.7			949.6	50962.0	104394.1	
TO0395000	PA BREJO FEIO I	550.0	83330.3	30.6	4804.7	101.4	15225.9	3198.3	520009.5			3299.7	535235.3	623370.3	
TO0397000	PA FORÇA DA ESPERANÇA	0.0	0.0	250.5	14269.8			1696.9	128437.0			1696.9	128437.0	142706.8	
TO0402000	PA BARREIRINHO	12.6	1731.0	565.7	20855.6	137.9	18949.5	1103.8	52317.6			1241.6	71267.1	93853.8	9.2
TO0403000	PA VARGEM DOURADA	0.0	0.0	804.9	15105.7			1345.4	33608.9			1345.4	33608.9	48714.6	
TO0404000	PA SALOMIRA	0.0	0.0	1485.4	14160.2			4603.3	45281.2			4603.3	45281.2	59441.4	
TO0405000	PA MULHER CIDADÃ	0.0	0.0	1007.7	36912.1			733.4	32526.9			733.4	32526.9	69439.0	
TO0406000	PA BACURI II	703.5	79754.6	7.0	1050.6	249.3	37344.6			14.5	2172.5	263.8	39517.1	120322.3	
TO0407000	PA NOVA CANAÃ II	0.0	0.0	195.5	6450.1			646.2	21316.5			646.2	21316.5	27766.6	
TO0408000	PA VALE DO BANANAL	0.0	0.0	384.4	3621.4			1323.3	12465.9			1323.3	12465.9	16087.3	
TO0409000	PA SÃO SEBASTIÃO I	0.0	0.0	67.8	639.0			1051.2	9901.9			1051.2	9901.9	10541.0	

TO0410000	PA TERRA VERMELHA	0.0	0.0	275.9	12266.8			1898.2	144672.7	1898.2	144672.7	156939.5	
TO0411000	PA SÃO BENTO I	0.0	0.0	398.1	21142.8			1895.6	136152.0	1895.6	136152.0	157294.7	
TO0412000	PA PINDORAMA I	0.0	0.0	95.6	7325.9			2159.8	165273.0	2159.8	165273.0	172598.9	
TO0413000	PA PALMARES	1913.8	316472.6	0.0	0.0					0.0	0.0	316472.6	
TO0414000	PA VALE DO BARREIRAS	222.7	2097.9	22.7	213.8	4.0	239.0	1032.8	9729.3	1036.9	9968.3	12280.1	0.0
TO0415000	PA MÁRTIRES DA TERRA	1925.3	313758.9	81.2	13233.1	884.2	144105.4			884.2	144105.4	471097.5	
TO0416000	PA MATıES	0.0	0.0	54.9	2615.0			648.4	45297.3	648.4	45297.3	47912.3	
TO0417000	PA VEREDÃO	0.0	0.0	372.0	12302.7			1286.2	47096.1	1286.2	47096.1	59398.9	
TO0418000	PA MATA AZUL I	4213.7	675354.3	0.0	0.0	47.1	7681.4			47.1	7681.4	683035.7	
TO0419000	PA NOSSA SENHORA DA CONCEIÇÃO	0.0	0.0	287.4	9479.8			898.2	29630.1	898.2	29630.1	39109.9	
TO0420000	PA CARAJÁS	0.0	0.0	217.6	7180.0			968.9	31964.8	968.9	31964.8	39144.8	
TO0421000	PA PRATINHA	0.0	0.0	0.0	0.0			227.5	7505.5	227.5	7505.5	7505.5	
TO0423000	PA PONTAL DAS ESTRELAS	0.0	0.0	28.7	972.1	0.0	1.8	1750.5	63982.4	1750.5	63984.2	64956.3	
TO0424000	PA PONTAL DAS ESTRELAS I	0.0	0.0	725.3	23927.4			650.2	21448.9	650.2	21448.9	45376.3	
TO0425000	PA CORUJA	30.8	5012.4	0.0	0.0			602.8	59265.0	602.8	59265.0	64277.4	
TO0426000	PA NOVA VIDA I	0.0	0.0	159.2	4900.8			344.5	9850.1	344.5	9850.1	14751.0	
TO0427000	PA MANGUEIRA	0.0	0.0	145.4	4796.8			3199.7	105557.4	3199.7	105557.4	110354.2	
TO0429000	PA MAJU	836.1	136266.3	66.4	10820.8	61.2	9979.0	143.1	23324.8	204.4	33303.7	180390.9	
TO0430000	PA NOVA UNIÃO	2540.7	472653.9	0.0	0.0	377.5	70479.4			377.5	70479.4	543133.4	
TO0431000	PA DA MATA	0.0	0.0	1212.2	39991.0	264.3	20025.4	3059.1	78448.3	3323.4	98473.6	138464.6	56.5
TO0432000	PA SITIO	0.0	0.0	17.9	581.5			2614.1	93547.7	2614.1	93547.7	94129.3	
TO0433000	PA BREJO DO CAMPO	0.0	0.0	8.0	168.9			1128.9	32396.9	1128.9	32396.9	32565.8	
TO0434000	PA PONTAL DAS ESTRELAS V	0.0	0.0	42.9	1415.7			663.0	31158.1	663.0	31158.1	32573.8	
TO0435000	PA CANOA	5643.8	919765.0	0.0	0.0	953.2	155343.6			953.2	155343.6	1075108.6	
TO0436000	PA SÃO FRANCISCO DE ASSIS	0.0	0.0	340.9	11247.5			2197.3	72490.4	2197.3	72490.4	83737.9	
TO0437000	PA VIRGÍNIA	0.0	0.0	759.4	25053.1			952.1	31410.4	952.1	31410.4	56463.5	
TO0438000	PA VITÓRIA III	0.0	0.0	0.0	0.0			1307.1	29683.4	1307.1	29683.4	29683.4	

TO0439000	PA BARONESA I	0.0	0.0	184.9	6091.6			3144.9	103638.4			3144.9	103638.4	109730.0	
TO0440000	PA PEQUIZEIRO	0.0	0.0	24.9	234.5			977.9	9211.4			977.9	9211.4	9445.8	
TO0441000	PA MUTAMBA	1021.3	170620.0	0.0	0.0							0.0	0.0	170620.0	12.5
TO0442000	PA 25 DE MARÇO	0.0	0.0	705.7	23280.4			838.8	27672.6			838.8	27672.6	50953.0	
TO0443000	PA TERRA PROMETIDA	0.0	0.0	560.1	19877.2			930.9	51513.2			930.9	51513.2	71390.4	0.4
TO0444000	PA SANTA MARIA I	235.2	35316.4	5.6	836.5	105.7	15834.8					105.7	15834.8	51987.7	
TO0445000	PA FORTALEZA I	0.0	0.0	1156.2	38142.9			2338.5	77148.4			2338.5	77148.4	115291.3	214.6
TO0446000	PA SANTA TEREZA II	0.0	0.0	1217.2	41162.8			2147.5	92363.3			2147.5	92363.3	133526.1	
TO0447000	PA SANTA LUZIA I	685.5	102692.4	10.8	1615.9	134.9	20216.8	260.2	38974.3			395.1	59191.1	163499.3	
TO0448000	PA LUAR DO SERTAO	1342.7	222040.2	9.6	1592.7	580.9	97216.5	7.3	1211.8			588.1	98428.3	322061.2	
TO0449000	PA PROVÍNCIA	2894.9	413136.9	0.0	0.0	709.3	115294.4					709.3	115294.4	528431.3	
TO0451000	PA MANOEL ALVES	2870.6	262162.2	249.0	19231.4	7.9	1169.5					7.9	1169.5	282563.0	
TO0452000	PA VALE DO PARAISO	480.6	74806.8	111.8	15011.3	221.2	35047.7	304.5	28600.1			525.7	63647.8	153465.9	
TO0453000	PA BARRA DO LONTRA	868.8	129680.7	0.0	0.0	505.7	58560.6					505.7	58560.6	188241.3	
Total in Tocantins		344534.1	52030079.9	234610.5	9969701.4	64946.7	9092853.1	496045.8	26716601.8	156.2	18963.6	561148.7	35828418.5	97828199.7	2565.5

Table S6 Estimates of area of forest and of clearing (km^2 and %) through the year of official creation of each settlement and in the period after creation (from one year after creation to 2005 and from 2006 or one year after creation to 2013) in 950 settlements created between 2000 and 2008

Year of creation	Settlement project code	Settlement project	Total area of forest (km^2)	Total area cleared through the year of creation (km^2)		Remaining forest in the year of settlement creation		From one year after creation to 2005: Area total cleared		From one year after creation to 2005: Annual mean clearing	Remaining forest in 2005		From 2006 to 2013 or from one year after creation to 2013: Total area cleared		From 2006 to 2013 or from one year after creation to 2013: Annual mean clearing	Remaining forest in 2013	
				km^2	%	km^2	%	km^2	%	%	km^2	%	km^2	%	%	km^2	%
2000	AM0039000	PDS MORENA	484.40	0.68	0%	483.71	100%	1.65	0%	0%	482.06	100%	0.84	0%	0%	481.22	99%
2000	AM0040000	PA ENGENHO	22.62	0.24	1%	22.38	99%	1.17	5%	1%	21.22	94%	2.77	13%	2%	18.44	82%
2000	AM0041000	PA CAVIANA	52.32	3.93	8%	48.40	92%	2.16	4%	1%	46.23	88%	2.22	5%	1%	44.01	84%
2000	AP0028000	PA PEDRA BRANCA	294.44	13.65	5%	280.79	95%	16.56	6%	1%	264.23	90%	4.19	2%	0%	260.04	88%
2000	AP0029000	PA CORRE ÁGUA	58.18	13.10	23%	45.08	77%	3.43	8%	2%	41.64	72%	3.85	9%	1%	37.80	65%
2000	MA0502000	PA CHICO MENDES/ BANANEIRAS	56.24	26.04	46%	30.20	54%	12.73	42%	8%	17.47	31%	9.02	52%	6%	8.45	15%
2000	MA0508000	PA CASA BRANCA	20.47	1.13	6%	19.34	94%	7.40	38%	8%	11.94	58%	3.11	26%	3%	8.83	43%
2000	MA0510000	PA VENEZA	3.81	1.41	37%	2.39	63%	1.27	53%	11%	1.12	29%	0.07	6%	1%	1.05	28%
2000	MA0511000	PA SANTA ISABEL	26.42	7.02	27%	19.40	73%	9.16	47%	9%	10.24	39%	5.47	53%	7%	4.77	18%
2000	MA0521000	PA TINGIDOR	50.51	0.00	0%	50.51	100%	0.54	1%	0%	49.98	99%	6.04	12%	2%	43.94	87%
2000	MA0523000	PA LAGO DA CARNAÚBA	92.50	92.50	100%	0.00	0%	0.00			0.00	0%	0.00		0%	0.00	0%
2000	MA0526000	PA ROSELI NUNES/BANANEIRAS	63.09	17.79	28%	45.30	72%	14.59	32%	6%	30.72	49%	11.22	37%	5%	19.50	31%
2000	MA0538000	PE VERA CRUZ I	104.35	23.16	22%	81.20	78%	18.75	23%	5%	62.45	60%	7.54	12%	2%	54.91	53%
2000	MA0540000	PA CIPOAL	241.13	0.00	0%	241.13	100%	194.64	81%	16%	46.49	19%	7.13	15%	2%	39.36	16%
2000	MA0541000	PA JAGUARIBE	19.39	0.00	0%	19.39	100%	19.39	100%	20%	0.00	0%	0.00		0%	0.00	0%

2000	MA0542000	PE MANAUS	31.38	22.42	71%	8.97	29%	4.95	55%	11%	4.02	13%	1.39	35%	4%	2.63	8%
2000	MB0268000	PA CHICO MENDES I	21.39	4.91	23%	16.49	77%	0.87	5%	1%	15.62	73%	2.92	19%	2%	12.69	59%
2000	MB0276000	PA SANTA EUDOXIA	30.14	25.19	84%	4.95	16%	3.66	74%	15%	1.29	4%	0.56	43%	5%	0.73	2%
2000	MB0278000	PA MARINGÁ	41.62	20.94	50%	20.68	50%	13.35	65%	13%	7.33	18%	3.45	47%	6%	3.88	9%
2000	MB0284000	PA RANCHARIA	31.27	8.64	28%	22.63	72%	3.09	14%	3%	19.54	63%	5.66	29%	4%	13.88	44%
2000	MB0286000	PA VISTA ALEGRE	20.99	2.95	14%	18.04	86%	7.95	44%	9%	10.09	48%	4.10	41%	5%	5.99	29%
2000	MB0287000	PA ACAPU	10.21	3.71	36%	6.50	64%	5.84	90%	18%	0.66	6%	0.14	22%	3%	0.52	5%
2000	MB0288000	PA VALE DA SERRA	21.60	18.38	85%	3.22	15%	1.88	58%	12%	1.34	6%	0.25	19%	2%	1.09	5%
2000	MB0289000	PA UNIAO DA VITORIA	41.60	34.11	82%	7.50	18%	3.56	47%	9%	3.94	9%	0.41	11%	1%	3.53	8%
2000	MB0290000	PA JERONIMO NUNES LACERDA	37.57	33.74	90%	3.84	10%	2.39	62%	12%	1.45	4%	0.39	27%	3%	1.06	3%
2000	MB0291000	PA MARIA DE LOURDES RODRIGUES	41.85	40.68	97%	1.17	3%	0.78	67%	13%	0.39	1%	0.09	23%	3%	0.30	1%
2000	MB0293000	PA RIO PARA	54.24	2.05	4%	52.19	96%	15.81	30%	6%	36.37	67%	14.68	40%	5%	21.69	40%
2000	MB0294000	PA RIO CINZA	56.16	10.84	19%	45.32	81%	15.21	34%	7%	30.11	54%	17.08	57%	7%	13.02	23%
2000	MB0295000	PA RIO NEGRO	43.89	4.04	9%	39.85	91%	13.92	35%	7%	25.93	59%	12.00	46%	6%	13.93	32%
2000	MB0296000	PA ARAPARI	142.25	17.75	12%	124.50	88%	32.32	26%	5%	92.18	65%	44.02	48%	6%	48.16	34%
2000	MB0297000	PA EMÍDIO BATISTA DE MOURA	117.36	111.56	95%	5.80	5%	2.91	50%	10%	2.89	2%	0.19	7%	1%	2.69	2%
2000	MB0298000	PA 21 DE ABRIL	17.75	13.06	74%	4.69	26%	3.88	83%	17%	0.81	5%	0.11	14%	2%	0.70	4%
2000	MB0299000	PA VALENTIM SERRA	22.88	14.91	65%	7.97	35%	1.65	21%	4%	6.32	28%	3.31	52%	7%	3.01	13%
2000	MT0240000	PA SANTA CLARA	183.72	35.87	20%	147.85	80%	38.63	26%	5%	109.22	59%	22.35	20%	3%	86.88	47%
2000	MT0253000	PA ESCONDIDO	44.70	0.00	0%	44.70	100%	2.53	6%	1%	42.17	94%	13.32	32%	4%	28.84	65%
2000	MT0275000	PA SÃO SATURNINO	28.92	7.84	27%	21.08	73%	18.33	87%	17%	2.76	10%	1.26	46%	6%	1.50	5%
2000	MT0295000	PA UNIÃO FLOR DA	8.21	7.98	97%	0.23	3%	0.14	62%	12%	0.09	1%	0.00	0%	0%	0.09	1%

		SERRA															
2000	MT0329000	PA LAGOA RICA	20.98	11.90	57%	9.08	43%	3.92	43%	9%	5.16	25%	1.05	20%	3%	4.11	20%
2000	MT0346000	PA MONTE VERDE	31.05	12.90	42%	18.14	58%	11.02	61%	12%	7.12	23%	2.99	42%	5%	4.13	13%
2000	MT0349000	PA SÃO VICENTE	233.90	79.48	34%	154.42	66%	75.73	49%	10%	78.69	34%	32.27	41%	5%	46.42	20%
2000	MT0356000	PA CACHIMBO II	494.47	209.06	42%	285.41	58%	146.66	51%	10%	138.75	28%	44.67	32%	4%	94.07	19%
2000	MT0366000	PA VALE DA ESPERANÇA	19.76	11.69	59%	8.07	41%	2.51	31%	6%	5.56	28%	0.47	9%	1%	5.09	26%
2000	MT0443000	PE JOÃO PONCE DE ARRUDA	1.09	0.00	0%	1.09	100%	0.00	0%	0%	1.09	100%	0.00	0%	0%	1.09	100%
2000	MT0444000	PA FLORESTAN FERNANDES	43.57	40.29	92%	3.28	8%	0.25	8%	2%	3.03	7%	0.22	7%	1%	2.81	6%
2000	PA0250000	PA JOÃO BATISTA II	21.15	16.36	77%	4.79	23%	1.87	39%	8%	2.92	14%	0.02	1%	0%	2.90	14%
2000	RO0110000	PA MARIA MENDES	17.16	2.59	15%	14.57	85%	6.88	47%	9%	7.69	45%	4.03	52%	7%	3.65	21%
2000	RO0111000	PA NELSON ALVES	9.38	7.38	79%	1.99	21%	0.33	17%	3%	1.66	18%	0.56	34%	4%	1.10	12%
2000	RO0112000	PA JANDAIRA	10.18	3.48	34%	6.70	66%	2.41	36%	7%	4.29	42%	1.78	41%	5%	2.51	25%
2000	RO0113000	PA JOANA D ARC I	219.67	31.65	14%	188.02	86%	26.98	14%	3%	161.04	73%	29.47	18%	2%	131.57	60%
2000	RO0114000	PA JOANA D ARC II	147.15	9.06	6%	138.09	94%	13.85	10%	2%	124.24	84%	12.96	10%	1%	111.28	76%
2000	RO0115000	PA JOANA D ARC III	241.57	4.14	2%	237.43	98%	22.09	9%	2%	215.34	89%	23.78	11%	1%	191.56	79%
2000	RO0116000	PA FRANCISCO JOAO	135.35	50.68	37%	84.68	63%	37.16	44%	9%	47.52	35%	8.21	17%	2%	39.30	29%
2000	RO0117000	PA FLORIANO MAGNO	164.28	43.16	26%	121.11	74%	56.49	47%	9%	64.62	39%	13.46	21%	3%	51.16	31%
2000	RO0118000	PA ESMOSINA PINHO	115.19	42.90	37%	72.29	63%	27.53	38%	8%	44.76	39%	15.44	34%	4%	29.33	25%
2000	RO0119000	PA IVO INACIO	126.44	37.63	30%	88.81	70%	31.35	35%	7%	57.46	45%	4.83	8%	1%	52.63	42%
2000	RO0120000	PA NOVA VIDA	85.84	42.89	50%	42.96	50%	24.45	57%	11%	18.51	22%	3.77	20%	3%	14.75	17%
2000	RO0121000	PA SÃO JOSÉ DO BURITIS	91.29	55.96	61%	35.32	39%	21.60	61%	12%	13.73	15%	4.56	33%	4%	9.17	10%

2000	RO0122000	PA SÃO PAULO	48.03	12.59	26%	35.44	74%	21.21	60%	12%	14.23	30%	6.45	45%	6%	7.78	16%
2000	RO0123000	PA NILSON CAMPOS	125.19	31.38	25%	93.81	75%	21.79	23%	5%	72.02	58%	16.14	22%	3%	55.88	45%
2000	RO0125000	PA ORIENTE	96.63	43.40	45%	53.23	55%	26.50	50%	10%	26.73	28%	5.59	21%	3%	21.14	22%
2000	TO0201000	PA CONQUISTA	22.14	18.97	86%	3.17	14%	3.08	97%	19%	0.09	0%	0.00	0%	0%	0.09	0%
2000	TO0212000	PA SÃO SEBASTIÃO	22.60	16.90	75%	5.71	25%	4.16	73%	15%	1.55	7%	0.85	55%	7%	0.70	3%
2000	TO0267000	PA SOSSEGO	4.68	4.68	100%	0.00	0%	0.00			0.00	0%	0.00			0.00	0%
2001	AC0068000	PDS SÃO SALVADOR	508.97	21.51	4%	487.46	96%	7.26	1%	0%	480.20	94%	3.44	1%	0%	476.77	94%
2001	AC0070000	PA TUPÁ	61.90	25.31	41%	36.59	59%	2.60	7%	2%	33.99	55%	4.10	12%	2%	29.90	48%
2001	AC0071000	PAE EQUADOR	78.71	7.40	9%	71.32	91%	2.75	4%	1%	68.57	87%	1.08	2%	0%	67.49	86%
2001	AC0072000	PAE CRUZEIRO DO VALE	779.68	6.59	1%	773.10	99%	4.20	1%	0%	768.90	99%	7.88	1%	0%	761.01	98%
2001	AC0074000	PA NARCISO ASSUNÇÃO	49.44	1.58	3%	47.86	97%	1.10	2%	1%	46.75	95%	3.86	8%	1%	42.89	87%
2001	AC0075000	PA PEDRO FIRMINO	63.93	1.54	2%	62.40	98%	2.38	4%	1%	60.01	94%	4.22	7%	1%	55.80	87%
2001	AC0076000	PA ZAQUEU MACHADO	37.58	33.07	88%	4.52	12%	1.56	35%	9%	2.96	8%	0.19	7%	1%	2.76	7%
2001	AC0077000	PE POLO AGROF. DOM MOACIR	3.21	2.85	89%	0.36	11%	0.01	2%	0%	0.35	11%	0.00	0%	0%	0.35	11%
2001	AC0078000	PA PORFIRIO PONCIANO	17.59	0.05	0%	17.54	100%	0.04	0%	0%	17.50	99%	0.02	0%	0%	17.48	99%
2001	AC0079000	PE POLO AGROFLORESTAL BRASILÉIA	5.39	3.49	65%	1.90	35%	0.50	26%	7%	1.40	26%	0.19	14%	2%	1.20	22%
2001	AC0081000	PE POLO AGROF. WILSON PINHEIRO	3.01	1.62	54%	1.39	46%	0.88	64%	16%	0.51	17%	0.01	2%	0%	0.50	16%
2001	AC0083000	PE POLO AGROF. MÂNCIO LIMA	3.76	1.94	52%	1.82	48%	0.13	7%	2%	1.70	45%	0.19	11%	1%	1.51	40%

2001	AC0084000	PE POLO AGROF. CRUZEIRO DO SUL	3.20	1.63	51%	1.57	49%	0.06	4%	1%	1.51	47%	0.13	8%	1%	1.38	43%
2001	AC0089000	PE POLO LEITEIRO DE PORTO ACRE	2.05	1.86	90%	0.20	10%	0.11	57%	14%	0.09	4%	0.01	15%	2%	0.07	4%
2001	AC0090000	PE POLO AGROF. EPITACIOLÂNDIA	1.30	0.47	36%	0.83	64%	0.14	17%	4%	0.69	53%	0.00	0%	0%	0.69	53%
2001	AM0042000	PAE MATUPIRI	82.21	2.47	3%	79.74	97%	0.15	0%	0%	79.58	97%	0.57	1%	0%	79.02	96%
2001	MA0546000	PA QUADRA BOA VIDA	3.17	2.62	83%	0.55	17%	0.01	1%	0%	0.54	17%	0.35	64%	8%	0.20	6%
2001	MA0553000	PCA VASSORAL	1.14	1.14	100%	0.00	0%	0.00			0.00	0%	0.00		0%	0.00	0%
2001	MA0555000	PA XIXANDÁ	21.13	15.62	74%	5.51	26%	1.35	24%	6%	4.16	20%	2.54	61%	8%	1.62	8%
2001	MA0566000	PA NOVO HORIZONTE II	2.42	2.42	100%	0.00	0%	0.00			0.00	0%	0.00		0%	0.00	0%
2001	MA0580000	PA PADRE JOSIMO I	12.07	12.07	100%	0.00	0%	0.00			0.00	0%	0.00		0%	0.00	0%
2001	MA0581000	PA BOA ESPERANÇA III	45.50	21.19	47%	24.31	53%	7.07	29%	7%	17.24	38%	7.45	43%	5%	9.79	22%
2001	MA0583000	PA COCALINHO	7.19	7.19	100%	0.00	0%	0.00			0.00	0%	0.00		0%	0.00	0%
2001	MA0584000	PA QUIXABA	4.98	4.98	100%	0.00	0%	0.00			0.00	0%	0.00		0%	0.00	0%
2001	MA0592000	PA ALVORADA IV	34.44	34.24	99%	0.20	1%	0.17	85%	21%	0.03	0%	0.01	27%	3%	0.02	0%
2001	MB0292000	PA LARANJEIRAS	72.92	29.29	40%	43.63	60%	8.00	18%	5%	35.63	49%	17.89	50%	6%	17.74	24%
2001	MB0306000	PA BEIRA RIO	18.71	1.26	7%	17.45	93%	4.63	27%	7%	12.82	69%	7.18	56%	7%	5.64	30%
2001	MB0307000	PA BURGO	35.94	25.07	70%	10.87	30%	3.96	36%	9%	6.92	19%	2.40	35%	4%	4.52	13%
2001	MB0308000	PA CARIMÃ	20.80	7.95	38%	12.85	62%	2.61	20%	5%	10.24	49%	3.99	39%	5%	6.25	30%
2001	MB0309000	PA BELO MIRAR	34.92	26.83	77%	8.09	23%	4.88	60%	15%	3.20	9%	1.26	39%	5%	1.94	6%
2001	MB0310000	PA NOSSA SENHORA DAS GRAÇAS	35.65	29.35	82%	6.30	18%	3.20	51%	13%	3.10	9%	1.40	45%	6%	1.70	5%
2001	MB0311000	PA BOCA DO LAGO	33.72	18.00	53%	15.72	47%	7.93	50%	13%	7.80	23%	3.13	40%	5%	4.66	14%

2001	MB0313000	PA BORRACHEIRA	25.68	7.61	30%	18.06	70%	6.52	36%	9%	11.54	45%	7.30	63%	8%	4.24	17%
2001	MB0314000	PA ARICA	16.54	15.15	92%	1.39	8%	1.38	99%	25%	0.01	0%	0.00	0%	0%	0.01	0%
2001	MB0315000	PA MINEIRO PRETO	99.45	2.23	2%	97.23	98%	24.34	25%	6%	72.89	73%	40.12	55%	7%	32.77	33%
2001	MB0316000	PA ALTO PACAJÁ	134.60	9.13	7%	125.47	93%	20.71	17%	4%	104.75	78%	64.45	62%	8%	40.30	30%
2001	MB0317000	PA BOCA LARGA	69.66	3.65	5%	66.01	95%	14.64	22%	6%	51.37	74%	30.65	60%	7%	20.73	30%
2001	MB0318000	PA JACARÉ-AÇÚ	115.25	0.54	0%	114.72	100%	14.59	13%	3%	100.13	87%	46.51	46%	6%	53.62	47%
2001	MB0319000	PA JOSÉ CIRILO GOMES	101.80	8.32	8%	93.48	92%	18.86	20%	5%	74.62	73%	44.20	59%	7%	30.43	30%
2001	MB0320000	PA RIO PRETO	59.22	3.92	7%	55.31	93%	10.54	19%	5%	44.77	76%	23.64	53%	7%	21.13	36%
2001	MB0321000	PA CASCALHO	26.69	5.12	19%	21.57	81%	3.25	15%	4%	18.33	69%	7.04	38%	5%	11.28	42%
2001	MB0322000	PA NOVA VIDA	29.89	6.19	21%	23.69	79%	3.58	15%	4%	20.11	67%	9.35	46%	6%	10.77	36%
2001	MB0323000	PA CASTANHAL RATO II	14.73	11.05	75%	3.68	25%	1.41	38%	10%	2.27	15%	1.12	49%	6%	1.15	8%
2001	MB0324000	PA SANTA MARIA	21.58	1.53	7%	20.06	93%	2.18	11%	3%	17.87	83%	8.87	50%	6%	9.00	42%
2001	MB0325000	PA SANTA LIDUINA	19.10	2.78	15%	16.32	85%	2.17	13%	3%	14.16	74%	6.19	44%	5%	7.97	42%
2001	MB0326000	PA IOLANDA	34.38	9.30	27%	25.08	73%	2.40	10%	2%	22.68	66%	12.16	54%	7%	10.52	31%
2001	MB0327000	PA SÃO JOSE	45.02	40.03	89%	4.99	11%	2.05	41%	10%	2.94	7%	0.58	20%	2%	2.35	5%
2001	MB0328000	PA PAU FERRADO	29.50	24.12	82%	5.38	18%	3.61	67%	17%	1.77	6%	0.58	33%	4%	1.19	4%
2001	MB0329000	PA BEIJA FLOR II	11.66	0.80	7%	10.86	93%	0.78	7%	2%	10.07	86%	4.77	47%	6%	5.30	45%
2001	MB0330000	PA AMAPÁ I	33.63	9.55	28%	24.09	72%	2.97	12%	3%	21.12	63%	6.85	32%	4%	14.27	42%
2001	MB0331000	PA ALTO AMAZONAS	24.11	1.53	6%	22.58	94%	2.43	11%	3%	20.16	84%	9.25	46%	6%	10.90	45%
2001	MB0332000	PA BARRAGEIRA	29.74	3.95	13%	25.79	87%	3.23	13%	3%	22.56	76%	5.46	24%	3%	17.10	58%
2001	MB0333000	PA CACHOEIRINHA	12.53	1.02	8%	11.51	92%	2.65	23%	6%	8.86	71%	4.78	54%	7%	4.07	33%
2001	MB0334000	PA AMAPA II	25.18	8.17	32%	17.00	68%	1.77	10%	3%	15.23	61%	3.62	24%	3%	11.62	46%
2001	MB0335000	PA SÃO GERALDO DO REPARTIMENTO	20.97	0.73	3%	20.24	97%	1.94	10%	2%	18.30	87%	8.77	48%	6%	9.53	45%
2001	MB0336000	PA NOVO MUNDO DO	12.41	7.48	60%	4.93	40%	0.58	12%	3%	4.35	35%	1.79	41%	5%	2.55	21%

		ITUPIRANGA															
2001	MB0337000	PA BOA ESPERANÇA DO ARAGUAIA	27.13	23.30	86%	3.83	14%	2.20	57%	14%	1.63	6%	0.42	26%	3%	1.21	4%
2001	MB0338000	PA UNIÃO	29.27	11.23	38%	18.04	62%	2.76	15%	4%	15.28	52%	4.88	32%	4%	10.40	36%
2001	MB0339000	PA JERUZALÉM	26.12	15.71	60%	10.41	40%	0.80	8%	2%	9.60	37%	2.47	26%	3%	7.13	27%
2001	MB0340000	PA SANTA TEREZINHA	24.56	10.67	43%	13.89	57%	9.95	72%	18%	3.94	16%	2.12	54%	7%	1.82	7%
2001	MB0341000	PA SAMAUMA	15.51	9.80	63%	5.70	37%	1.74	30%	8%	3.97	26%	2.53	64%	8%	1.44	9%
2001	MB0342000	PA MARRECAS DO ARAGUAIA	34.65	25.15	73%	9.49	27%	3.54	37%	9%	5.95	17%	1.50	25%	3%	4.45	13%
2001	MB0343000	PA CUXIÚ	4.96	1.00	20%	3.96	80%	0.41	10%	3%	3.55	72%	1.34	38%	5%	2.21	45%
2001	MB0344000	PA JOSE PINHEIRO LIMA	39.89	8.36	21%	31.53	79%	4.96	16%	4%	26.58	67%	17.37	65%	8%	9.21	23%
2001	MB0345000	PA JURUNA	122.11	21.01	17%	101.10	83%	6.71	7%	2%	94.39	77%	32.04	34%	4%	62.35	51%
2001	MB0346000	PA PALMARES SUL	156.06	95.93	61%	60.13	39%	14.10	23%	6%	46.03	29%	16.77	36%	5%	29.26	19%
2001	MT0278000	PA ROSELI NUNES	95.21	40.85	43%	54.36	57%	23.84	44%	11%	30.52	32%	14.34	47%	6%	16.18	17%
2001	MT0331000	PA SANTO ANTONIO DO BELEZA	124.45	15.66	13%	108.80	87%	54.70	50%	13%	54.09	43%	29.64	55%	7%	24.45	20%
2001	MT0457000	PA POUSO ALEGRE	22.10	4.40	20%	17.70	80%	3.44	19%	5%	14.26	65%	5.29	37%	5%	8.97	41%
2001	MT0458000	PA SANTO EXPEDITO	11.35	11.34	100%	0.01	0%	0.00	0%	0%	0.01	0%	0.00	0%	0%	0.01	0%
2001	MT0486000	PE VIDA NOVA	139.28	0.05	0%	139.23	100%	11.68	8%	2%	127.55	92%	21.34	17%	2%	106.20	76%
2001	MT0491000	PA GUAPORÉ	87.80	25.03	29%	62.77	71%	22.90	36%	9%	39.87	45%	8.84	22%	3%	31.03	35%
2001	MT0494000	PA BARRA NORTE	36.59	8.14	22%	28.45	78%	15.80	56%	14%	12.65	35%	5.13	41%	5%	7.52	21%
2001	PA0251000	PA CANAÃ	39.21	35.32	90%	3.89	10%	1.65	42%	11%	2.24	6%	1.08	48%	6%	1.16	3%
2001	PA0253000	PA TRÊS IRMÃOS	34.73	7.18	21%	27.54	79%	11.26	41%	10%	16.29	47%	5.68	35%	4%	10.61	31%
2001	PA0254000	PA TIMBORANA	32.42	22.12	68%	10.30	32%	4.24	41%	10%	6.06	19%	4.73	78%	10%	1.33	4%
2001	PA0261000	PCA MÁRTIRES DE	4.08	2.67	65%	1.41	35%	0.15	11%	3%	1.26	31%	0.01	1%	0%	1.24	30%

		ABRIL															
2001	RO0126000	PA EDMILSON PASTOR	4.16	0.72	17%	3.44	83%	0.15	4%	1%	3.29	79%	0.00	0%	0%	3.29	79%
2001	RO0127000	PA PADRE EZEQUIEL	59.73	28.12	47%	31.61	53%	4.92	16%	4%	26.69	45%	1.02	4%	0%	25.67	43%
2001	RO0129000	PA PARAISO DAS ACÁCIAS	5.83	1.53	26%	4.30	74%	3.36	78%	20%	0.94	16%	0.17	18%	2%	0.77	13%
2001	RO0131000	PA JATOBÁ	67.53	15.20	23%	52.33	77%	22.39	43%	11%	29.94	44%	11.13	37%	5%	18.81	28%
2001	RO0132000	PCA NOVO ALVORECER	7.25	6.66	92%	0.59	8%	0.20	34%	9%	0.39	5%	0.09	24%	3%	0.29	4%
2001	RO0133000	PA BOM PRINCÍPIO II	24.16	22.05	91%	2.12	9%	1.22	58%	14%	0.89	4%	0.38	43%	5%	0.51	2%
2001	RO0134000	PA BELA VISTA	17.48	8.82	50%	8.66	50%	2.88	33%	8%	5.78	33%	0.21	4%	0%	5.56	32%
2001	RO0135000	PA SERRA GRANDE	74.22	10.87	15%	63.34	85%	23.44	37%	9%	39.91	54%	18.74	47%	6%	21.17	29%
2001	RO0136000	PA SÃO FRANCISCO DO GUAPORÉ	43.19	33.91	79%	9.28	21%	5.16	56%	14%	4.13	10%	2.87	70%	9%	1.25	3%
2001	RO0149000	PA ROSANA LECY	30.04	0.06	0%	29.98	100%	6.65	22%	6%	23.33	78%	7.08	30%	4%	16.24	54%
2001	RR0037000	PA NOVA AMAZONIA	0.52	0.00	0%	0.52	100%	0.00	0%	0%	0.52	100%	0.00	0%	0%	0.52	100%
2001	SM0065000	PA ÁREA CUMINÁ E TROMBETAS	232.93	50.80	22%	182.13	78%	8.54	5%	1%	173.59	75%	15.74	9%	1%	157.85	68%
2001	SM0066000	PDS IGARAPÉ DO ANTA E CURUÁ UMA	105.85	0.17	0%	105.69	100%	0.66	1%	0%	105.03	99%	1.59	2%	0%	103.44	98%
2001	TO0268000	PA ITACOLOMI	10.20	10.20	100%	0.00	0%	0.00			0.00	0%	0.00		0%	0.00	0%
2001	TO0276000	PA CACHOEIRINHA	6.58	6.54	99%	0.04	1%	0.00	0%	0%	0.04	1%	0.00	0%	0%	0.04	1%
2001	TO0278000	PA PONTÃO	2.99	2.99	100%	0.00	0%	0.00			0.00	0%	0.00		0%	0.00	0%
2001	TO0279000	PA BASTÃO VELHO	0.24	0.24	100%	0.00	0%	0.00			0.00	0%	0.00		0%	0.00	0%
2001	TO0284000	PA SÃO GABRIEL	44.81	12.19	27%	32.62	73%	15.35	47%	12%	17.27	39%	6.95	40%	5%	10.32	23%
2002	AC0082000	PE POLO AGROF.	2.59	1.77	69%	0.81	31%	0.04	5%	2%	0.78	30%	0.23	30%	4%	0.54	21%

		RODRIGUES ALVES															
2002	AC0085000	PE POLO AGROF. XAPURI I	3.65	1.33	36%	2.32	64%	0.48	21%	7%	1.84	50%	0.32	17%	2%	1.52	42%
2002	AC0086000	PE POLO AGROF. XAPURI II	2.31	2.02	87%	0.29	13%	0.00	0%	0%	0.29	13%	0.00	0%	0%	0.29	13%
2002	AC0091000	PA BERLIM RECREIO	244.51	81.09	33%	163.42	67%	14.12	9%	3%	149.29	61%	25.11	17%	2%	124.18	51%
2002	AC0092000	PA JOAQUIM DE MATOS	52.24	7.88	15%	44.36	85%	11.49	26%	9%	32.87	63%	7.45	23%	3%	25.41	49%
2002	AM0045000	PA PIABA	33.96	2.52	7%	31.43	93%	2.50	8%	3%	28.93	85%	2.10	7%	1%	26.84	79%
2002	AM0048000	PA BÓIA	63.06	3.93	6%	59.13	94%	1.54	3%	1%	57.59	91%	0.95	2%	0%	56.64	90%
2002	AP0035000	PA IGARAPÉ GRANDE	11.74	0.00	0%	11.74	100%	0.00	0%	0%	11.74	100%	1.85	16%	2%	9.90	84%
2002	MA0593000	PE MARACUJÁ	3.45	0.36	10%	3.09	90%	0.03	1%	0%	3.06	89%	1.69	55%	7%	1.37	40%
2002	MA0600000	PE LARANJAL	9.62	9.62	100%	0.00	0%	0.00			0.00	0%	0.00		0%	0.00	0%
2002	MA0602000	PE PROMISSÃO	1.80	1.80	100%	0.00	0%	0.00			0.00	0%	0.00		0%	0.00	0%
2002	MA0603000	PE RIO GRANDE	3.51	0.48	14%	3.03	86%	1.18	39%	13%	1.84	53%	1.07	58%	7%	0.78	22%
2002	MA0604000	PA CANAÃ	8.37	8.37	100%	0.00	0%	0.00			0.00	0%	0.00		0%	0.00	0%
2002	MA0605000	PA BOA VISTA II	5.95	5.95	100%	0.00	0%	0.00			0.00	0%	0.00		0%	0.00	0%
2002	MA0606000	PA EL BETEL	3.11	3.11	100%	0.00	0%	0.00			0.00	0%	0.00		0%	0.00	0%
2002	MA0607000	PA CRUZEIRO DO SUL	11.13	6.50	58%	4.63	42%	0.49	11%	4%	4.14	37%	3.01	73%	9%	1.13	10%
2002	MA0609000	PA SUCESSO	4.21	4.21	100%	0.00	0%	0.00			0.00	0%	0.00		0%	0.00	0%
2002	MA0610000	PA IPIRANGA	2.02	2.02	100%	0.00	0%	0.00			0.00	0%	0.00		0%	0.00	0%
2002	MA0611000	PA MONTE LÍRIO	6.92	6.92	100%	0.00	0%	0.00			0.00	0%	0.00		0%	0.00	0%
2002	MA0612000	PA TAMANDARÉ	4.63	4.63	100%	0.00	0%	0.00			0.00	0%	0.00		0%	0.00	0%
2002	MA0614000	PA FÉ EM DEUS	5.84	5.84	100%	0.00	0%	0.00			0.00	0%	0.00		0%	0.00	0%
2002	MA0615000	PA BOA ESPERANÇA	8.97	8.97	100%	0.00	0%	0.00			0.00	0%	0.00		0%	0.00	0%

		I															
2002	MA0618000	PA VALE DO IPÊ	3.78	3.23	85%	0.55	15%	0.24	44%	15%	0.31	8%	0.08	26%	3%	0.23	6%
2002	MA0619000	PA ALTO BOA VISTA	21.06	9.33	44%	11.73	56%	2.39	20%	7%	9.34	44%	4.81	51%	6%	4.54	22%
2002	MA0635000	PA 6 DE MARÇO	3.14	3.14	100%	0.00	0%	0.00			0.00	0%	0.00		0%	0.00	0%
2002	MA0640000	PA REFUGIO	27.61	21.99	80%	5.62	20%	0.08	1%	0%	5.54	20%	1.17	21%	3%	4.37	16%
2002	MA0646000	PA ÁGUA FRIA DO ITINGA	74.41	36.09	48%	38.32	52%	14.28	37%	12%	24.05	32%	12.54	52%	7%	11.50	15%
2002	MA0647000	PA AGRÍCOLA 2002	25.04	24.66	98%	0.38	2%	0.04	11%	4%	0.34	1%	0.00	0%	0%	0.34	1%
2002	MA0648000	PA PIMENTA	2.82	2.82	100%	0.00	0%	0.00			0.00	0%	0.00		0%	0.00	0%
2002	MA0649000	PA QUADRA DIAMANTE	1.02	1.02	100%	0.00	0%	0.00			0.00	0%	0.00		0%	0.00	0%
2002	MA0650000	PA BELÉM	5.91	5.91	100%	0.00	0%	0.00			0.00	0%	0.00		0%	0.00	0%
2002	MA0651000	PA CUTIA/BAIÚNA	54.88	54.88	100%	0.00	0%	0.00			0.00	0%	0.00		0%	0.00	0%
2002	MA0655000	PA BURITIATÁ	8.73	8.25	94%	0.48	6%	0.05	11%	4%	0.43	5%	0.28	65%	8%	0.15	2%
2002	MA0668000	PA EL SHADAY	9.88	8.68	88%	1.20	12%	0.10	8%	3%	1.11	11%	0.00	0%	0%	1.11	11%
2002	MB0347000	PA ALCOBAÇA	1029.17	679.06	66%	350.12	34%	89.87	26%	9%	260.25	25%	75.48	29%	4%	184.77	18%
2002	MB0351000	PA IGARAPE DO RATO	21.89	16.71	76%	5.18	24%	1.83	35%	12%	3.36	15%	1.14	34%	4%	2.22	10%
2002	MB0352000	PA ARAÇATUBA CARAJÁS	14.16	11.97	85%	2.19	15%	0.89	41%	14%	1.30	9%	0.69	53%	7%	0.61	4%
2002	MB0353000	PA 4 DE JUNHO	11.94	5.49	46%	6.45	54%	3.11	48%	16%	3.34	28%	1.76	53%	7%	1.58	13%
2002	MB0354000	PA JARDIM	22.30	12.48	56%	9.82	44%	2.22	23%	8%	7.60	34%	3.08	41%	5%	4.51	20%
2002	MB0355000	PA FÊNIX	14.54	12.51	86%	2.03	14%	1.71	84%	28%	0.32	2%	0.09	30%	4%	0.22	2%
2002	MB0356000	PA JOSÉ DUTRA DA COSTA	29.27	15.69	54%	13.57	46%	4.95	36%	12%	8.62	29%	6.31	73%	9%	2.32	8%
2002	MB0358000	PA NOSSA SENHORA	34.05	21.48	63%	12.57	37%	5.07	40%	13%	7.50	22%	5.10	68%	8%	2.40	7%

		APARECIDA															
2002	MB0359000	PA NOVA VITORIA	59.66	37.27	62%	22.39	38%	5.62	25%	8%	16.78	28%	12.05	72%	9%	4.73	8%
2002	MB0360000	PA UNIDOS PARA VENCER	21.18	7.49	35%	13.69	65%	5.14	38%	13%	8.55	40%	5.45	64%	8%	3.10	15%
2002	MB0361000	PA PIQUIÁ	44.87	39.90	89%	4.97	11%	1.66	33%	11%	3.30	7%	0.60	18%	2%	2.70	6%
2002	MB0363000	PA PIRASSUNUNGA	4.85	1.53	32%	3.32	68%	0.46	14%	5%	2.86	59%	0.98	34%	4%	1.88	39%
2002	MT0604000	PE RP	62.48	40.15	64%	22.32	36%	9.37	42%	14%	12.95	21%	3.48	27%	3%	9.48	15%
2002	MT0608000	PA GAVIÃO	11.00	8.26	75%	2.74	25%	0.85	31%	10%	1.89	17%	1.58	84%	10%	0.31	3%
2002	MT0609000	PA CECILIA ANTUNES	11.58	10.47	90%	1.10	10%	0.08	7%	2%	1.02	9%	0.00	0%	0%	1.02	9%
2002	MT0615000	PA TEODOMIRO FERREIRA DOS SANTOS	114.11	91.00	80%	23.11	20%	1.77	8%	3%	21.34	19%	1.92	9%	1%	19.43	17%
2002	MT0627000	PA BOM SUCESSO	0.12	0.00	0%	0.12	100%	0.00	0%	0%	0.12	100%	0.01	10%	1%	0.11	90%
2002	MT0628000	PA RENASCER	165.37	111.37	67%	54.00	33%	9.39	17%	6%	44.61	27%	4.20	9%	1%	40.41	24%
2002	PA0256000	PA NOVA CONQUISTA	13.98	12.78	91%	1.20	9%	0.64	54%	18%	0.56	4%	0.00	0%	0%	0.56	4%
2002	PA0257000	PA OLHO D ÁGUA II	80.43	15.27	19%	65.16	81%	0.42	1%	0%	64.74	80%	7.24	11%	1%	57.50	71%
2002	RO0139000	PA MARTIM PESCADOR	154.44	114.26	74%	40.18	26%	19.30	48%	16%	20.88	14%	8.22	39%	5%	12.66	8%
2002	RO0140000	PA GOGÓ DA ONÇA	239.76	99.30	41%	140.46	59%	50.48	36%	12%	89.98	38%	44.81	50%	6%	45.17	19%
2002	RO0141000	PA CAUTARINHO	163.58	73.56	45%	90.02	55%	27.45	30%	10%	62.57	38%	22.96	37%	5%	39.61	24%
2002	RO0142000	PA SAGRADA FAMÍLIA	50.67	22.74	45%	27.93	55%	10.30	37%	12%	17.63	35%	7.75	44%	5%	9.88	19%
2002	RO0143000	PDS NAZARÉ E BOA VITÓRIA	35.14	7.30	21%	27.84	79%	0.04	0%	0%	27.80	79%	0.54	2%	0%	27.25	78%

2002	RO0146000	PA IGARAPÉ DAS ARARAS	154.44	15.06	10%	139.38	90%	38.66	28%	9%	100.72	65%	20.84	21%	3%	79.88	52%
2002	RO0147000	PA MARECHAL RONDON	114.70	18.08	16%	96.63	84%	29.81	31%	10%	66.81	58%	10.12	15%	2%	56.69	49%
2002	RO0148000	PA IGARAPÉ AZUL	106.12	10.54	10%	95.58	90%	25.05	26%	9%	70.53	66%	23.06	33%	4%	47.47	45%
2002	RO0150000	PA PAU BRASIL	148.82	33.57	23%	115.25	77%	38.23	33%	11%	77.02	52%	14.93	19%	2%	62.09	42%
2002	RO0151000	PA IGARAPE TAQUARA	196.21	43.94	22%	152.26	78%	37.18	24%	8%	115.08	59%	27.46	24%	3%	87.62	45%
2002	RO0152000	PA PAU DARCO	177.40	22.20	13%	155.20	87%	50.05	32%	11%	105.15	59%	25.47	24%	3%	79.69	45%
2002	RO0153000	PA RIBEIRÃO	129.01	17.20	13%	111.81	87%	31.02	28%	9%	80.79	63%	17.69	22%	3%	63.09	49%
2002	RR0038000	PA JACAMIM	25.43	1.18	5%	24.24	95%	0.00	0%	0%	24.24	95%	1.71	7%	1%	22.54	89%
2002	TO0297000	PA TUCUMIRIM	34.33	23.99	70%	10.34	30%	3.66	35%	12%	6.69	19%	1.90	28%	4%	4.79	14%
2002	TO0298000	PA ARRAIAS	13.87	11.55	83%	2.32	17%	0.35	15%	5%	1.97	14%	0.43	22%	3%	1.54	11%
2003	AC0093000	PCA CASULO GERALDO MESQUITA	2.23	2.16	97%	0.07	3%	0.00	0%	0%	0.07	3%	0.00	1%	0%	0.07	3%
2003	MA0608000	PA 1° DE JUNHO	2.87	2.86	100%	0.00	0%	0.00	0%	0%	0.00	0%	0.00	0%	0%	0.00	0%
2003	MA0666000	PA DEUS É FIEL	13.36	10.95	82%	2.41	18%	0.10	4%	2%	2.31	17%	0.14	6%	1%	2.17	16%
2003	MA0685000	PA QUADRA SÃO JOÃO BATISTA	1.89	1.89	100%	0.00	0%	0.00			0.00	0%	0.00		0%	0.00	0%
2003	MA0686000	PA QUADRA SÃO RAIMUNDO	2.04	2.04	100%	0.00	0%	0.00			0.00	0%	0.00		0%	0.00	0%
2003	MA0700000	PA PASSO LIVRE	56.53	9.37	17%	47.16	83%	6.92	15%	7%	40.24	71%	7.25	18%	2%	32.99	58%
2003	MA0688000	PE LARANJEIRAS / MURTURA	1.20	0.97	81%	0.23	19%	0.00	0%	0%	0.23	19%	0.04	16%	2%	0.19	16%
2003	MA0725000	PA FELIZ SÃO FRANCISCO	2.58	2.58	100%	0.00	0%	0.00			0.00	0%	0.00		0%	0.00	0%
2003	MA0727000	PA SÃO VICENTE	2.20	2.20	100%	0.00	0%	0.00			0.00	0%	0.00		0%	0.00	0%

2003	MA0729000	PA BEBE ÁGUA	2.39	2.39	100%	0.00	0%	0.00			0.00	0%	0.00		0%	0.00	0%
2003	MA0730000	PA TRÊS PALMEIRAS	2.06	2.01	98%	0.04	2%	0.00	0%	0%	0.04	2%	0.00	0%	0%	0.04	2%
2003	MB0364000	PA POUSO ALEGRE	11.34	4.24	37%	7.10	63%	0.25	4%	2%	6.85	60%	3.86	56%	7%	2.99	26%
2003	MB0365000	PA SANTA RITA	9.51	7.64	80%	1.87	20%	0.26	14%	7%	1.61	17%	0.98	61%	8%	0.64	7%
2003	MB0366000	PA POUSO BONITO	14.17	5.69	40%	8.49	60%	0.99	12%	6%	7.50	53%	2.06	28%	3%	5.44	38%
2003	MB0367000	PA BOA SORTE II	9.03	4.68	52%	4.35	48%	0.59	14%	7%	3.76	42%	0.45	12%	1%	3.31	37%
2003	MB0368000	PA SANTA MARTA	12.16	6.64	55%	5.52	45%	0.21	4%	2%	5.31	44%	2.14	40%	5%	3.17	26%
2003	MB0369000	PA 8 DE MARÇO	15.23	4.62	30%	10.61	70%	0.14	1%	1%	10.48	69%	0.64	6%	1%	9.84	65%
2003	MB0370000	PA NOSSA SENHORA DA GUIA	22.46	11.69	52%	10.78	48%	1.13	10%	5%	9.65	43%	5.76	60%	7%	3.89	17%
2003	MB0371000	PA VALE DO ARATAU	20.09	7.19	36%	12.90	64%	1.70	13%	7%	11.20	56%	4.62	41%	5%	6.58	33%
2003	MB0372000	PA NOVA UNIÃO	13.97	3.97	28%	10.00	72%	0.90	9%	5%	9.10	65%	2.51	28%	3%	6.59	47%
2003	MB0373000	PA COCALÂNDIA II	16.47	3.82	23%	12.65	77%	1.03	8%	4%	11.62	71%	6.06	52%	7%	5.56	34%
2003	MB0374000	PA PARAUNA	19.79	2.40	12%	17.39	88%	0.94	5%	3%	16.45	83%	4.58	28%	3%	11.86	60%
2003	MB0375000	PA REUNIDAS	43.13	7.55	18%	35.58	82%	2.43	7%	3%	33.15	77%	14.88	45%	6%	18.27	42%
2003	MB0376000	PA PALMEIRA JUSSARA	9.62	6.60	69%	3.02	31%	1.06	35%	18%	1.96	20%	0.79	40%	5%	1.17	12%
2003	MB0377000	PA CONSPEL	10.49	3.96	38%	6.54	62%	3.42	52%	26%	3.12	30%	0.70	23%	3%	2.42	23%
2003	MB0378000	PA JOÃO LANARI DO VAL	46.66	36.82	79%	9.84	21%	1.75	18%	9%	8.09	17%	1.29	16%	2%	6.80	15%
2003	MB0379000	PA CABANOS	29.10	26.61	91%	2.49	9%	0.18	7%	4%	2.30	8%	0.55	24%	3%	1.76	6%
2003	MB0380000	PA BELO VALE	12.96	8.66	67%	4.30	33%	2.13	50%	25%	2.17	17%	0.59	27%	3%	1.57	12%
2003	MB0381000	PA CASTANHAL ALMESCÃO	46.06	24.88	54%	21.17	46%	6.74	32%	16%	14.44	31%	7.44	52%	6%	7.00	15%
2003	MB0384000	PA MATA AZUL II	20.98	14.06	67%	6.93	33%	4.14	60%	30%	2.79	13%	1.46	52%	7%	1.33	6%
2003	MB0385000	PA IRMÃ DULCE	43.58	37.72	87%	5.86	13%	2.68	46%	23%	3.18	7%	1.42	45%	6%	1.76	4%

2003	MB0387000	PA AQUARIUS CRUZEIRO DO SUL	40.61	13.36	33%	27.25	67%	3.28	12%	6%	23.97	59%	10.57	44%	6%	13.41	33%
2003	MB0388000	PA BELO HORIZONTE II	23.84	17.77	75%	6.08	25%	0.29	5%	2%	5.79	24%	0.49	9%	1%	5.30	22%
2003	MB0389000	PA PEDRA DE AMOLAR	7.55	5.48	73%	2.07	27%	0.76	37%	18%	1.31	17%	0.79	61%	8%	0.52	7%
2003	MB0390000	PA ZUMBI DOS PALMARES	37.77	11.25	30%	26.51	70%	1.77	7%	3%	24.75	66%	7.38	30%	4%	17.37	46%
2003	MB0391000	PA SOL NASCENTE	12.14	8.97	74%	3.17	26%	1.64	52%	26%	1.53	13%	1.13	74%	9%	0.39	3%
2003	MB0392000	PA GABRIEL PIMENTA	31.61	24.24	77%	7.37	23%	0.31	4%	2%	7.06	22%	1.07	15%	2%	5.99	19%
2003	MB0394000	PA JOÃO VAZ	26.48	24.72	93%	1.77	7%	0.12	7%	3%	1.64	6%	0.26	16%	2%	1.38	5%
2003	MB0395000	PA LIBERDADE	56.08	37.26	66%	18.83	34%	6.50	35%	17%	12.33	22%	4.64	38%	5%	7.69	14%
2003	MB0396000	PA BOM JESUS	15.22	11.35	75%	3.88	25%	2.04	53%	26%	1.84	12%	1.07	58%	7%	0.77	5%
2003	MB0397000	PA SANTA LUCIA	12.52	8.80	70%	3.72	30%	1.58	43%	21%	2.13	17%	1.34	63%	8%	0.79	6%
2003	MB0398000	PA ESTRELA DALVA	27.48	7.40	27%	20.08	73%	0.24	1%	1%	19.84	72%	7.25	37%	5%	12.59	46%
2003	MT0640000	PA BELMONTE	12.76	12.76	100%	0.00	0%	0.00			0.00	0%	0.00		0%	0.00	0%
2003	MT0641000	PA VALE DO ARINOS	205.22	52.11	25%	153.11	75%	29.22	19%	10%	123.89	60%	58.83	47%	6%	65.06	32%
2003	MT0668000	PA KATIRA	0.83	0.00	0%	0.83	100%	0.00	0%	0%	0.83	100%	0.02	3%	0%	0.80	97%
2003	PA0258000	PA RIO DAS CRUZES	39.04	16.17	41%	22.87	59%	8.71	38%	19%	14.16	36%	8.87	63%	8%	5.29	14%
2003	PA0259000	PA ESPERANÇA	55.94	31.90	57%	24.05	43%	5.92	25%	12%	18.13	32%	4.57	25%	3%	13.56	24%
2003	PA0267000	PA PIRAMIDE	2.40	1.10	46%	1.30	54%	0.59	45%	23%	0.71	30%	0.15	22%	3%	0.55	23%
2003	PA0268000	PA BACABAL	22.99	14.83	65%	8.15	35%	3.33	41%	20%	4.83	21%	2.20	46%	6%	2.62	11%
2003	PA0269000	PA PROGRESSO	37.28	15.51	42%	21.77	58%	5.30	24%	12%	16.47	44%	7.31	44%	6%	9.16	25%
2003	PA0270000	PA NOVA SANTA MARIA	9.03	5.73	63%	3.30	37%	1.54	47%	23%	1.76	20%	0.10	6%	1%	1.67	18%
2003	PA0271000	PA TAPERUSSU	41.64	28.25	68%	13.39	32%	2.91	22%	11%	10.48	25%	2.01	19%	2%	8.47	20%

2003	PA0272000	PA VALE DO MOJU	202.85	53.79	27%	149.05	73%	13.27	9%	4%	135.78	67%	58.17	43%	5%	77.61	38%
2003	RR0039000	PA TATAJUBA	227.85	2.60	1%	225.24	99%	1.84	1%	0%	223.41	98%	15.67	7%	1%	207.73	91%
2003	RR0040000	PA AJARANI	1243.14	11.51	1%	1,231.63	99%	2.58	0%	0%	1,229.05	99%	59.73	5%	1%	1,169.31	94%
2003	AM0049000	PAE FLORA AGRÍCOLA	28.09	17.12	61%	10.97	39%	0.13	1%	1%	10.84	39%	1.05	10%	1%	9.79	35%
2003	RO0130000	PDS CERNAMBI	148.21	4.55	3%	143.66	97%	27.56	19%	10%	116.10	78%	41.33	36%	4%	74.77	50%
2003	RO0156000	PDS PORTO SEGURO	14.64	2.08	14%	12.56	86%	1.68	13%	7%	10.88	74%	1.06	10%	1%	9.81	67%
2004	AC0088000	PE POLO AGROFLORESTAL FEIJÓ	2.34	2.34	100%	0.00	0%	0.00			0.00	0%	0.00		0%	0.00	0%
2004	AC0099000	PAF PROVIDÊNCIA CAPITAL	323.06	19.66	6%	303.40	94%	1.79	1%	1%	301.62	93%	16.26	5%	1%	285.36	88%
2004	AC0101000	PA ITAMARATY	88.06	38.93	44%	49.13	56%	3.85	8%	8%	45.28	51%	9.99	22%	3%	35.29	40%
2004	AC0102000	PAF HAVAÍ	296.82	0.91	0%	295.91	100%	0.00	0%	0%	295.91	100%	0.11	0%	0%	295.79	100%
2004	AC0103000	PDS JAMIL JEREISSATI	448.02	27.71	6%	420.31	94%	1.26	0%	0%	419.05	94%	13.17	3%	0%	405.88	91%
2004	AC0104000	PA PORTO LUIZ II	19.74	1.09	6%	18.64	94%	0.02	0%	0%	18.62	94%	1.08	6%	1%	17.54	89%
2004	AC0105000	PDS PORTO LUIZ I	99.06	7.57	8%	91.50	92%	2.86	3%	3%	88.63	89%	16.62	19%	2%	72.01	73%
2004	AC0106000	PA FORTALEZA	10.01	2.45	24%	7.56	76%	0.49	7%	7%	7.06	71%	2.06	29%	4%	5.01	50%
2004	AC0107000	PA PARAGUASSU	37.82	29.15	77%	8.67	23%	1.79	21%	21%	6.88	18%	1.44	21%	3%	5.44	14%
2004	AC0108000	PA UIRAPURU	19.14	4.22	22%	14.92	78%	0.68	5%	5%	14.24	74%	3.99	28%	4%	10.24	54%
2004	AM0050000	PAE BOTOS	983.50	7.37	1%	976.13	99%	0.00	0%	0%	976.13	99%	0.33	0%	0%	975.80	99%
2004	AM0051000	PAE JENIPAPOS	385.68	13.51	4%	372.17	96%	0.32	0%	0%	371.85	96%	0.57	0%	0%	371.28	96%
2004	AM0054000	PAE CASTANHO	855.81	21.34	2%	834.47	98%	0.06	0%	0%	834.41	97%	0.99	0%	0%	833.43	97%
2004	AM0056000	PAE CANAÃ	1268.68	2.52	0%	1,266.16	100%	0.94	0%	0%	1,265.23	100%	4.09	0%	0%	1,261.13	99%
2004	AM0057000	PAE ABACAXIS	6771.72	22.99	0%	6,748.73	100%	0.48	0%	0%	6,748.25	100%	1.73	0%	0%	6,746.53	100%
2004	AM0058000	PAE CURUPIRA	1439.02	164.7	11%	1,274.30	89%	0.86	0%	0%	1,273.44	88%	21.64	2%	0%	1,251.80	87%

2004	MA0842000	PA SÃO FRANCISCO I	26.76	2<br>6.22	23%	20.54	77%	0.82	4%	4%	19.71	74%	4.49	23%	3%	15.22	57%
2004	MA0859000	PA SÃO JOÃO DO ROSÁRIO	36.77	0.14	0%	36.63	100%	0.38	1%	1%	36.25	99%	6.21	17%	2%	30.04	82%
2004	MA0860000	PA PRESIDENTE LULA	25.72	11.01	43%	14.71	57%	1.79	12%	12%	12.92	50%	5.03	39%	5%	7.89	31%
2004	MA0864000	PA CAMPO VERDE / COLONE	3.01	3.01	100%	0.00	0%	0.00			0.00	0%	0.00		0%	0.00	0%
2004	MA0865000	PA NOVA CANAÃ / COLONE	2.18	2.18	100%	0.00	0%	0.00			0.00	0%	0.00		0%	0.00	0%
2004	MA0866000	PA SANTO ANTONIO III	2.55	2.55	100%	0.00	0%	0.00			0.00	0%	0.00		0%	0.00	0%
2004	MA0869000	PA ALTO TURI III	231.76	229.2<br>6	99%	2.49	1%	0.71	28%	28%	1.79	1%	0.48	27%	3%	1.30	1%
2004	MA0870000	PA ALTO TURI II	198.33	196.4<br>9	99%	1.84	1%	0.73	40%	40%	1.11	1%	0.22	20%	2%	0.89	0%
2004	MA0871000	PA CENTRO DO ELIAS / BREJO DO LUCAS / COLONE	93.62	92.40	99%	1.22	1%	0.34	27%	27%	0.89	1%	0.00	0%	0%	0.89	1%
2004	MA0872000	PA QUADRA ÁGUA AZUL / COLONE	51.77	40.66	79%	11.10	21%	0.06	1%	1%	11.04	21%	4.41	40%	5%	6.63	13%
2004	MA0873000	PA QUADRA 21 DE ABRIL / COLONE	59.57	58.27	98%	1.30	2%	0.00	0%	0%	1.30	2%	0.25	20%	2%	1.04	2%
2004	MA0874000	PA UNIÃO/SANTO ANTONIO / COLONE	67.65	61.42	91%	6.23	9%	0.00	0%	0%	6.23	9%	4.31	69%	9%	1.93	3%
2004	MA0876000	PA FORTAL/COLONE	2.70	2.70	100%	0.00	0%	0.00			0.00	0%	0.00		0%	0.00	0%
2004	MA0877000	PA TERRA	7.38	7.38	100%	0.00	0%	0.00			0.00	0%	0.00		0%	0.00	0%

		PROMETIDA / COLONE															
2004	MA0878000	PA SÃO LUIS / COLONE	1.48	1.48	100%	0.00	0%	0.00			0.00	0%	0.00		0%	0.00	0%
2004	MA0879000	PA CENTRO DO PEDRO / BOM JESUS DA MATA/COLONE	41.40	41.40	100%	0.00	0%	0.00			0.00	0%	0.00		0%	0.00	0%
2004	MA0880000	PA VILA ESPERANÇA/COLONE	0.95	0.95	100%	0.00	0%	0.00			0.00	0%	0.00		0%	0.00	0%
2004	MA0881000	PA QUADRA BETEL / COLONE	36.31	35.82	99%	0.50	1%	0.00	0%	0%	0.50	1%	0.32	65%	8%	0.18	0%
2004	MA0882000	PA QUADRA CANAÃ/COLONE	27.04	27.04	100%	0.00	0%	0.00			0.00	0%	0.00		0%	0.00	0%
2004	MA0883000	PA QUADRA JERICÓ/COLONE	30.68	30.68	100%	0.00	0%	0.00			0.00	0%	0.00		0%	0.00	0%
2004	MA0884000	PA QUADRA BELÉM / COLONE	26.67	26.67	100%	0.00	0%	0.00			0.00	0%	0.00		0%	0.00	0%
2004	MA0885000	PA SANTA CLARA / COLONE	2.10	2.10	100%	0.00	0%	0.00			0.00	0%	0.00		0%	0.00	0%
2004	MA0886000	PA ALTO ALEGRE / COLONE	5.76	5.76	100%	0.00	0%	0.00			0.00	0%	0.00		0%	0.00	0%
2004	MA0887000	PA QUADRA BOA ESPERANÇA / COLONE	36.28	36.28	100%	0.00	0%	0.00			0.00	0%	0.00		0%	0.00	0%
2004	MA0888000	PA QUADRA SÃO FRANCISCO / COLONE	34.16	24.27	71%	9.89	29%	0.13	1%	1%	9.76	29%	4.05	41%	5%	5.71	17%

2004	MA0889000	PA QUADRA BRASIL / COLONE	40.74	40.74	100%	0.01	0%	0.00	0%	0%	0.01	0%	0.00	0%	0%	0.01	0%
2004	MA0890000	PA QUADRA PEDRO ALVARES CABRAL / COLONE	32.13	28.64	89%	3.49	11%	0.11	3%	3%	3.38	11%	0.96	28%	4%	2.42	8%
2004	MA0891000	PA QUADRA DUQUE DE CAXIAS / COLONE	49.63	49.59	100%	0.04	0%	0.00	0%	0%	0.04	0%	0.04	100%	12%	0.00	0%
2004	MA0892000	PA REDENÇÃO / BELÉM / COLONE	86.28	66.15	77%	20.13	23%	2.74	14%	14%	17.39	20%	6.68	38%	5%	10.71	12%
2004	MA0893000	PA QUADRA JERUSALÉM / COLONE	32.78	32.78	100%	0.00	0%	0.00			0.00	0%	0.00		0%	0.00	0%
2004	MA0894000	PA SÃO FRANCISCO / COLONE	1.79	1.63	91%	0.16	9%	0.00	0%	0%	0.16	9%	0.15	94%	12%	0.01	1%
2004	MA0896000	PA MORADA NOVA/CENTRO DOS PINHEIROS / COLONE	46.32	46.32	100%	0.00	0%	0.00			0.00	0%	0.00		0%	0.00	0%
2004	MA0897000	PA PEDREIRA II / INGAZAL / COLONE	12.72	12.72	100%	0.00	0%	0.00			0.00	0%	0.00		0%	0.00	0%
2004	MA0898000	PA CENTRO DOS MARTINS / AGRICOLÂNDIA / COLONE	124.39	122.42	98%	1.97	2%	0.37	19%	19%	1.60	1%	0.30	19%	2%	1.30	1%
2004	MA0901000	PA QUADRA NOVA / 13 DE JUNHO / COLONE	31.33	31.33	100%	0.00	0%	0.00			0.00	0%	0.00		0%	0.00	0%
2004	MA0902000	PA QUADRA	62.62	62.62	100%	0.00	0%	0.00			0.00	0%	0.00		0%	0.00	0%

		BENEDITO MENDES / QUADRA FORTALEZA / COLONE															
2004	MA0906000	PA QUADRA SÃO RAIMUNDO III / COLONE	181.56	101.85	56%	79.71	44%	20.50	26%	26%	59.21	33%	17.34	29%	4%	41.87	23%
2004	MA0909000	PA QUADRA SABIÁ / COLONE	53.36	45.90	86%	7.47	14%	0.00	0%	0%	7.47	14%	3.76	50%	6%	3.71	7%
2004	MA0911000	PA ITAMATARÉ / COLONE	231.92	159.15	69%	72.77	31%	9.47	13%	13%	63.30	27%	16.83	27%	3%	46.47	20%
2004	MA0912000	PA PINDORAMA / ARARIBÓIA / COLONE	305.65	267.47	88%	38.19	12%	3.73	10%	10%	34.46	11%	18.34	53%	7%	16.12	5%
2004	MA0913000	PA 15 DE JUNHO / IGARAPÉ AREIA / SÃO JOSE II / COLONE	76.40	75.41	99%	0.99	1%	0.06	7%	7%	0.93	1%	0.30	33%	4%	0.62	1%
2004	MA0914000	PA FÉ EM DEUS / COLONIAL / COLONE	150.30	150.30	100%	0.00	0%	0.00			0.00	0%	0.00		0%	0.00	0%
2004	MA0915000	PA JACI / COLONE	67.08	65.08	97%	2.00	3%	0.15	7%	7%	1.85	3%	0.66	35%	4%	1.19	2%
2004	MA0916000	PA SÃO JOÃO / 16 DE OUTUBRO / COLONE	41.47	41.47	100%	0.00	0%	0.00			0.00	0%	0.00		0%	0.00	0%
2004	MA0917000	PA NOVA VIDA / SERPETIARA / COLONE	49.62	45.53	92%	4.08	8%	0.00	0%	0%	4.08	8%	0.20	5%	1%	3.89	8%
2004	MA0918000	PA QUADRA	50.11	44.20	88%	5.91	12%	0.00	0%	0%	5.91	12%	3.12	53%	7%	2.79	6%

		CASTELO BRANCO / COLONE															
2004	MB0218000	PA VALE DO MUCURA	108.04	80.99	75%	27.06	25%	10.60	39%	39%	16.45	15%	7.41	45%	6%	9.04	8%
2004	MB0401000	PA GROTÃO DO SEVERINO	7.63	6.81	89%	0.82	11%	0.30	37%	37%	0.52	7%	0.35	68%	9%	0.16	2%
2004	MB0402000	PA TALISMÃ	9.87	2.73	28%	7.13	72%	0.00	0%	0%	7.13	72%	3.96	55%	7%	3.18	32%
2004	MB0404000	PA CANUDOS	27.42	21.41	78%	6.00	22%	0.50	8%	8%	5.50	20%	3.76	68%	9%	1.75	6%
2004	MB0405000	PA TARUMÃ	44.05	37.16	84%	6.89	16%	0.00	0%	0%	6.89	16%	3.08	45%	6%	3.81	9%
2004	MB0406000	PA ESPERANÇA	22.64	13.44	59%	9.20	41%	0.59	6%	6%	8.61	38%	6.25	73%	9%	2.35	10%
2004	MB0407000	PA LIMEIRA	13.43	9.79	73%	3.64	27%	0.00	0%	0%	3.64	27%	2.27	62%	8%	1.38	10%
2004	MB0408000	PA ANTARES	70.32	39.15	56%	31.18	44%	1.67	5%	5%	29.51	42%	14.96	51%	6%	14.55	21%
2004	MB0409000	PA PARAGOMINAS	16.00	15.32	96%	0.69	4%	0.09	13%	13%	0.60	4%	0.13	22%	3%	0.47	3%
2004	MB0411000	PA CARUMBÉ	28.52	21.35	75%	7.17	25%	2.67	37%	37%	4.50	16%	3.14	70%	9%	1.36	5%
2004	MB0412000	PA UNIÃO DO ANGICO	28.03	13.80	49%	14.23	51%	2.85	20%	20%	11.38	41%	9.55	84%	10%	1.83	7%
2004	MB0413000	PA MUTUÃ	35.99	21.37	59%	14.63	41%	5.72	39%	39%	8.91	25%	5.59	63%	8%	3.31	9%
2004	MB0417000	PA RAINHA DA PAZ	39.23	25.79	66%	13.44	34%	0.72	5%	5%	12.71	32%	3.60	28%	4%	9.11	23%
2004	MB0418000	PA GAVIÃO	104.74	65.81	63%	38.94	37%	5.27	14%	14%	33.66	32%	9.52	28%	4%	24.14	23%
2004	MB0419000	PA MANTENHA	11.71	8.70	74%	3.01	26%	0.69	23%	23%	2.32	20%	1.30	56%	7%	1.02	9%
2004	MB0420000	PA CACHOEIRA PRETA II	71.25	40.49	57%	30.76	43%	0.00	0%	0%	30.76	43%	13.01	42%	5%	17.75	25%
2004	MT0719000	PA FLOR DA MATA	4.30	0.17	4%	4.13	96%	0.04	1%	1%	4.09	95%	0.56	14%	2%	3.53	82%
2004	PA0255000	PA RIO ACAMPAMENTO	64.79	46.79	72%	18.01	28%	3.33	19%	19%	14.67	23%	8.89	61%	8%	5.78	9%
2004	PA0273000	PA INÁCIA	26.58	16.21	61%	10.37	39%	0.00	0%	0%	10.37	39%	5.85	56%	7%	4.52	17%
2004	PA0274000	PA JARARACA	13.81	8.13	59%	5.67	41%	0.00	0%	0%	5.67	41%	3.29	58%	7%	2.39	17%

2004	PA0275000	PAE SÃO JOÃO BATISTA	3.91	0.00	0%	3.91	100%	0.00	0%	0%	3.91	100%	0.00	0%	0%	3.91	100%
2004	PA0276000	PAE NOSSA SENHORA DO LIVRAMENTO	1.81	0.00	0%	1.81	100%	0.00	0%	0%	1.81	100%	0.00	0%	0%	1.81	100%
2004	PA0278000	PCA ELIZABETE TEIXEIRA	0.72	0.28	39%	0.44	61%	0.00	0%	0%	0.44	61%	0.00	0%	0%	0.44	61%
2004	RO0157000	PA MIGRANTES	20.24	15.90	79%	4.34	21%	0.32	7%	7%	4.02	20%	0.27	7%	1%	3.75	19%
2004	RO0158000	PA ALTAMIRA	99.32	58.04	58%	41.28	42%	6.03	15%	15%	35.25	35%	9.82	28%	3%	25.42	26%
2004	RO0159000	PA SANTA ELISA	68.40	49.24	72%	19.16	28%	2.07	11%	11%	17.09	25%	8.62	50%	6%	8.47	12%
2004	SM0067000	PDS ANAPU I	240.14	19.40	8%	220.73	92%	6.48	3%	3%	214.25	89%	28.73	13%	2%	185.52	77%
2004	SM0069000	PDS ANAPU III	242.57	1.98	1%	240.59	99%	0.11	0%	0%	240.47	99%	0.00	0%	0%	240.47	99%
2004	SM0070000	PDS ANAPU IV	61.40	1.77	3%	59.63	97%	0.20	0%	0%	59.43	97%	4.01	7%	1%	55.42	90%
2004	SM0073000	PDS CUPARI	299.99	0.58	0%	299.41	100%	0.00	0%	0%	299.41	100%	5.95	2%	0%	293.46	98%
2004	SM0074000	PDS DIVINÓPOLIS	294.86	5.15	2%	289.72	98%	0.18	0%	0%	289.54	98%	18.52	6%	1%	271.02	92%
2004	SM0075000	PDS NOVO MUNDO	299.94	4.42	1%	295.53	99%	0.10	0%	0%	295.42	98%	3.26	1%	0%	292.16	97%
2004	TO0317000	PA FORTALEZA	3.34	3.34	100%	0.00	0%	0.00			0.00	0%	0.00		0%	0.00	0%
2005	AC0100000	PA ARCO-IRIS	14.04	4.24	30%	9.79	70%				9.79	70%	2.20	22%	3%	7.60	54%
2005	AC0109000	PA JOÃO ADEMIR	2.58	0.42	16%	2.15	84%				2.15	84%	0.45	21%	3%	1.71	66%
2005	AC0110000	PA CASTELO	4.76	4.15	87%	0.62	13%				0.62	13%	0.14	23%	3%	0.47	10%
2005	AC0116000	PA PETROLINA	32.24	25.11	78%	7.13	22%				7.13	22%	0.95	13%	2%	6.18	19%
2005	AC0118000	PA JOÃO BATISTA	39.24	9.89	25%	29.35	75%				29.35	75%	4.88	17%	2%	24.47	62%
2005	AC0121000	PA ALBERTO SANTIAGO	21.69	11.10	51%	10.59	49%				10.59	49%	1.56	15%	2%	9.04	42%
2005	AC0117000	PAF VALENCIA	213.18	14.92	7%	198.26	93%				198.26	93%	9.17	5%	1%	189.10	89%
2005	AM0061000	PA ESPIGÃO DO ARARA	48.21	5.71	12%	42.50	88%				42.50	88%	2.56	6%	1%	39.94	83%

2005	AM0062000	PA MANAQUIRI I - GLEBA 06	39.97	5.94	15%	34.04	85%	34.04	85%	0.82	2%	0%	33.21	83%
2005	AM0063000	PA MANAQUIRI II - GLEBA 07	67.86	10.15	15%	57.71	85%	57.71	85%	0.58	1%	0%	57.12	84%
2005	AP0038000	PA FERREIRINHA	54.58	7.95	15%	46.63	85%	46.63	85%	1.24	3%	0%	45.40	83%
2005	MA0747000	PA RAIMUNDO PANELADA/SIMASA	69.95	44.93	64%	25.02	36%	25.02	36%	8.52	34%	4%	16.50	24%
2005	AC0124000	PAF RECANTO	90.08	1.10	1%	88.98	99%	88.98	99%	2.04	2%	0%	86.93	97%
2005	MA0748000	PA SÃO JOÃO I	8.15	7.19	88%	0.96	12%	0.96	12%	0.52	54%	7%	0.44	5%
2005	MA0836000	PA DEUS PROTEJA	22.71	13.37	59%	9.33	41%	9.33	41%	2.82	30%	4%	6.52	29%
2005	MA0921000	PA SANTA TEREZA / COLONE	1.53	1.39	90%	0.15	10%	0.15	10%	0.00	1%	0%	0.15	10%
2005	MA0924000	PA SÃO JUDAS TADEU	7.27	7.27	100%	0.01	0%	0.01	0%	0.00	0%	0%	0.01	0%
2005	MA0925000	PA CONCEIÇÃO I	8.48	8.48	100%	0.00	0%	0.00	0%	0.00		0%	0.00	0%
2005	MA0926000	PA SÃO PEDRO / COLONE	3.81	3.81	100%	0.00	0%	0.00	0%	0.00		0%	0.00	0%
2005	MA0927000	PA 22 DE SETEMBRO / SANTA TEREZA / COLONE	265.51	265.24	100%	0.27	0%	0.27	0%	0.10	38%	5%	0.16	0%
2005	MA0928000	PA CAFÉ DA MATA / COLONE	29.55	29.55	100%	0.00	0%	0.00	0%	0.00		0%	0.00	0%
2005	MA0929000	PA MATA AZUL / COLONE	24.24	13.11	54%	11.14	46%	11.14	46%	4.83	43%	5%	6.31	26%
2005	MA0931000	PA GRACILÂNDIA / CIDELÂNDIA / COLONE	153.84	151.81	99%	2.02	1%	2.02	1%	0.11	6%	1%	1.91	1%

2005	MA0932000	PA NADIR / SÃO VICENTE / COLONE	295.47	206.68	70%	88.79	30%	88.79	30%	25.30	28%	4%	63.49	21%
2005	MA0933000	PA SÃO FRANCISCO II / COLONE	1.48	1.48	100%	0.00	0%	0.00	0%	0.00		0%	0.00	0%
2005	MA0934000	PA FELIZ UNIÃO / COLONE	3.24	3.24	100%	0.00	0%	0.00	0%	0.00		0%	0.00	0%
2005	MA0935000	PA NOSSA VITÓRIA / COLONE	2.41	2.41	100%	0.00	0%	0.00	0%	0.00		0%	0.00	0%
2005	MA0939000	PA LEELAU /CAJUEIRO/COLONE	10.87	10.87	100%	0.00	0%	0.00	0%	0.00	0%	0%	0.00	0%
2005	MA0940000	PA ABAIXADINHO / COLONE	16.93	10.10	60%	6.83	40%	6.83	40%	2.35	34%	4%	4.47	26%
2005	MA0941000	PA UBINZAL / COLONE	155.03	152.27	98%	2.76	2%	2.76	2%	1.06	39%	5%	1.70	1%
2005	MA0944000	PA TATAJUBA / 10 DE ABRIL / COLONE	41.30	41.13	100%	0.17	0%	0.17	0%	0.15	87%	11%	0.02	0%
2005	MA0946000	PA MARACAÇUMÉ / RICOA	15.90	15.86	100%	0.05	0%	0.05	0%	0.05	100%	13%	0.00	0%
2005	MA0947000	PA JUSSARAL DO ANTONIO ROXO	8.09	8.09	100%	0.00	0%	0.00	0%	0.00		0%	0.00	0%
2005	MA0820000	PE BOA VISTA III	48.03	43.79	91%	4.25	9%	4.25	9%	0.00	0%	0%	4.25	9%
2005	MA0821000	PE ENCRUSO	41.94	0.42	1%	41.52	99%	41.52	99%	3.24	8%	1%	38.28	91%
2005	MA0948000	PA CUBA	7.41	2.84	38%	4.57	62%	4.57	62%	0.82	18%	2%	3.75	51%
2005	MA0969000	PA FLECHAL	171.84	132.92	77%	38.92	23%	38.92	23%	13.20	34%	4%	25.72	15%
2005	MA0973000	PA VILA NOVA	19.99	1.81	9%	18.18	91%	18.18	91%	3.16	17%	2%	15.02	75%
2005	MA0974000	PA NÚCLEO A - I /	96.45	96.45	100%	0.00	0%	0.00	0%	0.00		0%	0.00	0%

		COLONE												
2005	MA0975000	PA BOQUEIRÃO I / COLONE	3.77	3.50	93%	0.26	7%	0.26	7%	0.20	77%	10%	0.06	2%
2005	MA0976000	PA QUADRA PIMENTEIRA / COLONE	15.48	15.48	100%	0.00	0%	0.00	0%	0.00		0%	0.00	0%
2005	MA0977000	PA QUADRA 10 DE JANEIRO / COLONE	12.61	12.61	100%	0.00	0%	0.00	0%	0.00		0%	0.00	0%
2005	MA0978000	PA QUADRA NOVA PIMENTEIRA / COLONE	12.50	12.50	100%	0.00	0%	0.00	0%	0.00		0%	0.00	0%
2005	MA0979000	PA DIBOM I	31.07	30.87	99%	0.19	1%	0.19	1%	0.08	41%	5%	0.11	0%
2005	MA0980000	PA DIBOM II	16.02	15.50	97%	0.52	3%	0.52	3%	0.12	23%	3%	0.40	2%
2005	MA0981000	PA BOM JESUS III	20.66	0.00	0%	20.66	100%	20.66	100%	3.59	17%	2%	17.06	83%
2005	MA0982000	PA BOCA DA MATA I	24.02	16.89	70%	7.12	30%	7.12	30%	3.76	53%	7%	3.37	14%
2005	MB0422000	PA PADRE JOSIMO TAVARES II	10.20	5.56	55%	4.64	45%	4.64	45%	2.89	62%	8%	1.75	17%
2005	MB0423000	PA REINO ENCANTADO	23.27	6.30	27%	16.97	73%	16.97	73%	5.76	34%	4%	11.21	48%
2005	MB0424000	PA DOMINGOS OLIVEIRA BEZERRA	68.26	38.18	56%	30.08	44%	30.08	44%	11.12	37%	5%	18.96	28%
2005	MB0425000	PA SÃO SEBASTIÃO DO CRISTALINO	47.84	24.25	51%	23.59	49%	23.59	49%	7.75	33%	4%	15.84	33%
2005	MB0426000	PA SÃO PEDRO II	55.07	10.67	19%	44.40	81%	44.40	81%	21.30	48%	6%	23.09	42%
2005	MB0427000	PA PIONEIRA	20.36	8.49	42%	11.87	58%	11.87	58%	4.13	35%	4%	7.74	38%
2005	MB0428000	PA RIBEIRÃO DAS PEDRAS	20.17	9.74	48%	10.42	52%	10.42	52%	3.43	33%	4%	7.00	35%

2005	MB0430000	PA ARIZONA	133.09	97.22	73%	35.87	27%	35.87	27%	12.86	36%	4%	23.01	17%
2005	MB0432000	PA PANORAMA	35.82	34.09	95%	1.73	5%	1.73	5%	0.43	25%	3%	1.30	4%
2005	MB0433000	PA CRISTO VIVE	66.77	29.34	44%	37.42	56%	37.42	56%	11.97	32%	4%	25.45	38%
2005	MB0434000	PA SERRA GRANDE	58.98	27.41	46%	31.58	54%	31.58	54%	10.91	35%	4%	20.67	35%
2005	MB0435000	PA NOVA INDEPENDÊNCIA	60.61	16.89	28%	43.71	72%	43.71	72%	12.66	29%	4%	31.06	51%
2005	MB0436000	PA ITAMARATY	76.11	36.63	48%	39.48	52%	39.48	52%	20.37	52%	6%	19.10	25%
2005	MB0437000	PA ÁGUA BOA	7.04	7.04	100%	0.00	0%	0.00	0%	0.00		0%	0.00	0%
2005	MB0438000	PA SUMAUMA II	50.74	13.15	26%	37.58	74%	37.58	74%	19.62	52%	7%	17.96	35%
2005	MB0439000	PA CAMPOS VERDES	1.67	0.69	42%	0.98	58%	0.98	58%	0.00	0%	0%	0.98	58%
2005	MB0440000	PA BOM FUTURO	29.37	1.65	6%	27.72	94%	27.72	94%	6.39	23%	3%	21.33	73%
2005	MB0441000	PA SUNIL	23.58	4.11	17%	19.46	83%	19.46	83%	9.18	47%	6%	10.28	44%
2005	MB0442000	PA JORDÃO	30.30	4.63	15%	25.67	85%	25.67	85%	11.23	44%	5%	14.44	48%
2005	MA0994000	PE TATAJUBAL	3.84	3.58	93%	0.26	7%	0.26	7%	0.16	62%	8%	0.10	3%
2005	MB0443000	PA CARAJÁS	50.98	2.85	6%	48.13	94%	48.13	94%	20.10	42%	5%	28.03	55%
2005	MB0444000	PA VIDA NOVA	30.53	8.05	26%	22.48	74%	22.48	74%	11.57	51%	6%	10.91	36%
2005	MB0445000	PA BARRACA DO MEIO	43.78	19.21	44%	24.56	56%	24.56	56%	9.36	38%	5%	15.20	35%
2005	MB0446000	PA UXI	79.54	34.84	44%	44.70	56%	44.70	56%	18.44	41%	5%	26.26	33%
2005	MB0448000	PA PEDRA BRANCA	186.51	95.66	51%	90.85	49%	90.85	49%	39.23	43%	5%	51.62	28%
2005	MB0450000	PA RIO BANDEIRA	111.48	8.72	8%	102.76	92%	102.76	92%	11.54	11%	1%	91.21	82%
2005	MB0451000	PA SANTIAGO	37.19	0.87	2%	36.32	98%	36.32	98%	15.53	43%	5%	20.79	56%
2005	MB0452000	PA NOVA DESCOBERTA	32.54	31.61	97%	0.94	3%	0.94	3%	0.00	0%	0%	0.94	3%
2005	MB0453000	PA LIMPEZA	37.74	36.84	98%	0.90	2%	0.90	2%	0.06	7%	1%	0.83	2%
2005	MB0454000	PA MAL ARRUMADO	9.90	9.35	94%	0.55	6%	0.55	6%	0.15	26%	3%	0.41	4%
2005	MB0455000	PA JOSÉ INOCÊNCIO	2.14	0.65	31%	1.48	69%	1.48	69%	0.00	0%	0%	1.48	69%

		NERES												
2005	MB0456000	PA ÁGUA BRANCA	32.33	6.11	19%	26.23	81%	26.23	81%	17.18	65%	8%	9.05	28%
2005	MB0458000	PA COLÔNIA PODEROSA	70.63	36.99	52%	33.64	48%	33.64	48%	17.37	52%	6%	16.27	23%
2005	MB0459000	PA OESTE	58.21	33.67	58%	24.54	42%	24.54	42%	10.03	41%	5%	14.51	25%
2005	MB0460000	PA RIO CURURUÍ	314.12	36.55	12%	277.57	88%	277.57	88%	22.10	8%	1%	255.47	81%
2005	MB0461000	PA PETRONÍLIO ALVES BATISTA	145.52	27.01	19%	118.52	81%	118.52	81%	29.08	25%	3%	89.43	61%
2005	MB0462000	PA NOVA JERUZALÉM	5.77	5.63	98%	0.14	2%	0.14	2%	0.14	100%	13%	0.00	0%
2005	MB0463000	PA SÃO VICENTE	55.46	10.32	19%	45.15	81%	45.15	81%	14.63	32%	4%	30.51	55%
2005	MB0467000	PA HAMILTON CORDEIRO CELSO	138.84	46.31	33%	92.53	67%	92.53	67%	47.99	52%	6%	44.54	32%
2005	MB0469000	PA CENTRO NOVO	27.09	25.38	94%	1.72	6%	1.72	6%	0.07	4%	1%	1.64	6%
2005	MB0472000	PA BACURIZINHO	13.76	7.54	55%	6.22	45%	6.22	45%	3.80	61%	8%	2.42	18%
2005	MB0473000	PA SOL NASCENTE	29.63	11.73	40%	17.90	60%	17.90	60%	3.22	18%	2%	14.68	50%
2005	MT0724000	PA ALIANÇA	4.89	1.38	28%	3.52	72%	3.52	72%	2.64	75%	9%	0.87	18%
2005	MT0725000	PA CRISTALINO	24.22	0.42	2%	23.80	98%	23.80	98%	11.60	49%	6%	12.20	50%
2005	MT0726000	PA CRISTALINO II	8.83	2.68	30%	6.15	70%	6.15	70%	3.13	51%	6%	3.02	34%
2005	MT0727000	PA CRISTALINO - IV	6.75	3.42	51%	3.34	49%	3.34	49%	1.77	53%	7%	1.57	23%
2005	MT0731000	PA ANTONIO SOARES	159.73	9.26	6%	150.47	94%	150.47	94%	35.10	23%	3%	115.37	72%
2005	MT0733000	PA NOSSA SENHORA DE FÁTIMA	24.25	24.23	100%	0.02	0%	0.02	0%	0.00	0%	0%	0.02	0%
2005	MT0734000	PA PADRE JOSÉ TENCATE	13.24	11.25	85%	1.99	15%	1.99	15%	0.00	0%	0%	1.99	15%
2005	MT0735000	PA UIRAPURU	5.42	4.39	81%	1.03	19%	1.03	19%	0.41	40%	5%	0.62	11%
2005	MT0736000	PA LOURIVAL D'ABIC	11.99	6.22	52%	5.76	48%	5.76	48%	3.35	58%	7%	2.41	20%

2005	MT0737000	PA VIDA NOVA II	97.72	11.56	12%	86.16	88%	86.16	88%	24.47	28%	4%	61.69	63%
2005	MT0746000	PA SANTO ANTONIO	7.97	5.62	71%	2.35	29%	2.35	29%	0.37	16%	2%	1.98	25%
2005	MT0768000	PA SANTO ANTONIO I	6.67	5.88	88%	0.79	12%	0.79	12%	0.32	40%	5%	0.47	7%
2005	MT0769000	PA SANTO ANTONIO II	7.62	7.56	99%	0.06	1%	0.06	1%	0.00	0%	0%	0.06	1%
2005	MT0776000	PA PLANALTO DO IRIRI	159.18	5.59	4%	153.59	96%	153.59	96%	59.94	39%	5%	93.65	59%
2005	MT0777000	PA ARAUNA	29.05	0.67	2%	28.38	98%	28.38	98%	17.42	61%	8%	10.97	38%
2005	MT0778000	PA ARAUNA II	11.51	0.00	0%	11.51	100%	11.51	100%	6.55	57%	7%	4.97	43%
2005	PA0286000	PA MANDACARU	43.88	29.24	67%	14.63	33%	14.63	33%	9.32	64%	8%	5.31	12%
2005	PA0287000	PA DIAMANTINA II	22.61	17.66	78%	4.95	22%	4.95	22%	1.66	33%	4%	3.29	15%
2005	PA0288000	PA ALTA FLORESTA	40.18	13.42	33%	26.75	67%	26.75	67%	10.72	40%	5%	16.04	40%
2005	PA0289000	PA TERRA NOVA	57.35	47.54	83%	9.81	17%	9.81	17%	3.55	36%	5%	6.26	11%
2005	PA0290000	PA SUÇUARANA	42.22	28.98	69%	13.24	31%	13.24	31%	8.62	65%	8%	4.61	11%
2005	RR0041000	PA PAU RAINHA	118.30	3.08	3%	115.22	97%	115.22	97%	3.63	3%	0%	111.59	94%
2005	RR0042000	PA CUPIUBA	132.45	3.29	2%	129.16	98%	129.16	98%	4.32	3%	0%	124.84	94%
2005	RR0043000	PA CASTANHEIRA	103.22	0.45	0%	102.76	100%	102.76	100%	3.08	3%	0%	99.68	97%
2005	RR0044000	PA ANGELIN	55.51	1.24	2%	54.27	98%	54.27	98%	2.38	4%	1%	51.89	93%
2005	RR0045000	PA SERINGUEIRA	121.19	4.43	4%	116.76	96%	116.76	96%	8.85	8%	1%	107.91	89%
2005	RR0046000	PA JATOBA	195.80	9.52	5%	186.28	95%	186.28	95%	7.37	4%	0%	178.91	91%
2005	SM0105000	PA MARIPÁ	85.88	59.61	69%	26.27	31%	26.27	31%	2.48	9%	1%	23.79	28%
2005	SM0106000	PA BAIXÃO	100.60	64.00	64%	36.60	36%	36.60	36%	5.42	15%	2%	31.18	31%
2005	SM0107000	PA MORIÇOCA	8.85	5.22	59%	3.63	41%	3.63	41%	1.02	28%	4%	2.61	29%
2005	SM0108000	PA VAI QUEM QUER	20.54	9.84	48%	10.70	52%	10.70	52%	1.68	16%	2%	9.02	44%
2005	SM0109000	PA MIRITI	126.64	87.11	69%	39.53	31%	39.53	31%	9.69	25%	3%	29.84	24%
2005	SM0110000	PA TERRA PRETA E	34.64	18.65	54%	15.99	46%	15.99	46%	2.35	15%	2%	13.64	39%

		OLHO D'ÁGUA												
2005	SM0111000	PA CURUMU	14.37	12.50	87%	1.87	13%	1.87	13%	0.52	28%	3%	1.35	9%
2005	SM0112000	PA CAMBURÃO I	129.12	118.60	92%	10.53	8%	10.53	8%	0.94	9%	1%	9.58	7%
2005	SM0114000	PA URUCURITUBA	151.45	44.33	29%	107.12	71%	107.12	71%	6.78	6%	1%	100.33	66%
2005	SM0115000	PA SANTA CRUZ	217.73	35.75	16%	181.98	84%	181.98	84%	2.99	2%	0%	178.98	82%
2005	SM0116000	PA DANIEL DE CARVALHO	193.49	45.62	24%	147.86	76%	147.86	76%	6.35	4%	1%	141.51	73%
2005	SM0117000	PA BRASÍLIA LEGAL	244.38	120.26	49%	124.12	51%	124.12	51%	17.13	14%	2%	106.99	44%
2005	SM0119000	PA CAMBURÃO II	39.54	20.54	52%	19.00	48%	19.00	48%	3.37	18%	2%	15.63	40%
2005	SM0120000	PA PORÃO	27.44	26.96	98%	0.48	2%	0.48	2%	0.09	19%	2%	0.39	1%
2005	SM0128000	PA RENASCER	85.80	16.12	19%	69.68	81%	69.68	81%	21.86	31%	4%	47.81	56%
2005	SM0129000	PA CUPUZAL	113.02	28.32	25%	84.70	75%	84.70	75%	18.83	22%	3%	65.87	58%
2005	SM0130000	PA TERRA PARA PAZ	641.63	14.00	2%	627.63	98%	627.63	98%	23.03	4%	0%	604.61	94%
2005	SM0131000	PA JAMARY	314.48	140.51	45%	173.97	55%	173.97	55%	12.21	7%	1%	161.76	51%
2005	SM0135000	PA CRISTO REI	75.97	75.97	100%	0.00	0%	0.00	0%	0.00		0%	0.00	0%
2005	SM0136000	PA REPARTIMENTO	375.16	220.64	59%	154.52	41%	154.52	41%	18.61	12%	2%	135.91	36%
2005	SM0137000	PA MAMURU	214.68	131.50	61%	83.18	39%	83.18	39%	8.13	10%	1%	75.05	35%
2005	SM0138000	PA CURUMU II	225.85	124.94	55%	100.90	45%	100.90	45%	9.84	10%	1%	91.07	40%
2005	SM0139000	PA CIPOAL	348.62	237.69	68%	110.93	32%	110.93	32%	22.29	20%	3%	88.64	25%
2005	SM0140000	PA VALE DO AÇAÍ	178.51	138.9	78%	39.56	22%	39.56	22%	6.81	17%	2%	32.76	18%

				4										
2005	SM0143000	PA ITAQUERA I	318.55	88.54	28%	230.02	72%	230.02	72%	15.15	7%	1%	214.87	67%
2005	SM0145000	PA CURUÁ	758.58	130.55	17%	628.04	83%	628.04	83%	21.00	3%	0%	607.04	80%
2005	SM0147000	PA VIRA VOLTA	33.89	14.12	42%	19.77	58%	19.77	58%	3.53	18%	2%	16.24	48%
2005	SM0148000	PA ACOMEC	84.72	13.09	15%	71.64	85%	71.64	85%	4.90	7%	1%	66.73	79%
2005	TO0327000	PA MARÍLIA	26.88	25.82	96%	1.06	4%	1.06	4%	0.30	28%	4%	0.76	3%
2005	TO0328000	PA ORLÂNDIA	7.59	7.17	94%	0.42	6%	0.42	6%	0.10	24%	3%	0.32	4%
2005	TO0330000	PA 20 MIL	9.85	9.85	100%	0.00	0%	0.00	0%	0.00		0%	0.00	0%
2005	TO0372000	PA LAGO DA UMBAÚBA	0.05	0.05	100%	0.00	0%	0.00	0%	0.00		0%	0.00	0%
2005	AC0115000	PAE BARREIRO	81.61	2.82	3%	78.79	97%	78.79	97%	2.57	3%	0%	76.22	93%
2005	AM0059000	PAE ABACAXIS II	2757.33	43.43	2%	2,713.90	98%	2,713.90	98%	5.15	0%	0%	2,708.75	98%
2005	AM0060000	PAE TROCANÃ	689.39	29.78	4%	659.60	96%	659.60	96%	5.00	1%	0%	654.60	95%
2005	AM0065000	PAE INAJÁ	498.37	26.64	5%	471.72	95%	471.72	95%	2.56	1%	0%	469.17	94%
2005	AM0066000	PAE ACARÁ	1665.60	48.80	3%	1,616.80	97%	1,616.80	97%	18.27	1%	0%	1,598.53	96%
2005	AM0068000	PAE PIRANHA	430.32	2.82	1%	427.50	99%	427.50	99%	7.14	2%	0%	420.36	98%
2005	AM0069000	PAE NOVO JARDIM	354.11	23.14	7%	330.97	93%	330.97	93%	8.26	2%	0%	322.72	91%
2005	AM0073000	PAE TERRA FIRME	73.60	2.09	3%	71.51	97%	71.51	97%	0.98	1%	0%	70.54	96%
2005	AM0074000	PAE ARIPUANÃ-GUARIBA	10418.41	105.32	1%	10,313.09	99%	10,313.09	99%	48.74	0%	0%	10,264.35	99%
2005	AM0080000	PAE SÃO BENEDITO	1935.67	7.46	0%	1,928.21	100%	1,928.21	100%	3.20	0%	0%	1,925.00	99%
2005	AM0082000	PAE TUPANA IGAPÓ-AÃU I	1569.83	4.03	0%	1,565.80	100%	1,565.80	100%	0.35	0%	0%	1,565.45	100%
2005	PA0291000	PAE ILHA DAS ONÇAS	73.48	0.17	0%	73.31	100%	73.31	100%	0.27	0%	0%	73.05	99%
2005	PA0292000	PAE JOÃO PILATOS	36.62	2.25	6%	34.38	94%	34.38	94%	0.66	2%	0%	33.72	92%

2005	PA0293000	PAE SANTO ANTONIO	13.17	0.00	0%	13.17	100%	13.17	100%	0.00	0%	0%	13.17	100%
2005	PA0294000	PAE NOSSA SENHORA DE NAZARÉ	9.73	0.18	2%	9.55	98%	9.55	98%	0.00	0%	0%	9.55	98%
2005	PA0295000	PAE NOSSA SENHORA DO PERPETUO SOCORRO	27.28	1.34	5%	25.94	95%	25.94	95%	0.34	1%	0%	25.61	94%
2005	PA0296000	PAE ILHA SANTA ROSA	3.66	0.04	1%	3.62	99%	3.62	99%	0.08	2%	0%	3.54	97%
2005	PA0298000	PAE ILHA VIÇOSA	3.27	0.00	0%	3.27	100%	3.27	100%	0.00	0%	0%	3.27	100%
2005	PA0299000	PAE ILHA ARAPIRANGA	34.68	1.30	4%	33.38	96%	33.38	96%	0.13	0%	0%	33.25	96%
2005	PA0300000	PAE SANTA MARIA	6.75	0.00	0%	6.75	100%	6.75	100%	0.00	0%	0%	6.75	100%
2005	PA0301000	PAE SANTO AFONSO	25.59	10.34	40%	15.24	60%	15.24	60%	0.17	1%	0%	15.07	59%
2005	PA0302000	PAE NOSSA SENHORA DAS GRAÇAS	18.54	2.23	12%	16.31	88%	16.31	88%	0.00	0%	0%	16.31	88%
2005	PA0303000	PAE SANTO ANTONIO II	8.72	2.10	24%	6.63	76%	6.63	76%	0.00	0%	0%	6.63	76%
2005	PA0304000	PAE SÃO FRANCISCO DE ASSIS	4.31	0.00	0%	4.31	100%	4.31	100%	0.00	0%	0%	4.31	100%
2005	PA0305000	PAE SÃO RAIMUNDO	24.78	5.38	22%	19.41	78%	19.41	78%	0.14	1%	0%	19.27	78%
2005	SM0104000	PAE LAGO GRANDE	2557.36	1134.05	44%	1,423.31	56%	1,423.31	56%	18.56	1%	0%	1,404.76	55%
2005	SM0113000	PAE JURUTI VELHO	854.29	227.27	27%	627.03	73%	627.03	73%	12.52	2%	0%	614.51	72%

2005	SM0121000	PAE MISSIONÁRIO RUFINO	57.59	19.98	35%	37.61	65%	37.61	65%	1.59	4%	1%	36.02	63%
2005	SM0122000	PAE VALE DO SALGADO	169.29	104.67	62%	64.61	38%	64.61	38%	16.71	26%	3%	47.91	28%
2005	SM0141000	PAE EIXO FORTE	163.52	73.98	45%	89.53	55%	89.53	55%	10.14	11%	1%	79.39	49%
2005	AC0114000	PDS NOVA ESPERANÇA	31.23	2.06	7%	29.18	93%	29.18	93%	5.16	18%	2%	24.02	77%
2005	AC0119000	PDS BONAL	112.23	16.55	15%	95.68	85%	95.68	85%	0.50	1%	0%	95.18	85%
2005	AC0122000	PDS MINAS	28.94	0.27	1%	28.67	99%	28.67	99%	0.99	3%	0%	27.68	96%
2005	AC0123000	PDS FRANCISCO PIMENTEL	543.59	20.22	4%	523.36	96%	523.36	96%	5.01	1%	0%	518.35	95%
2005	AM0064000	PDS MANDIOCA	48.74	30.98	64%	17.77	36%	17.77	36%	1.37	8%	1%	16.39	34%
2005	AM0067000	PDS RAINHA	196.95	7.66	4%	189.29	96%	189.29	96%	4.18	2%	0%	185.10	94%
2005	AM0070000	PDS CUIEIRAS/ANAVILHANAS	1900.05	18.38	1%	1,881.67	99%	1,881.67	99%	1.33	0%	0%	1,880.34	99%
2005	AP0037000	PDS - IRINEU E FELIPE	99.98	0.54	1%	99.44	99%	99.44	99%	0.80	1%	0%	98.63	99%
2005	SM0099000	PDS SERRA AZUL	739.45	7.23	1%	732.22	99%	732.22	99%	30.99	4%	1%	701.23	95%
2005	SM0101000	PDS ADEMIR FEDERICCE	2331.50	77.33	3%	2,254.17	97%	2,254.17	97%	54.09	2%	0%	2,200.09	94%
2005	SM0118000	PDS BRASÍLIA	192.89	4.82	3%	188.07	97%	188.07	97%	71.35	38%	5%	116.72	61%
2005	SM0123000	PDS MALOCA	312.28	93.08	30%	219.21	70%	219.21	70%	19.97	9%	1%	199.24	64%
2005	SM0125000	PDS VALE DO JAMANXIM	882.37	69.34	8%	813.03	92%	813.03	92%	48.95	6%	1%	764.08	87%
2005	SM0126000	PDS ÁGUA PRETA	248.40	3.06	1%	245.34	99%	245.34	99%	16.36	7%	1%	228.99	92%
2005	SM0127000	PDS CASTANHEIRA	258.15	0.64	0%	257.51	100%	257.51	100%	13.11	5%	1%	244.40	95%

2005	SM0144000	PDS RENASCER II	373.87	1.08	0%	372.79	100%	372.79	100%	1.95	1%	0%	370.85	99%
2005	SM0146000	PDS PARAÍSO	1246.55	15.96	1%	1,230.59	99%	1,230.59	99%	19.65	2%	0%	1,210.94	97%
2005	SM0149000	PDS LIBERDADE	2437.63	374.61	15%	2,063.02	85%	2,063.02	85%	270.87	13%	2%	1,792.15	74%
2006	AC0127000	PDS IVO NEVES	40.57	5.85	14%	34.72	86%	34.72	86%	10.23	29%	4%	24.49	60%
2006	AC0128000	PDS WILSON LOPES	168.62	57.01	34%	111.60	66%	111.60	66%	29.47	26%	4%	82.13	49%
2006	AC0129000	PA LUZ DA VIDA	3.79	3.07	81%	0.72	19%	0.72	19%	0.12	16%	2%	0.61	16%
2006	AC0130000	PDS POLO RECANTO DO EQUADOR	19.24	6.33	33%	12.91	67%	12.91	67%	4.11	32%	5%	8.81	46%
2006	AC0131000	PDS GERALDO FERNANDES	19.50	0.68	3%	18.82	97%	18.82	97%	5.86	31%	4%	12.96	66%
2006	AC0132000	PDS POLO PIRÃ-DE-RÃ	13.66	10.82	79%	2.84	21%	2.84	21%	0.03	1%	0%	2.82	21%
2006	AC0136000	PDS LÍDIA CRAVEIRO	8.05	0.11	1%	7.94	99%	7.94	99%	2.64	33%	5%	5.30	66%
2006	AC0137000	PA URUBURETAMA	22.59	0.98	4%	21.61	96%	21.61	96%	0.14	1%	0%	21.47	95%
2006	AC0138000	PDS SETE LAGOAS	24.04	2.98	12%	21.06	88%	21.06	88%	0.77	4%	1%	20.29	84%
2006	AC0139000	PA CALIFÓRNIA	56.58	21.04	37%	35.54	63%	35.54	63%	4.34	12%	2%	31.21	55%
2006	AM0081000	PAE CABALIANA I	622.45	199.86	32%	422.58	68%	422.58	68%	6.71	2%	0%	415.87	67%
2006	AM0083000	PAE PURUS	6488.12	114.70	2%	6,373.42	98%	6,373.42	98%	17.30	0%	0%	6,356.11	98%
2006	AM0085000	PDS NOVA ESPERANÇA	3.12	2.76	88%	0.36	12%	0.36	12%	0.17	47%	7%	0.19	6%
2006	AM0087000	PAE MARIPITI	1175.71	24.71	2%	1,151.00	98%	1,151.00	98%	6.75	1%	0%	1,144.24	97%
2006	AM0094000	PAE ONÇAS	39.86	0.03	0%	39.82	100%	39.82	100%	0.90	2%	0%	38.92	98%
2006	AM0095000	PAE CABALIANA II	960.91	23.44	2%	937.47	98%	937.47	98%	15.95	2%	0%	921.52	96%
2006	AM0098000	PAE BELA VISTA II	283.43	146.2	52%	137.21	48%	137.21	48%	1.90	1%	0%	135.30	48%

2

2006	MA0136000	PA QUADRA RENASCER II/COLONE	1.52	1.52	100%	0.00	0%	0.00	0%	0.00		0%	0.00	0%
2006	MA0153000	PA OLHO D ÁGUA DO TURI/COLONE	10.20	10.20	100%	0.00	0%	0.00	0%	0.00		0%	0.00	0%
2006	MA0835000	PA TAMBOR CENTRO VELHO	32.78	32.63	100%	0.15	0%	0.15	0%	0.06	41%	6%	0.09	0%
2006	MA0954000	PA MAPISA	91.85	63.19	69%	28.66	31%	28.66	31%	6.94	24%	3%	21.72	24%
2006	MA0962000	PE TATAJUBA	54.77	30.67	56%	24.09	44%	24.09	44%	0.90	4%	1%	23.19	42%
2006	MA0965000	PA JIQUIRI / SANTO AGOSTINHO	11.52	10.79	94%	0.73	6%	0.73	6%	0.30	42%	6%	0.43	4%
2006	MA0998000	PA SÃO BARTOLOMEU / CIAMA	32.35	24.66	76%	7.70	24%	7.70	24%	2.87	37%	5%	4.82	15%
2006	MA0999000	PA TORRÃO MUQUEM	4.89	4.89	100%	0.00	0%	0.00	0%	0.00		0%	0.00	0%
2006	MA1002000	PA FÊNIX	93.48	48.44	52%	45.04	48%	45.04	48%	22.86	51%	7%	22.18	24%
2006	MA1003000	PA TERRA LIVRE	33.23	8.31	25%	24.92	75%	24.92	75%	4.14	17%	2%	20.78	63%
2006	MA1025000	PA CATINGUEIRO	6.70	6.70	100%	0.00	0%	0.00	0%	0.00		0%	0.00	0%
2006	MB0474000	PA CLÉZINHO	46.65	25.30	54%	21.35	46%	21.35	46%	8.84	41%	6%	12.51	27%
2006	MB0475000	PA NOSSA SENHORA APARECIDA II	2.12	0.95	45%	1.17	55%	1.17	55%	0.00	0%	0%	1.17	55%
2006	MB0476000	PA TIRADENTES	2.73	1.47	54%	1.26	46%	1.26	46%	0.52	42%	6%	0.74	27%
2006	MB0477000	PA ARARANDEUA	273.91	89.13	33%	184.77	67%	184.77	67%	42.25	23%	3%	142.53	52%
2006	MB0478000	PA IRMÃ DOROTY	30.93	0.39	1%	30.54	99%	30.54	99%	3.22	11%	2%	27.32	88%
2006	MB0480000	PA COSME E DAMIÃO	15.47	12.40	80%	3.07	20%	3.07	20%	1.95	64%	9%	1.12	7%

2006	MB0481000	PA SANTA CECILIA	9.34	1.99	21%	7.34	79%	7.34	79%	2.27	31%	4%	5.08	54%
2006	MB0483000	PA CENTRO NOVO II	36.14	35.04	97%	1.10	3%	1.10	3%	0.10	9%	1%	1.00	3%
2006	MB0484000	PA SANTA ELIZA	33.46	33.45	100%	0.01	0%	0.01	0%	0.00	0%	0%	0.01	0%
2006	MB0485000	PA MORADA NOVA	148.12	73.87	50%	74.26	50%	74.26	50%	20.35	27%	4%	53.91	36%
2006	MB0486000	PA MORADA NOVA II	114.47	39.03	34%	75.44	66%	75.44	66%	21.04	28%	4%	54.40	48%
2006	MB0489000	PA DEUS É PAZ	77.70	68.79	89%	8.91	11%	8.91	11%	0.42	5%	1%	8.49	11%
2006	MB0491000	PA POMBAL	862.49	176.31	20%	686.18	80%	686.18	80%	192.62	28%	4%	493.56	57%
2006	MB0492000	PA FELICIDADE	52.86	47.65	90%	5.20	10%	5.20	10%	2.38	46%	7%	2.82	5%
2006	MB0493000	PA 1° DE MAIO	28.71	19.51	68%	9.19	32%	9.19	32%	4.29	47%	7%	4.90	17%
2006	MT0758000	PA MARUMBI	39.12	24.28	62%	14.84	38%	14.84	38%	1.76	12%	2%	13.08	33%
2006	MT0760000	PA PINHEIRO VELHO	30.13	15.89	53%	14.24	47%	14.24	47%	4.80	34%	5%	9.44	31%
2006	MT0764000	PA SÃO JUDAS	68.21	54.28	80%	13.94	20%	13.94	20%	3.49	25%	4%	10.44	15%
2006	MT0789000	PA BOA ESPERANÇA I	3.40	2.73	80%	0.67	20%	0.67	20%	0.30	46%	7%	0.36	11%
2006	MT0793000	PA CANÃA I	37.64	28.78	76%	8.86	24%	8.86	24%	2.06	23%	3%	6.80	18%
2006	MT0794000	PA SÃO PEDRO/CAMBARA	41.25	17.79	43%	23.47	57%	23.47	57%	4.33	18%	3%	19.14	46%
2006	PA0306000	PA PAULO FONTELES	8.45	2.84	34%	5.61	66%	5.61	66%	0.14	3%	0%	5.47	65%
2006	PA0307000	PA ALTO BONITO	24.27	16.25	67%	8.03	33%	8.03	33%	4.10	51%	7%	3.93	16%
2006	PA0308000	PA SANTA PAULA I	3.52	3.52	100%	0.00	0%	0.00	0%	0.00		0%	0.00	0%
2006	PA0309000	PAE ILHA MAMANGAL	24.85	0.48	2%	24.37	98%	24.37	98%	0.14	1%	0%	24.23	98%
2006	PA0310000	PAE ILHA URUBUOCA	3.12	0.00	0%	3.12	100%	3.12	100%	0.00	0%	0%	3.12	100%
2006	PA0311000	PAE ILHA LONGA	1.59	0.00	0%	1.59	100%	1.59	100%	0.00	0%	0%	1.59	100%
2006	PA0312000	PAE ILHA SUMAUMA	42.59	1.86	4%	40.73	96%	40.73	96%	0.13	0%	0%	40.60	95%

2006	PA0313000	PAE ILHA GRANDE - PACAJAI	345.06	67.71	20%	277.34	80%	277.34	80%	8.98	3%	0%	268.36	78%
2006	PA0314000	PAE ILHA BUÇU	3.49	0.07	2%	3.42	98%	3.42	98%	0.11	3%	0%	3.31	95%
2006	PA0315000	PAE ILHA MUCURA	3.94	0.00	0%	3.94	100%	3.94	100%	0.00	0%	0%	3.94	100%
2006	PA0316000	PAE ILHA ARAPARI	27.56	0.41	1%	27.15	99%	27.15	99%	0.00	0%	0%	27.15	99%
2006	PA0317000	PAE ILHA MOCAJUBA	1.37	0.81	59%	0.55	41%	0.55	41%	0.00	0%	0%	0.55	41%
2006	PA0318000	PA NOVO JAUARA	14.32	12.25	86%	2.07	14%	2.07	14%	0.00	0%	0%	2.07	14%
2006	PA0319000	PA ARAXITEUA	14.66	11.17	76%	3.49	24%	3.49	24%	0.55	16%	2%	2.94	20%
2006	PA0320000	PA FLOR DE MINAS	26.03	21.98	84%	4.06	16%	4.06	16%	1.26	31%	4%	2.80	11%
2006	PA0321000	PAE ILHA SÃO MATEUS	16.85	0.24	1%	16.61	99%	16.61	99%	0.03	0%	0%	16.58	98%
2006	PA0322000	PAE ILHA GRANDE CAMETÁ	66.94	0.02	0%	66.92	100%	66.92	100%	0.00	0%	0%	66.92	100%
2006	PA0323000	PAE NOSSA SENHORA DA PAZ	21.63	8.23	38%	13.40	62%	13.40	62%	0.24	2%	0%	13.16	61%
2006	PA0324000	PAE SÃO JOÃO BATISTA II	31.80	7.06	22%	24.74	78%	24.74	78%	0.43	2%	0%	24.31	76%
2006	PA0325000	PAE ILHA UMARITUBA	7.08	0.00	0%	7.08	100%	7.08	100%	0.00	0%	0%	7.08	100%
2006	PA0326000	PAE ILHA DO PAULO	3.46	0.00	0%	3.46	100%	3.46	100%	0.00	0%	0%	3.46	100%
2006	PA0327000	PAE ILHA DO TESO	10.20	0.00	0%	10.20	100%	10.20	100%	0.00	0%	0%	10.20	100%
2006	PA0328000	PAE ILHA RAQUEL	15.90	0.13	1%	15.77	99%	15.77	99%	0.00	0%	0%	15.77	99%
2006	PA0329000	PAE ILHA CAETÉ	11.33	0.00	0%	11.33	100%	11.33	100%	0.00	0%	0%	11.33	100%
2006	PA0330000	PAE ILHA CUXIPIARI	34.98	0.00	0%	34.98	100%	34.98	100%	0.00	0%	0%	34.98	100%
2006	PA0331000	PAE ILHA JACARÉ XINGU	8.61	0.00	0%	8.61	100%	8.61	100%	0.00	0%	0%	8.61	100%

2006	PA0332000	PAE ILHA JAITUBA	6.73	0.00	0%	6.73	100%	6.73	100%	0.00	0%	0%	6.73	100%
2006	PA0333000	PAE ILHA JOROCA	19.38	0.05	0%	19.34	100%	19.34	100%	0.00	0%	0%	19.34	100%
2006	PA0334000	PAE ILHA MANOEL RAIMUNDO	10.66	0.00	0%	10.66	100%	10.66	100%	0.00	0%	0%	10.66	100%
2006	PA0337000	PAE ILHA GAMA	0.89	0.00	0%	0.89	100%	0.89	100%	0.00	0%	0%	0.89	100%
2006	PA0338000	PAE ILHA GUAJARÁ	3.93	0.00	0%	3.93	100%	3.93	100%	0.00	0%	0%	3.93	100%
2006	PA0339000	PAE ILHA CACOAL	3.31	0.00	0%	3.31	100%	3.31	100%	0.00	0%	0%	3.31	100%
2006	PA0340000	PAE ILHA JARACUERA	5.73	0.00	0%	5.73	100%	5.73	100%	0.00	0%	0%	5.73	100%
2006	PA0341000	PAE ILHA MAPEUÁ	3.55	0.00	0%	3.55	100%	3.55	100%	0.00	0%	0%	3.55	100%
2006	PA0342000	PAE ILHA MOIRABA	2.30	0.00	0%	2.30	100%	2.30	100%	0.00	0%	0%	2.30	100%
2006	PA0343000	PAE ILHA TABATINGA DO CARAPAJÓ	4.27	0.00	0%	4.27	100%	4.27	100%	0.00	0%	0%	4.27	100%
2006	PA0344000	PAE ILHA PARURU	37.24	0.00	0%	37.24	100%	37.24	100%	0.00	0%	0%	37.24	100%
2006	PA0345000	PAE ILHA CARIPETUBA	36.71	10.15	28%	26.56	72%	26.56	72%	0.46	2%	0%	26.10	71%
2006	PA0346000	PAE ILHA URUÁ I	15.79	0.53	3%	15.26	97%	15.26	97%	0.10	1%	0%	15.16	96%
2006	PA0347000	PAE ILHA PIQUIARANA	7.69	1.45	19%	6.23	81%	6.23	81%	0.00	0%	0%	6.23	81%
2006	PA0348000	PAE ILHA XIPAIÁ	12.12	1.96	16%	10.16	84%	10.16	84%	0.00	0%	0%	10.16	84%
2006	PA0349000	PAE ILHA GRANDE BELÉM	7.46	0.00	0%	7.46	100%	7.46	100%	0.00	0%	0%	7.46	100%
2006	PA0350000	PAE ILHA JUTUBA	4.53	0.00	0%	4.53	100%	4.53	100%	0.00	0%	0%	4.53	100%
2006	PA0351000	PAE ILHA MURUTUCU	8.27	0.00	0%	8.27	100%	8.27	100%	0.00	0%	0%	8.27	100%
2006	PA0352000	PAE ILHA PAQUETÁ	7.21	0.00	0%	7.21	100%	7.21	100%	0.00	0%	0%	7.21	100%

2006	PA0353000	PAE ILHA SANTANA	45.13	10.30	23%	34.83	77%	34.83	77%	0.10	0%	0%	34.73	77%
2006	PA0354000	PAE ILHA JUPATITUBA	0.22	0.00	0%	0.22	100%	0.22	100%	0.00	0%	0%	0.22	100%
2006	PA0355000	PAE ILHA URUÁ	4.33	0.00	0%	4.33	100%	4.33	100%	0.00	0%	0%	4.33	100%
2006	PA0356000	PAE ILHA SANTARÉM	41.39	0.00	0%	41.39	100%	41.39	100%	0.00	0%	0%	41.39	100%
2006	PA0357000	PAE ILHA CASTANHAL	12.33	0.00	0%	12.33	100%	12.33	100%	0.00	0%	0%	12.33	100%
2006	PA0358000	PAE ILHA UNIÃO	6.15	0.00	0%	6.15	100%	6.15	100%	0.00	0%	0%	6.15	100%
2006	PA0359000	PAE ILHA DAS CINZAS	29.18	0.00	0%	29.18	100%	29.18	100%	0.00	0%	0%	29.18	100%
2006	PA0360000	PAE ILHA PALHETA	3.87	0.13	3%	3.73	97%	3.73	97%	0.00	0%	0%	3.73	97%
2006	PA0361000	PAE ILHA DAS PRACUUBINHAS	8.07	0.03	0%	8.04	100%	8.04	100%	0.00	0%	0%	8.04	100%
2006	PA0362000	PAE ILHA CHIQUEIRO	24.70	0.00	0%	24.70	100%	24.70	100%	0.00	0%	0%	24.70	100%
2006	PA0363000	PAE ILHA CAMPUMPEMA	2.04	0.00	0%	2.04	100%	2.04	100%	0.00	0%	0%	2.04	100%
2006	PA0364000	PAE ILHA URUTAÍ PARTE	105.32	0.00	0%	105.32	100%	105.32	100%	0.00	0%	0%	105.32	100%
2006	PA0365000	PAE ILHA MURUMURU	14.34	0.00	0%	14.34	100%	14.34	100%	0.00	0%	0%	14.34	100%
2006	PA0366000	PAE ILHA GOIABAL	11.01	0.00	0%	11.01	100%	11.01	100%	0.00	0%	0%	11.01	100%
2006	PA0367000	PAE ILHA BACURI	20.51	0.00	0%	20.51	100%	20.51	100%	0.00	0%	0%	20.51	100%
2006	PA0368000	PAE ILHA SANTO AMARO	40.86	0.00	0%	40.86	100%	40.86	100%	0.00	0%	0%	40.86	100%
2006	PA0369000	PAE ILHA	5.45	0.46	9%	4.99	91%	4.99	91%	0.10	2%	0%	4.89	90%

		PIQUIARANA MIRIM												
2006	PA0370000	PA PALHETA	66.38	59.58	90%	6.79	10%	6.79	10%	1.00	15%	2%	5.79	9%
2006	PA0371000	PAE ILHA SACAJÓS GRANDE	32.23	0.00	0%	32.23	100%	32.23	100%	0.00	0%	0%	32.23	100%
2006	PA0372000	PAE ILHA PRAIA GRANDE	44.16	0.65	1%	43.52	99%	43.52	99%	0.00	0%	0%	43.51	99%
2006	PA0373000	PAE ILHA MURUJUCÁ MIRI	21.15	0.00	0%	21.15	100%	21.15	100%	0.06	0%	0%	21.09	100%
2006	PA0374000	PAE ILHA ATURIÁ GRANDE	4.05	0.00	0%	4.05	100%	4.05	100%	0.00	0%	0%	4.05	100%
2006	PA0375000	PAE ILHA DAMIÃO	38.11	0.00	0%	38.11	100%	38.11	100%	0.00	0%	0%	38.11	100%
2006	PA0376000	PAE ILHA CAÍ GRANDE	5.58	0.00	0%	5.58	100%	5.58	100%	0.00	0%	0%	5.58	100%
2006	PA0377000	PAE ILHA JARIMBU	83.48	0.41	0%	83.07	100%	83.07	100%	1.05	1%	0%	82.02	98%
2006	PA0378000	PAE ILHA PANACAUERA-MIRI	39.42	1.19	3%	38.23	97%	38.23	97%	0.29	1%	0%	37.94	96%
2006	PA0379000	PAE ILHA DO CUMBU	14.17	0.00	0%	14.17	100%	14.17	100%	0.04	0%	0%	14.13	100%
2006	PA0380000	PAE ILHA PAULISTA	15.65	0.01	0%	15.63	100%	15.63	100%	0.00	0%	0%	15.63	100%
2006	PA0381000	PAE ILHA CONCEIÇÃO	47.12	0.00	0%	47.12	100%	47.12	100%	0.00	0%	0%	47.12	100%
2006	PA0382000	PAE ILHA MUTIRÃO	16.63	1.73	10%	14.90	90%	14.90	90%	0.44	3%	0%	14.46	87%
2006	PA0383000	PA AREIA BRANCA	14.25	11.56	81%	2.69	19%	2.69	19%	0.98	36%	5%	1.71	12%
2006	PA0384000	PA LUIZ INÁCIO	349.43	130.11	37%	219.32	63%	219.32	63%	75.79	35%	5%	143.53	41%
2006	PA0385000	PAE ILHA COMPRIDA	0.15	0.00	0%	0.15	100%	0.15	100%	0.00	0%	0%	0.15	100%
2006	PA0386000	PAE ILHA MAUBA	8.34	0.00	0%	8.34	100%	8.34	100%	0.12	1%	0%	8.22	99%
2006	PA0389000	PAE ILHA ANUERÁ	2.14	0.04	2%	2.11	98%	2.11	98%	0.00	0%	0%	2.11	98%

2006	PA0390000	PAE ILHA DE MELGAÇO	117.79	13.02	11%	104.76	89%	104.76	89%	0.36	0%	0%	104.40	89%
2006	PA0391000	PAE ILHA MUJIRUM	151.71	1.49	1%	150.22	99%	150.22	99%	0.38	0%	0%	149.84	99%
2006	PA0394000	PAE TRÊS IRMÃS	5.18	0.00	0%	5.18	100%	5.18	100%	0.00	0%	0%	5.18	100%
2006	PA0395000	PAE ILHA COROA NOVA	6.71	0.00	0%	6.71	100%	6.71	100%	0.00	0%	0%	6.71	100%
2006	PA0396000	PAE ILHA MARINTEUA	3.47	0.03	1%	3.43	99%	3.43	99%	0.00	0%	0%	3.43	99%
2006	PA0397000	PAE ILHA PACUÍ	7.60	0.00	0%	7.60	100%	7.60	100%	0.00	0%	0%	7.60	100%
2006	PA0398000	PAE ILHA ITANDUBA	7.81	0.00	0%	7.81	100%	7.81	100%	0.00	0%	0%	7.81	100%
2006	PA0399000	PAE ILHA ITAUNA	7.55	0.00	0%	7.55	100%	7.55	100%	0.00	0%	0%	7.55	100%
2006	PA0400000	PAE ILHA AJARAÍ	3.45	0.00	0%	3.45	100%	3.45	100%	0.00	0%	0%	3.45	100%
2006	PA0401000	PAE ILHA SANTANA DE CAMETÁ	25.47	0.00	0%	25.47	100%	25.47	100%	0.00	0%	0%	25.47	100%
2006	PA0402000	PA NOVA VIDA	40.69	8.63	21%	32.06	79%	32.06	79%	11.36	35%	5%	20.70	51%
2006	PA0403000	PA GLEBINHA	17.60	3.90	22%	13.70	78%	13.70	78%	3.19	23%	3%	10.51	60%
2006	PA0405000	PAE ILHA JUPATITUBA DE CURRALINHO	106.31	6.50	6%	99.81	94%	99.81	94%	0.16	0%	0%	99.65	94%
2006	PA0406000	PAE ILHA CANATICU	11.23	0.00	0%	11.23	100%	11.23	100%	0.00	0%	0%	11.23	100%
2006	PA0407000	PAE ILHA DO MUTUM	42.72	0.05	0%	42.67	100%	42.67	100%	0.00	0%	0%	42.67	100%
2006	PA0408000	PAE ILHA DO FURO GRANDE	13.18	0.06	0%	13.11	100%	13.11	100%	0.00	0%	0%	13.11	100%
2006	PA0409000	PAE ILHA ITABOCA	22.33	0.08	0%	22.25	100%	22.25	100%	0.00	0%	0%	22.25	100%
2006	PA0410000	PAE ILHA PINDOBAL GRANDE	37.31	0.05	0%	37.26	100%	37.26	100%	0.38	1%	0%	36.89	99%

2006	PA0411000	PA JUTAÍ MIRIM	21.92	16.99	77%	4.93	23%	4.93	23%	0.36	7%	1%	4.58	21%
2006	PA0412000	PAE ILHA COROCA	18.53	0.00	0%	18.53	100%	18.53	100%	0.00	0%	0%	18.53	100%
2006	PA0413000	PAE ILHA PAQUETÁ II	6.76	0.00	0%	6.76	100%	6.76	100%	0.00	0%	0%	6.76	100%
2006	PA0414000	PAE ILHA BOA VISTA	9.24	0.13	1%	9.12	99%	9.12	99%	0.00	0%	0%	9.11	99%
2006	PA0415000	PAE ILHA CHAVES	8.82	0.00	0%	8.82	100%	8.82	100%	0.00	0%	0%	8.82	100%
2006	PA0416000	PAE ILHA LARANJA	15.37	0.13	1%	15.24	99%	15.24	99%	0.08	1%	0%	15.15	99%
2006	PA0417000	PAE ILHA SÃO JOÃO	7.86	0.00	0%	7.86	100%	7.86	100%	0.00	0%	0%	7.86	100%
2006	PA0418000	PAE ILHA TUCUPI GRANDE	14.29	0.00	0%	14.29	100%	14.29	100%	0.00	0%	0%	14.29	100%
2006	PA0419000	PAE ILHA SANTO ANTÔNIO III	12.77	0.00	0%	12.77	100%	12.77	100%	0.00	0%	0%	12.77	100%
2006	RO0161000	PA PAULO FREIRE	18.65	7.32	39%	11.33	61%	11.33	61%	5.56	49%	7%	5.77	31%
2006	RO0162000	PA ANTONIO CONSELHEIRO	9.57	1.55	16%	8.03	84%	8.03	84%	3.57	44%	6%	4.46	47%
2006	RO0164000	PDS ROLIM DE MOURA DO GUAPORÉ	18.30	6.71	37%	11.59	63%	11.59	63%	1.55	13%	2%	10.04	55%
2006	RO0165000	PA BOA ESPERANÇA	3.17	2.52	80%	0.64	20%	0.64	20%	0.32	49%	7%	0.33	10%
2006	RO0166000	PA RIO SÃO PEDRO	19.08	11.39	60%	7.68	40%	7.68	40%	1.22	16%	2%	6.47	34%
2006	RR0049000	PA RENASCER	4.51	0.71	16%	3.80	84%	3.80	84%	0.09	2%	0%	3.71	82%
2006	RR0051000	PA PAU- BRASIL	251.41	0.42	0%	250.99	100%	250.99	100%	0.27	0%	0%	250.72	100%
2006	RR0052000	PA TERRA NOVA	34.35	8.61	25%	25.74	75%	25.74	75%	4.05	16%	2%	21.70	63%
2006	RR0053000	PA SERRA TALHADA	32.49	1.95	6%	30.54	94%	30.54	94%	5.03	16%	2%	25.51	79%
2006	RR0054000	PA ARCO-ÍRIS	146.04	0.11	0%	145.94	100%	145.94	100%	3.38	2%	0%	142.56	98%
2006	SM0150000	PDS NELSON DE OLIVEIRA	45.34	3.18	7%	42.16	93%	42.16	93%	5.72	14%	2%	36.44	80%

2006	SM0151000	PDS TERRA NOSSA	1473.33	195.22	13%	1,278.11	87%	1,278.11	87%	205.42	16%	2%	1,072.69	73%
2006	SM0153000	PAE REGIÃO DO CUÇARI	287.24	70.18	24%	217.05	76%	217.05	76%	26.91	12%	2%	190.15	66%
2006	SM0154000	PDS MÁRIO BRAULE PINTO DA SILVA	70.14	0.00	0%	70.14	100%	70.14	100%	0.00	0%	0%	70.14	100%
2006	SM0155000	PDS ESPERANÇA DO TRAIRÃO	247.95	44.43	18%	203.52	82%	203.52	82%	16.57	8%	1%	186.95	75%
2006	SM0156000	PDS NOVA UNIÃO	116.44	74.79	64%	41.65	36%	41.65	36%	3.40	8%	1%	38.25	33%
2006	SM0157000	PA ARIXI	57.56	9.62	17%	47.94	83%	47.94	83%	1.43	3%	0%	46.51	81%
2006	SM0162000	PAE CUÇARU	7.93	4.21	53%	3.72	47%	3.72	47%	0.26	7%	1%	3.46	44%
2006	SM0165000	PAE NAZARÉ	14.85	9.92	67%	4.93	33%	4.93	33%	0.59	12%	2%	4.34	29%
2006	SM0167000	PAE IGARAPE DO CUÇARI	0.08	0.00	0%	0.08	100%	0.08	100%	0.00	0%	0%	0.08	100%
2006	SM0169000	PAE COSTA DO AMAZONAS	0.55	0.29	52%	0.26	48%	0.26	48%	0.13	50%	7%	0.13	24%
2006	SM0170000	PAE ARITAPERA	0.04	0.00	0%	0.04	100%	0.04	100%	0.00	0%	0%	0.04	100%
2006	SM0171000	PA ANAPUZINHO	181.39	71.21	39%	110.18	61%	110.18	61%	17.69	16%	2%	92.49	51%
2006	SM0172000	PDS SANTA CLARA	241.77	4.75	2%	237.02	98%	237.02	98%	3.08	1%	0%	233.94	97%
2006	SM0174000	PAE SÃO DIOGO	0.82	0.82	100%	0.00	0%	0.00	0%	0.00		0%	0.00	0%
2006	SM0175000	PDS LIBERDADE I	4513.92	87.13	2%	4,426.79	98%	4,426.79	98%	69.37	2%	0%	4,357.43	97%
2006	SM0177000	PDS HORIZONTE NOVO	216.41	5.04	2%	211.38	98%	211.38	98%	9.46	4%	1%	201.92	93%
2006	SM0180000	PAE ATUMÃ	0.06	0.00	0%	0.06	100%	0.06	100%	0.00	0%	0%	0.06	100%
2006	SM0181000	PA MACANÃ I	261.02	10.10	4%	250.92	96%	250.92	96%	24.75	10%	1%	226.18	87%
2006	SM0182000	PA MACANÃ II	314.12	9.77	3%	304.35	97%	304.35	97%	18.40	6%	1%	285.95	91%
2006	SM0183000	PDS ITATA	1052.85	0.63	0%	1,052.22	100%	1,052.22	100%	56.70	5%	1%	995.53	95%

2006	SM0186000	PAE TAPARÁ	10.25	2.60	25%	7.65	75%	7.65	75%	0.18	2%	0%	7.47	73%
2006	SM0187000	PAC ARAIPACUPU	302.35	161.07	53%	141.28	47%	141.28	47%	10.08	7%	1%	131.20	43%
2006	SM0188000	PDS NOVA BRASILIA II	56.96	9.75	17%	47.22	83%	47.22	83%	3.12	7%	1%	44.09	77%
2006	SM0189000	PA NOSSA SENHORA DE FATIMA	42.40	4.85	11%	37.55	89%	37.55	89%	5.84	16%	2%	31.71	75%
2006	SM0190000	PA RIO CIGANO	22.00	5.23	24%	16.78	76%	16.78	76%	2.71	16%	2%	14.06	64%
2006	SM0191000	PDS ÁGUA AZUL	296.93	47.70	16%	249.23	84%	249.23	84%	20.08	8%	1%	229.15	77%
2006	SM0192000	PDS BOA VISTA DO CARACOL	227.54	15.43	7%	212.11	93%	212.11	93%	16.52	8%	1%	195.59	86%
2006	SM0193000	PAC NOVA ALTAMIRA	18.30	14.98	82%	3.32	18%	3.32	18%	0.00	0%	0%	3.32	18%
2006	SM0194000	PAC CAUÃU B E BALANÇA	24.39	17.03	70%	7.36	30%	7.36	30%	0.31	4%	1%	7.05	29%
2006	SM0197000	PAE MARIA TEREZA	1.65	0.27	16%	1.38	84%	1.38	84%	0.00	0%	0%	1.38	84%
2006	SM0203000	PAE PAITUNA	1.51	1.08	72%	0.43	28%	0.43	28%	0.00	0%	0%	0.43	28%
2006	SM0204000	PAE REGIAO DOS LAGOS	7.22	4.81	67%	2.42	33%	2.42	33%	0.31	13%	2%	2.10	29%
2006	SM0206000	PAC BELA TERRA I	108.55	55.99	52%	52.56	48%	52.56	48%	5.15	10%	1%	47.41	44%
2006	SM0207000	PAC BELA TERRA II	109.94	81.12	74%	28.81	26%	28.81	26%	1.89	7%	1%	26.92	24%
2006	SM0208000	PDS ARTHUR FALEIRO	227.10	9.24	4%	217.86	96%	217.86	96%	10.22	5%	1%	207.64	91%
2006	SM0209000	PDS AVELINO RIBEIRO	216.62	6.32	3%	210.29	97%	210.29	97%	6.46	3%	0%	203.83	94%
2006	SM0213000	PAE SALÉ	13.64	9.99	73%	3.65	27%	3.65	27%	0.15	4%	1%	3.50	26%
2006	SM0214000	PAE BALAIO	3.83	3.32	87%	0.50	13%	0.50	13%	0.10	20%	3%	0.40	11%

2006	SM0215000	PDS MÃE MENININHA	188.28	14.15	8%	174.13	92%	174.13	92%	24.09	14%	2%	150.04	80%
2006	SM0216000	PDS ESPERANÇA	180.10	12.16	7%	167.94	93%	167.94	93%	8.05	5%	1%	159.89	89%
2006	SM0217000	PA ESPERANÇA	144.74	57.82	40%	86.93	60%	86.93	60%	22.84	26%	4%	64.09	44%
2006	SM0218000	PAC ANANIZAL	24.48	9.36	38%	15.12	62%	15.12	62%	0.91	6%	1%	14.22	58%
2006	SM0219000	PAC ITAPECURU	47.22	15.87	34%	31.35	66%	31.35	66%	2.62	8%	1%	28.73	61%
2006	SM0220000	PAC IRIPIXI	24.33	13.60	56%	10.72	44%	10.72	44%	0.19	2%	0%	10.54	43%
2006	SM0221000	PAC MONTE MURIA	48.53	13.95	29%	34.58	71%	34.58	71%	6.92	20%	3%	27.66	57%
2006	SM0222000	PAE ARAMANAÍ	33.32	17.17	52%	16.15	48%	16.15	48%	1.68	10%	1%	14.47	43%
2006	SM0223000	PAE PINDOBAL	80.47	16.25	20%	64.22	80%	64.22	80%	0.14	0%	0%	64.08	80%
2006	SM0225000	PAE CACHOERY	0.93	0.06	7%	0.87	93%	0.87	93%	0.00	0%	0%	0.87	93%
2006	SM0226000	PAE ITUQUI	43.27	28.02	65%	15.24	35%	15.24	35%	1.91	13%	2%	13.34	31%
2006	SM0227000	PAE CHICANTÃ	1.96	0.54	27%	1.42	73%	1.42	73%	0.17	12%	2%	1.25	64%
2006	SM0230000	PAE JACARECAPÁ	11.85	9.23	78%	2.62	22%	2.62	22%	0.22	8%	1%	2.40	20%
2006	SM0231000	PAE JAQUARÁ	18.24	15.15	83%	3.09	17%	3.09	17%	0.18	6%	1%	2.91	16%
2006	SM0232000	PAE ALDEIA	26.42	22.58	85%	3.84	15%	3.84	15%	0.72	19%	3%	3.13	12%
2006	SM0233000	PAC BOM SOSSEGO	868.60	247.77	29%	620.83	71%	620.83	71%	32.61	5%	1%	588.22	68%
2006	SM0234000	PA PARAISO DO NORTE	1129.70	106.74	9%	1,022.95	91%	1,022.95	91%	47.08	5%	1%	975.87	86%
2006	SM0235000	PAC SÃO SEBASTIÃO DO TUTUÍ	398.27	5.00	1%	393.27	99%	393.27	99%	12.63	3%	0%	380.64	96%
2006	SM0236000	PAC NOVA UNIÃO	316.02	22.73	7%	293.29	93%	293.29	93%	15.24	5%	1%	278.05	88%
2006	SM0237000	PDS OURO BRANCO	545.77	42.04	8%	503.72	92%	503.72	92%	17.69	4%	1%	486.03	89%
2006	SM0238000	PAC OURO BRANCO I	398.07	21.90	6%	376.17	94%	376.17	94%	17.65	5%	1%	358.52	90%
2006	SM0239000	PAC OURO BRANCO II	369.93	10.35	3%	359.58	97%	359.58	97%	14.99	4%	1%	344.59	93%

2006	SM0241000	PDS LARANJAL	340.48	10.80	3%	329.68	97%	329.68	97%	12.90	4%	1%	316.78	93%
2006	SM0242000	PDS PIMENTAL	62.27	12.47	20%	49.80	80%	49.80	80%	3.09	6%	1%	46.72	75%
2006	SM0246000	PDS IRMÃ DOROTY	305.08	17.63	6%	287.45	94%	287.45	94%	8.06	3%	0%	279.39	92%
2006	TO0386000	PA SANTA HELENA II	14.53	11.45	79%	3.08	21%	3.08	21%	0.07	2%	0%	3.02	21%
2007	AC0141000	PDS FLORESTA	63.90	27.38	43%	36.53	57%	36.53	57%	3.67	10%	2%	32.86	51%
2007	AM0086000	PDS GEDEÃO	113.26	4.54	4%	108.73	96%	108.73	96%	12.18	11%	2%	96.55	85%
2007	AM0089000	PAE URUAPIARA	397.45	15.04	4%	382.41	96%	382.41	96%	1.09	0%	0%	381.33	96%
2007	AM0090000	PAE SANTA FÉ	38.34	0.84	2%	37.51	98%	37.51	98%	0.07	0%	0%	37.44	98%
2007	AM0091000	PAE SÃO JOAQUIM	1484.28	13.45	1%	1,470.83	99%	1,470.83	99%	0.51	0%	0%	1,470.32	99%
2007	AM0092000	PAE FLORESTA DO IPIXUNA	290.50	2.16	1%	288.34	99%	288.34	99%	0.21	0%	0%	288.13	99%
2007	AM0093000	PAE TUPANA IGAPÓ AÇU II	4483.25	5.50	0%	4,477.74	100%	4,477.74	100%	0.20	0%	0%	4,477.55	100%
2007	AM0099000	PAE NOVO ORIENTE	185.25	0.90	0%	184.35	100%	184.35	100%	9.74	5%	1%	174.61	94%
2007	AM0101000	PAE RIO AÇUÃ	131.94	4.58	3%	127.36	97%	127.36	97%	4.95	4%	1%	122.42	93%
2007	AM0103000	PDS REALIDADE	434.67	3.11	1%	431.57	99%	431.57	99%	2.97	1%	0%	428.60	99%
2007	AM0106000	PAE SANTA MARIA AUXILIADORA	347.44	3.17	1%	344.27	99%	344.27	99%	0.43	0%	0%	343.84	99%
2007	AM0108000	PAE ILHA DO ARAMAÇA	82.98	1.94	2%	81.04	98%	81.04	98%	0.19	0%	0%	80.84	97%
2007	AM0109000	PAE ANUMAÃ	226.31	18.82	8%	207.50	92%	207.50	92%	3.26	2%	0%	204.24	90%
2007	AM0119000	PDS RIO TACANA	34.98	2.52	7%	32.46	93%	32.46	93%	1.17	4%	1%	31.30	89%
2007	AM0120000	PDS SAMAUMA	360.25	3.77	1%	356.48	99%	356.48	99%	3.03	1%	0%	353.45	98%
2007	AM0121000	PDS PRIMAVERA	184.92	1.36	1%	183.57	99%	183.57	99%	0.28	0%	0%	183.28	99%
2007	AM0123000	PCA SÃO PAULO DE OLIVENÇA	28.37	8.13	29%	20.24	71%	20.24	71%	0.00	0%	0%	20.24	71%
2007	MA0758000	PA JEOVAH	31.75	31.75	100%	0.00	0%	0.00	0%	0.00		0%	0.00	0%

2007	MA0768000	PA SÃO JOSÉ / SATUBINHA	6.26	6.26	100%	0.00	0%	0.00	0%	0.00		0%	0.00	0%
2007	MA0771000	PA PADRE PAULO	37.18	35.06	94%	2.12	6%	2.12	6%	0.09	4%	1%	2.03	5%
2007	MA0772000	PA LAGOA DOS PATOS	17.13	8.89	52%	8.24	48%	8.24	48%	2.00	24%	4%	6.24	36%
2007	MA0773000	PA DALBAM	24.72	24.72	100%	0.00	0%	0.00	0%	0.00		0%	0.00	0%
2007	MA0945000	PA VILA NOVA / ÁGUA BRANCA	1.47	0.01	1%	1.46	99%	1.46	99%	0.14	10%	2%	1.32	90%
2007	MB0497000	PA RECANTO DA IARA	3.77	1.81	48%	1.97	52%	1.97	52%	0.38	19%	3%	1.59	42%
2007	MB0499000	PA ANTONIO NONATO	45.74	13.81	30%	31.93	70%	31.93	70%	9.57	30%	5%	22.36	49%
2007	MB0500000	PA UNIÃO DA VITÓRIA	10.12	5.05	50%	5.07	50%	5.07	50%	0.94	19%	3%	4.12	41%
2007	MB0503000	PA PROGRESSO	14.59	12.47	85%	2.12	15%	2.12	15%	1.15	54%	9%	0.97	7%
2007	MB0504000	PA BARRA MANSA	157.22	50.53	32%	106.69	68%	106.69	68%	23.67	22%	4%	83.02	53%
2007	MT0807000	PA ZUMBI DOS PALMARES II	65.13	26.26	40%	38.87	60%	38.87	60%	10.98	28%	5%	27.90	43%
2007	MT0815000	PA BRIDÃO BRASILEIRO	187.15	76.97	41%	110.18	59%	110.18	59%	60.88	55%	9%	49.29	26%
2007	MT0816000	PA MIURA	26.05	15.86	61%	10.19	39%	10.19	39%	0.08	1%	0%	10.11	39%
2007	MT0817000	PA BARRA DO MARCO	7.76	5.03	65%	2.73	35%	2.73	35%	0.00	0%	0%	2.73	35%
2007	MT0818000	PA MEDALHA MILAGROSA	278.41	143.3 0	51%	135.11	49%	135.11	49%	36.26	27%	4%	98.85	36%
2007	MT0824000	PDS OLGA BENARIO	14.57	3.97	27%	10.60	73%	10.60	73%	0.46	4%	1%	10.14	70%
2007	MT0827000	PA JAPURANOMANN	287.24	131.2	46%	155.95	54%	155.95	54%	11.56	7%	1%	144.39	50%

				9										
2007	PA0430000	PA PATAUATEUA	27.57	24.08	87%	3.49	13%	3.49	13%	0.00	0%	0%	3.49	13%
2007	RO0163000	PAF JEQUITIBÁ	1371.86	148.3 7	11%	1,223.48	89%	1,223.48	89%	40.34	3%	1%	1,183.14	86%
2007	RO0167000	PA LAMARQUINHA	4.92	1.55	32%	3.37	68%	3.37	68%	2.50	74%	12%	0.86	18%
2007	RO0168000	PDS DOM XAVIER REY	35.13	2.12	6%	33.02	94%	33.02	94%	0.02	0%	0%	33.00	94%
2007	RO0170000	PA NOVO PROGRESSO	4.54	4.04	89%	0.50	11%	0.50	11%	0.00	0%	0%	0.50	11%
2007	RO0171000	PA LAMARCA	10.17	3.90	38%	6.27	62%	6.27	62%	4.09	65%	11%	2.18	21%
2007	RR0055000	PA TALISMÃ	23.07	0.42	2%	22.65	98%	22.65	98%	0.91	4%	1%	21.74	94%
2007	RR0056000	PA NOVA FLORESTA	84.49	0.89	1%	83.61	99%	83.61	99%	5.60	7%	1%	78.00	92%
2007	TO0413000	PA PALMARES	19.14	19.14	100%	0.00	0%	0.00	0%	0.00		0%	0.00	0%
2007	TO0418000	PA MATA AZUL I	42.61	42.14	99%	0.47	1%	0.47	1%	0.00	0%	0%	0.47	1%
2007	TO0425000	PA CORUJA	0.31	0.31	100%	0.00	0%	0.00	0%	0.00			0.00	0%
2008	AC0148000	PA BARRO ALTO	58.84	28.44	48%	30.40	52%	30.40	52%	2.67	9%	2%	27.73	47%
2008	MA0782000	PA MANGAUBA	2.99	1.51	51%	1.47	49%	1.47	49%	0.14	9%	2%	1.33	45%
2008	MA0783000	PA CIGANA/BARRIGUDO /COLONE	1.69	1.44	85%	0.25	15%	0.25	15%	0.00	0%	0%	0.25	15%
2008	MA1030000	PA SÃO FRANCISCO/BOA VIAGEM	12.22	8.21	67%	4.01	33%	4.01	33%	1.15	29%	6%	2.87	23%
2008	MA1031000	PA RENASCER	6.13	1.40	23%	4.73	77%	4.73	77%	0.06	1%	0%	4.67	76%
2008	AM0131000	PAF RIO IÇÁ	473.74	4.80	1%	468.94	99%	468.94	99%	0.07	0%	0%	468.87	99%
2008	MA1032000	PA HORIZONTE AZUL	19.40	3.02	16%	16.38	84%	16.38	84%	0.55	3%	1%	15.83	82%
2008	MA1042000	PA	2.29	2.29	100%	0.00	0%	0.00	0%	0.00		0%	0.00	0%

		CRISTALINA/COLONE												
2008	MB0505000	PA 26 DE MARÇO	101.82	79.25	78%	22.57	22%	22.57	22%	2.98	13%	3%	19.59	19%
2008	PA0458000	PA RIO BUJARU	80.62	71.77	89%	8.86	11%	8.86	11%	0.06	1%	0%	8.79	11%
2008	PA0459000	PA TARIRATEUA	34.36	24.90	72%	9.46	28%	9.46	28%	0.32	3%	1%	9.14	27%
2008	PA0493000	PA LUIS CARLOS PRESTES	12.75	12.75	100%	0.00	0%	0.00	0%	0.00		0%	0.00	0%
2008	PA0494000	PA MARIA BONITA	13.37	12.53	94%	0.84	6%	0.84	6%	0.00	0%	0%	0.84	6%
2008	RO0172000	PA FLOR DO AMAZONAS 1	107.94	31.92	30%	76.02	70%	76.02	70%	8.31	11%	2%	67.71	63%
2008	RO0173000	PA FLOR DO AMAZONAS 2	51.64	8.82	17%	42.83	83%	42.83	83%	7.47	17%	3%	35.36	68%
2008	RO0174000	PA FLOR DO AMAZONAS 3	71.00	18.97	27%	52.03	73%	52.03	73%	7.85	15%	3%	44.18	62%
2008	RO0175000	PA NORTE SUL	50.65	22.87	45%	27.78	55%	27.78	55%	13.87	50%	10%	13.91	27%
2008	RO0176000	PA NORTE SUL I	13.29	4.61	35%	8.68	65%	8.68	65%	3.72	43%	9%	4.95	37%
2008	RO0177000	PA PORTO MURTINHO	17.14	13.14	77%	4.00	23%	4.00	23%	0.46	12%	2%	3.54	21%
2008	RO0178000	PA FLOR DO AMAZONAS 4	58.20	18.56	32%	39.63	68%	39.63	68%	3.89	10%	2%	35.74	61%
2008	RO0179000	PA CAMPO NOVO	6.43	4.27	66%	2.15	34%	2.15	34%	0.99	46%	9%	1.16	18%
2008	RO0180000	PA RABO DO TAMANDUÁ	39.84	30.80	77%	9.04	23%	9.04	23%	1.01	11%	2%	8.04	20%
2008	RO0181000	PA 13 DE OUTUBRO	5.17	4.31	83%	0.86	17%	0.86	17%	0.16	18%	4%	0.71	14%
2008	RO0182000	PA UNIÃO I	19.64	16.89	86%	2.75	14%	2.75	14%	0.25	9%	2%	2.50	13%
2008	RO0183000	PA CALADINHO	8.08	5.68	70%	2.40	30%	2.40	30%	0.18	7%	1%	2.22	27%
2008	TO0435000	PA CANOA	65.97	56.44	86%	9.53	14%	9.53	14%	0.00	0%	0%	9.53	14%

2008	AC0149000	PAE TRIUNFO/PORONGABA	244.73	15.97	7%	228.76	93%	228.76	93%	1.58	1%	0%	227.19	93%
2008	AM0132000	PAE VILA ALTEROSA DO JUI	507.41	18.24	4%	489.17	96%	489.17	96%	0.54	0%	0%	488.63	96%
2008	AM0133000	PAE GUARANÍ	98.03	19.26	20%	78.78	80%	78.78	80%	0.47	1%	0%	78.31	80%
2008	PA0388000	PAE COMPLEXO MARACUJÁ	7.60	0.15	2%	7.45	98%	7.45	98%	0.00	0%	0%	7.45	98%
2008	PA0436000	PAE ILHA SANTA MARIA	11.28	0.00	0%	11.28	100%	11.28	100%	0.00	0%	0%	11.28	100%
2008	PA0437000	PAE ILHA MARITUBINHA	43.11	0.00	0%	43.11	100%	43.11	100%	0.00	0%	0%	43.11	100%
2008	PA0438000	PAE ILHA ATATAZINHO	19.49	0.36	2%	19.13	98%	19.13	98%	0.00	0%	0%	19.13	98%
2008	PA0439000	PAE ILHA CAJUUBINHA	19.92	0.00	0%	19.92	100%	19.92	100%	0.00	0%	0%	19.92	100%
2008	PA0441000	PAE ILHA SANTA CATARINA	3.95	0.00	0%	3.95	100%	3.95	100%	0.00	0%	0%	3.95	100%
2008	PA0442000	PAE ILHA TRACUATEUA	8.98	0.71	8%	8.26	92%	8.26	92%	0.00	0%	0%	8.26	92%
2008	PA0443000	PAE ILHA BELA PATRIA	4.08	0.06	1%	4.02	99%	4.02	99%	0.00	0%	0%	4.02	99%
2008	PA0444000	PAE ILHA SÃO RAIMUNDO	29.46	1.23	4%	28.23	96%	28.23	96%	0.26	1%	0%	27.97	95%
2008	PA0445000	PAE ILHA SORVA	14.35	0.05	0%	14.30	100%	14.30	100%	0.00	0%	0%	14.30	100%
2008	PA0447000	PAE ILHA SÃO JOÃO I	38.60	1.34	3%	37.25	97%	37.25	97%	0.30	1%	0%	36.95	96%
2008	PA0448000	PAE ILHA CALHEIRA	23.52	1.95	8%	21.57	92%	21.57	92%	0.00	0%	0%	21.57	92%

2008	PA0449000	PAE ILHA MOSSORO	7.54	0.00	0%	7.54	100%	7.54	100%	0.00	0%	0%	7.54	100%
2008	PA0450000	PAE ILHA SAMANAJÓS	25.70	0.04	0%	25.66	100%	25.66	100%	0.00	0%	0%	25.66	100%
2008	PA0451000	PAE ILHA ARARAS	2.59	0.00	0%	2.59	100%	2.59	100%	0.00	0%	0%	2.59	100%
2008	PA0452000	PAE ILHA SAO PEDRO E BARBOSA	6.88	0.07	1%	6.81	99%	6.81	99%	0.00	0%	0%	6.81	99%
2008	PA0453000	PAE ILHA GRANDE DO LAGUNA	1721.86	81.71	5%	1,640.15	95%	1,640.15	95%	2.98	0%	0%	1,637.18	95%
2008	PA0454000	PAE ILHA SANTA MARIA I	10.99	0.00	0%	10.99	100%	10.99	100%	0.00	0%	0%	10.99	100%
2008	PA0455000	PAE ILHA MACUJUBIM	116.69	5.05	4%	111.64	96%	111.64	96%	0.07	0%	0%	111.56	96%
2008	PA0456000	PAE ILHA ATURIÁ	132.05	0.41	0%	131.63	100%	131.63	100%	0.00	0%	0%	131.63	100%
2008	PA0457000	PAE ILHA DOS MACACOS	1222.28	83.39	7%	1,138.89	93%	1,138.89	93%	0.23	0%	0%	1,138.66	93%
2008	PA0461000	PAE ILHA CARARUÁ-GRANDE	64.33	0.19	0%	64.15	100%	64.15	100%	0.00	0%	0%	64.15	100%
2008	PA0462000	PAE LUZ DA VIDA	33.01	0.00	0%	33.01	100%	33.01	100%	0.00	0%	0%	33.01	100%
2008	PA0464000	PAE ILHA JURUPARI	43.57	1.66	4%	41.91	96%	41.91	96%	0.00	0%	0%	41.91	96%
2008	PA0465000	PAE ILHA TAQUARI	57.84	1.30	2%	56.54	98%	56.54	98%	0.00	0%	0%	56.54	98%
2008	PA0466000	PAE ILHA ITAPERA	50.69	3.08	6%	47.61	94%	47.61	94%	0.12	0%	0%	47.50	94%
2008	PA0467000	PAE ILHA URUÁ II	27.64	0.05	0%	27.59	100%	27.59	100%	0.00	0%	0%	27.59	100%
2008	PA0468000	PAE ILHA SANTA APOLONIA	15.61	0.59	4%	15.02	96%	15.02	96%	0.00	0%	0%	15.02	96%
2008	PA0469000	PAE ILHA PANACU	38.42	0.00	0%	38.42	100%	38.42	100%	0.00	0%	0%	38.42	100%
2008	PA0470000	PAE ILHA SAPATEIRO	2.19	0.00	0%	2.19	100%	2.19	100%	0.00	0%	0%	2.19	100%

2008	PA0471000	PAE SANTA ROSA DO MARACATI	29.35	0.45	2%	28.90	98%		28.90	98%	0.08	0%	0%	28.82	98%
2008	PA0472000	PAE ILHA SANTA MARIA II	8.37	0.00	0%	8.37	100%		8.37	100%	0.00	0%	0%	8.37	100%
2008	PA0473000	PAE ILHA SANTA MARIA III	0.68	0.00	0%	0.68	100%		0.68	100%	0.00	0%	0%	0.68	100%
2008	PA0474000	PAE ILHA MARIANA	2.22	0.00	0%	2.22	100%		2.22	100%	0.00	0%	0%	2.22	100%
2008	PA0475000	PAE ILHA NOSSA SENHORA DO LIVRAMENTO I	21.28	0.51	2%	20.77	98%		20.77	98%	0.00	0%	0%	20.77	98%
2008	PA0477000	PAE ILHA ENTRE-ILHAS	0.23	0.00	0%	0.23	100%		0.23	100%	0.00	0%	0%	0.23	100%
2008	PA0478000	PAE ILHA JACAREUÁ	4.46	0.03	1%	4.43	99%		4.43	99%	0.04	1%	0%	4.39	98%
2008	PA0479000	PAE ILHA BIRIBATUBA	2.26	0.04	2%	2.22	98%		2.22	98%	0.00	0%	0%	2.22	98%
2008	PA0480000	PAE ILHA DO MEIO	257.76	0.04	0%	257.72	100%		257.72	100%	0.00	0%	0%	257.72	100%
2008	PA0481000	PAE ILHA MARACUJÁ I	215.43	0.00	0%	215.43	100%		215.43	100%	0.00	0%	0%	215.43	100%
2008	PA0482000	PAE ILHA RASA	24.79	0.00	0%	24.79	100%		24.79	100%	0.00	0%	0%	24.79	100%
2008	PA0483000	PAE ILHA DO TELES	34.41	0.00	0%	34.41	100%		34.41	100%	0.00	0%	0%	34.41	100%
2008	PA0484000	PAE ILHA CALDEIRÃO	74.13	0.00	0%	74.13	100%		74.13	100%	0.00	0%	0%	74.13	100%
2008	PA0485000	PAE ILHA CONCEIÇÃO I	125.80	0.84	1%	124.96	99%		124.96	99%	0.00	0%	0%	124.96	99%
2008	PA0486000	PAE ILHA SALVADOR	256.75	0.02	0%	256.73	100%		256.73	100%	0.00	0%	0%	256.73	100%
2008	PA0487000	PAE ILHA QUEIMADA	613.25	1.83	0%	611.42	100%		611.42	100%	0.00	0%	0%	611.42	100%
2008	PA0488000	PAE ILHA DOS	126.39	0.00	0%	126.39	100%		126.39	100%	0.00	0%	0%	126.39	100%

		CARÁS													
2008	PA0489000	PAE ILHA PANEMA	70.01	0.01	0%	70.00	100%	70.00	100%	0.00	0%	0%	70.00	100%	
2008	PA0490000	PAE ILHA JURUPARI I	297.91	5.51	2%	292.40	98%	292.40	98%	0.54	0%	0%	291.87	98%	
2008	PA0491000	PAE ILHA BAIANO	35.90	0.00	0%	35.90	100%	35.90	100%	0.00	0%	0%	35.90	100%	
2008	PA0492000	PAE ILHA TRAMBIOCA	70.19	18.66	27%	51.53	73%	51.53	73%	0.15	0%	0%	51.38	73%	
2008	PA0495000	PAE ILHA PIRARUAIA	5.12	0.22	4%	4.90	96%	4.90	96%	0.00	0%	0%	4.90	96%	
2008	PA0496000	PAE ILHA IOIÁS	2.12	0.00	0%	2.12	100%	2.12	100%	0.00	0%	0%	2.12	100%	
2008	PA0497000	PAE ILHA DO CABO DICO	5.37	0.00	0%	5.37	100%	5.37	100%	0.00	0%	0%	5.37	100%	
2008	PA0498000	PAE TIRIRICA	26.29	1.99	8%	24.30	92%	24.30	92%	0.17	1%	0%	24.13	92%	
2008	PA0499000	PAE ILHA DO BOI	24.38	0.00	0%	24.38	100%	24.38	100%	0.00	0%	0%	24.38	100%	
2008	PA0500000	PAE ILHA JAPIIM SECO	20.74	0.00	0%	20.74	100%	20.74	100%	0.00	0%	0%	20.74	100%	
2008	PA0501000	PAE ILHA MURUMURU I	52.39	0.80	2%	51.59	98%	51.59	98%	0.00	0%	0%	51.59	98%	
2008	PA0502000	PAE ILHA DO TANGARAZINHO	18.52	0.31	2%	18.21	98%	18.21	98%	0.00	0%	0%	18.21	98%	
2008	PA0503000	PAE ILHA FURO MUANÁ	21.79	0.17	1%	21.62	99%	21.62	99%	0.00	0%	0%	21.62	99%	
2008	PA0504000	PAE COMPLEXO BATUQUE	1.61	0.00	0%	1.61	100%	1.61	100%	0.00	0%	0%	1.61	100%	
2008	AC0147000	PDS NOVA BAIXA VERDE	50.84	27.21	54%	23.64	46%	23.64	46%	1.07	5%	1%	22.56	44%	
2008	AM0125000	PDS COSTA DO CALDEIRÃO	20.14	3.19	16%	16.95	84%	16.95	84%	0.09	1%	0%	16.86	84%	
2008	AM0126000	PDS LAGO DO	36.03	8.57	24%	27.46	76%	27.46	76%	0.46	2%	0%	27.00	75%	

		TUCUNARÉ													
2008	AM0134000	PDS COSTA DO IRANDUBA	16.63	1.39	8%	15.24	92%		15.24	92%	0.39	3%	1%	14.85	89%
2008	AM0135000	PDS NOVO REMANSO	224.02	133.06	59%	90.96	41%		90.96	41%	5.03	6%	1%	85.94	38%
2008	AM0136000	PDS AMATARÍ	38.54	9.65	25%	28.89	75%		28.89	75%	1.03	4%	1%	27.87	72%
2008	AM0137000	PDS COSTA DA CONCEIÇÃO	117.70	33.49	28%	84.21	72%		84.21	72%	0.32	0%	0%	83.89	71%

Table S7 Total area originally forested and area of forest remaining in the year of official creation of each settlement and in two different years (2005 and 2013) and the annual mean area cleared in two periods in Federal Settlement Projects (PA), Sustainable Development Projects (PDS) and Agro-Extractivist Settlement Projects (PAE) created from 2000 to 2008 (total = 899 settlements)

Year of creation and category	Number analyzed	Total area of forest in km^2 (%)	Remaining forest			Mean clearing per year in km^2	
			In the year of settlement creation in km^2 (%)	In 2005 in km^2 (%)	In 2013 in km^2 (%)	From one year after creation to 2005	From 2006 to 2013 or from one year after creation to 2013[(1)]
**2000**							
PA	59	4,567.1 (100%)	3,130.0 (69%)	2,068.5 (45%)	1,633.5 (36%)	212.3	54.4
PDS	1	484.4 (100%)	483.7 (100%)	482.1 (100%)	481.2 (99%)	0.3	0.1
**2001**							
PA	79	3,216.1 (100%)	2,136.1 (66%)	1,657.0 (52%)	1,028.5 (32%)	119.8	78.6
PAE	3	940.6 (100%)	924.2 (98%)	917.0 (97%)	907.5 (96%)	1.8	1.2
PDS	2	614.8 (100%)	593.1 (96%)	585.2 (95%)	580.2 (94%)	2.0	0.6
**2002**							
PA	60	4,118.2 (100%)	2,168.3 (53%)	1,623.8 (39%)	1,209.4 (29%)	181.5	51.8
PDS	1	35.1 (100%)	27.8 (79%)	27.8 (79%)	27.3 (78%)	0.0	0.1
**2003**							
PA	53	2,905.0 (100%)	2,222.4 (77%)	2,089.0 (72%)	1,765.2 (61%)	66.7	40.5
PAE	1	28.1 (100%)	11.0 (39%)	10.8 (39%)	9.8 (35%)	0.1	0.1
PDS	2	162.8 (100%)	156.2 (96%)	127.0 (78%)	84.6 (52%)	14.6	5.3
**2004**							
PA	75	3,895.2 (100%)	760.0 (20%)	667.6 (17%)	425.4 (11%)	92.4	30.3
PAE	8	11,710.1 (100%)	11,477.7 (98%)	11,475.0 (98%)	11,445.7 (98%)	2.6	3.7

PDS	8	1,986.0 (100%)	1,917.4 (97%)	1,906.2 (96%)	1,815.9 (91%)	11.2	11.3
**2005**							
PA	143	10,955.9 (100%)	6,313.9 (58%)		5,286.2 (48%)		128.5
PAE	30	24,566.9 (100%)	22,685.3 (92%)		22,521.8 (92%)		20.4
PDS	18	11,984.8 (100%)	11,241.0 (94%)		10,674.4 (89%)		70.8
**2006**							
PA	64	5,644.6 (100%)	4,019.0 (71%)		3,397.2 (60%)		88.8
PAE	123	12,427.5 (100%)	11,564.3 (93%)		11,466.9 (92%)		13.9
PDS	29	10,940.4 (100%)	10,234.3 (94%)		9,679.5 (88%)		79.3
**2007**							
PA	26	1,418.5 (100%)	733.0 (52%)		562.2 (40%)		28.5
PAE	10	7,667.7 (100%)	7,601.4 (99%)		7,580.7 (99%)		3.4
PDS	8	1,241.7 (100%)	1,192.9 (96%)		1,169.2 (94%)		4.0
**2008**							
PA	25	861.5 (100%)	376.7 (44%)		320.6 (37%)		11.2
PAE	64	7,104.3 (100%)	6,836.0 (96%)		6,828.5 (96%)		1.5
PDS	7	503.9 (100%)	287.4 (57%)		279.0 (55%)		1.7

(1) In the case of settlements created between 2006 and 2008, we considered the period from the year of creation through 2013.
